# Glucocorticoid receptor Thr524 phosphorylation by MINK1 induces interactions with 14-3-3 protein regulators

**DOI:** 10.1016/j.jbc.2021.100551

**Published:** 2021-03-17

**Authors:** Claire C. Munier, Leonardo De Maria, Karl Edman, Anders Gunnarsson, Marianna Longo, Carol MacKintosh, Saleha Patel, Arjan Snijder, Lisa Wissler, Luc Brunsveld, Christian Ottmann, Matthew W.D. Perry

**Affiliations:** 1 Research and Early Development, Respiratory & Immunology, BioPharmaceuticals R&D, AstraZeneca, Gothenburg, Sweden; 2 Laboratory of Chemical Biology, Department of Biomedical Engineering and Institute for Complex Molecular Systems, Technische Universiteit Eindhoven, Eindhoven, The Netherlands; 3 Discovery Sciences, BioPharmaceuticals R&D, AstraZeneca, Gothenburg, Sweden; 4 Division of Cell and Developmental Biology (C.M.), College of Life Sciences, University of Dundee, Dundee, Scotland, UK; 5 Discovery Biology, Discovery Sciences, R&D, AstraZeneca, Cambridge, UK

**Keywords:** 14-3-3 protein, glucocorticoid receptor, MINK1 kinase, nuclear receptor, phosphorylation, protein–protein interaction, Co-IP, co-immunoprecipitation, DBD, DNA-binding domain, DCM, dichloromethane, DIPEA, N,N-diisopropylethylamine, DMF, dimethylformamide, ERα, estrogen receptor α, ERRγ, estrogen-related receptor γ, FP, fluorescence polarization, GR, glucocorticoid receptor, HATU, 1-[bis(dimethylamino)methylene]-1H-1,2,3-triazolo[4,5-b]pyridinium 3-oxid hexafluorophosphate, KPSI, kilopounds per square inch, LBD, ligand-binding domain, MINK1, misshapen-like kinase 1, NMP, N-methyl-2-pyrrolidone, NTD, N-terminal domain, PDB, Protein Data Bank, ROCK1, Rho-associated coiled-coil containing protein kinase 1, SPR, surface plasmon resonance, TBST, tris-buffered saline with Polysorbate 20, TCEP, tris(2-carboxyethyl)phosphine

## Abstract

The glucocorticoid receptor (GR) is a ligand-dependent transcription factor that plays a central role in inflammation. The GR activity is also modulated *via* protein–protein interactions, including binding of 14-3-3 proteins induced by GR phosphorylation. However, the specific phosphorylation sites on the GR that trigger these interactions and their functional consequences are less clear. Hence, we sought to examine this system in more detail. We used phosphorylated GR peptides, biophysical studies, and X-ray crystallography to identify key residues within the ligand-binding domain of the GR, T524 and S617, whose phosphorylation results in binding of the representative 14-3-3 protein 14-3-3ζ. A kinase screen identified misshapen-like kinase 1 (MINK1) as responsible for phosphorylating T524 and Rho-associated protein kinase 1 for phosphorylating S617; cell-based approaches confirmed the importance of both GR phosphosites and MINK1 but not Rho-associated protein kinase 1 alone in inducing GR–14-3-3 binding. Together our results provide molecular-level insight into 14-3-3-mediated regulation of the GR and highlight both MINK1 and the GR–14-3-3 axis as potential targets for future therapeutic intervention.

The glucocorticoid receptor (GR) is a ligand-dependent transcription factor that belongs to the superfamily of nuclear hormone receptors, a highly conserved ligandable protein family. Ubiquitously expressed throughout the human body, the GR regulates the expression of thousands of genes that control a wide range of fundamental processes (1). The role of the GR is to mediate the actions of glucocorticoids, steroid hormones produced by the adrenal cortex and under tight regulation by the hypothalamic–pituitary–adrenal axis (2). GR agonists are widely prescribed drugs used in the treatment of inflammatory and immunological conditions as well as the treatment of some cancers. By their nature in affecting the transcription of many genes, GR agonists are associated with multiple effects, both beneficial and undesired, thus understanding the underlying signaling network of the GR is of great importance (2, 3, 4).

The GR is divided into three major domains: the N-terminal domain (NTD), the DNA-binding domain (DBD), and the C-terminal ligand-binding domain (LBD) with a short hinge region between the DBD and LBD. Upon translocation to the nucleus, the GR binds to GR binding DNA sequences and, as a scaffolding protein, brings different cofactors and other transcription factors together to build a transcriptional complex that regulates gene expression (1, 5, 6). In addition, the GR can take part in nongenomic signaling (5, 6), adding another layer of complexity, while the GR turnover is regulated by the ubiquitin–proteasome pathway (7).

14-3-3 proteins form a family of eukaryotic regulatory proteins that act as dimers, principally heterodimers, which recognize and bind to specific pairs of phosphorylated serine/threonine residues, thus forming part of a regulatory system with kinases and phosphatases (8, 9). 14-3-3 proteins are involved in regulating a large number of cellular processes, such as cell cycle progression, apoptosis, intracellular protein trafficking, and signal transduction (8). More than 200 structurally and functionally diverse 14-3-3 protein partners have been identified (10). 14-3-3 has been, for example, reported to interact with numerous nuclear hormone receptors to modulate their activity, including the estrogen receptor α (ERα) (11), estrogen-related receptor γ (ERRγ) (12), and pregnane X receptor (13).

Early evidence for the interaction between the 14-3-3 and GR came from yeast two-hybrid studies (14) and immunoaffinity chromatographic studies on rat liver cytosol (15). The 14-3-3 isoform η has been reported to bind the GR LBD and increase the GR transcriptional activity through blocking the ubiquitin–proteasome GR degradation pathway (14, 16). The 14-3-3 β and γ isoforms have been found to bind the full-length GR and to upregulate the GR activity in a ligand-dependent manner (17). The GR transcriptional activity was, however, repressed by phosphorylation of S134 in a p38 MAPK (p38 mitogen-activated protein kinases)-dependent manner, driving GR interaction with 14-3-3ζ (18). Interestingly, 14-3-3σ has been reported to bind two different portions of the GR: GR S134 or the GR LBD and to antagonize the GR transcriptional activity (17, 19, 20). The interaction between the 14-3-3 and GR has been suspected to play a role in pathological inflammatory disorders (18) and cancer (20).

Given this background, we wanted to explore the role of the GR–14-3-3 protein–protein interaction using a bottom-up molecular approach because information on this level is virtually absent. Identification of the GR residues, whose phosphorylation is recognized by 14-3-3, and the kinases responsible for phosphorylation of these GR sites is crucial to gain this molecular-level insight into the mechanism underlying the 14-3-3-mediated GR regulation. Herein, we explore for the first time this molecular mechanism and report on the phosphosites of the GR that are recognized by 14-3-3. The GR–14-3-3 interaction was first investigated at the GR peptide level and then extended to the GR LBD and finally to full-length proteins in cells to validate the relevance of the peptide studies. We found that T524 of the GR is the most important phosphosite, particularly in association with S617. We also found that the kinase misshapen-like kinase 1 (MINK1), not previously associated with the GR, phosphorylates the key T524 residue and that, in a cellular system, this kinase is required for most of the binding of the GR to 14-3-3. This work thus contributes to unraveling the long-standing question on the 14-3-3 regulation of GR signaling pathways, assessing the GR–14-3-3 protein–protein interaction implication in the disease state and highlighting both MINK1 and the GR–14-3-3 axis as potential targets for future therapeutic intervention.

## Results

### Binding of 14-3-3 to phosphopeptides, centered on putative 14-3-3-binding sites of the GR

**Figure 1:**
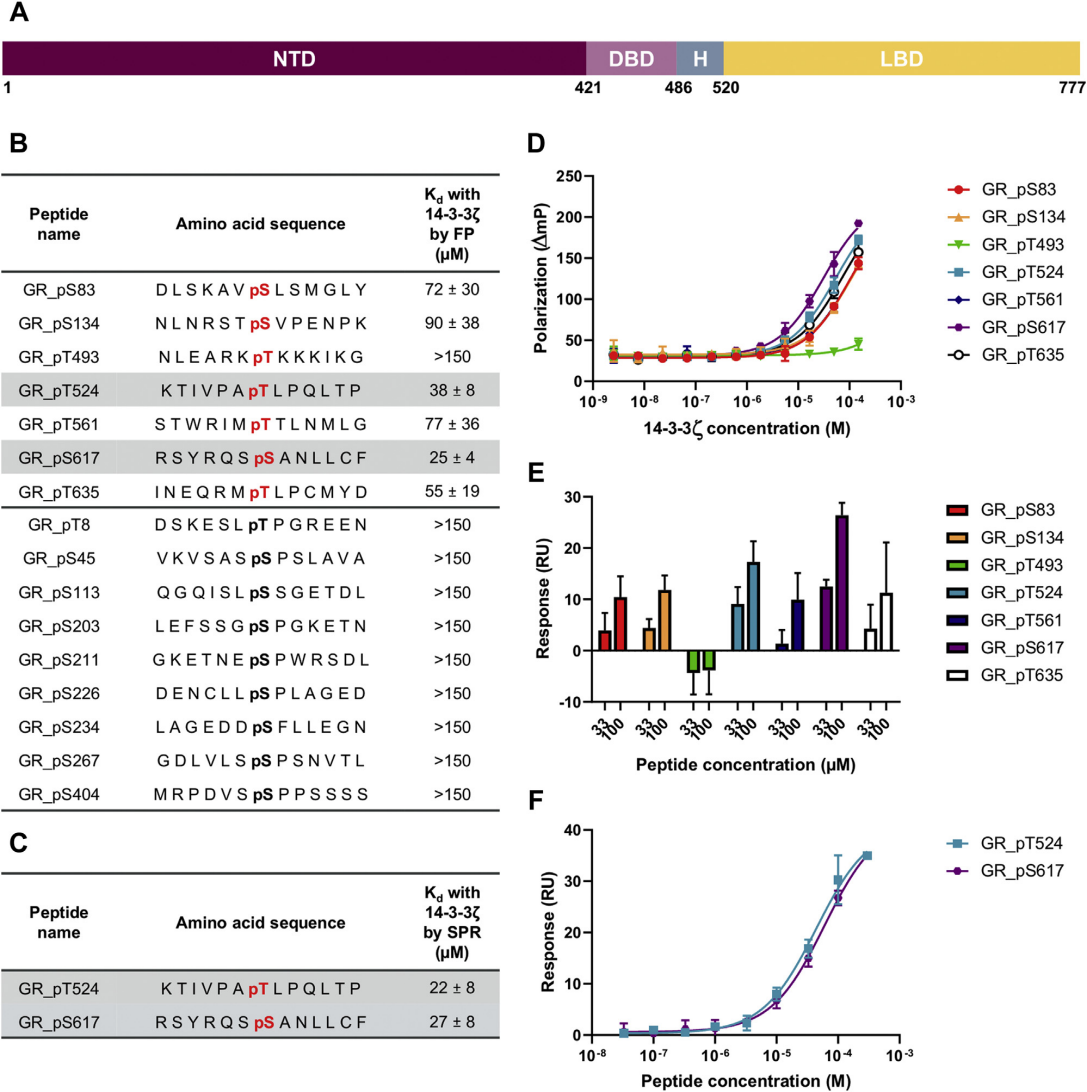
**Interaction of the GR monophosphopeptides with 14-3-3ζ.***A*, schematic representation of the GR sequence. The number of the residues located at the interface of each GR domain is reported below the sequence. *B*, amino acid sequences of the monophosphorylated GR peptides centered on the key residues and their binding affinity (K_d_) measured by FP. Binding sites of the GR from the literature are depicted in *bold black* and prediction in *bold red*. The most potent GR peptides are highlighted in *gray*. *C*, *K*_d_ of the two most potent monophosphorylated GR peptides measured by SPR. *D*, concentration–response curves of FP assays of seven peptides centered on predicted 14-3-3 binding sites with 14-3-3ζ. *E*, affinity of monophosphorylated GR peptides with 14-3-3ζ measured by SPR at two different peptide concentrations (33 and 100 μM). *F*, concentration–response curves of SPR assays of the two most potent peptides with 14-3-3ζ. All measurements were performed as triplicates, and the error bars represent the SD of these three independent experiments. FP, fluorescence polarization; GR, glucocorticoid receptor; SPR, surface plasmon resonance.

Although 14-3-3 proteins can recognize some unphosphorylated sequences in nonphysiological contexts, such as R18 and exoenzyme S (21, 22), the normal recognition elements in binding with 14-3-3 are phosphoserines and phosphothreonines (9). Potential phosphosites on the GR for recognition by 14-3-3 proteins were identified from literature reports and by the use of the 14-3-3-Pred webserver (www.compbio.dundee.ac.uk/1433pred) (23) (Fig. 1*A*). We synthesized 13-mer peptides centered on each of these residues and measured their affinity to 14-3-3ζ and σ by both fluorescence polarization (FP) and surface plasmon resonance (SPR).

Multiple potential phosphosites on the GR have been reported in the literature including T8, S45, S113, S134, S203, S211, S226, S234, S267, and S404 (6, 24). All are part of the NTD and, apart from T8 and S404, they are located within the transactivation domain activation function 1, suggesting a role in the modulation of the GR transcriptional activity (6, 24). Surprisingly, only one of these phosphosites, GR S134, was identified as a putative 14-3-3-binding site by the prediction algorithm. The 13-mer peptide centered on GR S134 was the only phosphopeptide, from this set, to show an interaction with 14-3-3 (*K*_d_ = 90 and 110 μM with 14-3-3ζ and 14-3-3σ, respectively) (Fig. 1*B*).

Seven putative GR phosphorylation sites were identified by the algorithm as candidates for recognition by 14-3-3 proteins: S83, S134, T493, T524, T561, S617, and T635. Six of these 13-mer GR peptides showed binding with 14-3-3ζ and σ, the exception being pT493. Peptides centered on the GR residues pT524 and pS617 were found to be the strongest binders with K_d_s in the low micromolar range (Fig. 1, *B*–*F*, Fig. S1). Of note, these two sites belong to the GR LBD.

### Interaction of 14-3-3 with a dimeric peptide: GR_pT524-pS617

**Figure 2:**
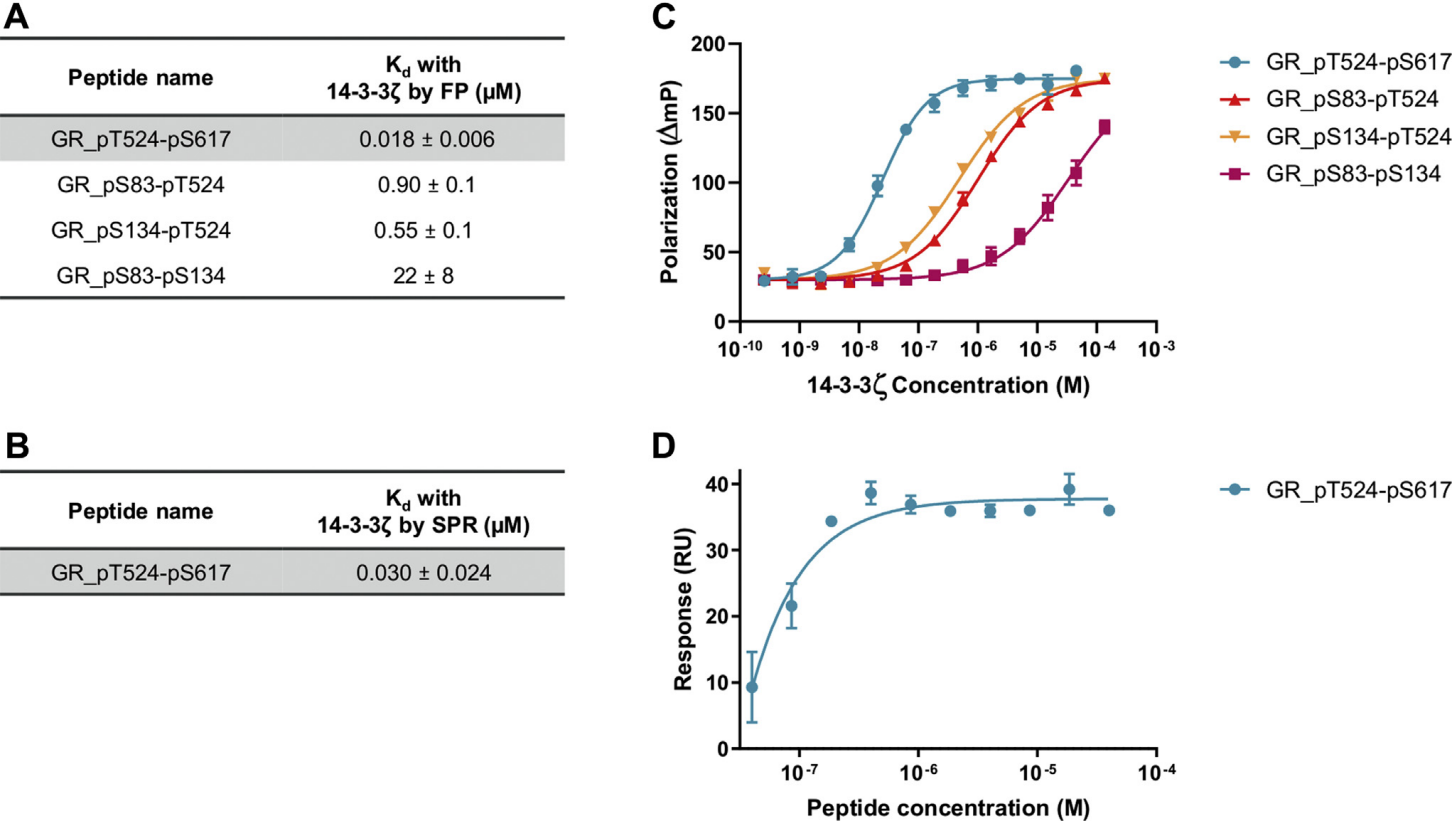
**Interaction of the GR doubly phosphorylated peptides with 14-3-3ζ.***A*, binding affinity (*K*_d_) of the diphosphorylated GR peptides measured by FP. The most potent GR peptide is highlighted in *gray*. *B*, binding affinity (*K*_d_) of GR_pT524-pS617 measured by SPR. *C*, concentration–response curves of FP assays of the four diphosphorylated GR peptides with 14-3-3ζ. *D*, concentration–response curve of SPR assays of GR_pT524-pS617 with 14-3-3ζ. Measurements were performed as triplicates, and the error bars represent the SD of these three independent experiments. FP, fluorescence polarization; GR, glucocorticoid receptor; SPR, surface plasmon resonance.

14-3-3s act as a dimer and thus effectively provide 2 amphipathic binding grooves (8). Many 14-3-3 targets (PKC, C-Raf, CFTR, Gab2) have been shown to interact through tandem phosphosites simultaneously, greatly increasing their binding affinity with 14-3-3 (25, 26, 27, 28). Hence, a dimeric peptide, GR_pT524-pS617, consisting of the two 13-mers GR_pT524 and GR_pS617, identified above, linked by a pentaglycine moiety to permit flexibility (25), was synthesized and tested for binding to 14-3-3ζ. The dimeric peptide displayed a remarkable avidity effect, with a 1000-fold improvement of its binding affinity with 14-3-3ζ, compared with the individual monomers, and a *K*_d_ of 18 nM, one of the highest affinities reported for a 14-3-3 motif (25, 26, 27, 28) (Fig. 2 and Fig. S1).

Previous work has reported 14-3-3 to interact with phosphosites from different domains of its partner proteins (29). Hence, the phosphosites from the GR NTD and GR LBD could also be envisioned to interact simultaneously with a 14-3-3 dimer. We had identified two potential 14-3-3-binding sites in the NTD, S83 and S134, from the bioinformatic analysis. To test this hypothesis, similar 31-mer peptides containing pentaglycine-linked 13-mers, GR_pS83-pT524, GR_pS134-pT524, and GR_pS83-pS134, were synthesized. These peptides also showed significantly increased binding affinity with 14-3-3ζ compared with the monomers, but were at least 15-fold weaker than GR_pT524-pS617 (Fig. 2).

### Identification of GR_pT524-pS617-binding hotspots by mutation and alanine scan

We synthesized 24 peptides where each amino acid of the 31-mer GR_pT524-pS617, except the pentaglycine section, was replaced in turn by alanine. Upon replacement of the phosphothreonine pT524, the binding to 14-3-3 decreased by 6700-fold, from *K*_d_ = 18 nM to *K*_d_ = 120 μM. Mutation of the phosphoserine pS617 had a less marked effect and led to a 72-fold loss in potency resulting in a *K*_d_ of 1.3 μM. The two phosphoresidues are, as expected, the key residues in the binding by 14-3-3 to that doubly phosphorylated peptide. P526 proved to be the most important nonphosphorylated residue with mutation of P526 resulting in an almost 70-fold decrease in potency (*K*_d_ = 1.2 μM). Smaller effects were observed for R614 and K518, resulting in a 12-fold (*K*_d_ = 220 nM) and 6-fold (*K*_d_ = 110 nM) reduction in binding affinity, respectively (Table 1 and Table S1).

**Table 1:** Binding affinity of mutated GR_pT524-pS617

FP, fluorescence polarization.

Binding affinity (*K*_d_ and p*K*_d_) of GR_pT524-pS617, relevant peptides from the alanine scan, and GR_pS524-pS617. The residues mutated into alanine are depicted in bold black and the phosphorylated sites in bold red. Measurements were performed as triplicates.

To further understand the role of pT524, a diphosphoserine analogue (GR_pS524-pS617) was synthesized. This peptide showed an 8-fold reduction in potency (*K*_d_ = 140 nM), revealing the importance of the singular methyl group of T524. Even so, the affinity drop for pS524 to A524 was still more than an order of magnitude larger than the drop for the pS617 to A617 substitution, highlighting the unexpected relative importance of pT524 *versus* pS617. This is particularly surprising, given the similar affinities for the two individual 13-mers but suggests that the artificial boundary conditions of the linked peptides influence one site more than the other. The potent binding of a diphosphorylated GR peptide with 14-3-3 is in line with the thermodynamic model for multivalency in 14-3-3 protein–protein interactions (30). The effect of the individual phosphosites on the strength of GR_pT524-pS617 binding could thus be simulated, assuming an average effective molarity value of 10 mM (Equation S1).

### Crystallization of GR_pT524-pS617, GR_pT524, and GR_pS617 with 14-3-3ζ

The mechanistic details of the GR–14-3-3 interaction were further resolved by determining the crystal structures of 14-3-3ζ in complex with GR_pT524, GR_pS617, and GR_pT524-pS617. GR_pT524 was cocrystallized with 14-3-3ζ, and the crystal structure was solved to a resolution of 2.09 Å [Protein Data Bank (PDB) code 6YO8]. This high-resolution crystal structure allowed us to assign 12 of 13 amino acids of the GR peptide. Two GR_pT524 peptides were found to bind the two respective central binding channels of one 14-3-3 dimer in an extended conformation. Details of the interaction showed that electrostatic effects dominated, with pT524 bound in the positively charged pocket of the 14-3-3 monomer made from K49, R56, and R127. Other polar contacts were observed between the peptide backbone and 14-3-3 residues, such as N173 and N224 (Table 2 and Fig. S2*A*).

**Table 2:** Data collection and refinement statistics (molecular replacement)

Parameters	Binary complex of 14-3-3ζ with human glucocorticoid receptor pT524 peptide	Binary complex of 14-3-3ζ with human glucocorticoid receptor pS617 peptide	Binary complex of 14-3-3ζ with human glucocorticoid receptor pT524 pS617 peptide
PDB entry	6YO8	6YMO	6YOS
Data collection			
Space group	C2	P2_1_2_1_2_1_	P4_3_2_1_2
Cell dimensions			
a, b, c (Å)	158.65, 99.88, 84.81	72.28, 104.35, 112.43	60.21, 60.21, 284.21
α, β, γ (°)	90.00, 93.73, 90.00	90.00, 90.00, 90.00	90.00, 90.00, 90.00
Resolution (Å)	84.47–2.09 (2.12–2.09)	47.33–2.02 (2.02–2.07)	58.90–2.75 (2.90–2.75)
Rmerge	0.06	0.08	0.11
I/σI	9.0 (0.6)	12.07 (0.53)	12.4 (1.3)
Completeness (%)	98.3 (97.8)	99.6 (95.8)	98.3 (94.0)
Redundancy	3.4 (3.4)	6.5 (6.3)	9.4 (6.9)
Refinement			
Resolution (Å)	60.85–2.09	47.33–2.02	58.90–2.75
No. of reflections	76,616	56,503	14,250
Rwork/Rfree	0.224/0.235	0.212/0.228	0.265/0.292
Clashscore	8	2	8
Ramachandran outliers (%)	0.2	0	0
Sidechain outliers (%)	4.0	2.0	5.8
RSRZ outliers (%)	6.0	7.9	7.4
Matthews coefficient (Å^3^/Da)	2.16	2.05	2.49
Solvent content (%)	43.08	39.99	50.60
No. of atoms			
Protein	7693	3769	3690
Ligand/ion	0	25	0
Water	240	236	0
B-factors (Å^2^)			
Protein	76.7	78.2	92.8
Ligand/ion	-	59.5	-
Water	68.0	68.9	-
RMSD			
Bond lengths (Å)	0.72	0.49	0.90
Bond angles (°)	0.69	0.60	0.86
Interface parameters			
Buried surface area (sq Å)	761.8 (46.1%)	525.8 (63.1%)	1247.4 (43.3%)
Interface area (sq Å)	675.7	460.0	1132.5
Delta G (kcal/mol)	−12.2	−3.9	−16.9
Binding energy (kcal/mol)	−18.0	−16.9	−27.7
*p* value	0.5042	0.5829	0.5381
Hydrogen bonds	13	11	24
Effect of crystal contacts	The closest crystallographic neighbor is over 4 Å away and is therefore unlikely to affect the binding of GR_pT524	The closest crystallographic neighbor is over 4 Å away and is therefore unlikely to affect the binding of GR_pS617	The closest crystallographic neighbor is over 4 Å away and is therefore unlikely to affect the binding of GR_pT524-pS617

PDB, Protein Data Bank.

Values in parentheses are for highest resolution shell.

The structure of GR_pS617 with 14-3-3ζ was solved to a resolution of 2.01 Å (PDB code: 6YMO). Similar to GR_pT524, 2 GR_pS617 peptides were observed to interact with one 14-3-3 dimer and pS617 made tight electrostatic contacts with the positively charged pocket of 14-3-3. Interestingly, the electron density allowed us to assign only 6 of 13 amino acids (Table 2 and Fig. S2*B*).

**Figure 3:**
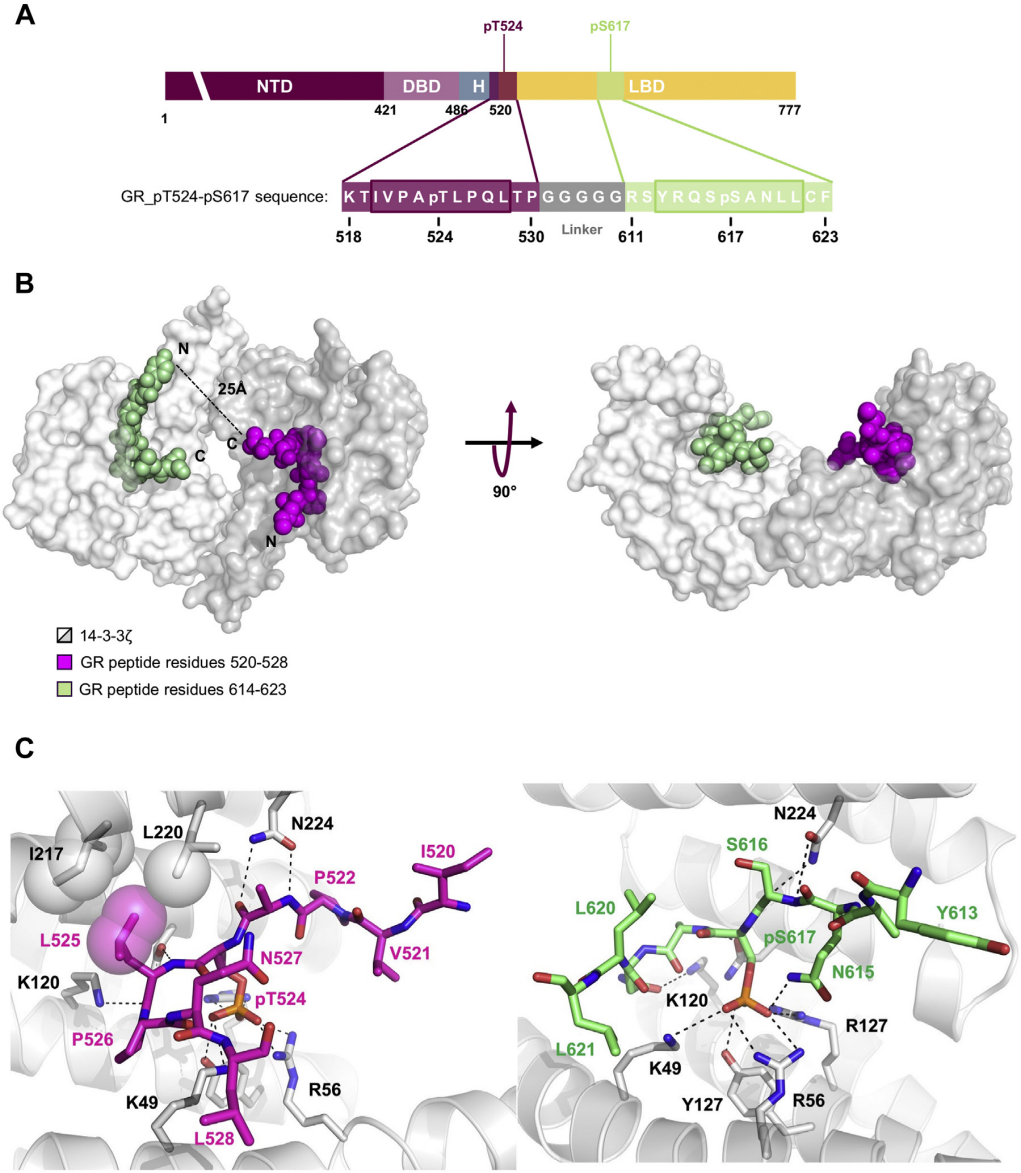
**Crystal structure of GR_pT524-pS617 bound to 14-3-3ζ dimer.***A*, the location within the GR sequence of the two 13-mer peptides centered on pT524 and pS617, respectively, and sequence of GR_pT524-pS617. The framed amino acids were assigned in the X-ray structure. *B*, surface representation of 14-3-3ζ dimer (*white* and *gray solid surface*) complexed with GR_pT524-pS617. Residues 520 to 528 are depicted in *magenta* and residues 614 to 623 in *green*. The *black dashed line* shows amino acid residues, not observed in the electron density, connecting the two binding sites. *C*, details of the interaction between GR_pT524-pS617 and 14-3-3ζ. Polar interactions are depicted as *black dotted lines* and hydrophobic contracts as *magenta* or *white spheres* with a semitransparent surface. GR, glucocorticoid receptor.

GR_pT524-pS617 was cocrystalized with 14-3-3ζ, and the complex was solved to a resolution of 2.75 Å (PDB code 6YOS). One diphosphorylated GR peptide was bound to one 14-3-3ζ dimer. The electron density allowed the assignment of 18 of 31 amino acid residues. The pentaglycine moiety was not visible in the electron density because of its flexibility. However, the distance between L528 and Y613, about 22 Å, is sufficient to be bridged by 9 residues (Fig. 3, *A* and *B*), supported by computational modeling of the pentaglycine linker (chapter 6 of the Supporting information). The crystal structure of GR_pT524-pS617 interacting with 14-3-3ζ was consistent with the structures of GR_T524 and GR_S617 cocrystallized with 14-3-3ζ. The main interactions between the phosphorylated residues and 14-3-3 were in line with the GR_pT524-pS617 alanine scan results and previous published crystal structures of phosphopeptides interacting with 14-3-3 (25, 26). Many residues pointed their side chains away from 14-3-3, consistent with the observation from the alanine scan where mutation had a relatively small impact on the binding affinity with 14-3-3 (Fig. 3*C* and Fig. S2, *C*–*E*). A proline at position +2 relative to the phosphorylated serine/threonine residue, such as P526, is a common feature of 14-3-3-binding motifs 1 and 2, and this amino acid produces a sharp change in the chain direction (9). P526 of GR_pT524-pS617 adopts a *cis*-conformation. On one side, it allows the carbonyl oxygen of L525 at the position +1 to form a hydrogen bond with the amino groups of K120 and N173. On the other side, P526 induces the peptide exit from the binding groove and avoids a clash of the remaining portion of the peptide with S45 and K49. The role of the proline in the structure was supported by the alanine scan. This turn creates a new pocket at the interface of the GR peptide and 14-3-3. Similarly, a second pocket is formed between GR residues 618 to 621 and the second 14-3-3 unit (Table 2 and Fig. 3). Of note, an alternative orientation of GR_pT524-pS617 in the crystal structure with 14-3-3ζ could not be totally excluded and is described in chapter 6 of the Supporting information.

### Assessment of the GR LBD phosphorylation profile by kinase screening

The two potential 14-3-3-binding phosphosites identified here, pT524 and pS617, are situated within the GR LBD. Hence, we aimed to test the binding affinity of the doubly phosphorylated full-length GR LBD with 14-3-3. The kinase(s) responsible for phosphorylation of the GR LBD have not yet been reported. We therefore aimed to identify the relevant kinase(s). The expression of the WT human GR LBD from *Escherichia coli* was unsuccessful because of solubility problems. Switching to the GR LBD mutant F602S (31) allowed us to express sufficient GR F602S N514-K777, in the presence of dexamethasone to be purified and used as a substrate for candidate kinases. The phosphorylation profile of the GR LBD was determined in a radiometric protein kinase filter-binding assay (performed by ProQinase GmbH, Germany) in which the activity of 245 serine/threonine kinases was measured (Fig. S3). This phosphorylation profiling showed a differential ability of the GR LBD to act as a substrate for the tested kinases (Fig. S4). We observed that 38 kinases displayed an activity ratio above 3 with 12 kinases above 5. MST2, MAP4K4, MST1, and TAOK2 were the kinases with the highest ratio, 19, 16, 14, and 13, respectively (Table S2). All of them belong to the STE20 kinase family, being upstream activators of the p38 mitogen-activated protein kinases pathways. The 12 kinases with the highest activity ratios were selected for a follow-up assay, and the phosphorylation profile of the GR LBD by the 12 selected kinases was determined. MAP4K4, MINK1, MST1, MST2, and Rho-associated protein kinase 1 (ROCK1) were observed to phosphorylate the GR LBD (Fig. S5). The varying response may be due to the activity rate of the particular kinases having different kinetics under the chosen conditions and since the ATP concentration was fixed at 1 μM, regardless of the ATP-Km of each kinase.

Phosphorylation of the GR LBD by MAP4K4, MINK1, MST1, MST2, and ROCK1 followed by enzyme digestion and analysis by MS revealed different phosphorylation patterns (Table 3). MAP4K4 showed monophosphorylation and diphosphorylation at T519 and T562. MST1 and MST2 both phosphorylated T562, T668, S682, and S746; each also phosphorylated additional sites, although these sites were markedly weaker in the LC-MS analysis. MST2 showed a wider phosphorylation pattern than MST1 with up to seven phosphorylations observed. The two sites we had identified as most promising for 14-3-3 recognition (T524 and S617) were phosphorylated by MINK1 and ROCK1, respectively. In particular, ROCK1 phosphorylated two different sites, T519 and S617, while MINK1 gave mainly monophosphorylation and diphosphorylation of T524 and T562, with traces of phosphorylation at T635 (Table 3 and Fig. S6). Thus, the screening showed that MINK1 and ROCK1 can phosphorylate GR T524 and S617, respectively, *in vitro*.

**Table 3:** Phosphorylation of the GR LBD by five kinases identified from the kinase screen

GR, glucocorticoid receptor; LBD, ligand-binding domain; MINK1, misshapen-like kinase 1; ROCK1, Rho-associated protein kinase 1.

For each kinase, intact mass and peptide mapping enable the identification of the phosphorylation state and phosphorylation sites. The kinases further investigated are highlighted in gray.

### MINK1 phosphorylates the GR LBD driving the interaction with 14-3-3

**Figure 4:**
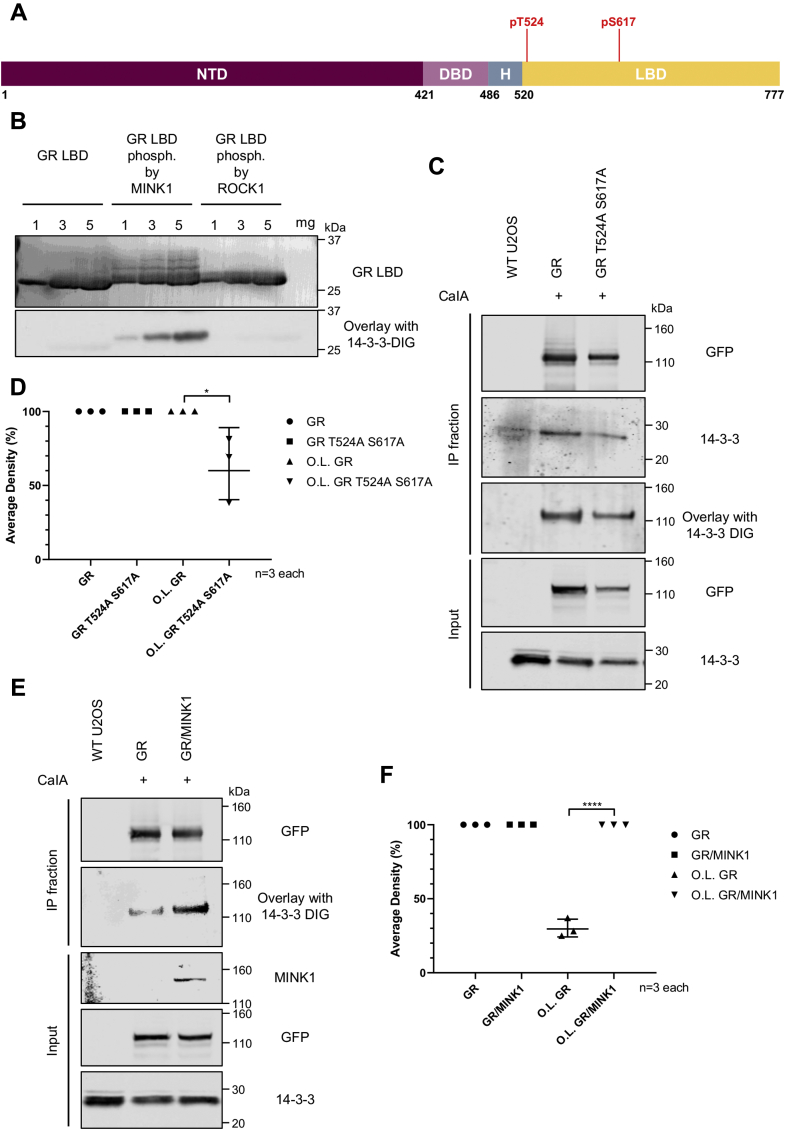
**Interaction of the GR LBD and full-length GR with pan 14-3-3.***A*, the location within the GR sequence of pT524 and pS617. *B*, far-Western blotting overlay of the GR LBD phosphorylated by MINK1 or ROCK1 with BMH1-BMH2-digoxigenin. Unphosphorylated and *in vitro* phosphorylated GR LBDs were detected using anti-6X His tag antibody. GR LBD-bound 14-3-3 proteins were detected using anti-DIG antibody. *C*, U2OS cells were transfected with GFP-GR or GFP-GR T524A S617A plasmids and stimulated with calyculin A. Cell lysates were immunoprecipitated with GFP-Trap beads. The GFP-GR and GFP-GR mutant were detected using the anti-GFP antibody, and GR-associated 14-3-3 was detected using anti-pan 14-3-3 antibody. Far-Western blotting overlay was performed by incubation of the GR-containing membrane with BMH1-BMH2-DIG and subsequent detection of GR-bound 14-3-3 protein using the anti-DIG antibody. *D*, quantification of three independent experiments. *E*, U2OS cells were transfected with GFP-GR or cotransfected with GFP-GR and FLAG-MINK1 plasmids and treated as mentioned previously. FLAG-MINK1 was detected using the anti-MINK1 antibody. Far-Western blotting overlay was performed as mentioned previously. *F*, quantification of three independent experiments. Data are normalized to the amount of GFP-GR and GFP-GR mutant from the IP (immunoprecipitation) fraction. The error bars represent the SD of the three independent assays. All *p* values were obtained using the *t* test. ∗*p* < 0.05, ∗∗∗∗*p* < 0.0001. CaIA, calyculin A; GR, glucocorticoid receptor; LBD, ligand-binding domain; MINK1, misshapen-like kinase 1; OL, overlay; ROCK1, Rho-associated protein kinase 1.

The ability of the GR LBD, phosphorylated by either MINK1 or ROCK1, to interact with 14-3-3 was investigated. A far-Western blotting overlay assay was performed where we assessed the capacity of the different phosphorylated GR LBDs, immobilized on a membrane, to bind the two recombinant yeast isoforms of 14-3-3 tagged with digoxigenin (BMH1-BMH2-digoxigenin). In this assay, the GR LBD phosphorylated by MINK1 showed direct interaction with 14-3-3 in a dose-dependent manner, whereas the GR LBD phosphorylated by ROCK1 and the unphosphorylated GR LBD did not (Fig. 4*B*), highlighting the specificity and sensitivity of the assays toward phosphorylated substrates.

MINK1 phosphorylates the GR LBD at residues T524 and T562 and to a lesser extent T635. T524 and T635 have been identified by the algorithm as plausible 14-3-3-binding sites with T561 but not T562. The 13-mer peptide centered on pT562 showed a weak interaction with 14-3-3ζ (*K*_d_ = 200 μM). Interestingly, the 51-mer peptide GR_pT524-pT562, consisting of the two 13-mer peptides GR_pT524 and GR_pT562 with the native sequence as a linker, did not show a similar avidity effect as previously observed for GR_pT524-pS617. No significant trend has been reported between the effective molarity and the linker length (30, 32). Instead, the effective molarity depends on the flexibility of the linker between the two different binding sites. Thus, the binding affinity of GR_pT524-pT562 with 14-3-3ζ (*K*_d_ = 23 and 15 μM, measured by FP and SPR, respectively) and its low effective molarity (0.2 mM) might suggest a more structured linker. Crystal structure analysis (PDB code: 1M2Z) placed GR T562 in the helix 3, which forms the spine of the protein. It would be unlikely that this helix changes its structure, questioning the ability of GR T562 to interact with 14-3-3 proteins.

### Interaction between full-length GR and 14-3-3

The interaction between the full-length GR and 14-3-3 was investigated in a cellular system to validate the relevance of the peptide studies. In an initial experiment, HEK293 and U2OS cells were transiently transfected with the WT GR and, after overnight starvation, the cells were treated with a panel of stimuli that have previously been shown to promote phosphorylation of 14-3-3-binding sites of other target proteins (33). Results from co-immunoprecipitation (Co-IP) experiments and far-Western blotting overlay assay showed that the GR interacted with 14-3-3 proteins upon stimulation with forskolin (a cell-permeable diterpenoid which activates adenylyl cyclase, increases intracellular cAMP concentration and thus activates PKA) and calyculin A (a cell-permeable potent and selective protein phosphatase inhibitor) (Fig. S7).

The role of the previously identified GR phosphosites in the binding with 14-3-3 was investigated in cells. U2OS cells were transiently transfected with the WT GR or the GR double mutant, T524A S617A, before treatment with calyculin A. Under our experimental conditions, Co-IP and far-Western blotting overlay experiments revealed a significant decrease, about 35%, of the interaction between the 14-3-3 and GR double mutant as compared with the WT GR (Fig. 4, *C* and *D*). These results confirm the interaction between the GR and 14-3-3 and highlight the importance of the phosphorylation of the 2 GR residues T524 and S614.

Upon stimulation with forskolin or calyculin A, MINK1 also interacts with 14-3-3 (Fig. S8) (consistent with unpublished data by Gavuthami Murugesan and CM, University of Dundee). Thus, the GR–14-3-3 interaction and the MINK1–14-3-3 interaction are both promoted in parallel in cells stimulated with forskolin and calyculin A (Fig. S7). We established that U2OS cells have a very low endogenous expression of MINK1, as no signal from this kinase could be detected by Western blotting (Fig. S9). Therefore, U2OS cells were either transfected with the WT GR or cotransfected with the WT GR and MINK1. In the absence of further stimulation, no GR–14-3-3 interaction was observed in either cells. Stimulation with calyculin A increased the level of phosphorylated GR by preventing GR dephosphorylation, so that the protein was capable of binding to 14-3-3 in a far-Western blotting overlay. This interaction increased, by about 70%, in the presence of overexpressed MINK1 (Fig. 4, *E* and *F*). Together these results suggest that activated MINK1 kinase phosphorylates the GR and triggers the GR–14-3-3 protein–protein interaction.

## Discussion

In this study, we systematically investigated the GR–14-3-3 interaction at a GR peptide level, using biophysical assays, alanine scanning, and X-ray crystallography. We particularly focused on 14-3-3σ and 14-3-3ζ, two very representative isoforms, because the seven 14-3-3 isoforms hold a high sequence similarity, notably in the amphipathic binding groove (34). Two GR phosphorylation sites, within the GR LBD, were identified as mediating the strongest binding to 14-3-3: T524 and S617. The GR LBD has been previously reported to interact with 14-3-3, using yeast two-hybrid assays, GST pull-down, and Co-IP experiments, while the GRβ isoform, which shares the same NTD and DBD but contains a different LBD, did not (14, 19, 20). 14-3-3-binding sites are mostly located within intrinsically disordered regions or bordering the functional domains (35). Interestingly, the GR residue T524 was anticipated to be within an intrinsically disordered region (globplot.embl.de), and the crystal structure analysis of the GR LBD placed this residue within a random coil (36). S617 was found in a relatively disordered area, that is, the loop between helix 5 and β-sheet 1, but within an ordered domain, which, to the best of our knowledge, might be one of the first examples of a 14-3-3-binding site located in ordered regions. Mechanistically, after phosphorylation, 14-3-3 partners bind in the amphipathic groove of 14-3-3, leading to a disorder-to-order transition, entropically disadvantageous but enthalpically favorable, driven by the formation of charge–charge interactions with the phosphate group and hydrogen bonding to neighboring residues (35).

One site, GR S134, previously reported to be a 14-3-3 recognition site and predicted by the algorithm used in this study, matches the mode-1 binding motif, RSX-pS/T-XP, where X is any amino acid (cysteine excluded) and pS/T is a phosphorylated serine or threonine. Phosphorylation of GR S134, upon oxidative stimuli, was found to enhance GR interaction with 14-3-3 proteins (18). Here, we report that GR_pS134 interacts with the ζ and σ isoforms of 14-3-3 but with a weaker binding affinity than the two identified sites from the GR LBD.

The dimeric GR_pT524-pS617 peptide revealed an impressive avidity gain over the monomeric components with a low nanomolar affinity toward 14-3-3. Examination of the crystal structure of the GR LBD showed that T524 and S617 are both solvent exposed, and that the distance between these two residues in the crystal structure is 37 Å, closely corresponding to the distance between the two phospho-binding sites of 14-3-3. Other steroid hormone receptors, however, such as the androgen and the mineralocorticoid receptors, undergo a N–C interaction for complete transcriptional activity (37, 38). The full-length GR has not been crystallized yet, and an interdomain interaction through 14-3-3 binding, namely an interaction between the NTD, such as S134, and the LBD of the GR, could be envisioned. Our biophysical assays showed that the affinity of GR_pS134-pT524, although 15-fold weaker than GR_pT524-pS617, is still in the submicromolar range (*K*_d_ = 0.55 μM), which cannot exclude a potential interaction of GR pS134 with 14-3-3. In addition, cell-based approaches revealed that the affinity between the GR double mutant, T524A S617A, and 14-3-3 decreased by 35% as compared with the WT GR, but was not abolished. These results suggest that 14-3-3 interacts with the GR through multiple sites. Further experiments would be necessary to fully quantify the individual effect of the GR phosphosites in 14-3-3 binding with the full-length GR and their biological role by monitoring GR translocation, GR transcriptional activity, or GR protein level.

14-3-3 has been shown to interact with various nuclear hormone receptors and modulate their activity, adding another layer of regulation beyond ligand-driven activation. Interaction of 14-3-3 with ERRγ, upon S179 phosphorylation, favors ERRγ cytoplasmic localization and alters its transcriptional activity and its ability to promote hepatic gluconeogenesis (12). Interaction of 14-3-3 with ERα, upon T594 phosphorylation, inhibits ERα dimerization and transactivation (11). Interaction of 14-3-3 with the pregnane X receptor leads to the overexpression of P-glycoprotein (also known as multidrug resistance protein 1) (13). Previous studies on GR–14-3-3 protein–protein interaction have reported on the 14-3-3 modulation of the GR activity, with different consequences assigned, including the GR translocation and GR transcriptional activity (14, 16, 17, 18, 19, 20). These studies focused on distinct 14-3-3 isoforms and different cellular contexts, which could explain the seemingly conflicting results. The 14-3-3 isoforms indeed interact with different protein partners and have different affinities, distinct *in vivo* effects on targets, and a specific tissue distribution (39, 40, 41). The GR has been reported to dimerize through amino acids found in the GR DBD and GR LBD such as residues P625 and I628 (42, 43). These two residues are closely located to the 14-3-3-binding site of the GR, S617, and it is enticing to speculate that phosphorylation of S617 and interaction with 14-3-3 would negatively impact GR dimerization. To the best of our knowledge, however, no study on the role of 14-3-3 in GR dimerization has been reported. Co-crystallization of GR_pT524-pS617 with 14-3-3ζ led to the identification of two new binding pockets created at the interface of the GR peptide and 14-3-3 protein. It is enticing to speculate that these pockets (pocket size estimation of 390 Å^3^) could be exploited to find a molecule that would form positive interactions with each partner, acting as a “molecular glue” to stabilize the GR–14-3-3 protein–protein interaction. Such a tool compound could play a significant role in the quest to understand the physiological role of the GR–14-3-3 protein–protein interaction.

It is of great interest to fully unravel the underlying signaling network of the GR because of the enormous importance of GR drugs and their widespread use, despite their many side effects. Collectively, our results contribute to answering the long-standing question on the 14-3-3 regulation of GR signaling pathways and highlight both MINK1 and the GR–14-3-3 axis as potential targets for future therapeutic intervention.

## Experimental procedures

### Material, instruments, reagents, antibodies, and plasmids

Detailed information is provided in Supporting information.

### Peptide synthesis

#### General protocol for solid-phase peptide synthesis

The peptides were synthesized *via* Fmoc/tBu solid-phase peptide. The first amino acid was attached to the 2-Chlorotrityl chloride resin following method A to reach a loading of 0.66 mmol/g. The peptide chains were then assembled according to method B. The peptides were then labeled by either addition of an FITC tag (method C) or N-terminal acetylation (method D) before being cleaved from the resin, deprotected according to method E and purified.

#### Method A: Attachment of the first amino acid on the 2-chlorotrityl chloride resin

2-Chlorotrityl chloride resin (0.3–0.8 meq/g, final loading 0.66 mmol/g) was swollen in dichloromethane (DCM) for 10 min, and then the solvent was drained. The first amino acid (1 eq) was dissolved in DCM (1 ml), and N,N-diisopropylethylamine (DIPEA) (3 eq) was added. The solution was added to the resin and stirred at room temperature (RT) under nitrogen bubbling. After 10 min, DIPEA (7 eq) was added to the resin and the reaction mixture was stirred for 40 min. MeOH (0.8 μl/mg of resin) was added to the resin and was stirred for 10 min. The mixture was drained, and the resin was washed with dimethylformamide (DMF) (3×) and DCM (3×).

#### Method B1: Automated Fmoc solid-phase peptide synthesis, Biotage Initiator + Alstra automated microwave peptide synthesizer

The resin was swollen with DMF (2×), and the Fmoc Nα-protecting group was removed with 20% piperidine in DMF (2×; 3 min then 10 min) at RT. The amino acids dissolved in DMF (0.3 M) were repeatedly coupled with 1-[bis(dimethylamino)methylene]-1H-1,2,3-triazolo[4,5-b]pyridinium 3-oxid hexafluorophosphate (HATU) (3 eq) in N-methyl-2-pyrrolidone (NMP) (0.5 M) and DIPEA (6 eq) in NMP (2 M) at RT for 45 or 60 min. The resin was finally washed with DCM (6×).

#### Method B2: Automated Fmoc solid-phase peptide synthesis, Biotage Syro II automated parallel peptide synthesizer

The resin was swollen with DMF (6×), and the Fmoc Nα-protecting group was removed with 40% piperidine in DMF (3 min) followed by 20% piperidine in DMF (10 min) at RT. The amino acids (4 eq) dissolved in DMF (0.5 M) were repeatedly coupled with HATU (4 eq) in DMF (0.48 M) and DIPEA (8 eq) in NMP (2 M) at RT for 40 min. The resin was finally washed with DCM (3×), MeOH (3×), and Et2O (3×).

#### Method C: Addition of an FITC tag at the N-terminal position

After the final Fmoc deprotection, the resin was swollen with DCM (2×). In the dark, FITC (3 eq) was dissolved in DMF, and HATU (3 eq) and DIPEA (4.5 eq) were added. The reaction mixture was added to the resin and stirred at RT under nitrogen bubbling for 45 min. The mixture was drained, and the resin was washed with DMF (3×) and DCM (3×).

#### Method D: Acetylation of the N-terminal position

After the final Fmoc deprotection, the resin was swollen with DCM (2×). A solution of acetic anhydride (10 eq) and DIPEA (10 eq) in DMF was added to the resin and stirred at RT under nitrogen bubbling for 15 min before the mixture was drained. This procedure was repeated twice, and the resin was washed with DMF (3×) and DCM (3×).

#### Method E: Cleavage from resin/side-chain deprotection

A solution of TFA/water/1,2-ethanedithiol/triisopropylsilane 94:2.5:2.5:1 (5 ml) was added to the dry resin. The reaction mixture was shaken at RT for 3 h. TFA solution was then collected in cold diethyl ether. The resin was rinsed with TFA (1 ml, 3×) and the TFA solution collected. The precipitated peptide was centrifuged, and the crude peptide was lyophilized from acetonitrile–water.

### Full-length expression and purification of 14-3-3

The σ and ζ isoforms of human 14-3-3 were cloned in the pProEx Htb vector as 6His-Linker (MSYYHHHHHHDYDIPTTENLYFQGAMGS)-h14-3-3 and expressed in *E. coli* BL21 (DE3)STAR competent cells. Cultures were grown in Terrific Broth media supplemented by 3-mM MgCl_2_, 0.02% glucose, 0.8% glycerol, and 50 μg/ml carbenicillin at 37 °C to an optical density at 600 nm of 0.4 to 0.6 and then induced overnight with 0.4 mM of IPTG at 18 °C to reach an optical density at 600 nm higher than 20. Cells were harvested by centrifugation (5000*g*, 20 min) and lysed using a cell disruptor (Constant Systems Limited) at 25 kilopounds per square inch (KPSI) in the lysis buffer containing 50-mM Tris, 300-mM NaCl, 12.5-mM imidazole, 1-mM tris(2-carboxyethyl)phosphine (TCEP), 5-mM MgCl_2_, and 1 tablet protease inhibitor Roche per 100 ml of the lysis buffer. After centrifugation (35,000*g*, 45 min), the lysate was incubated with Ni-NTA derivatized Sepharose resin (Qiagen) overnight at 4 °C. The nickel-resin was washed with the buffer containing 0.1% Triton X-100 then with lysis buffer and the proteins were eluted with buffer containing 250-mM imidazole. 14-3-3 proteins were further purified by size-exclusion chromatography on a HiLoad 26/60 Superdex 75 per grade (Pharmacia Biotech) using HBS P+ buffer (10-mM Hepes pH 7.4, 150-mM NaCl, 0.005% v/v Tween-P20). The correct fractions were combined, concentrated, aliquoted, frozen in liquid nitrogen, and stored at −80 °C.

### Expression and purification of 14-3-3 ΔC

Human 14-3-3ζΔC (C-terminally truncated, including residues 1–231, for crystallography purposes) was cloned in the pProEx Htb vector and expressed in *E. coli* BL21 (DE3) competent cells. Cultures were grown in TB media at 37 °C to an optical density at 600 nm of 0.8 to 1 and then induced overnight with 0.4-mM of IPTG at 18 °C to reach an optical density at 600 nm higher than 20. Cells were harvested by centrifugation (10,000*g*, 15 min) and lysed using a cell disruptor (Avestin EmulsiFlex C3 Homogenizer) at 20 KPSI in the lysis buffer containing 50-mM Tris, 300-mM NaCl, 12.5-mM imidazole, and 2-mM β-mercaptoethanol. After centrifugation (40,000*g*, 30 min), the lysate was applied to a HisTrap HP column (GE) and washed with the lysis buffer, and the 14-3-3 proteins were eluted with 50-mM Tris, 300-mM NaCl, 250-mM imidazole, and 2-mM β-mercaptoethanol. The right fractions were combined, and the full-length proteins were dialyzed and rebuffered in 25-mM Hepes, pH 7.5, 100-mM NaCl, 10-mM MgCl_2_, 0.5-mM Tris(2-carboxyethyl)phosphine. The His Tag was removed *via* TEV cleavage. The cleavage solution was applied to the HisTrap HP column to remove the TEV protease. The protein was further purified *via* size-exclusion chromatography on a Superdex 75 (GE) using the following buffer: 20-mM Tris, pH 7.4, 150-mM NaCl, and 2-mM β-mercaptoethanol. The correct fractions were combined, concentrated, aliquoted, frozen in liquid nitrogen, and stored at −80 °C.

### GR LBD expression and purification

GR N514-K777 and GR F602S N514-K777 were cloned in the pET24a base vector as the N-terminal 6His tag with an adjacent TEV cleavage site (ENLYFQG) and expressed in *E. coli* BL21 (DE3)STAR competent cells. Cultures were grown in autoinduction TB media 5052: TB media supplemented by 3-mM MgCl_2_, 0.05% glucose, 0.5% glycerol, 0.2% lactose, 100 μg/ml kanamycin, and 100-μM dexamethasone at 37 °C to an optical density at 600 nm of 1 and then induced for 48 h at 16 °C. The culture was centrifuged (5000*g*, 20 min), and the pellet was lysed using a cell disruptor (Constant Systems Limited) at 25 KPSI in the lysis buffer containing 50-mM Tris, pH 8, 1% CHAPS, 10% glycerol, 1-mM TCEP, 50-μM dexamethasone, and 1 tablet protease inhibitor Roche per 100 ml of the lysis buffer. The lysate was incubated with Ni-NTA derivatized Sepharose resin (Qiagen) overnight at 4 °C washed with the buffer containing 60-mM NaCl and 30-mM imidazole and eluted with the buffer containing 30-mM NaCl, 300-mM imidazole. GR LBD proteins were further purified by size-exclusion chromatography on a HiLoad 16/60 Superdex 75 per grade (Pharmacia Biotech) using the following buffer: 50-mM Tris, pH 9, 1-mM TCEP, and 25-μM dexamethasone. The correct fractions were combined, concentrated, aliquoted, frozen in liquid nitrogen, and stored at −80 °C.

### FP assay with GR phosphorylated peptides and 14-3-3

The FITC-labeled peptides were solubilized to a final concentration of 60 nM (for the monophosphorylated peptides) or 10 nM (for the doubly phosphorylated peptides) and were titrated with 14-3-3ζ or 14-3-3σ in concentration dilution series from 300 μM or 150 μM, depending on the binding affinity of the peptide, in a 384-well flat-bottom black polypropylene microplate (Greiner Bio-One). After 30-min incubation at RT, the plates were read on a plate reader PHERAstar (BMG LABTECH GmbH) for FP signal using white light and standard excitation (485 nm) and emission (520 nm). Measurements were performed as triplicates. Binding data were fit to a standard Hill equation using the software Genedata Screener. The figures were made using the software GraphPad Prism 8. FP data were expressed as millipolarization (mP) units, and the dynamic range (ΔmP) was the difference of the signals from the bound peptide and free peptide. Errors are the standard error of the fit.

### SPR assays with GR phosphorylated peptides and 14-3-3

SPR binding assays were performed using a Biacore 3000 (GE Healthcare). 6His-tagged 14-3-3 proteins (3 μM) were immobilized on a SPR sensor chip NTA derivatized carboxymethyl dextran hydrogel (XanTec Bioanalytics) at 4000 RU for 14-3-3σ and 2000 RU for 14-3-3ζ according to the manufacturer’s instructions. A 96-well V-shaped polypropylene microtiter plate was prepared with different GR peptides concentrations in HBS P+ buffer and normalized to 1 or 3% v/v DMSO, depending on the maximum peptide concentration, using the HP D300 Digital Dispenser (Hewlett-Packard Company). In the dose–response assay, 10 dilutions of the GR peptide from 300 μM or 45 μM, depending on the peptide binding affinity, were performed. The peptide samples were injected using the HBSP + buffer with 1 or 3% v/v DMSO at a continuous flow rate of 20 μl/min for 1 min on the immobilized 14-3-3 and before the next injection 2.5 min after. An extra waiting time of 12.5 min was set to allow complete dissociation of the doubly phosphorylated peptides. Measurements were performed as triplicates. When necessary data were corrected using a solvent correction curve, binding data were fit to a standard Hill using the software Genedata Screener and the figures were made using the software GraphPad Prism 8. Errors are standard error of the fit.

### X-ray crystallography studies

#### Sitting-drop cocrystallization of 14-3-3ζ and GR peptides

Crystals were grown using the sitting-drop vapor diffusion crystallization method by mixing 14-3-3ζΔC with a monophosphorylated GR peptide in a 1:2 ratio or with a doubly phosphorylated peptide in a 1:1 ratio with a resulting protein concentration of 10 mg/ml in a crystallization buffer containing 20-mM Hepes, pH 7.5, 2-mM MgCl_2_, and 2-mM DTT. Sitting drops were formed by mixing equal volumes of protein/peptide solution and precipitant (2 × 100 nl) on a 96-well Art Robbins Flat Ledge plate (Hampton Research) using a nanoliter liquid handler mosquito (SPT Labtech) and equilibrated over an 80-μl reservoir at 4 or 20 °C. Crystals were harvested after a few days, soaked in a cryoprotectant containing the precipitant supplemented with 20% v/v glycerol, and flash-cooled in liquid nitrogen before data collection. The precipitant solutions are listed below.

#### GR_pT524 cocrystallized with 14-3-3ζ

The crystals were grown at 4 °C using a precipitant solution (1.29 M MgCl_2_, 22.5% PEG 3350, 0.1 M Tris, pH 8.3) supplemented with 10% of an additive containing 40% v/v of 2,5-hexanediol. The crystals were harvested after 8 days.

#### GR_pS617 cocrystallized with 14-3-3ζ

The crystals were grown at 4 °C using a precipitant solution (2.04 M ammonium sulfate, 0.2 M sodium citrate) supplemented with 10% of an additive containing 0.33% w/v 3-aminobenzoic acid, 0.33% w/v 3-aminosalicylic acid, 0.33% w/v salicylic acid, and 0.02 M Hepes sodium, pH 6.8. The crystals were harvested after 5 days.

#### GR_pT524-pS617 cocrystallized with 14-3-3ζ

The crystals were grown at 20 °C using a precipitant solution (0.35 M MgCl_2_, 24.4% PEG 3350, 0.1 M Bis Tris pH 5.5) supplemented with 10% of an additive containing 0.06 M CHAPS, 0.06 M Hepes, 0.06 M Tris, 0.25% w/v hexamminecobalt(III) chloride, and 0.02 M Hepes sodium, pH 6.8. The crystals were harvested after 8 days.

#### Data collection and processing

Diffraction data were collected at the European Synchrotron Radiation Facility in Grenoble, France (0.976 Å, 100 K, European Synchrotron Radiation Facility Beamline ID30B) and at Diamond Light Source at the Harwell Science and Innovation Campus in Oxfordshire, UK (0.916 Å, 100 K, Diamond Beamline I04-1). Molecular replacement was performed using Phaser from the ccp4i package and refinement, and manual rebuilding was performed using Buster and Coot software packages. The structures (PDB codes: 6YO8, 6YMO, and 6YOS) were refined to a resolution of 2.09 Å, 2.01 Å, and 2.75 Å with Rwork/Rfree factors of 0.224/0.235, 0.212/0.228, and 0.268/0.291, respectively. X-ray diffraction data collection and structure refinement statistics are summarized in Table 2. The figures were made using the software PyMol (DeLano Scientific LLC).

### Radiometric protein kinase filter-binding assay

The kinase screen was performed by ProQinase GmbH, Freiburg im Breisgau, Germany. A radiometric protein kinase filter-binding assay was used for measuring the kinase activity of the 245 serine/threonine kinases. The reaction cocktails were pipetted into 96-well V-shaped polypropylene microtiter plates in the following order: kinase solution (10 μl) and buffer/ATP/test sample mixture (40 μl). The reaction cocktails contained 60-mM Hepes-NaOH, pH 7.5, 3-mM MgCl_2_, 3-mM MnCl_2_, 3-μM Na-orthovanadate, 1.2-mM DTT, 1-μM ATP/[γ-33P]-ATP (8.68 × 1005 cpm per well), protein kinase (1–400 ng/50 μl), and sample protein (5 μg/50 μl) with minor modifications as stated in the Supporting information. Each assay plate comprised one well for a buffer/substrate control containing no enzyme. The assay plates were incubated at 30 °C for 60 min, and the reaction cocktails were stopped with 20 μl of 10% v/v H_3_PO_4_. The reaction cocktails were transferred into 96-well glass-fiber filter plates (MultiScreen MSFC, Millipore) and prewetted with 150 mM H_3_PO_4_, followed by 10 min incubation at RT. After washing with 250 μl of 150-mM H_3_PO_4_ (3×) and with 20 μl of 100% ethanol and drying for 30 min at 40 °C, 50 μl of scintillator (ROTISZINT eco plus, Roth) was added to each well and incorporation of ^33^Pi (“counting of cpm”) was determined with a microplate scintillation counter (MicroBeta, PerkinElmer).

### Peptide mapping

The protein samples were phosphorylated as described above with nonradioactive ATP. About 10, 5, 3, and 1 μg of protein sample, supplemented with 25% NuPAGE LDS sample buffer (4×) and 10% NuPAGE sample reducing agent (10×), was run on a NuPAGE 4 to 12% Bis-Tris Protein Gels and 1.0 mm with Mops buffer and the bands were revealed using Coomassie Brilliant Blue. Bands of interest were cut from the gel to be washed with water, 1:1 acetonitrile/water, 100-mM ammonium bicarbonate, and 1:1 acetonitrile/100-mM ammonium bicarbonate successively for 10 min. The bands were reduced and alkylated with a solution of 10-mM DTT in 100-mM ammonium bicarbonate for 45 min at 65 °C followed by a solution of 50-mM iodoacetamide in ammonium bicarbonate for 20 min in the dark. The samples were washed with 50-mM ammonium bicarbonate, 1:1100-mM ammonium bicarbonate/acetonitrile, and 100% acetonitrile successively for 10 min and dried. The bands were digested with 10 ng/μl trypsin and 12.5 ng/μl chymotrypsin in 50-mM ammonium bicarbonate at RT overnight. The material was extracted twice with 0.1% TFA/60% acetonitrile and then dried before being analyzed by MS. The data files generated were searched against the in-house or SwissProt database using the software Mascot Daemon. The searches were then manually verified.

### Intact mass

The protein samples were phosphorylated as described above with nonradioactive ATP. The samples were diluted to 0.01 mg/ml in 0.1% formic acid and 5% acetonitrile and were analyzed by MS. The TIC peak data were used to generate the m/z spectrum, and the data were deconvoluted to give final mass.

### Database search parameters and acceptance criteria for identifications

Search engine and release version: Mascot Daemon, version 2.7.0

Sequence database searched: SwissProt, Human

Release version/date of sequence database searched: August 2018

The number of entries in the database actually searched: Whole of human SwissProt database

Specificity of all proteases used to generate peptides: Trypsin and chymotrypsin. Trypsin specificity is the C-terminal side of lysine and arginine unless there is a proline on the carboxyl side. Chymotrypsin’s main cleavage sites are after tryptophan, tyrosine, and phenylalanine.

The number of missed and/or nonspecific cleavages permitted: 2

Fixed modifications (including residue specificity) considered: Carbamidomethylation on cysteine (+57.021)

Variable modifications (including residue specificity) considered: Oxidized methionine (+15.995 Da) and phosphorylation of threonine, serine, tyrosine (+15.966 Da)

Mass tolerance for precursor ions: ±0.6 Da

Mass tolerance for fragment ions: ±0.6 Da

Threshold score/expectation value for accepting individual spectra: 20

### Cell culture, transfection, and stimulation

The HEK293 and U2OS cell lines (American Type Culture Collection) were cultured in Dulbecco's modified Eagle's medium, supplemented with 10% fetal bovine serum and 1% penicillin-streptomycin (10,000 U/ml) at 37 °C in a 5% CO_2_ environment. For the Co-IP assay, 3 × 106 cells were seeded in a 15-cm cell culture dish. At 60 to 70% confluence, the HEK293 cells were transiently transfected with a mixture of 6-μg GFP-MINK1 or GFP-GR DNA and 24-μl lipofectamine 2000 in 2-ml Opti-MEM for 24 h. At 60 to 70% confluence, the U2OS cells were transiently transfected with a mixture of 8-μg GFP-MINK1 or GFP-GR and/or FLAG-MINK1 DNA and 32-μl X-tremeGENE 9 DNA in 2 ml Opti-MEM for 24 h. Stimulation with forskolin, H89, IGF-1, and PI303 was preceded by a cell starvation step where the cells were incubated with DMEM without supplements overnight. The cells were treated with the right compound at the concentration and during the time stated in the table found in the Supporting information.

### Co-IP assay

Cells were collected and lysed in radioimmunoprecipitation assay buffer containing 50-mM Tris-HCl, pH 8, 150-mM NaCl, 1-mM EDTA, 0.1% SDS, 1% Na deoxycholate, and 1% NP-40 supplemented with a protease inhibitor cocktail. After lysis, the concentration of protein was quantified by the Bradford test. The dilution/washing buffer containing 50-mM Tris HCl, pH 8, 150-mM NaCl, and 1-mM EDTA was added to reach equal concentration, and 50 μl was saved as the input fraction for future analysis. Equal volumes of the protein solution were incubated with 10-μl GFP-Trap Agarose beads (ChromoTek) at 4 °C for 2 h. 50 μl was saved as a flow-through fraction for future analysis, and the beads were washed with dilution/washing buffer (2×). 30 μl NuPAGE LDS sample buffer (4×) was added to the beads, and proteins from the input, flow-through, and IP fractions were denatured at 95 °C for 5 min.

Eluted proteins were resolved on a NuPAGE 4 to 12% Bis-Tris Protein Gels, 1.0 mm. The gels were transferred to polyvinylidene difluoride membranes (previously activated in methanol) that were then blocked with Intercept (TBS) blocking buffer (LI-COR). After washing with tris-buffered saline with Polysorbate 20 (TBST), the membranes were probed at 4 °C overnight with the primary antibodies listed in Supporting information. After washing with TBST, the membranes were incubated with the specific rabbit IRDye 800CW secondary antibody (LI-COR) for 1 h at RT. After washing with TBST, the blots were detected using an Odyssey CLx Imaging system (LI-COR). Data analysis was performed using Image Studio (LI-COR).

### Far-Western blot assay

Samples containing proteins from the Co-IP assay or GR LBD *in vitro* phosphorylated were loaded on a NuPAGE 4 to 12% Bis-Tris Protein Gels, 1.0 mm, and separated. The gels were transferred to polyvinylidene difluoride membranes that were blocked with the Intercept (TBS) blocking buffer (LI-COR). After washing with TBST, the membranes were probed at 4 °C overnight with BMH1-BMH2-digoxigenin probe diluted in 5% bovine serum albumin TBST. After washing with TBST, the membranes were incubated with anti-digoxigenin antibody (R&D Systems) at 4 °C overnight. After washing with TBST, the membranes were incubated with the specific mouse IRDye 800CW or IRDye 680LT secondary antibody (LI-COR) for 1 h at RT. After washing with TBST, the blots were detected using an Odyssey CLx Imaging system (LI-COR). Data analysis was performed using Image Studio (LI-COR). Measurements were performed as triplicates. The figures were made using the software GraphPad Prism 8.

### Experimental design and statistical rationale

Individual data points are shown when possible and always for n ≤ 20. Clearly defined error bars are present, representing the SD of three independent experiments.

For the investigation of the interaction between full-length GR and 14-3-3, Western blot quantification was obtained using Image Studio (LI-COR) from three independent cell-based assays. The significance (two-tailed *p* value) was assessed by *t* test. Asterisks were attributed for the following significance values: ∗*p* < 0.05 and ∗∗∗∗*p* < 0.0001.

## Data availability

The authors declare that all data supporting the findings of this study are available within the article and its Supporting information files. The MS proteomics data have been deposited to the ProteomeXchange Consortium *via* the PRIDE (44) partner repository with the dataset identifiers PXD022364 and 10.6019/PXD022364.

## Supporting information

This article contains supporting information.

## Conflict of interest

L. D. M., K. E., A. G., A. S., L. W., and M. W. D. P. are employed by and/or own shares in AstraZeneca.

## Supporting information

Supplemental Figures S1–S9, Tables S1–S2 and Equation S1
