# Supplementary material for: Glucocorticoid receptor Thr524 phosphorylation by MINK1 induces interactions with 14-3-3 protein regulators
Source: J Biol Chem. 2021 Mar 17;296:100551. doi: 10.1016/j.jbc.2021.100551 (PMC8080530; doi:10.1016/j.jbc.2021.100551)
Supplement: Supplemental Figures S1–S9, Tables S1–S2 and Equation S1 [file mmc1.pdf]

Supporting Information

Glucocorticoid receptor Thr524 phosphorylation by MINK1 induces interactions with 14-3-3 protein regulators

Claire C. Munier[1,2], Leonardo De Maria[1], Karl Edman[3], Anders Gunnarsson[3], Marianna Longo[4], Carol MacKintosh[4], Saleha Patel[5], Arjan Snijder[3], Lisa Wissler[3], Luc Brunsveld[2], Christian Ottmann[2], Matthew W. D. Perry[1,*]

[1] Research and Early Development, Respiratory & Immunology, BioPharmaceuticals R&D, AstraZeneca, Gothenburg, Sweden.

[2] Laboratory of Chemical Biology, Department of Biomedical Engineering and Institute for Complex Molecular Systems, Technische Universiteit Eindhoven, Den Dolech 2, 5612 AZ Eindhoven, The Netherlands.

[3] Discovery Sciences, BioPharmaceuticals R&D, AstraZeneca, Gothenburg, Sweden.

[4] Division of Cell and Developmental Biology (C.M.), College of Life Sciences, University of Dundee, Dundee DD1 5EH, Scotland, UK.

[5] Discovery Biology, Discovery Sciences, R&D, AstraZeneca, Cambridge, UK

Table of content

General information

Supporting details

Fig. S1. Interaction of GR peptides with 14-3-3σ.

Fig. S2. GR peptides co-crystallized with 14-3-3ζ.

Fig. S3. List of the 245 serine/threonine kinases tested in the initial kinase screening.

Fig. S4. Activity of 245 serine/threonine kinases with GR LBD.

Fig. S5. Results from the hit confirmation assay of the 12 selected kinases with GR LBD.

Fig. S6. MS spectra and peptide mapping of GR LBD phosphorylated by 5 kinases.

Fig. S7. Initial Co-IP assay revealing the same stimuli trigger the GR–14-3-3 interaction as those that lead to phosphorylation of MINK1.

Fig. S8. MINK1–14-3-3 protein–protein interaction upon forskolin and calyculin A cell stimulation.

Fig. S9. Very low endogenous expression of MINK1 in U2OS cells.

Table S1. Binding affinity of the GR peptides from the alanine scan and mutations with 14-3-3ζ measured by FP.

Table S2. Activity values by the selected kinases for hit confirmation.

Equation S1. Mass balance equation for the interaction ditopic host−guest systems.

GR_pT524-pS617 orientation in the crystal structure with 14-3-3ζ

GR peptide analysis

1. General information

1.1. Material, instruments and reagents

1.1.1. Peptide synthesis

The peptides were synthesized via Fmoc/tBu solid-phase peptide synthesis using a Biotage® Initiator+ AlstraTM automated microwave peptide synthesizer or a Biotage® Syro II automated parallel peptide synthesizer. Peptide syntheses were monitored by reversed-phase (RP) UPLC-MS or were bought from commercial custom peptide suppliers. Analytical RP-UPLC-MS was performed on a Waters Acquity UPLC system (PDA, sample manager, sample organizer, column oven modules) and Waters SQD2 mass spectrometer using the following column: Waters Acquity CSH C18 column, 130Å, 1.7 µm, 50 × 2.1 mm at a flow rate of 0.5 mL/min at 45 °C. A linear gradient of mobile phase: A=H_2O + 10 mM formic acid, 1 mM ammonia and 0.03% TFA and B=acetonitrile/H_2O 95/5 v/v + 10 mM formic acid, 1 mM ammonia and 0.03% TFA was used with detection at 220 nm.

The peptides were purified by preparative RP HPLC-MS performed on Waters FractionLynx HPLC MS systems equipped with Waters 2545 pump module, Waters 2767 injector/fraction collector, and either Waters 2998 PDA detector and Waters 3100 mass spectrometer or Waters 2489 UV detector and Waters ZQ mass spectrometer, using the following columns: Waters Atlantis T3 OBD column, 100Å, 5 µm, 150 × 19 mm; Waters XSelect CSH C18 OBD column, 130Å, 5 µm, 150 × 19 mm or Waters XSelect CSH Fluoro-Phenyl OBD column, 130Å, 5 µm, 150 × 19 mm. The mobile phases were: A=H_2O + 0.15% TFA and B=acetonitrile, with a flow of 30 mL/min at room temperature. The fraction collection was triggered on mass. Gradient systems were adjusted according to the elution profiles and peak profiles obtained from the analytical RP-UPLC chromatograms.

Water was purified using a Millipore MilliQ water purification system. All solvents and reagents were purchased from commercial suppliers and used without further purification unless indicated otherwise. RP-HPLC solvents were purchased as LC/MS grade. Peptides were synthesized using standard Fmoc SPPS except for Fluoro(6FAM)_GR_pS113, Fluoro(6FAM)_GR_pS134-pT524, Fluoro(6FAM)_GR_pS83-pT524, Fluoro(6FAM)_GR_pS83-pS134, Fluoro(6FAM)_GR_pT524-pT562 and acetylated_GR_pT524-pT562 which were purchased from ThermoFisher Scientific. Fmoc-amino acids were purchased from Chem-Impex International, Inc., with the following side-chain protection: Fmoc-Arg(Pbf)-OH, Fmoc-Asn(Trt)-OH, Fmoc-Asp(OtBu)-OH, Fmoc-Cys(Trt)-OH, Fmoc-Gln(Trt)-OH, Fmoc-Glu(OtBu)-OH, Fmoc-His(Trt)-OH, Fmoc-Lys(Boc)-OH, Fmoc-Ser(tBu)-OH, Fmoc-Thr(tBu)-OH, Fmoc-Trp(Boc)-OH, Fmoc-Tyr(tBu)-OH, Fmoc-Ser(PO(Obzl)OH)-OH, Fmoc-Thr(PO(Obzl)OH)-OH. Only L-Amino acids were used. 2-Chlorotrityl chloride resin (100-200 mesh), 1% DVB was purchased from Sigma-Aldrich. HATU was purchased from Chem-Impex International, Inc., DIPEA and FITC from Sigma-Aldrich. Acetic anhydride was purchased from Acros Organics.

1.1.2. Peptide mapping

Material obtained from the trypsin and the chymotrypsin digestion was analysed onto ESI MSMS using X500B mass spectrometer (Sciex) using the Exion chromatography system (Sciex): 20 minute reversed phase gradient with 2.1 × 100 mm Waters Acquity UPLC CSH C18 analytical column and the X500B for analysis at a flow rate of 300 µL/min. Data were collected in positive ion mode and an autoswitching setup was initiated with automatic precursor selection based on peak intensity and charge state. The collision energies were automatically adjusted based on the precursor and nitrogen was used as the collision gas. Electrospray parameters were as follows: curtain gas, 30 psi; ion source gas 1, 30 psi; ion source gas 2, 40

psi; and temperature, 400 °C. TOF MS mass range was set for 300-1800 Da and TOF MSMS mass range was 100-1500 Da.

1.1.3. Intact mass

The protein samples were analysed on Exion chromatography system(Sciex) with 3.5 µm, 2.1x50 mm, 300Å Waters XBridge Protein BEH C4 as column and a reversed phase gradient for 5 min. Acquisition was performed on X500B QTOF flow rate of 500 µL/min with a Turbo V™ ion source using protein mode acquisition and detector voltage selected over a range of 500–3000 Da. Electrospray parameters were as follows: curtain gas, 50 psi; ion source gas 1, 50 psi; ion source gas 2, 50 psi; and temperature, 400 °C. The X500B collected data in positive ion mode over a mass range of 500-3000 Da. Standard mobile phases were used (Mobile Phase A: 0.1% formic acid in water, Mobile Phase B: 0.1% formic acid in acetonitrile).

1.1.4. Radiometric protein kinase filter-binding assay

All protein kinases provided by ProQinase were expressed in Sf9 insect cells or in E.coli as recombinant GST-fusion proteins or His-tagged proteins. All kinases were produced from human cDNAs. Kinases were purified by either GSH-affinity chromatography or immobilized-metal affinity chromatography. Affinity tags were removed from a number of kinases during purification. The purity of the protein kinases was examined by SDS-PAGE/Coomassie staining, the identity was checked by mass spectroscopy. Kinases from external vendors (Carna Biosciences Inc.; Life Technologies (Invitrogen Corporation); Merck-Millipore (Millipore Corporation)) were expressed, purified and quality-controlled by virtue of the vendors readings.

1.1.5. Cell-based assays

15 cm Nunc™ cell culture dishes, DMEM Gibco®, trypsin-EDTA (0.05%) phenol red Gibco®, DPBS without Calcium and Magnesium Gibco®, Opti-MEM Gibco®, penicillin-streptomycin (10,000 U/mL) Gibco® and Fetal Bovine Serum (origin New Zealand) Gibco® were purchased from ThermoFisher Scientific. The transfection reagent Lipofectamine™ 2000 was purchased from ThermoFisher Scientific and X-tremeGENE™ 9 DNA, Roche from Sigma-Aldrich. The compounds forskolin, H89 and calyculin A were purchased from Sigma-Aldrich, IGF-1 was purchased from from Cell Signaling Technology whilst the pyridinylfuranopyrimidine inhibitor PI103 was a kind gift of Professor Carol Mackintosh (Division of Cell and Developmental Biology, School of Life Sciences, University of Dundee, Scotland, United Kingdom). Reagents required for western-blotting namely NuPAGE™ 4-12% Bis-Tris Protein Gels, 1.0 mm, NuPAGE™ LDS Sample Buffer (4X), NuPAGE™ Sample Reducing Agent (10X) and Coomassie Brilliant Blue were purchased from ThermoFisher Scientific. The primary antibodies used in the Co-IP experiments were: D8H2 (Cell Signaling Technology), GFP (Sigma-Aldrich), Anti-MAP4K6 antibody – C-terminal (abcam), pan 14-3-3(Cell Signaling Technology), Phospho-VASP Ser239 (Cell Signaling Technology) and Phospho-Akt (Ser473) (Cell Signaling Technology). The BMH1-BMH2-digoxygenin probe was a kind gift of Professor Carol Mackintosh (Division of Cell and Developmental Biology, School of Life Sciences, University of Dundee, Scotland, United Kingdom).

1.2. Plasmids

1.2.1. 14-3-3 plasmids

The vector plasmids pPROEX-HTb – h14-3-3ζ (including the gene coding sequence for h14-3-3ζ, cloned within the BamHI – NotI restriction sites, and a TEV-cleavable His6-tag), pPROEX-HTb – h14-3-3ζΔC (including the gene coding sequence for h14-3-3ζΔC, cloned within the BamHI – NotI restriction sites, and

a TEV-cleavable His6-tag) and pPROEX-HTb – h14-3-3σ (including the gene coding sequence for h14-3-3σ, cloned within the BamHI – NotI restriction sites, and a TEV-cleavable His6-tag) were a kind gift of Professor Christian Ottmann (Department of Biomedical Engineering, Eindhoven University of Technology, Netherlands).

14-3-3ζ sequencing

Client : Eindhoven University of Technology

Project Code : 123135

Sequence date : 04-02-2020

Filename : P200351164

Dna Code, name : 1741202, zet_FL

Primer Code, name : 352482, QS

Result : OK - Herhalen

Phred score : Q15/Q20=840/743

>P200351164_H17_073.ab1

ATYYSCCRTCYCCCTTCMYMGATTACKATATCCCAACGACCGAAAACCTGTATTTTCAGGGC
GCCATGGGATCCATGGATAAAAATGAGCTGGTTCAGAAGGCCAAACTGGCCGAGCAGGCTG
AGCGATATGATGACATGGCAGCCTGCATGAAGTCTGTAACTGAGCAAGGAGCTGAATTATC
CAATGAGGAGAGGAATCTTCTCTCAGTTGCTTATAAAAATGTTGTAGGAGCCCGTAGGTCAT
CTTGGAGGGTCGTCTCAAGTATTGAACAAAAGACGGAAGGKGCTGAKAAAAAACAGCAGAT
GGCTCGAGAATACAKAGAGAAAATTGAGACGGAGCTAAGAGATATCTGCAATGATGTACTG
TCTCTTTTGGAAAAGTTCTTGATCCCCAATGCTTCACAAGCAGASASCAMAKTCTTCTATTTG
AAAATGAAAGGAGATTACTACCGTTACTTGGCTGAGGTTGCCGCTGGTGATGACAAGAAAG
GGATTGTCGATCAKTCACAACAAGCATACCAAGAAGCTTTTGAAATCASCAAAAAGGAAAT
GCAACCAACACATCCTATCAKACTGGGTCTGGCCCTTAACTTCTCTGTGTTCTATTATGAGAT
TCTGAACTCCCCAGARAAAGCCTGCTCTCTTGCAAASACAGCTTTTGATGAAGCCATTGCTG
AACTTGATACATTAAGTGAASAGTCATACAAAGACAGCACGCTAATAATGCAATTACTGAKA
GACAACTTGACATTGTGGACATCGGATACCCAAGGAGACGAAGCTGAAGCATGAKAAGGAT
GGGAAAATTAACTCGACGAGCTCACTAGTCGCGGCCGCTTTCGAATCTAGAGCCTGCAGTCT
CGAGGCATGCGGTACCAWGCTTGGCTGTTTTGCCGGATGASAKAAGATTTTCASCTGAYACM
SATTAATCAAACGCWSATSCGGTCTKATWAAAYASATTTGCCTGGCGGCAGTAKCGCGGTGG
TYCYAYCTGACYCYCATGCKAWCTCAGACATGAAYGCCGWACTGCCGATCGTTAKTGTKGR
KTCTYCCCATGCTAGAWTAGMAACTGCCMRGCATCAWCTAAACKAACGKTTC

14-3-3ζΔC sequencing

Client : Eindhoven University of Technology

Project Code : 123135

Sequence date : 04-02-2020

Filename : P200351165

Dna Code, name : 1741203, zet_dC

Primer Code, name : 352482, QS

Result : OK - Herhalen

Phred score : Q15/Q20=925/832

>P200351165_J17_071.ab1

TGTTSGACTCCTTCMCYATCMCCATCACGATTACGATATCCCAACGACCGAAAACCTGTATT
TTCAGGGCGCCATGGGATCCATGGATAAAAATGAGCTGGTTCAGAAGGCCAAACTGGCCKA
GCAGGCTGAGCGATATGATGACWTGGCAKCCTGCATGAAGTCTGTAWCTGAGCAAGGAGCT
GAATTATCCAATGAGGAGAGGAATCTTCTCTCAGTTGCTTATAAAAATGTTGTAGGAGCCCG
TAGGTCATCTTGGAGGGTCGTCTCAAGTATTGAACAAAAGACGGAAGGTGCTGAGAAAAAA
CAGCAGATGGCTCGAGAATACWGAGAGAAAATTGAGACGGAGCTAAGAGATATCTGCAAT
GATGTACTGTCTCTTTTGGAAAAGTTCTTGATCCCCAATGCTTCACAAGCAGAGAGCAAAGT
CTTCTATTTGAAAATGAAAGGAGATTACTACCGTTACTTGGCTGAGGTTGCCGCTGGTGATG
ACAAGAAAGGGATTGTCGATCAGTCACAACAAGCATACCAAGAAGCTTTTGAAATCAGCAA
AAAGGAAATGCAACCAACACATCCTATCAGACTGGGTCTGGCCCTTAACTTCTCTGTGTTCT
ATTATGAGATTCTGAACTCCCCASAGAAAGCCTGCTCTCTTGCAAAGACAGCTTTTGATGAA
GCCATTGCTGAACTTGATACATTAAGTGAAGAGTCATACAAAGACAGCACGCTAATAATGC
AATTACTGAGAGACAACTTGACATTGTGGACATCGTGATACCCAMKGAGACGAYATTGTGG
ACATCGTGATACCCAAGGACATTGTGGACATCGTGATACCCAAGGAKACGAAGCTGAAGCA
GGAGAASGAGGGGAAAATTAAGTCGACGAGCTCACTAGTCGCGKCCGCTTTYCGAATCTAG
AGCCTGCAKTCTCGAGGYATGCGGTACCWAGCTTGTCTGTTTTGGCGGATGAGAGAAGATTT
TCAKCCTGATACAGATTAWTCATAACGCACAARCGGTCTGATAAACAGAATTTGCCTGTCGG
YAKTAGCGCKGTGGTCCCACCTGACYCCATGCCGACYCAGAGTGAACSCCGTASCG

14-3-3σ protein analysis

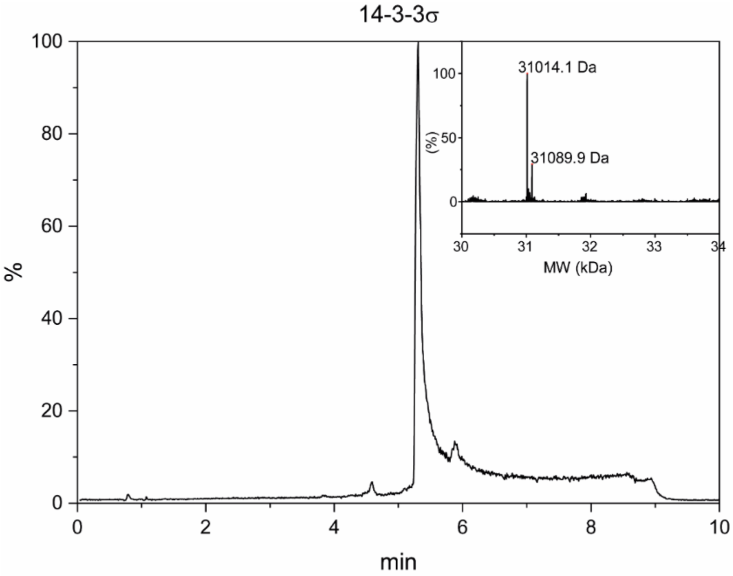

1.2.2. GR LBD plasmid

The vector plasmid pET24a-GR_F602S_LBD_N514_K777 (including the gene coding sequence for GR_F602S_LBD_N514_K777, cloned within the Nde1 – ECOR1 restriction sites, and a TEV-cleavable His6-tag) came from AstraZeneca's plasmid collection.

(**A**), Purity of GR_F602S_LBD_N514_K777 proteins: 95% according to SDS-PAGE. (**B**), Western blot of the GR_F602S_LBD_N514_K777 proteins at two concentrations. Monomeric, dimeric and oligomeric forms were observed. (**C**) Mass calculated for GR_F602S_LBD_N514_K777, 32.43 kDa; found 32.43 kDa.

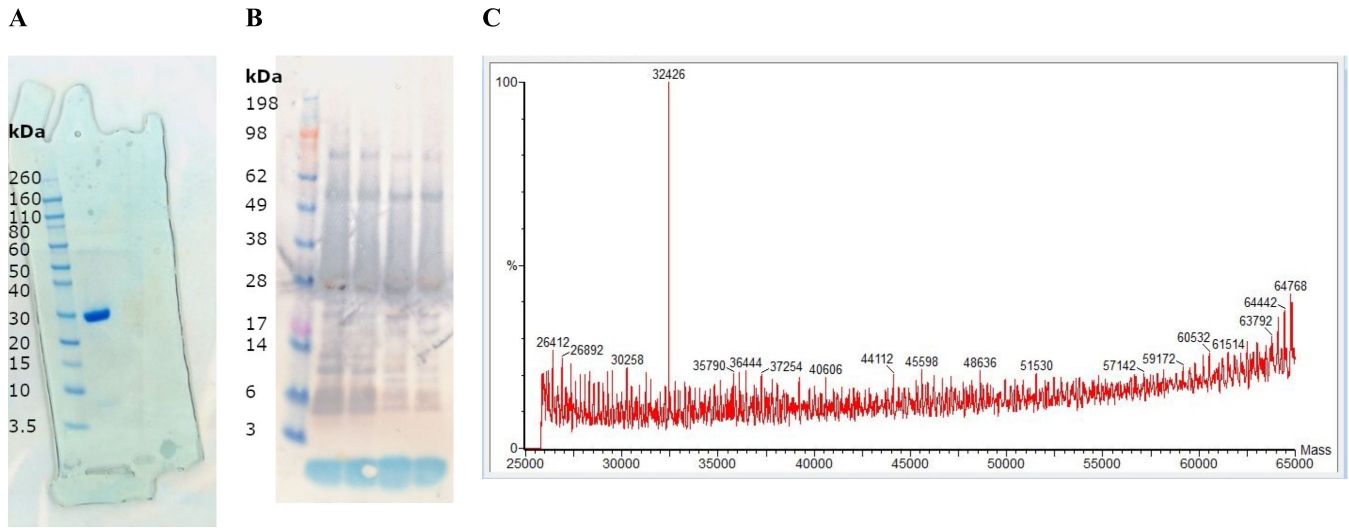

1.2.3. Full length GR plamids

The vector plasmids pcDNA3.1+N-eGFP-GR and pcDNA3.1+N-eGFP-GR_T524A_S617A (including the gene coding sequence for GR or the GR mutant GR_T524A_S617A cloned within the Nhe1 – Not1 restriction sites) were purchased from GenScript.

Certificate of Analysis

Project ID: U9661DG170-8

Construct Information:

Gene Name: Seq4 GR T524A S617A

Clone ID: V67840 **Gene Length:** 2351 bp

Cloning Vector: pCDNA3.1+N-eGFP **Cloning Strategy:** Mutation

QC Items	Specifications	Results	
**Sequencing Alignment**	Sequencing results are consistent with the targeted insert sequence.	Pass	Consistent
**Vector Sequence**	The flanking sequences of the cloning site are correct.	Pass	Correct<br>Shown in the SQD file
**Restriction Digests**	The size of inserted fragment is correct and free of unexpected bands suggesting contamination.	Pass	Correct<br>Shown in attachment 1
**DNA Quality**	Miniprep: 4 μg<br>OD260/280=1.8~2.0<br>Free of contamination	Pass	⩾ 4 μg<br>OD260/280=1.80<br>Pure
**Quality grade**	Research Grade	Pass	Research Grade
**Appearance**	Clear and free of foreign particles.	Pass	Clear<br>Free of foreign particles
**Additional Test**		N/A	

NOTE

Shipping at	Plasmid Storing at	Bacstab Storing at	Glycerol Stock Storing at
Room Temperature	-20℃	4℃	-20℃/-80℃

Morgan

Certified by: **Date: 08/05/2018**

Thank you for your patronage to our Gene services! To maintain this working relationship, we shall be grateful if you can add our webpage URL into your lab website. As a token of appreciation, you will be rewarded by 1,000 EZcoupon™ points. For more information, please contact us by e-mail at web@genscript.com.

GenScript — Make Research Easy
CONFIDENTIAL

Certificate of Analysis

Project ID: U9661DG170-2

Construct Information:

Gene Name: Seq1 GR wt_pCDNA3.1+N-eGFP

Clone ID: V65434 **Gene Length:** 2351 bp

Cloning Vector: pCDNA3.1+N-eGFP **Cloning Strategy:** EcoRI / NotI

QC Items	Specifications	Results	
**Sequencing Alignment**	Sequencing results are consistent with the targeted insert sequence.	Pass	Consistent
**Vector Sequence**	The flanking sequences of the cloning site are correct.	Pass	Correct<br>Shown in the SQD file
**Restriction Digests**	The size of inserted fragment is correct and free of unexpected bands suggesting contamination.	Pass	Correct<br>Shown in attachment 1
**DNA Quality**	Miniprep: 4 μg<br>OD260/280=1.8~2.0<br>Free of contamination	Pass	⩾ 4 μg<br>OD260/280=1.85<br>Pure
**Quality grade**	Research Grade	Pass	Research Grade
**Appearance**	Clear and free of foreign particles.	Pass	Clear<br>Free of foreign particles
**Additional Test**		N/A	

NOTE

Shipping at	Plasmid Storing at	Bacstab Storing at	Glycerol Stock Storing at
Room Temperature	-20℃	4℃	-20℃/-80℃

Morgan

Certified by: **Date: 08/04/2018**

Thank you for your patronage to our Gene services! To maintain this working relationship, we shall be grateful if you can add our webpage URL into your lab website. As a token of appreciation, you will be rewarded by 1,000 EZcoupon™ points. For more information, please contact us by e-mail at web@genscript.com.

For research use only

860 Centennial Ave., Piscataway, NJ 08854, USA

Toll-Free: 1-877-436-7274 Tel: 1-732-885-9188 Fax: 1-732-210-0262 Email: order@genscript.com Web: www.genscript.com

Page 1 of 2

1.2.4. FLAG-MINK1

The vector plasmid pcDNA3.1-MINK1 (including the gene coding sequence for MINK1, cloned within the Nde1 – XhoI restriction sites, and a N-terminal FLAG-tag) was purchased from GenScript.

GenScript

Make Research Easy
CONFIDENTIAL

Certificate of Analysis

Project ID: U322BEH140-2

Construct Information:

Gene Name: FLAG-MINK1_pcDNA3.1(+)

Clone ID: A43416 **Gene Length:** 4044 bp

Cloning Vector: pcDNA3.1(+) **Cloning Strategy:** NheI/XhoI

Growth in Bacteria:

Plasmid resistance: Ampicillin

Suggested competent cell: JM108

Growth Temperature: 37 °C

Sequencing primer(s) *:

PEGFP-N-5: TGGGAGGTCTATATAAGCAGAG

BGH: TAGAAGGCACAGTCGAGG

*: The primers are located at the 5'and/or 3'sides of MCS region.

Default	Shipping at	Plasmid Storing at	Bacstab Storing at	Glycerol Stock Storing at
Lyophilized	Room Temperature	-20°C	4°C	-80°C

QC Items	Specifications	Results	
**Sequencing Alignment**	Sequencing results are consistent with the targeted insert sequence.	Pass	Consistent
**Vector Sequence**	The flanking sequences of the cloning site are correct.	Pass	Correct
			Shown in the SQD file
**Restriction Digests**	The size of inserted fragment is correct and free of unexpected bands.	Pass	Correct
			Shown in attachment 1
**DNA Quality**	Miniprep: 4 µg	Pass	≥ 4 µg
	OD260/280=1.8~2.0		OD260/280=1.89
	Free of contamination		Pure
**Quality Grade**	Research Grade	Pass	Research Grade
**Appearance**	Clear and free of foreign particles.	Pass	Clear
			Free of foreign particles
**Additional Test**	N/A	N/A	

Certified by: Morgan **Date:** 09/01/2019

For research use only

860 Centennial Ave., Piscataway, NJ 08854, USA

Toll-Free: 1-877-436-7274 Tel: 1-732-885-9188 Fax: 1-732-210-0262 Email: order@genscript.com Web: www.genscript.com

Page 1 of 2

1.2.5. MINK1-GFP

The vector plasmid pcDNA5-FRT/TO-MINK1 (including the gene coding sequence for MINK1 cloned within the BamH1– Not1 restriction sites and a C-terminal GFP-tag) was a kind gift of Professor Carol Mackintosh (Division of Cell and Developmental Biology, School of Life Sciences, University of Dundee, Scotland, United Kingdom). Information and sequencing confirmation of MINK1-GFP plasmid:

TCGCCACCTACTACGGAGCCTTCATCAAGAAGAGCCCCCCGGGAAACGATGACCAGCTCTGGCTG
GTGATGGAGTTCTGTGGTGCTGGTTCAGTGACTGACCTGGTAAAGAACACAAAAGGCAACGCCCT
GAAGGAGGACTGTATCGCCTATATCTGCAGGGAGATCCTCAGGGGTCTGGCCCATCTCCATGCCC
ACAAGGTGATCCATCGAGACATCAAGGGGCAGAATGTGCTGCTGACAGAGAATGCTGAGGTCAAG
CTAGTGGATTTTGGGGTGAGTGCTCAGCTGGACCGCACCGTGGGCAGACGGAACACTTTCATTGG
GACTCCCTACTGGATGGCTCCAGAGGTCATCGCCTGTGATGAGAACCCTGATGCCACCTATGATT
ACAGGAGTGATATTTGGTCTCTAGGAATCACAGCCATCGAGATGGCAGAGGGAGCCCCCCCTCTG
TGTGACATGCACCCCATGCGAGCCCTCTTCCTCATTCCTCGGAACCCTCCGCCCAGGCTCAAGTC
CAAGAAGTGGTCTAAGAAGTTCATTGACTTCATTGACACATGTCTCATCAAGACTTACCTGAGCCG
CCCACCCACGGAGCAGCTACTGAAGTTTCCCTTCATCCGGGACCAGCCCACGGAGCGGCAGGTC
CGCATCCAGCTTAAGGACCACATTGACCGATCCCGGAAGAAGCGGGGTGAGAAAGAGGAGACAG
AATATGAGTACAGCGGCAGCGAGGAGGAAGATGACAGCCATGGAGAGGAAGGAGAGCCAAGCTC
CATCATGAACGTGCCTGGAGAGTCGACTCTACGCCGGGAGTTTCTCCGGCTCCAGCAGGAAAATA
AGAGCAACTCAGAGGCTTTAAAACAGCAGCAGCAGCTGCAGCAGCAGCAGCAGCGAGACCCCGA
GGCACACATCAAACACCTGCTGCACCAGCGGCAGCGGCGCATAGAGGAGCAGAAGGAGGAGCG
GCGCCGCGTGGAGGAGCAACAGCGGCGGGAGCGGGAGCAGCGGAAGCTGCAGGAGAAGGAGC
AGCAGCGGCGGCTGGAGGACATGCAGGCTCTGCGGCGGGAGGAGGAGCGGCGGCAGGCGGAG
CGCGAGCAGGAATACAAGCGGAAGCAGCTGGAGGAGCAGCGGCAGTCAGAACGTCTCCAGAGG
CAGCTGCAGCAGGAGCATGCCTACCTCAAGTCCCTGCAGCAGCAGCAACAGCAGCAGCAGCTTC
AGAAACAGCAGCAGCAGCAGCTCCTGCCTGGGGACAGGAAGCCCCTGTACCATTATGGTCGGGG
CATGAATCCCGCTGACAAACCAGCCTGGGCCCGAGAGGTAGAAGAGAGAACAAGGATGAACAAG
CAGCAGAACTCTCCCTTGGCCAAGAGCAAGCCAGGCAGCACGGGGCCTGAGCCCCCCATCCCCC
AGGCCTCCCCAGGGCCCCCAGGACCCCTTTCCCAGACTCCTCCTATGCAGAGGCCGGTGGAGCC
CCAGGAGGGACCGCACAAGAGCCTGGTGGCACACCGGGTCCCACTGAAGCCATATGCAGCACCT
GTACCCCGATCCCAGTCCCTGCAGGACCAGCCCACCCGAAACCTGGCTGCCTTCCCAGCCTCCC
ATGACCCCGACCCTGCCATCCCCGCACCCACTGCCACGCCCAGTGCCCGAGGAGCTGTCATCCG
CCAGAATTCAGACCCCACCTCTGAAGGACCTGGCCCCAGCCCGAATCCCCCAGCCTGGGTCCGC
CCAGATAACGAGGCCCCACCCAAGGTGCCTCAGAGGACCTCATCTATCGCCACTGCCCTTAACAC
CAGTGGGGCCGGAGGGTCCCGGCCAGCCCAGGCAGTCCGTGCCAGACCTCGCAGCAACTCCGC
CTGGCAAATCTATCTGCAAAGGCGGGCAGAGCGGGGCACCCCAAAGCCTCCAGGGCCCCCTGCT
CAGCCCCCTGGCCCGCCCAACGCCTCTAGTAACCCCGACCTCAGGAGGAGCGACCCTGGCTGGG
AACGCTCGGACAGCGTCCTTCCAGCCTCTCACGGGCACCTCCCCCAGGCTGGCTCACTGGAGCG
GAACCGCGTGGGAGCCTCCTCCAAACTGGACAGCTCCCCTGTGCTCTCCCCTGGGAATAAAGCCA
AGCCCGACGACCACCGCTCACGGCCAGGCCGGCCCGCAGACTTTGTGTTGCTGAAAGAGCGGAC
TCTGGACGAGGCCCCTCGGCCTCCCAAGAAGGCCATGGACTACTCGTCGTCCAGCGAGGAGGTG
GAAAGCAGTGAGGACGACGAGGAGGAAGGCGAAGGCGGGCCAGCAGAGGGGAGCAGAGATACC
CCTGGGGGCCGCAGCGATGGGGATACAGACAGCGTCAGCACCATGGTGGTCCACGACGTCGAG
GAGATCACCGGGACCCAGCCCCCATACGGGGGCGGCACCATGGTGGTCCAGCGCACCCCTGAA
GAGGAGCGGAACCTGCTGCATGCTGACAGCAATGGGTACACAAACCTGCCTGACGTGGTCCAGC
CCAGCCACTCACCCACCGAGAACAGCAAAGGCCAAAGCCCACCCTCGAAGGATGGGAGTGGTGA
CTACCAGTCTCGTGGGCTGGTAAAGGCCCCTGGCAAGAGCTCGTTCACGATGTTTGTGGATCTAG
GGATCTACCAGCCTGGAGGCAGTGGGGACAGCATCCCCATCACAGCCCTAGTGGGTGGAGAGGG
CACTCGGCTCGACCAGCTGCAGTACGACGTGAGGAAGGGTTCTGTGGTCAACGTGAATCCCACCA
ACACCCGGGCCCACAGTGAGACCCCTGAGATCCGGAAGTACAAGAAGCGATTCAACTCCGAGATC
CTCTGTGCAGCCCTTTGGGGGGTCAACCTGCTGGTGGGCACGGAGAACGGGCTGATGTTGCTGG
ACCGAAGTGGGCAGGGCAAGGTGTATGGACTCATTGGGCGGCGACGCTTCCAGCAGATGGATGT
GCTGGAGGGGCTCAACCTGCTCATCACCATCTCAGGGAAAAGGAACAAACTGCGGGTGTATTACC
TGTCCTGGCTCCGGAACAAGATTCTGCACAATGACCCAGAAGTGGAGAAGAAGCAGGGCTGGAC
CACCGTGGGGGACATGGAGGGCTGCGGGCACTACCGTGTTGTGAAATACGAGCGGATTAAGTTC
CTGGTCATCGCCCTCAAGAGCTCCGTGGAGGTGTATGCCTGGGCCCCCAAACCCTACCACAAATT
CATGGCCTTCAAGTCCTTTGCCGACCTCCCCCACCGCCCTCTGCTGGTCGACCTGACAGTAGAGG
AGGGGCAGCGGCTCAAGGTCATCTATGGCTCCAGTGCTGGCTTCCATGCTGTGGATGTCGACTCG
GGGAACAGCTATGACATCTACATCCCTGTGCACATCCAGAGCCAGATCACGCCCCATGCCATCAT
CTTCCTCCCCAACACCGACGGCATGGAGATGCTGCTGTGCTACGAGGACGAGGGTGTCTACGTCA
ACACGTACGGGCGCATCATTAAGGATGTGGTGCTGCAGTGGGGGGAGATGCCTACTTCTGTGGC

telephone +44 (0) 1382 384 241 fax +44 (0) 1382 223 778 www.ppu.mrc.ac.uk

CTACATCTGCTCCAACCAGATAATGGGCTGGGGTGAGAAAGCCATTGAGATCCGCTCTGTGGAGA
CGGGCCACCTCGACGGGGTCTTCATGCACAAACGAGCTCAGAGGCTCAAGTTCCTGTGTGAGCG
GAATGACAAGGTGTTTTTTGCCTCAGTCCGCTCTGGGGGCAGCAGCCAAGTTTACTTCATGACTCT
GAACCGTAACTGCATCATGAACTGGGCGGCCGC

Sequence of
Cassette:
ATGGGCGACCCAGCCCCCGCCCGCAGCCTGGACGACATCGACCTGTCCGCCCTGCGGGACCCT
GCTGGGATCTTTGAGCTTGTGGAGGTGGTCGGCAATGGAACCTACGGACAGGTGTACAAGGGTC
GGCATGTCAAGACGGGGCAGCTGGCTGCCATCAAGGTCATGGATGTCACGGAGGACGAGGAGGA
AGAGATCAAACAGGAGATCAACATGCTGAAAAAGTACTCTCACCACCGCAACATCGCCACCTACTA
CGGAGCCTTCATCAAGAAGAGCCCCCCGGGAAACGATGACCAGCTCTGGCTGGTGATGGAGTTC
TGTGGTGCTGGTTCAGTGACTGACCTGGTAAAGAACACAAAAGGCAACGCCCTGAAGGAGGACTG
TATCGCCTATATCTGCAGGGAGATCCTCAGGGGTCTGGCCCATCTCCATGCCCACAAGGTGATCC
ATCGAGACATCAAGGGGCAGAATGTGCTGCTGACAGAGAATGCTGAGGTCAAGCTAGTGGATTTT
GGGGTGAGTGCTCAGCTGGACCGCACCGTGGGCAGACGGAACACTTTCATTGGGACTCCCTACT
GGATGGCTCCAGAGGTCATCGCCTGTGATGAGAACCCTGATGCCACCTATGATTACAGGAGTGAT
ATTTGGTCTCTAGGAATCACAGCCATCGAGATGGCAGAGGGAGCCCCCCCTCTGTGTGACATGCA
CCCCATGCGAGCCCTCTTCCTCATTCCTCGGAACCCTCCGCCCAGGCTCAAGTCCAAGAAGTGGT
CTAAGAAGTTCATTGACTTCATTGACACATGTCTCATCAAGACTTACCTGAGCCGCCCACCCACGG
AGCAGCTACTGAAGTTTCCCTTCATCCGGGACCAGCCCACGGAGCGGCAGGTCCGCATCCAGCTT
AAGGACCACATTGACCGATCCCGGAAGAAGCGGGGTGAGAAAGAGGAGACAGAATATGAGTACA
GCGGCAGCGAGGAGGAAGATGACAGCCATGGAGAGGAAGGAGAGCCAAGCTCCATCATGAACGT
GCCTGGAGAGTCGACTCTACGCCGGGAGTTTCTCCGGCTCCAGCAGGAAAATAAGAGCAACTCAG
AGGCTTTAAAACAGCAGCAGCAGCTGCAGCAGCAGCAGCAGCGAGACCCCGAGGCACACATCAA
ACACCTGCTGCACCAGCGGCAGCGGCGCATAGAGGAGCAGAAGGAGGAGCGGCGCCGCGTGGA
GGAGCAACAGCGGCGGGAGCGGGAGCAGCGGAAGCTGCAGGAGAAGGAGCAGCAGCGGCGGC
TGGAGGACATGCAGGCTCTGCGGCGGGAGGAGGAGCGGCGGCAGGCGGAGCGCGAGCAGGAA
TACAAGCGGAAGCAGCTGGAGGAGCAGCGGCAGTCAGAACGTCTCCAGAGGCAGCTGCAGCAG
GAGCATGCCTACCTCAAGTCCCTGCAGCAGCAGCAACAGCAGCAGCAGCTTCAGAAACAGCAGCA
GCAGCAGCTCCTGCCTGGGGACAGGAAGCCCCTGTACCATTATGGTCGGGGCATGAATCCCGCT
GACAAACCAGCCTGGGCCCGAGAGGTAGAAGAGAGAACAAGGATGAACAAGCAGCAGAACTCTC
CCTTGGCCAAGAGCAAGCCAGGCAGCACGGGGCCTGAGCCCCCCATCCCCCAGGCCTCCCCAG
GGCCCCCAGGACCCCTTTCCCAGACTCCTCCTATGCAGAGGCCGGTGGAGCCCCAGGAGGGACC
GCACAAGAGCCTGGTGGCACACCGGGTCCCACTGAAGCCATATGCAGCACCTGTACCCCGATCC
CAGTCCCTGCAGGACCAGCCCACCCGAAACCTGGCTGCCTTCCCAGCCTCCCATGACCCCGACC
CTGCCATCCCCGCACCCACTGCCACGCCCAGTGCCCGAGGAGCTGTCATCCGCCAGAATTCAGA
CCCCACCTCTGAAGGACCTGGCCCCAGCCCGAATCCCCCAGCCTGGGTCCGCCCAGATAACGAG
GCCCCACCCAAGGTGCCTCAGAGGACCTCATCTATCGCCACTGCCCTTAACACCAGTGGGGCCG
GAGGGTCCCGGCCAGCCCAGGCAGTCCGTGCCAGACCTCGCAGCAACTCCGCCTGGCAAATCTA
TCTGCAAAGGCGGGCAGAGCGGGGCACCCCAAAGCCTCCAGGGCCCCCTGCTCAGCCCCCTGG
CCCGCCCAACGCCTCTAGTAACCCCGACCTCAGGAGGAGCGACCCTGGCTGGGAACGCTCGGAC
AGCGTCCTTCCAGCCTCTCACGGGCACCTCCCCCAGGCTGGCTCACTGGAGCGGAACCGCGTGG
GAGCCTCCTCCAAACTGGACAGCTCCCCTGTGCTCTCCCCTGGGAATAAAGCCAAGCCCGACGAC
CACCGCTCACGGCCAGGCCGGCCCGCAGACTTTGTGTTGCTGAAAGAGCGGACTCTGGACGAGG
CCCCTCGGCCTCCCAAGAAGGCCATGGACTACTCGTCGTCCAGCGAGGAGGTGGAAAGCAGTGA
GGACGACGAGGAGGAAGGCGAAGGCGGGCCAGCAGAGGGGAGCAGAGATACCCCTGGGGGCC
GCAGCGATGGGGATACAGACAGCGTCAGCACCATGGTGGTCCACGACGTCGAGGAGATCACCGG
GACCCAGCCCCCATACGGGGGCGGCACCATGGTGGTCCAGCGCACCCCTGAAGAGGAGCGGAA
CCTGCTGCATGCTGACAGCAATGGGTACACAAACCTGCCTGACGTGGTCCAGCCCAGCCACTCAC
CCACCGAGAACAGCAAAGGCCAAAGCCCACCCTCGAAGGATGGGAGTGGTGACTACCAGTCTCG
TGGGCTGGTAAAGGCCCCTGGCAAGAGCTCGTTCACGATGTTTGTGGATCTAGGGATCTACCAGC
CTGGAGGCAGTGGGGACAGCATCCCCATCACAGCCCTAGTGGGTGGAGAGGGCACTCGGCTCGA
CCAGCTGCAGTACGACGTGAGGAAGGGTTCTGTGGTCAACGTGAATCCCACCAACACCCGGGCC
CACAGTGAGACCCCTGAGATCCGGAAGTACAAGAAGCGATTCAACTCCGAGATCCTCTGTGCAGC

CCTTTGGGGGGTCAACCTGCTGGTGGGCACGGAGAACGGGCTGATGTTGCTGGACCGAAGTGGG
CAGGGCAAGGTGTATGGACTCATTGGGCGGCGACGCTTCCAGCAGATGGATGTGCTGGAGGGGC
TCAACCTGCTCATCACCATCTCAGGGAAAAGGAACAAACTGCGGGTGTATTACCTGTCCTGGCTCC
GGAACAAGATTCTGCACAATGACCCAGAAGTGGAGAAGAAGCAGGGCTGGACCACCGTGGGGGA
CATGGAGGGCTGCGGGCACTACCGTGTTGTGAAATACGAGCGGATTAAGTTCCTGGTCATCGCCC
TCAAGAGCTCCGTGGAGGTGTATGCCTGGGCCCCCAAACCCTACCACAAATTCATGGCCTTCAAG
TCCTTTGCCGACCTCCCCCACCGCCCTCTGCTGGTCGACCTGACAGTAGAGGAGGGGCAGCGGC
TCAAGGTCATCTATGGCTCCAGTGCTGGCTTCCATGCTGTGGATGTCGACTCGGGGAACAGCTAT
GACATCTACATCCCTGTGCACATCCAGAGCCAGATCACGCCCCATGCCATCATCTTCCTCCCCAAC
ACCGACGGCATGGAGATGCTGCTGTGCTACGAGGACGAGGGTGTCTACGTCAACACGTACGGGC
GCATCATTAAGGATGTGGTGCTGCAGTGGGGGGAGATGCCTACTTCTGTGGCCTACATCTGCTCC
AACCAGATAATGGGCTGGGGTGAGAAAGCCATTGAGATCCGCTCTGTGGAGACGGGCCACCTCG
ACGGGGTCTTCATGCACAAACGAGCTCAGAGGCTCAAGTTCCTGTGTGAGCGGAATGACAAGGTG
TTTTTTGCCTCAGTCCGCTCTGGGGGCAGCAGCCAAGTTTACTTCATGACTCTGAACCGTAACTGC
ATCATGAACTGGGCGGCCGCGATGGTGAGCAAGGGCGAGGAGCTGTTCACCGGGGTGGTGCCC
ATCCTGGTCGAGCTGGACGGCGACGTAAACGGCCACAAGTTCAGCGTGTCCGGCGAGGGCGAG
GGCGATGCCACCTACGGCAAGCTGACCCTGAAGTTCATCTGCACCACCGGCAAGCTGCCCGTGC
CCTGGCCCACCCTCGTGACCACCCTGACCTACGGCGTGCAGTGCTTCAGCCGCTACCCCGACCA
CATGAAGCAGCACGACTTCTTCAAGTCCGCCATGCCCGAAGGCTACGTCCAGGAGCGCACCATCT
TCTTCAAGGACGACGGCAACTACAAGACCCGCGCCGAGGTGAAGTTCGAGGGCGACACCCTGGT
GAACCGCATCGAGCTGAAGGGCATCGACTTCAAGGAGGACGGCAACATCCTGGGGCACAAGCTG
GAGTACAACTACAACAGCCACAACGTCTATATCATGGCCGACAAGCAGAAGAACGGCATCAAGGT
GAACTTCAAGATCCGCCACAACATCGAGGACGGCAGCGTGCAGCTCGCCGACCACTACCAGCAG
AACACCCCCATCGGCGACGGCCCCGTGCTGCTGCCCGACAACCACTACCTGAGCACCCAGTCCG
CCCTGAGCAAAGACCCCAACGAGAAGCGCGATCACATGGTCCTGCTGGAGTTCGTGACCGCCGC
CGGGATCACTCTCGGCATGGACGAGCTGTACAAGTAA

Antibiotic: Amp

Oligos: RT9071/RT9072

Restriction Enzyme: BamH1/Not1

Insert Source: Re-amplified from DU19124

Multi Position: N/A

Precursor: N/A

Contact: Rachel Toth

2. Supporting details

2.1. Radiometric protein kinase filter-binding assay

Modification to the reaction cocktails are the following. All PKC assays (except the PKC-μ and the PKC-ν assay) additionally contained 1 mM $CaCl_2$, 4 mM EDTA, 5 μg/mL Phosphatidylserine and 1 μg/mL 1.2-Dioleyl-glycerol. The MYLK2, CAMK1D, CAMK2A, CAMK2B, CAMK2D, CAMK4, CAMKK2 and DAPK2 assays additionally contained 1 μg/mL Calmodulin and 0.5 mM $CaCl_2$. The PRKG1 and PRKG2 assays additionally contained 1 μM cGMP. The DNA-PK assay additionally contained 2.5 μg/mL DNA.

2.2. Protein and peptide identification

2.2.1. Chymotrypsin digestion and data analysis using the internal database: UKCM.

MAP4K4 chymotrypisin digest

Prot0560

**Database:**	UserUKCM
**Score:**	1394
**Monoisotopic mass (M_r):**	32690
**Calculated pI:**	6.83

Sequence similarity is available as an NCBI BLAST search of Prot0560 against nr.

Search parameters

**MS data file:**	`D:\SCIEX OS Data\MGF files\May2018\300818 LBD GR MAP4K4 chymo.mgf`
**Enzyme:**	Chymotrypsin: cuts C-term side of FLWY unless next residue is P.
**Fixed modifications:**	Carbamidomethyl (C)
**Variable modifications:**	Oxidation (M), Phospho (ST), Phospho (Y)

Protein sequence coverage: 81%

Matched peptides shown in ***bold red***.

1 MHHHHHHGGE NLYF**QGNPGN** **KTIVPATLPQ** **LTPTLVSLLE** **VIEPEVLYAG**

51 **YDSSVPDSTW** **RIMTTLNMLG** **GRQVIAAVKW** **AKAIPGFRNL** **HLDDQMTLL**Q

101 Y**SWMSLMAFA** **LGWRSYRQSS** **ANLLCFAPDL** **IINEQRMTLP** **CMYDQCKHML**

151 **YVSSELHRLQ** **VSYEEYLCMK** **TLLLLSSVPK** **DGLKSQELFD** **EIRMTY**IKEL

201 GKAIVKREGN SSQNWQRFYQ LTKL**LDSMHE** **VVENLLNY**CF QTF**LDKTMSI**

251 **EFPEMLAEII** **TNQIPKYSNG** **NIKKLL**FHQK

Unformatted sequence string: 280 residues (for pasting into other applications).

Query	Start	–	End	Observed	Mr(expt)	Mr(calc)	Delta	M	Score	Expect	Rank	U	Peptide
2503	15	–	38	820.4545	2458.3417	2458.3795	-0.0379	2	63	1.4e-06	1Score **>** **18** indicates **identity**	U	F.QGNPGNKTIVPATLPQLTPTLVSL.L
1262	36	–	47	670.3864	1338.7582	1338.7646	-0.0064	2	25	0.014	1Score **>** **19** indicates **identity**	U	L.VSLLEVIEPEVL.Y
725	39	–	47	520.7974	1039.5803	1039.5801	0.0002	1	28	0.0039	1Score **>** **20** indicates **identity** Score **>** **17** indicates **homology**	U	L.LEVIEPEVL.Y
1029	39	–	48	602.3270	1202.6394	1202.6434	-0.0041	2	55	1.6e-05	1Score **>** **19** indicates **identity**	U	L.LEVIEPEVLY.A
469	40	–	47	464.2548	926.4951	926.4960	-0.0010	0	41	0.00029	1Score **>** **18** indicates **identity**	U	L.EVIEPEVL.Y
816	40	–	48	545.7841	1089.5537	1089.5594	-0.0057	1	40	0.00041	1Score **>** **19** indicates **identity**	U	L.EVIEPEVLY.A
817	40	–	48	545.7854	1089.5563	1089.5594	-0.0030	1	23	0.023	1Score **>** **19** indicates **identity**	U	L.EVIEPEVLY.A
1434	48	–	60	724.3022	1446.5899	1446.5939	-0.0040	2	34	0.0016	1Score **>** **19** indicates **identity**	U	L.YAGYDSSVPDSTW.R
1162	49	–	60	642.7706	1283.5267	1283.5306	-0.0040	1	23	0.022	1Score **>** **19** indicates **identity**	U	Y.AGYDSSVPDSTW.R
617	52	–	60	497.2109	992.4072	992.4087	-0.0015	0	24	0.014	1Score **>** **18** indicates **identity**	U	Y.DSSVPDSTW.R
853	61	–	69	554.7970	1107.5795	1107.5780	0.0015	1	31	0.0028	1Score **>** **18** indicates **identity**	U	W.RIMTTLNML.G + Oxidation (M)
980	61	–	69	586.7776	1171.5406	1171.5494	-0.0088	1	37	0.00094	1Score **>** **19** indicates **identity**	U	W.RIMTTLNML.G + Phospho (ST)
1003	61	–	69	594.7771	1187.5396	1187.5443	-0.0047	1	24	0.022	1Score **>** **20** indicates **identity**	U	W.RIMTTLNML.G + Oxidation (M); Phospho (ST)
1566	67	–	80	514.9572	1541.8497	1541.8500	-0.0003	1	32	0.0018	1Score **>** **17** indicates **identity**	U	L.NMLGGRQVIAAVKW.A
1587	67	–	80	520.2853	1557.8341	1557.8450	-0.0109	1	37	0.00085	1Score **>** **20** indicates **identity** Score **>** **19** indicates **homology**	U	L.NMLGGRQVIAAVKW.A + Oxidation (M)
997	70	–	80	592.8490	1183.6835	1183.6826	0.0009	0	48	6.6e-05	1Score **>** **19** indicates **identity**	U	L.GGRQVIAAVKW.A
79	81	–	87	352.2079	702.4013	702.4064	-0.0051	0	41	8.1e-05	1Score **>** **13** indicates **identity**	U	W.AKAIPGF.R
804	81	–	90	543.8250	1085.6355	1085.6345	0.0009	1	21	0.035	1Score **>** **19** indicates **identity**	U	W.AKAIPGFRNL.H
1284	88	–	98	452.5619	1354.6637	1354.6663	-0.0026	2	37	0.0012	1Score **>** **21** indicates **identity**	U	F.RNLHLDDQMTL.L

Query	Start	End	Observed	Mr(expt)	Mr(calc)	Delta	M	Score	Expect	Rank		U	Peptide
1305	88	98	686.3396	1370.6647	1370.6612	0.0034	2	29	0.0081	1	Score **>** **20** indicates **identity**	U	F.RNLHLDDQMTL.L + Oxidation (M)
568	91	98	486.7257	971.4369	971.4382	-0.0013	1	37	0.00059	1	Score **>** **17** indicates **identity**	U	L.HLDDQMTL.L
610	91	98	494.7244	987.4343	987.4332	0.0011	1	40	0.00032	1	Score **>** **17** indicates **identity**	U	L.HLDDQMTL.L + Oxidation (M)
802	91	99	543.2661	1084.5176	1084.5223	-0.0047	2	41	0.00032	1	Score **>** **18** indicates **identity**	U	L.HLDDQMTLL.Q
842	91	99	551.2642	1100.5139	1100.5172	-0.0033	2	44	0.00015	1	Score **>** **18** indicates **identity**	U	L.HLDDQMTLL.Q + Oxidation (M)
567	102	109	486.7158	971.4171	971.4245	-0.0074	2	23	0.015	1	Score **>** **17** indicates **identity**	U	Y.SWMSLMAF.A
641	102	109	502.7120	1003.4095	1003.4143	-0.0049	2	20	0.033	1	Score **>** **18** indicates **identity**	U	Y.SWMSLMAF.A + 2 Oxidation (M)
582	104	111	490.2014	978.3882	978.3956	-0.0074	2	19	0.043	1	Score **>** **18** indicates **identity**	U	W.MSLMAFAL.G + Oxidation (M); Phospho (ST)
176	107	113	398.1952	794.3759	794.3785	-0.0026	2	42	6.7e-05	1	Score **>** **13** indicates **identity**	U	L.MAFALGW.R
202	107	113	406.1936	810.3727	810.3734	-0.0007	2	37	0.00039	1	Score **>** **15** indicates **identity**	U	L.MAFALGW.R + Oxidation (M)
276	110	116	426.7212	851.4279	851.4290	-0.0011	2	30	0.0011	1	Score **>** **13** indicates **identity**	U	F.ALGWRSY.R
354	117	124	444.7491	887.4836	887.4825	0.0012	1	43	0.00013	1	Score **>** **16** indicates **identity**	U	Y.RQSSANLL.C
355	117	124	444.7496	887.4846	887.4825	0.0021	1	45	7.6e-05	1	Score **>** **16** indicates **identity**	U	Y.RQSSANLL.C
2435	125	143	796.6979	2387.0718	2387.0782	-0.0064	2	23	0.0088	1	Score **>** **21** indicates **identity**<br>Score **>** **15** indicates **homology**	U	L.CFAPDLIINEQRMTLPCMY.D + Oxidation (M)
2449	125	143	802.0296	2403.0668	2403.0731	-0.0063	2	43	0.00016	1	Score **>** **21** indicates **identity**<br>Score **>** **17** indicates **homology**	U	L.CFAPDLIINEQRMTLPCMY.D + 2 Oxidation (M)
2493	125	143	818.0202	2451.0388	2451.0496	-0.0108	2	28	0.0045	1	Score **>** **17** indicates **identity**	U	L.CFAPDLIINEQRMTLPCMY.D + Phospho (ST)
2170	127	143	694.3316	2079.9729	2079.9791	-0.0062	1	34	0.0012	1	Score **>** **18** indicates **identity**	U	F.APDLIINEQRMTLPCMY.D + Oxidation (M)
828	144	151	547.7426	1093.4706	1093.4685	0.0021	1	24	0.0082	1	Score **>** **16** indicates **identity**	U	Y.DQCKHMLY.V
857	144	151	555.7396	1109.4646	1109.4634	0.0012	1	23	0.017	1	Score **>** **18** indicates **identity**	U	Y.DQCKHMLY.V + Oxidation (M)
858	144	151	555.7409	1109.4672	1109.4634	0.0038	1	30	0.0039	1	Score **>** **18** indicates **identity**	U	Y.DQCKHMLY.V + Oxidation (M)
503	152	159	470.7654	939.5162	939.5138	0.0024	1	52	2.1e-05	1	Score **>** **18** indicates **identity**	U	Y.VSSELHRL.Q
394	157	163	451.7467	901.4788	901.4770	0.0018	1	26	0.006	1	Score **>** **16** indicates **identity**	U	L.HRLQVSY.E
1242	157	166	662.3187	1322.6228	1322.6255	-0.0027	2	55	1.1e-05	1	Score **>** **18** indicates **identity**	U	L.HRLQVSYEEY.L
331	167	173	439.7474	877.4803	877.4765	0.0038	2	25	0.0082	1	Score **>** **17** indicates **identity**	U	Y.LCMKTLL.L
365	167	173	447.7438	893.4731	893.4714	0.0017	2	31	0.0022	1	Score **>** **17** indicates **identity**	U	Y.LCMKTLL.L

+ Oxidation (M)

Query	Start	– End	Observed	Mr(expt)	Mr(calc)	Delta	M	Score	Expect	Rank	U	Peptide
694	174	– 183	514.8031	1027.5916	1027.5914	0.0003	2	48	5.1e-05	1 Score **>** **18** indicates **identity**	U	L.LLSSVPKDGL.K
185	176	– 183	401.7206	801.4266	801.4232	0.0034	0	33	0.00062	1 Score **>** **13** indicates **identity**	U	L.SSVPKDGL.K
1339	176	– 188	694.3750	1386.7354	1386.7354	0.0000	1	32	0.0042	1 Score **>** **21** indicates **identity**	U	L.SSVPKDGLKSQEL.F
1553	176	– 189	767.9058	1533.7971	1533.8039	-0.0068	2	24	0.016	1 Score **>** **19** indicates **identity**	U	L.SSVPKDGLKSQELF.D
1768	184	– 196	830.4025	1658.7904	1658.7974	-0.0069	2	51	4.8e-05	1 Score **>** **20** indicates **identity**	U	L.KSQELFDEIRMTY.I
1794	184	– 196	559.2715	1674.7927	1674.7923	0.0004	2	40	0.00077	1 Score **>** **21** indicates **identity**	U	L.KSQELFDEIRMTY.I + Oxidation (M)
785	189	– 196	537.7481	1073.4817	1073.4852	-0.0035	1	52	2.4e-05	1 Score **>** **18** indicates **identity**	U	L.FDEIRMTY.I
813	189	– 196	545.7465	1089.4785	1089.4801	-0.0016	1	22	0.028	1 Score **>** **19** indicates **identity**	U	L.FDEIRMTY.I + Oxidation (M)
814	189	– 196	545.7476	1089.4807	1089.4801	0.0006	1	44	0.00018	1 Score **>** **19** indicates **identity**	U	L.FDEIRMTY.I + Oxidation (M)
467	190	– 196	464.2156	926.4166	926.4167	-0.0001	0	22	0.021	1 Score **>** **18** indicates **identity**	U	F.DEIRMTY.I
468	190	– 196	464.2156	926.4167	926.4167	0.0000	0	38	0.00056	1 Score **>** **18** indicates **identity**	U	F.DEIRMTY.I
509	190	– 196	472.2127	942.4108	942.4117	-0.0009	0	37	0.00063	1 Score **>** **17** indicates **identity**	U	F.DEIRMTY.I + Oxidation (M)
1356	225	– 236	699.8494	1397.6842	1397.6860	-0.0018	2	54	1.4e-05	1 Score **>** **18** indicates **identity**	U	L.LDSMHEVVENLL.N
1381	225	– 236	707.8458	1413.6770	1413.6810	-0.0039	2	43	0.00031	1 Score **>** **20** indicates **identity**	U	L.LDSMHEVVENLL.N + Oxidation (M)
1167	226	– 236	643.3053	1284.5960	1284.6020	-0.0060	1	52	2.9e-05	1 Score **>** **19** indicates **identity**	U	L.DSMHEVVENLL.N
1203	226	– 236	651.3046	1300.5947	1300.5969	-0.0022	1	23	0.01	1 Score **>** **21** indicates **identity** Score **>** **15** indicates **homology**	U	L.DSMHEVVENLL.N + Oxidation (M)
1592	226	– 238	781.8585	1561.7024	1561.7082	-0.0058	2	51	3e-05	1 Score **>** **19** indicates **identity**	U	L.DSMHEVVENLLNY.C
1612	226	– 238	789.8539	1577.6932	1577.7032	-0.0100	2	40	0.0003	1 Score **>** **17** indicates **identity**	U	L.DSMHEVVENLLNY.C + Oxidation (M)
1581	244	– 256	777.3794	1552.7442	1552.7517	-0.0075	1	33	0.0019	1 Score **>** **19** indicates **identity**	U	F.LDKTMSIEFPEML.A
1600	244	– 256	785.3778	1568.7410	1568.7466	-0.0056	1	35	0.0012	1 Score **>** **18** indicates **identity**	U	F.LDKTMSIEFPEML.A + Oxidation (M)
1621	244	– 256	529.2529	1584.7368	1584.7415	-0.0047	1	19	0.039	1 Score **>** **20** indicates **identity** Score **>** **18** indicates **homology**	U	F.LDKTMSIEFPEML.A + 2 Oxidation (M)
2681	244	– 267	942.1494	2823.4263	2823.4438	-0.0175	2	54	9.2e-06	1 Score **>** **16** indicates **identity**	U	F.LDKTMSIEFPEMLAEIITNQIPKY.S
2692	244	– 267	947.4801	2839.4186	2839.4387	-0.0201	2	55	1e-05	1 Score **>** **18** indicates **identity**	U	F.LDKTMSIEFPEMLAEIITNQIPKY.S + Oxidation (M)
2693	244	– 267	947.4803	2839.4192	2839.4387	-0.0195	2	33	0.0017	1 Score **>** **18** indicates **identity**	U	F.LDKTMSIEFPEMLAEIITNQIPKY.S + Oxidation (M)
2698	244	– 267	952.8115	2855.4125	2855.4336	-0.0211	2	58	4.3e-06	1 Score **>** **16** indicates **identity**	U	F.LDKTMSIEFPEMLAEIITNQIPKY.S

Query	Start – End	Observed	Mr(expt)	Mr(calc)	Delta	M	Score	Expect	Rank	U	Peptide
1447	245 – 256	728.8359	1455.6573	1455.6625	-0.0052	0	29	0.0041	1	Score > 20 indicates identity Score > 18 indicates homology	U	L.DKTMSIEFPEML.A + 2 Oxidation (M)
1176	257 – 267	645.3560	1288.6975	1288.7027	-0.0051	0	66	9.9e-07	1	Score > 18 indicates identity	U	L.AEIITNQIPKY.S + Oxidation (M)
602	268 – 276	493.8038	985.5930	985.5920	0.0010	1	54	7.7e-06	1	Score > 15 indicates identity	U	Y.SNGNIKKLL.F

MINK1 chymotrypisin digest

Prot0560

**Database:**	UserUKCM
**Score:**	1385
**Monoisotopic mass (M_r):**	32690
**Calculated pI:**	6.83

Sequence similarity is available as an NCBI BLAST search of Prot0560 against nr.

Search parameters

**MS data file:**	D:\SCIEX OS Data\MGF files\May2018\300818 LBD GR MINK1 chymo.mgf
**Enzyme:**	Chymotrypsin: cuts C-term side of FLWY unless next residue is P.
**Fixed modifications:**	Carbamidomethyl (C)
**Variable modifications:**	Oxidation (M), Phospho (ST), Phospho (Y)

Protein sequence coverage: 79%

Matched peptides shown in ***bold red***.

1 MHHHHHHGGE NLYFQGNPGN KTIVPATLPQ LTPTLVSL**LE** **VIEPEVLYAG**

51 **YDSSVPDSTW RIMTTLNMLG GRQVIAAVKW AKAIPGFRNL HLDDQMTLL**Q

101 Y**SWMSLMAFA LGWRSYRQSS ANLLCFAPDL IINEQRMTLP CMYDQCKHML**

151 **YVSSELHRLQ VSYEEYLCMK TLLLLSSVPK DGLKSQELFD EIRMTYIKEL**

201 **GKAIVKREGN SSQNW**QRFYQ LTKL**LDSMHE VVENLLNY**CF QTF**LDKTMSI**

251 **EFPEMLAEII TNQIPKYSNG NIKKLL**FHQK

Unformatted sequence string: 280 residues (for pasting into other applications).

Query	Start	-	End	Observed	Mr(expt)	Mr(calc)	Delta	M	Score	Expect	Rank	U	Peptide
645	39	-	47	520.7938	1039.5731	1039.5801	-0.0070	1	27	0.01	1Score **> 20** indicates **identity**	U	L.LEVIEPEVL.Y
917	39	-	48	602.3267	1202.6388	1202.6434	-0.0046	2	67	8.7e-07	1Score **> 19** indicates **identity**	U	L.LEVIEPEVLY.A
412	40	-	47	464.2535	926.4925	926.4960	-0.0035	0	24	0.014	1Score **> 18** indicates **identity**	U	L.EVIEPEVL.Y
734	40	-	48	545.7848	1089.5551	1089.5594	-0.0042	1	41	0.00034	1Score **> 19** indicates **identity**	U	L.EVIEPEVLY.A
1299	48	-	60	724.2996	1446.5847	1446.5939	-0.0092	2	29	0.0051	1Score **> 19** indicates **identity**	U	L.YAGYDSSVPDSTW.R
544	52	-	60	497.2101	992.4056	992.4087	-0.0031	0	23	0.016	1Score **> 18** indicates **identity**	U	Y.DSSVPDSTW.R
545	52	-	60	497.2104	992.4062	992.4087	-0.0025	0	24	0.013	1Score **> 18** indicates **identity**	U	Y.DSSVPDSTW.R
762	61	-	69	554.7964	1107.5783	1107.5780	0.0002	1	22	0.022	1Score **> 18** indicates **identity**	U	W.RIMTTLNML.G<br>+ Oxidation (M)
798	61	-	69	562.7926	1123.5707	1123.5729	-0.0023	1	27	0.006	1Score **> 17** indicates **identity**	U	W.RIMTTLNML.G<br>+ 2 Oxidation (M)
875	61	-	69	586.7799	1171.5452	1171.5494	-0.0043	1	43	0.00022	1Score **> 19** indicates **identity**	U	W.RIMTTLNML.G<br>+ Phospho (ST)
893	61	-	69	594.7775	1187.5404	1187.5443	-0.0040	1	38	0.00098	1Score **> 20** indicates **identity**	U	W.RIMTTLNML.G<br>+ Oxidation (M); Phospho (ST)
894	61	-	69	594.7776	1187.5406	1187.5443	-0.0038	1	17	0.025	1Score **> 20** indicates **identity**<br>Score **> 14** indicates **homology**	U	W.RIMTTLNML.G<br>+ Oxidation (M); Phospho (ST)
918	61	-	69	602.7718	1203.5291	1203.5393	-0.0102	1	20	0.038	1Score **> 18** indicates **identity**	U	W.RIMTTLNML.G<br>+ 2 Oxidation (M); Phospho (ST)
2123	61	-	80	593.3087	2369.2055	2369.2113	-0.0058	2	23	0.016	1Score **> 18** indicates **identity**	U	W.RIMTTLNMLGGRQVIAAVKW.A<br>+ 2 Oxidation (M); Phospho (ST)
1409	67	-	80	514.9528	1541.8367	1541.8500	-0.0133	1	32	0.0015	1Score **> 17** indicates **identity**	U	L.NMLGGRQVIAAVKW.A
1427	67	-	80	520.2852	1557.8337	1557.8450	-0.0112	1	41	0.00029	1Score **> 20** indicates **identity**<br>Score **> 18** indicates **homology**	U	L.NMLGGRQVIAAVKW.A<br>+ Oxidation (M)
889	70	-	80	592.8462	1183.6778	1183.6826	-0.0048	0	20	0.047	1Score **> 19** indicates **identity**	U	L.GGRQVIAAVKW.A
890	70	-	80	395.5679	1183.6820	1183.6826	-0.0006	0	50	4.3e-05	1Score **> 19** indicates **identity**	U	L.GGRQVIAAVKW.A

Query	Start	– End	Observed	Mr(expt)	Mr(calc)	Delta	M	Score	Expect	Rank	U	Peptide
73	81	– 87	352.2074	702.4002	702.4064	-0.0063	0	41	8.2e-05	1 Score **>** **13** indicates **identity**	U	W.AKAIPGF.R
724	81	– 90	362.8849	1085.6328	1085.6345	-0.0017	1	32	0.003	1 Score **>** **19** indicates **identity**	U	W.AKAIPGFRNL.H
725	81	– 90	543.8241	1085.6336	1085.6345	-0.0009	1	31	0.0035	1 Score **>** **19** indicates **identity**	U	W.AKAIPGFRNL.H
1158	88	– 98	452.5599	1354.6579	1354.6663	-0.0084	2	42	0.00042	1 Score **>** **21** indicates **identity**	U	F.RNLHLDDQMTL.L
1180	88	– 98	686.3392	1370.6639	1370.6612	0.0027	2	58	4e-06	1 Score **>** **20** indicates **identity** Score **>** **16** indicates **homology**	U	F.RNLHLDDQMTL.L + Oxidation (M)
503	91	– 98	486.7250	971.4354	971.4382	-0.0028	1	20	0.027	1 Score **>** **17** indicates **identity**	U	L.HLDDQMTL.L
504	91	– 98	486.7251	971.4356	971.4382	-0.0027	1	21	0.021	1 Score **>** **17** indicates **identity**	U	L.HLDDQMTL.L
540	91	– 98	494.7233	987.4320	987.4332	-0.0012	1	45	9e-05	1 Score **>** **17** indicates **identity**	U	L.HLDDQMTL.L + Oxidation (M)
721	91	– 99	543.2656	1084.5166	1084.5223	-0.0057	2	42	0.00023	1 Score **>** **18** indicates **identity**	U	L.HLDDQMTLL.Q
750	91	– 99	551.2629	1100.5113	1100.5172	-0.0059	2	35	0.0011	1 Score **>** **18** indicates **identity**	U	L.HLDDQMTLL.Q + Oxidation (M)
571	102	– 109	502.7115	1003.4084	1003.4143	-0.0060	2	30	0.0033	1 Score **>** **18** indicates **identity**	U	Y.SWMSLMAF.A + 2 Oxidation (M)
162	107	– 113	398.1946	794.3747	794.3785	-0.0038	2	30	0.00097	1 Score **>** **13** indicates **identity**	U	L.MAFALGW.R
185	107	– 113	406.1914	810.3683	810.3734	-0.0051	2	42	0.00011	1 Score **>** **15** indicates **identity**	U	L.MAFALGW.R + Oxidation (M)
247	110	– 116	426.7216	851.4286	851.4290	-0.0004	2	34	0.00043	1 Score **>** **13** indicates **identity**	U	F.ALGWRSY.R
1070	114	– 124	432.2351	1293.6834	1293.6789	0.0045	2	21	0.03	1 Score **>** **19** indicates **identity**	U	W.RSYRQSSANLL.C
321	117	– 124	444.7483	887.4821	887.4825	-0.0004	1	50	2.3e-05	1 Score **>** **16** indicates **identity**	U	Y.RQSSANLL.C
322	117	– 124	444.7491	887.4837	887.4825	0.0013	1	41	0.00019	1 Score **>** **16** indicates **identity**	U	Y.RQSSANLL.C
2147	125	– 143	802.0271	2403.0594	2403.0731	-0.0137	2	35	0.00091	1 Score **>** **21** indicates **identity** Score **>** **17** indicates **homology**	U	L.CFAPDLIINEQRMTLPCMY.D + 2 Oxidation (M)
2178	125	– 143	818.0198	2451.0374	2451.0496	-0.0122	2	29	0.0038	1 Score **>** **17** indicates **identity**	U	L.CFAPDLIINEQRMTLPCMY.D + Phospho (ST)
2200	125	– 143	828.6825	2483.0258	2483.0394	-0.0137	2	39	0.00052	1 Score **>** **19** indicates **identity**	U	L.CFAPDLIINEQRMTLPCMY.D + 2 Oxidation (M); Phospho (ST)
1932	127	– 143	694.3312	2079.9717	2079.9791	-0.0074	1	40	0.00032	1 Score **>** **18** indicates **identity**	U	F.APDLIINEQRMTLPCMY.D + Oxidation (M)
1969	127	– 143	726.3151	2175.9235	2175.9404	-0.0168	1	24	0.016	1 Score **>** **19** indicates **identity**	U	F.APDLIINEQRMTLPCMY.D + 2 Oxidation (M); Phospho (ST)
429	144	– 150	466.2120	930.4094	930.4052	0.0042	0	22	0.035	1 Score **>** **20** indicates **identity**	U	Y.DQCKHML.Y
741	144	– 151	547.7424	1093.4702	1093.4685	0.0017	1	25	0.0072	1 Score **>** **16** indicates **identity**	U	Y.DQCKHMLY.V
764	144	– 151	555.7395	1109.4645	1109.4634	0.0011	1	32	0.0023	1 Score **>** **18** indicates **identity**	U	Y.DQCKHMLY.V + Oxidation (M)
765	144	– 151	555.7420	1109.4695	1109.4634	0.0061	1	31	0.0028	1 Score **>** **18** indicates **identity**	U	Y.DQCKHMLY.V

Query	Start	–	End	Observed	Mr(expt)	Mr(calc)	Delta	M	Score	Expect	Rank		U	Peptide
														+ Oxidation (M)
444	152	–	159	470.7651	939.5156	939.5138	0.0018	1	61	2.6e-06	1	Score **>** **18** indicates **identity**	U	Y.VSSELHRL.Q
349	157	–	163	451.7463	901.4780	901.4770	0.0010	1	37	0.00049	1	Score **>** **16** indicates **identity**	U	L.HRLQVSY.E
1115	157	–	166	662.3187	1322.6229	1322.6255	-0.0026	2	61	3e-06	1	Score **>** **18** indicates **identity**	U	L.HRLQVSYEEY.L
299	167	–	173	439.7447	877.4748	877.4765	-0.0017	2	25	0.0085	1	Score **>** **17** indicates **identity**	U	Y.LCMKTLL.L
326	167	–	173	447.7430	893.4715	893.4714	0.0000	2	43	0.00013	1	Score **>** **17** indicates **identity**	U	Y.LCMKTLL.L + Oxidation (M)
616	174	–	183	514.8026	1027.5907	1027.5914	-0.0007	2	39	0.00044	1	Score **>** **18** indicates **identity**	U	L.LLSSVPKDGL.K
1365	175	–	188	750.9111	1499.8077	1499.8195	-0.0118	2	35	0.0017	1	Score **>** **20** indicates **identity**	U	L.LSSVPKDGLKSQEL.F
170	176	–	183	401.7192	801.4239	801.4232	0.0006	0	33	0.00062	1	Score **>** **13** indicates **identity**	U	L.SSVPKDGL.K
1622	184	–	196	559.2692	1674.7856	1674.7923	-0.0067	2	44	0.00011	1	Score **>** **21** indicates **identity**<br>Score **>** **17** indicates **homology**	U	L.KSQELFDEIRMTY.I + Oxidation (M)
705	189	–	196	537.7474	1073.4802	1073.4852	-0.0050	1	52	2.5e-05	1	Score **>** **18** indicates **identity**	U	L.FDEIRMTY.I
733	189	–	196	545.7470	1089.4795	1089.4801	-0.0006	1	44	0.00018	1	Score **>** **19** indicates **identity**	U	L.FDEIRMTY.I + Oxidation (M)
409	190	–	196	464.2121	926.4097	926.4167	-0.0070	0	33	0.0017	1	Score **>** **18** indicates **identity**	U	F.DEIRMTY.I
410	190	–	196	464.2159	926.4173	926.4167	0.0005	0	19	0.047	1	Score **>** **18** indicates **identity**	U	F.DEIRMTY.I
450	190	–	196	472.2129	942.4112	942.4117	-0.0005	0	35	0.00097	1	Score **>** **17** indicates **identity**	U	F.DEIRMTY.I + Oxidation (M)
1959	197	–	215	540.0498	2156.1701	2156.1702	-0.0001	1	31	0.0023	1	Score **>** **20** indicates **identity**<br>Score **>** **17** indicates **homology**	U	Y.IKELGKAIVKREGNSSQNW.Q
1225	225	–	236	699.8463	1397.6781	1397.6860	-0.0080	2	55	1.1e-05	1	Score **>** **18** indicates **identity**	U	L.LDSMHEVVENLL.N
1252	225	–	236	707.8464	1413.6783	1413.6810	-0.0027	2	40	0.00054	1	Score **>** **20** indicates **identity**	U	L.LDSMHEVVENLL.N + Oxidation (M)
1054	226	–	236	643.3051	1284.5957	1284.6020	-0.0063	1	42	0.00032	1	Score **>** **19** indicates **identity**	U	L.DSMHEVVENLL.N
1085	226	–	236	651.3050	1300.5955	1300.5969	-0.0014	1	44	0.00032	1	Score **>** **21** indicates **identity**	U	L.DSMHEVVENLL.N + Oxidation (M)
1430	226	–	238	781.8576	1561.7006	1561.7082	-0.0076	2	53	2.3e-05	1	Score **>** **19** indicates **identity**	U	L.DSMHEVVENLLNY.C
1447	226	–	238	789.8570	1577.6995	1577.7032	-0.0036	2	45	9.5e-05	1	Score **>** **17** indicates **identity**	U	L.DSMHEVVENLLNY.C + Oxidation (M)
1422	244	–	256	518.5870	1552.7392	1552.7517	-0.0125	1	21	0.032	1	Score **>** **19** indicates **identity**	U	F.LDKTMSIEFPEML.A
1434	244	–	256	785.3771	1568.7397	1568.7466	-0.0069	1	41	0.00033	1	Score **>** **18** indicates **identity**	U	F.LDKTMSIEFPEML.A + Oxidation (M)
1456	244	–	256	793.3745	1584.7344	1584.7415	-0.0071	1	29	0.0071	1	Score **>** **20** indicates **identity**	U	F.LDKTMSIEFPEML.A + 2 Oxidation (M)
2340	244	–	267	706.8652	2823.4316	2823.4438	-0.0122	2	20	0.021	1	Score **>** **16** indicates **identity**	U	F.LDKTMSIEFPEMLAEIITNQIPKY.S
2350	244	–	267	947.4780	2839.4121	2839.4387	-0.0266	2	60	3.3e-06	1	Score **>** **18** indicates **identity**	U	F.LDKTMSIEFPEMLAEIITNQIPKY.S + Oxidation (M)

Query	Start	– End	Observed	Mr (expt)	Mr (calc)	Delta	M	Score	Expect	Rank	U	Peptide
2354	244	– 267	952.8113	2855.4121	2855.4336	-0.0215	2	37	0.00046	1Score > **16** indicates **identity**	U	F.LDKTMSIEFPEMLAEIITNQIPKY.S + 2 Oxidation (M)
1308	245	– 256	728.8318	1455.6491	1455.6625	-0.0135	0	31	0.0021	1Score > **20** indicates **identity** Score > **17** indicates **homology**	U	L.DKTMSIEFPEML.A + Oxidation (M)
1064	257	– 267	645.3538	1288.6931	1288.7027	-0.0096	0	63	2e-06	1Score > **18** indicates **identity**	U	L.AEIITNQIPKY.S
532	268	– 276	493.8043	985.5940	985.5920	0.0020	1	37	0.00039	1Score > **15** indicates **identity**	U	Y.SNGNIKKLL.F

MST1 chymotrypisin digest

Prot0560

**Database:**	UserUKCM
**Score:**	1460
**Monoisotopic mass (M_r):**	32690
**Calculated pI:**	6.83

Sequence similarity is available as an NCBI BLAST search of Prot0560 against nr.

Search parameters

**MS data file:**	D:\SCIEX OS Data\MGF files\May2018\300818 LBD GR MST1 chymo.mgf
**Enzyme:**	Chymotrypsin: cuts C-term side of FLWY unless next residue is P.
**Fixed modifications:**	Carbamidomethyl (C)
**Variable modifications:**	Oxidation (M), Phospho (ST), Phospho (Y)

Protein sequence coverage: 80%

Matched peptides shown in ***bold red***.

1 MHHHHHHGGE NLYFQGNPGN KTIVPATLPQ LTPTL**VSLLE** **VIEPEVLYAG**

51 **YDSSVPDSTW** **RIMTTLNMLG** **GRQVIAAVKW** **AKAIPGFRNL** **HLDDQMTLL**Q

101 Y**SWMSLMAFA** **LGWRSYRQSS** **ANLLCFAPDL** **IINEQRMTLP** **CMYDQCKHML**

151 **YVSSELHRLQ** **VSYEEYLCMK** **TLLLLSSVPK** **DGLKSQELFD** **EIRMTYIKEL**

201 **GKAIVKREGN** **SSQNW**QRFYQ LTKL**LDSMHE** **VVENLLNY**CF QTF**LDKTMSI**

251 **EFPEMLAEII** **TNQIPKYSNG** **NIKKLL**FHQK

Unformatted sequence string: 280 residues (for pasting into other applications).

Query	Start	- End	Observed	Mr(expt)	Mr(calc)	Delta	M	Score	Expect	Rank	U	Peptide
1096	36	- 47	670.3850	1338.7554	1338.7646	-0.0092	2	22	0.031	1	Score > 19 indicates identity	U	L.VSLLEVIEPEVL.Y
607	39	- 47	520.7932	1039.5719	1039.5801	-0.0082	1	51	4.1e-05	1	Score > 20 indicates identity	U	L.LEVIEPEVL.Y
883	39	- 48	602.3250	1202.6355	1202.6434	-0.0079	2	57	8.5e-06	1	Score > 19 indicates identity	U	L.LEVIEPEVLY.A
386	40	- 47	464.2535	926.4924	926.4960	-0.0036	0	33	0.0017	1	Score > 18 indicates identity	U	L.EVIEPEVL.Y
695	40	- 48	545.7824	1089.5503	1089.5594	-0.0091	1	40	0.00038	1	Score > 19 indicates identity	U	L.EVIEPEVLY.A
1236	48	- 60	724.2996	1446.5847	1446.5939	-0.0093	2	25	0.012	1	Score > 19 indicates identity	U	L.YAGYDSSVPDSTW.R
508	52	- 60	497.2099	992.4053	992.4087	-0.0034	0	25	0.01	1	Score > 18 indicates identity	U	Y.DSSVPDSTW.R
725	61	- 69	554.7942	1107.5739	1107.5780	-0.0041	1	55	1.2e-05	1	Score > 18 indicates identity	U	W.RIMTTLNML.G + Oxidation (M)
845	61	- 69	586.7805	1171.5464	1171.5494	-0.0031	1	36	0.0013	1	Score > 19 indicates identity	U	W.RIMTTLNML.G + Phospho (ST)
859	61	- 69	594.7767	1187.5388	1187.5443	-0.0055	1	29	0.0082	1	Score > 20 indicates identity	U	W.RIMTTLNML.G + Oxidation (M); Phospho (ST)
860	61	- 69	594.7776	1187.5406	1187.5443	-0.0038	1	24	0.0058	1	Score > 20 indicates identity Score > 15 indicates **homology**	U	W.RIMTTLNML.G + Oxidation (M); Phospho (ST)
2115	61	- 80	593.3078	2369.2020	2369.2113	-0.0092	2	32	0.002	1	Score > 18 indicates identity	U	W.RIMTTLNMLGGRQVIAAVKW.A + 2 Oxidation (M); Phospho (ST)
1364	67	- 80	514.9562	1541.8467	1541.8500	-0.0033	1	29	0.0036	1	Score > 17 indicates identity	U	L.NMLGGRQVIAAVKW.A
1380	67	- 80	779.9268	1557.8391	1557.8450	-0.0059	1	55	6.3e-06	1	Score > 20 indicates identity Score > 16 indicates **homology**	U	L.NMLGGRQVIAAVKW.A + Oxidation (M)
857	70	- 80	395.5676	1183.6809	1183.6826	-0.0017	0	55	1.5e-05	1	Score > 19 indicates identity	U	L.GGRQVIAAVKW.A

Query	Start	-	End	Observed	Mr(expt)	Mr(calc)	Delta	M	Score	Expect	Rank	U	Peptide
65	81	-	87	352.2070	702.3994	702.4064	-0.0071	0	41	8.1e-05	1 Score **>** **13** indicates **identity**	U	W.AKAIPGF.R
684	81	-	90	543.8210	1085.6275	1085.6345	-0.0070	1	29	0.0059	1 Score **>** **19** indicates **identity**	U	W.AKAIPGFRNL.H
685	81	-	90	362.8852	1085.6338	1085.6345	-0.0007	1	29	0.0054	1 Score **>** **19** indicates **identity**	U	W.AKAIPGFRNL.H
1117	88	-	98	452.5617	1354.6634	1354.6663	-0.0030	2	43	0.00036	1 Score **>** **21** indicates **identity**	U	F.RNLHLDDQMTL.L
1130	88	-	98	686.3355	1370.6565	1370.6612	-0.0048	2	41	0.00025	1 Score **>** **20** indicates **identity** Score **>** **17** indicates **homology**	U	F.RNLHLDDQMTL.L + Oxidation (M)
467	91	-	98	486.7247	971.4349	971.4382	-0.0033	1	45	9e-05	1 Score **>** **17** indicates **identity**	U	L.HLDDQMTL.L
504	91	-	98	494.7240	987.4335	987.4332	0.0003	1	42	0.00017	1 Score **>** **17** indicates **identity**	U	L.HLDDQMTL.L + Oxidation (M)
683	91	-	99	543.2644	1084.5142	1084.5223	-0.0081	2	40	0.00038	1 Score **>** **18** indicates **identity**	U	L.HLDDQMTLL.Q
503	102	-	109	494.7150	987.4154	987.4194	-0.0040	2	28	0.0042	1 Score **>** **17** indicates **identity**	U	Y.SWMSLMAF.A + Oxidation (M)
532	102	-	109	502.7110	1003.4074	1003.4143	-0.0070	2	23	0.02	1 Score **>** **18** indicates **identity**	U	Y.SWMSLMAF.A + 2 Oxidation (M)
135	107	-	113	398.1936	794.3726	794.3785	-0.0059	2	32	0.00062	1 Score **>** **13** indicates **identity**	U	L.MAFALGW.R
153	107	-	113	406.1914	810.3683	810.3734	-0.0051	2	42	0.00012	1 Score **>** **15** indicates **identity**	U	L.MAFALGW.R + Oxidation (M)
215	110	-	116	426.7208	851.4270	851.4290	-0.0019	2	23	0.0053	1 Score **>** **13** indicates **identity**	U	F.ALGWRSY.R
285	117	-	124	444.7487	887.4829	887.4825	0.0004	1	46	5.7e-05	1 Score **>** **16** indicates **identity**	U	Y.RQSSANLL.C
286	117	-	124	444.7495	887.4844	887.4825	0.0020	1	36	0.0006	1 Score **>** **16** indicates **identity**	U	Y.RQSSANLL.C
453	117	-	124	484.7287	967.4429	967.4488	-0.0058	1	23	0.012	1 Score **>** **16** indicates **identity**	U	Y.RQSSANLL.C + Phospho (ST)
454	117	-	124	484.7296	967.4446	967.4488	-0.0041	1	20	0.024	1 Score **>** **16** indicates **identity**	U	Y.RQSSANLL.C + Phospho (ST)
2137	125	-	143	796.6951	2387.0635	2387.0782	-0.0147	2	30	0.0031	1 Score **>** **21** indicates **identity** Score **>** **18** indicates **homology**	U	L.CFAPDLIINEQRMTLPCMY.D + Oxidation (M)
2148	125	-	143	802.0277	2403.0613	2403.0731	-0.0118	2	33	0.0028	1 Score **>** **21** indicates **identity** Score **>** **20** indicates **homology**	U	L.CFAPDLIINEQRMTLPCMY.D + 2 Oxidation (M)
2185	125	-	143	818.0169	2451.0287	2451.0496	-0.0209	2	19	0.035	1 Score **>** **17** indicates **identity**	U	L.CFAPDLIINEQRMTLPCMY.D + Phospho (ST)
2201	125	-	143	823.3496	2467.0270	2467.0445	-0.0175	2	21	0.036	1 Score **>** **21** indicates **identity** Score **>** **19** indicates **homology**	U	L.CFAPDLIINEQRMTLPCMY.D + Oxidation (M); Phospho (ST)
2212	125	-	143	828.6818	2483.0237	2483.0394	-0.0158	2	24	0.018	1 Score **>** **19** indicates **identity**	U	L.CFAPDLIINEQRMTLPCMY.D + 2 Oxidation (M); Phospho (ST)

Query	Start – End	Observed	Mr(expt)	Mr(calc)	Delta	M	Score	Expect	Rank	U	Peptide
1877	127 – 143	689.0015	2063.9828	2063.9842	-0.0014	1	26	0.0092	1Score > 18 indicates identity	U	F.APDLIINEQRMTLPCMY.D
1886	127 – 143	694.3272	2079.9599	2079.9791	-0.0192	1	29	0.0044	1Score > 18 indicates identity	U	F.APDLIINEQRMTLPCMY.D + Oxidation (M)
1898	127 – 143	1048.9874	2095.9602	2095.9740	-0.0139	1	24	0.022	1Score > 20 indicates identity	U	F.APDLIINEQRMTLPCMY.D + 2 Oxidation (M)
402	144 – 150	466.2102	930.4059	930.4052	0.0008	0	19	0.04	1Score > 20 indicates identity Score > 17 indicates homology	U	Y.DQCKHML.Y
701	144 – 151	547.7409	1093.4673	1093.4685	-0.0012	1	28	0.0031	1Score > 16 indicates identity	U	Y.DQCKHMLY.V
727	144 – 151	555.7389	1109.4632	1109.4634	-0.0002	1	32	0.0025	1Score > 18 indicates identity	U	Y.DQCKHMLY.V + Oxidation (M)
728	144 – 151	555.7410	1109.4674	1109.4634	0.0040	1	34	0.0015	1Score > 18 indicates identity	U	Y.DQCKHMLY.V + Oxidation (M)
417	152 – 159	470.7635	939.5125	939.5138	-0.0012	1	39	0.00048	1Score > 18 indicates identity	U	Y.VSSELHRL.Q
1190	152 – 163	473.2510	1416.7311	1416.7361	-0.0050	2	36	0.0012	1Score > 19 indicates identity	U	Y.VSSELHRLQVSY.E
321	157 – 163	451.7457	901.4769	901.4770	-0.0001	1	32	0.0016	1Score > 16 indicates identity	U	L.HRLQVSY.E
1070	157 – 166	662.3186	1322.6227	1322.6255	-0.0027	2	58	6e-06	1Score > 18 indicates identity	U	L.HRLQVSYEEY.L
1071	157 – 166	441.8815	1322.6228	1322.6255	-0.0027	2	26	0.009	1Score > 18 indicates identity	U	L.HRLQVSYEEY.L
264	167 – 173	439.7444	877.4743	877.4765	-0.0022	2	36	0.00067	1Score > 17 indicates identity	U	Y.LCMKTLL.L
296	167 – 173	447.7427	893.4708	893.4714	-0.0007	2	32	0.0019	1Score > 17 indicates identity	U	Y.LCMKTLL.L + Oxidation (M)
580	174 – 183	514.8010	1027.5874	1027.5914	-0.0039	2	35	0.0011	1Score > 18 indicates identity	U	L.LLSSVPKDGL.K
141	176 – 183	401.7185	801.4224	801.4232	-0.0009	0	37	0.00027	1Score > 13 indicates identity	U	L.SSVPKDGL.K
1355	176 – 189	512.2738	1533.7995	1533.8039	-0.0043	2	27	0.0076	1Score > 19 indicates identity	U	L.SSVPKDGLKSQELF.D
1546	184 – 196	830.4020	1658.7894	1658.7974	-0.0079	2	54	2.6e-05	1Score > 20 indicates identity	U	L.KSQELFDEIRMTY.I
1572	184 – 196	559.2694	1674.7862	1674.7923	-0.0061	2	40	0.00033	1Score > 21 indicates identity Score > 17 indicates homology	U	L.KSQELFDEIRMTY.I + Oxidation (M)
666	189 – 196	537.7477	1073.4809	1073.4852	-0.0042	1	49	4.8e-05	1Score > 18 indicates identity	U	L.FDEIRMTY.I
693	189 – 196	545.7461	1089.4776	1089.4801	-0.0025	1	44	0.00018	1Score > 19 indicates identity	U	L.FDEIRMTY.I + Oxidation (M)
694	189 – 196	545.7464	1089.4783	1089.4801	-0.0018	1	19	0.049	1Score > 19 indicates identity	U	L.FDEIRMTY.I + Oxidation (M)
385	190 – 196	464.2143	926.4141	926.4167	-0.0026	0	36	0.00093	1Score > 18 indicates identity	U	F.DEIRMTY.I

Query	Start	–	End	Observed	Mr(expt)	Mr(calc)	Delta	M	Score	Expect	Rank		U	Peptide
423	190	–	196	472.2121	942.4096	942.4117	-0.0021	0	23	0.015	1	Score > **17** indicates **identity**	U	F.DEIRMTY.I + Oxidation (M)
1920	197	–	215	540.0495	2156.1690	2156.1702	-0.0012	1	17	0.036	1	Score > **20** indicates **identity** Score > **15** indicates **homology**	U	Y.IKELGKAIVKREGNSSQNW.Q
1634	201	–	215	585.2849	1752.8328	1752.8308	0.0020	0	19	0.047	1	Score > **19** indicates **identity**	U	L.GKAIVKREGNSSQNW.Q + Phospho (ST)
1168	225	–	236	699.8454	1397.6762	1397.6860	-0.0099	2	64	1.4e-06	1	Score > **18** indicates **identity**	U	L.LDSMHEVVENLL.N
1188	225	–	236	707.8449	1413.6752	1413.6810	-0.0058	2	40	0.00057	1	Score > **20** indicates **identity**	U	L.LDSMHEVVENLL.N + Oxidation (M)
1007	226	–	236	643.3054	1284.5963	1284.6020	-0.0057	1	42	0.00031	1	Score > **19** indicates **identity**	U	L.DSMHEVVENLL.N
1036	226	–	236	651.3047	1300.5948	1300.5969	-0.0021	1	37	0.0017	1	Score > **21** indicates **identity**	U	L.DSMHEVVENLL.N + Oxidation (M)
1383	226	–	238	781.8555	1561.6965	1561.7082	-0.0118	2	55	1.3e-05	1	Score > **19** indicates **identity**	U	L.DSMHEVVENLLNY.C
1401	226	–	238	789.8554	1577.6963	1577.7032	-0.0069	2	51	2.4e-05	1	Score > **17** indicates **identity**	U	L.DSMHEVVENLLNY.C + Oxidation (M)
1375	244	–	256	518.5868	1552.7386	1552.7517	-0.0131	1	33	0.0021	1	Score > **19** indicates **identity**	U	F.LDKTMSIEFPEML.A
1390	244	–	256	523.9200	1568.7383	1568.7466	-0.0083	1	28	0.0059	1	Score > **18** indicates **identity**	U	F.LDKTMSIEFPEML.A + Oxidation (M)
1411	244	–	256	529.2502	1584.7288	1584.7415	-0.0127	1	26	0.017	1	Score > **20** indicates **identity**	U	F.LDKTMSIEFPEML.A + 2 Oxidation (M)
2352	244	–	267	942.1471	2823.4194	2823.4438	-0.0244	2	46	5.9e-05	1	Score > **16** indicates **identity**	U	F.LDKTMSIEFPEMLAEIITNQIPKY.S
2358	244	–	267	947.4760	2839.4062	2839.4387	-0.0325	2	34	0.0014	1	Score > **18** indicates **identity**	U	F.LDKTMSIEFPEMLAEIITNQIPKY.S + Oxidation (M)
2359	244	–	267	947.4764	2839.4073	2839.4387	-0.0314	2	37	0.00066	1	Score > **18** indicates **identity**	U	F.LDKTMSIEFPEMLAEIITNQIPKY.S + Oxidation (M)
2360	244	–	267	947.4771	2839.4094	2839.4387	-0.0293	2	56	9.4e-06	1	Score > **18** indicates **identity**	U	F.LDKTMSIEFPEMLAEIITNQIPKY.S + Oxidation (M)
2366	244	–	267	952.8061	2855.3965	2855.4336	-0.0371	2	60	2.5e-06	1	Score > **16** indicates **identity**	U	F.LDKTMSIEFPEMLAEIITNQIPKY.S + 2 Oxidation (M)
1246	245	–	256	728.8305	1455.6465	1455.6625	-0.0161	0	30	0.0057	1	Score > **20** indicates **identity**	U	L.DKTMSIEFPEML.A + Oxidation (M)
1018	257	–	267	645.3568	1288.6990	1288.7027	-0.0037	0	51	2.8e-05	1	Score > **18** indicates **identity**	U	L.AEIITNQIPKY.S
497	268	–	276	493.8031	985.5917	985.5920	-0.0003	1	45	6.6e-05	1	Score > **15** indicates **identity**	U	Y.SNGNIKKLL.F

MST2 chymotrypisin digest

Prot0560

Database: UserUKCM

Score: 1481

Monoisotopic mass (M_r): 32690

Calculated pI: 6.83

Sequence similarity is available as an NCBI BLAST search of Prot0560 against nr.

Search parameters

MS data file: D:\SCIEX OS Data\MGF files\May2018\300818 LBD GR MST2 chymo.mgf

Enzyme: Chymotrypsin: cuts C-term side of FLWY unless next residue is P.

Fixed modifications: Carbamidomethyl (C)

Variable modifications: Oxidation (M), Phospho (ST), Phospho (Y)

Protein sequence coverage: 82%

Matched peptides shown in ***bold red***.

1 MHHHHHHGGE NLYFQGNPGN KTIVPATLPQ LTPTLVSL**LE** **VIEPEVLYAG**

51 **YDSSVPDSTW** **RIMTTLNMLG** **GRQVIAAVKW** **AKAIPGFRNL** **HLDDQMTLLQ**

101 **YSWMSLMAFA** **LGWRSYRQSS** **ANLLCFAPDL** **IINEQRMTLP** **CMYDQCKHML**

151 **YVSSELHRLQ** **VSYEEYLCMK** **TLLLLSSVPK** **DGLKSQELFD** **EIRMTYIKEL**

201 **GKAIVKREGN** **SSQNWQRFY**Q L**TKLLDSMHE** **VVENLLNY**CF QTF**LDKTMSI**

251 **EFPEMLAEII** **TNQIPKYSNG** **NIKKLL**FHQK

Unformatted sequence string: 280 residues (for pasting into other applications).

Query	Start	-	End	Observed	Mr(expt)	Mr(calc)	Delta	M	Score	Expect	Rank	U	Peptide
578	39	-	47	520.7943	1039.5741	1039.5801	-0.0060	1	44	0.00021	1 Score > 20 indicates identity	U	L.LEVIEPEVL.Y
833	39	-	48	602.3261	1202.6376	1202.6434	-0.0059	2	69	6e-07	1 Score > 19 indicates identity	U	L.LEVIEPEVLY.A
376	40	-	47	464.2541	926.4936	926.4960	-0.0025	0	28	0.0055	1 Score > 18 indicates identity	U	L.EVIEPEVL.Y
666	40	-	48	545.7853	1089.5560	1089.5594	-0.0034	1	41	0.00036	1 Score > 19 indicates identity	U	L.EVIEPEVLY.A
1166	48	-	60	724.3004	1446.5863	1446.5939	-0.0077	2	23	0.021	1 Score > 19 indicates identity	U	L.YAGYDSSVPDSTW.R
948	49	-	60	642.7708	1283.5270	1283.5306	-0.0036	1	24	0.02	1 Score > 19 indicates identity	U	Y.AGYDSSVPDSTW.R
496	52	-	60	497.2104	992.4062	992.4087	-0.0025	0	27	0.0074	1 Score > 18 indicates identity	U	Y.DSSVPDSTW.R
692	61	-	69	554.7951	1107.5757	1107.5780	-0.0023	1	29	0.0051	1 Score > 18 indicates identity	U	W.RIMTTLNML.G + Oxidation (M)
798	61	-	69	586.7802	1171.5459	1171.5494	-0.0035	1	37	0.0009	1 Score > 19 indicates identity	U	W.RIMTTLNML.G + Phospho (ST)
816	61	-	69	594.7784	1187.5423	1187.5443	-0.0021	1	29	0.0081	1 Score > 20 indicates identity	U	W.RIMTTLNML.G + Oxidation (M); Phospho (ST)
817	61	-	69	594.7786	1187.5427	1187.5443	-0.0017	1	30	0.0047	1 Score > 20 indicates identity Score > 19 indicates homology	U	W.RIMTTLNML.G + Oxidation (M); Phospho (ST)
1879	61	-	80	593.3088	2369.2062	2369.2113	-0.0051	2	36	0.00094	1 Score > 18 indicates identity	U	W.RIMTTLNMLGGRQVIAAVKW.A + 2 Oxidation (M); Phospho (ST)
1943	61	-	80	817.3953	2449.1641	2449.1776	-0.0135	2	18	0.041	1 Score > 17 indicates identity	U	W.RIMTTLNMLGGRQVIAAVKW.A + 2 Oxidation (M); 2 Phospho (ST)
1266	67	-	80	514.9561	1541.8464	1541.8500	-0.0036	1	37	0.00057	1 Score > 17 indicates identity	U	L.NMLGGRQVIAAVKW.A
1282	67	-	80	520.2874	1557.8404	1557.8450	-0.0045	1	35	0.0012	1 Score > 20 indicates identity Score > 19 indicates homology	U	L.NMLGGRQVIAAVKW.A + Oxidation (M)
812	70	-	80	592.8490	1183.6835	1183.6826	0.0009	0	40	0.00039	1 Score > 19 indicates identity	U	L.GGRQVIAAVKW.A
65	81	-	87	352.2100	702.4055	702.4064	-0.0010	0	41	7.9e-05	1 Score > 13 indicates identity	U	W.AKAIPGF.R
655	81	-	90	543.8253	1085.6361	1085.6345	0.0016	1	30	0.004	1 Score > 19 indicates identity	U	W.AKAIPGFRNL.H
1056	88	-	98	452.5617	1354.6632	1354.6663	-0.0031	2	34	0.0029	1 Score > 21 indicates identity	U	F.RNLHLDDQMTL.L
1071	88	-	98	686.3402	1370.6658	1370.6612	0.0045	2	50	3e-05	1 Score > 20 indicates identity Score > 17 indicates homology	U	F.RNLHLDDQMTL.L + Oxidation (M)

Query	Start	– End	Observed	Mr(expt)	Mr(calc)	Delta	M	Score	Expect	Rank	U	Peptide
456	91	– 98	486.7253	971.4361	971.4382	-0.0021	1	27	0.0064	1 Score > **17** indicates **identity**	U	L.HLDDQMTL.L
490	91	– 98	494.7248	987.4350	987.4332	0.0018	1	44	0.00011	1 Score > **17** indicates **identity**	U	L.HLDDQMTL.L + Oxidation (M)
653	91	– 99	543.2654	1084.5162	1084.5223	-0.0061	2	39	0.00043	1 Score > **18** indicates **identity**	U	L.HLDDQMTLL.Q
682	91	– 99	551.2628	1100.5110	1100.5172	-0.0062	2	24	0.015	1 Score > **18** indicates **identity**	U	L.HLDDQMTLL.Q + Oxidation (M)
683	91	– 99	551.2650	1100.5154	1100.5172	-0.0018	2	37	0.0008	1 Score > **18** indicates **identity**	U	L.HLDDQMTLL.Q + Oxidation (M)
384	100	– 106	465.7031	929.3917	929.3953	-0.0036	2	35	0.0016	1 Score > **19** indicates **identity**	U	L.QYSWMSL.M + Oxidation (M)
489	102	– 109	494.7152	987.4158	987.4194	-0.0036	2	21	0.023	1 Score > **17** indicates **identity**	U	Y.SWMSLMAF.A + Oxidation (M)
514	102	– 109	502.7125	1003.4104	1003.4143	-0.0039	2	30	0.0038	1 Score > **18** indicates **identity**	U	Y.SWMSLMAF.A + 2 Oxidation (M)
146	107	– 113	398.1950	794.3755	794.3785	-0.0030	2	25	0.0032	1 Score > **13** indicates **identity**	U	L.MAFALGW.R
214	110	– 116	426.7213	851.4280	851.4290	-0.0010	2	36	0.00025	1 Score > **13** indicates **identity**	U	F.ALGWRSY.R
221	117	– 123	428.2603	854.5060	854.3647	0.1413	0	16	0.041	1 Score > **15** indicates **identity**	U	Y.RQSSANL.L + Phospho (ST)
288	117	– 124	444.7490	887.4835	887.4825	0.0010	1	46	6.1e-05	1 Score > **16** indicates **identity**	U	Y.RQSSANLL.C
289	117	– 124	444.7491	887.4836	887.4825	0.0012	1	38	0.0004	1 Score > **16** indicates **identity**	U	Y.RQSSANLL.C
442	117	– 124	484.7313	967.4481	967.4488	-0.0006	1	30	0.0024	1 Score > **16** indicates **identity**	U	Y.RQSSANLL.C + Phospho (ST)
443	117	– 124	484.7317	967.4489	967.4488	0.0001	1	26	0.0057	1 Score > **16** indicates **identity**	U	Y.RQSSANLL.C + Phospho (ST)
1898	125	– 143	796.6968	2387.0687	2387.0782	-0.0095	2	31	0.0012	1 Score > **21** indicates **identity** Score > **14** indicates **homology**	U	L.CFAPDLIINEQRMTLPCMY.D + Oxidation (M)
1910	125	– 143	802.0282	2403.0627	2403.0731	-0.0104	2	30	0.0031	1 Score > **21** indicates **identity** Score > **17** indicates **homology**	U	L.CFAPDLIINEQRMTLPCMY.D + 2 Oxidation (M)
1944	125	– 143	818.0201	2451.0385	2451.0496	-0.0111	2	25	0.0077	1 Score > **17** indicates **identity**	U	L.CFAPDLIINEQRMTLPCMY.D + Phospho (ST)
1965	125	– 143	828.6824	2483.0255	2483.0394	-0.0139	2	20	0.04	1 Score > **19** indicates **identity**	U	L.CFAPDLIINEQRMTLPCMY.D + 2 Oxidation (M); Phospho (ST)
1682	127	– 143	694.3307	2079.9704	2079.9791	-0.0088	1	31	0.0029	1 Score > **18** indicates **identity**	U	F.APDLIINEQRMTLPCMY.D + Oxidation (M)
672	144	– 151	547.7419	1093.4692	1093.4685	0.0007	1	20	0.02	1 Score > **16** indicates **identity**	U	Y.DQCKHMLY.V

Query	Start - End	Observed	Mr(expt)	Mr(calc)	Delta	M	Score	Expect	Rank		U	Peptide
697	144 - 151	555.7406	1109.4667	1109.4634	0.0033	1	34	0.0015	1	Score > 18 indicates identity	U	Y.DQCKHMLY.V + Oxidation (M)
698	144 - 151	555.7407	1109.4669	1109.4634	0.0035	1	33	0.0018	1	Score > 18 indicates identity	U	Y.DQCKHMLY.V + Oxidation (M)
405	152 - 159	470.7651	939.5156	939.5138	0.0018	1	48	5.8e-05	1	Score > 18 indicates identity	U	Y.VSSELHRL.Q
1131	152 - 163	473.2525	1416.7357	1416.7361	-0.0004	2	29	0.0061	1	Score > 19 indicates identity	U	Y.VSSELHRLQVSY.E
323	157 - 163	451.7465	901.4784	901.4770	0.0015	1	32	0.0014	1	Score > 16 indicates identity	U	L.HRLQVSY.E
1015	157 - 166	441.8820	1322.6242	1322.6255	-0.0013	2	26	0.0085	1	Score > 18 indicates identity	U	L.HRLQVSYEEY.L
267	167 - 173	439.7429	877.4713	877.4765	-0.0052	2	34	0.0012	1	Score > 17 indicates identity	U	Y.LCMKTLL.L
299	167 - 173	447.7431	893.4716	893.4714	0.0002	2	33	0.0015	1	Score > 17 indicates identity	U	Y.LCMKTLL.L + Oxidation (M)
556	174 - 183	514.8020	1027.5894	1027.5914	-0.0019	2	44	0.00013	1	Score > 18 indicates identity	U	L.LLSSVPKDGL.K
351	175 - 183	458.2606	914.5066	914.5073	-0.0007	1	19	0.042	1	Score > 18 indicates identity	U	L.LSSVPKDGL.K
1224	175 - 188	750.9153	1499.8161	1499.8195	-0.0034	2	22	0.013	1	Score > 20 indicates identity Score > 15 indicates homology	U	L.LSSVPKDGLKSQEL.F
1225	175 - 188	500.9475	1499.8207	1499.8195	0.0012	2	32	0.0016	1	Score > 20 indicates identity Score > 17 indicates homology	U	L.LSSVPKDGLKSQEL.F
152	176 - 183	401.7195	801.4245	801.4232	0.0013	0	33	0.00062	1	Score > 13 indicates identity	U	L.SSVPKDGL.K
1093	176 - 188	694.3754	1386.7363	1386.7354	0.0009	1	26	0.019	1	Score > 21 indicates identity	U	L.SSVPKDGLKSQEL.F
1258	176 - 189	512.2740	1533.8000	1533.8039	-0.0038	2	26	0.0091	1	Score > 19 indicates identity	U	L.SSVPKDGLKSQELF.D
1427	184 - 196	553.9383	1658.7930	1658.7974	-0.0044	2	44	0.00022	1	Score > 20 indicates identity Score > 20 indicates homology	U	L.KSQELFDEIRMTY.I
1450	184 - 196	559.2706	1674.7900	1674.7923	-0.0023	2	64	2.4e-06	1	Score > 21 indicates identity	U	L.KSQELFDEIRMTY.I + Oxidation (M)
639	189 - 196	537.7478	1073.4810	1073.4852	-0.0042	1	49	4.8e-05	1	Score > 18 indicates identity	U	L.FDEIRMTY.I
664	189 - 196	545.7467	1089.4789	1089.4801	-0.0012	1	44	0.00018	1	Score > 19 indicates identity	U	L.FDEIRMTY.I + Oxidation (M)
665	189 - 196	545.7479	1089.4813	1089.4801	0.0012	1	46	0.00011	1	Score > 19 indicates identity	U	L.FDEIRMTY.I + Oxidation (M)
375	190 - 196	464.2152	926.4159	926.4167	-0.0009	0	38	0.00054	1	Score > 18 indicates identity	U	F.DEIRMTY.I
410	190 - 196	472.2132	942.4119	942.4117	0.0002	0	37	0.00063	1	Score > 17 indicates identity	U	F.DEIRMTY.I + Oxidation (M)

Query	Start	–	End	Observed	Mr(expt)	Mr(calc)	Delta	M	Score	Expect	Rank	U	Peptide
1708	197	–	215	540.0500	2156.1709	2156.1702	0.0007	1	15	0.044	1 Score > 20 indicates identity Score > 14 indicates homology	U	Y.IKELGKAIVKREGNSSQNW.Q
1864	201	–	219	587.7883	2347.1241	2347.1222	0.0018	2	22	0.014	1 Score > 16 indicates identity	U	L.GKAIVKREGNSSQNWQRFY.Q + Phospho (ST)
1518	222	–	235	894.4368	1786.8591	1786.7613	0.0977	2	18	0.046	1 Score > 20 indicates identity Score > 17 indicates homology	U	L.TKLLDSMHEVVENL.L + 2 Phospho (ST)
1109	225	–	236	699.8478	1397.6811	1397.6860	-0.0049	2	67	8e-07	1 Score > 18 indicates identity	U	L.LDSMHEVVENLL.N
1128	225	–	236	707.8457	1413.6768	1413.6810	-0.0042	2	55	1.8e-05	1 Score > 20 indicates identity	U	L.LDSMHEVVENLL.N + Oxidation (M)
954	226	–	236	643.3039	1284.5932	1284.6020	-0.0088	1	43	0.00024	1 Score > 19 indicates identity	U	L.DSMHEVVENLL.N
985	226	–	236	651.3050	1300.5955	1300.5969	-0.0014	1	43	0.00037	1 Score > 21 indicates identity	U	L.DSMHEVVENLL.N + Oxidation (M)
1285	226	–	238	521.5738	1561.6996	1561.7082	-0.0087	2	47	7.7e-05	1 Score > 19 indicates identity	U	L.DSMHEVVENLLNY.C
1304	226	–	238	789.8564	1577.6982	1577.7032	-0.0050	2	55	9.6e-06	1 Score > 17 indicates identity	U	L.DSMHEVVENLLNY.C + Oxidation (M)
1276	244	–	256	777.3793	1552.7440	1552.7517	-0.0076	1	56	9.6e-06	1 Score > 19 indicates identity	U	F.LDKTMSIEFPEML.A
1290	244	–	256	523.9210	1568.7411	1568.7466	-0.0055	1	29	0.0049	1 Score > 18 indicates identity	U	F.LDKTMSIEFPEML.A + Oxidation (M)
1312	244	–	256	793.3736	1584.7327	1584.7415	-0.0088	1	41	0.00054	1 Score > 20 indicates identity	U	F.LDKTMSIEFPEML.A + 2 Oxidation (M)
1396	244	–	256	817.3614	1632.7082	1632.7180	-0.0098	1	26	0.012	1 Score > 20 indicates identity Score > 20 indicates homology	U	F.LDKTMSIEFPEML.A + Phospho (ST)
2072	244	–	267	942.1487	2823.4242	2823.4438	-0.0196	2	45	6.7e-05	1 Score > 16 indicates identity	U	F.LDKTMSIEFPEMLAEIITNQIPKY.S
2076	244	–	267	947.4790	2839.4150	2839.4387	-0.0237	2	22	0.024	1 Score > 18 indicates identity	U	F.LDKTMSIEFPEMLAEIITNQIPKY.S + Oxidation (M)
2077	244	–	267	947.4792	2839.4159	2839.4387	-0.0228	2	64	1.4e-06	1 Score > 18 indicates identity	U	F.LDKTMSIEFPEMLAEIITNQIPKY.S + Oxidation (M)
2081	244	–	267	952.8123	2855.4151	2855.4336	-0.0185	2	45	7.6e-05	1 Score > 16 indicates identity	U	F.LDKTMSIEFPEMLAEIITNQIPKY.S + 2 Oxidation (M)
1174	245	–	256	728.8354	1455.6563	1455.6625	-0.0063	0	26	0.014	1 Score > 20 indicates identity	U	L.DKTMSIEFPEML.A + Oxidation (M)
964	257	–	267	645.3572	1288.6998	1288.7027	-0.0029	0	52	2.4e-05	1 Score > 18 indicates identity	U	L.AEIITNQIPKY.S
483	268	–	276	493.8042	985.5938	985.5920	0.0018	1	36	0.00047	1 Score > 15 indicates identity	U	Y.SNGNIKKLL.F

Rock1 chymotrypisin digest

Protein View: Prot0560

Prot0560

**Database:**	UserUKCM
**Score:**	1294
**Monoisotopic mass (M_r):**	32690
**Calculated pI:**	6.83

Sequence similarity is available as an NCBI BLAST search of Prot0560 against nr.

Search parameters

**MS data file:**	D:\SCIEX OS Data\MGF files\May2018\300818 LBD GR Rock1 chymo.mgf
**Enzyme:**	Chymotrypsin: cuts C-term side of FLWY unless next residue is P.
**Fixed modifications:**	Carbamidomethyl (C)
**Variable modifications:**	Oxidation (M), Phospho (ST), Phospho (Y)

Protein sequence coverage: 76%

Matched peptides shown in ***bold red***.

```
  1 MHHHHHHGGE NLYFQGNPGN KTIVPATLPQ LTPTLVSLLE VIEPEVLYAG
 51 YDSSVPDSTW RIMTTLNMLG GRQVIAAVKW AKAIPGFRNL HLDDQMTLLQ
101 YSWMSLMAFA LGWRSYRQSS ANLLCFAPDL IINEQRMTLP CMYDQCKHML
151 YVSSELHRLQ VSYEEYLCMK TLLLLSSVPK DGLKSQELFD EIRMTYIKEL
```

201 GKAIVKREGN SSQNWQRFYQ LTKL**LDSMHE** **VVENLLNYCF** **QTFLDKTMSI**

251 **EFPEMLAEII** **TNQIPKYSNG** **NIKKLL**FHQK

Unformatted sequence string: 280 residues (for pasting into other applications).

Query	Start – End	Observed	Mr(expt)	Mr(calc)	Delta	M	Score	Expect	Rank		U	Peptide
967	36 – 47	670.3863	1338.7580	1338.7646	-0.0066	2	27	0.01	1	Score **>** **19** indicates **identity**	U	L.VSLLEVIEPEVL.Y
813	39 – 48	602.3275	1202.6405	1202.6434	-0.0030	2	40	0.00045	1	Score **>** **19** indicates **identity**	U	L.LEVIEPEVLY.A
367	40 – 47	464.2534	926.4923	926.4960	-0.0038	0	25	0.012	1	Score **>** **18** indicates **identity**	U	L.EVIEPEVL.Y
658	40 – 48	545.7864	1089.5582	1089.5594	-0.0012	1	43	0.00022	1	Score **>** **19** indicates **identity**	U	L.EVIEPEVLY.A
1077	48 – 60	724.3015	1446.5884	1446.5939	-0.0055	2	25	0.012	1	Score **>** **19** indicates **identity**	U	L.YAGYDSSVPDSTW.R
891	49 – 60	642.7719	1283.5293	1283.5306	-0.0013	1	25	0.017	1	Score **>** **19** indicates **identity**	U	Y.AGYDSSVPDSTW.R
497	52 – 60	497.2112	992.4078	992.4087	-0.0009	0	25	0.01	1	Score **>** **18** indicates **identity**	U	Y.DSSVPDSTW.R
685	61 – 69	554.7967	1107.5789	1107.5780	0.0009	1	36	0.00091	1	Score **>** **18** indicates **identity**	U	W.RIMTTLNML.G<br>+ Oxidation (M)
1160	67 – 80	514.9557	1541.8453	1541.8500	-0.0047	1	33	0.0013	1	Score **>** **17** indicates **identity**	U	L.NMLGGRQVIAAVKW.A
1170	67 – 80	520.2885	1557.8437	1557.8450	-0.0013	1	32	0.0018	1	Score **>** **20** indicates **identity**<br>Score **>** **17** indicates **homology**	U	L.NMLGGRQVIAAVKW.A<br>+ Oxidation (M)
1504	67 – 87	748.3640	2242.0702	2242.2408	-0.1707	2	24	0.012	1	Score **>** **20** indicates **identity**<br>Score **>** **17** indicates **homology**	U	L.NMLGGRQVIAAVKWAKAIPGF.R<br>+ Oxidation (M)
791	70 – 80	592.8490	1183.6835	1183.6826	0.0009	0	36	0.001	1	Score **>** **19** indicates **identity**	U	L.GGRQVIAAVKW.A
62	81 – 87	352.2097	702.4049	702.4064	-0.0015	0	42	5.7e-05	1	Score **>** **13** indicates **identity**	U	W.AKAIPGF.R
648	81 – 90	543.8247	1085.6349	1085.6345	0.0004	1	24	0.017	1	Score **>** **19** indicates **identity**	U	W.AKAIPGFRNL.H
985	88 – 98	452.5623	1354.6650	1354.6663	-0.0013	2	37	0.0013	1	Score **>** **21** indicates **identity**	U	F.RNLHLDDQMTL.L
454	91 – 98	486.7263	971.4380	971.4382	-0.0002	1	30	0.0032	1	Score **>** **17** indicates **identity**	U	L.HLDDQMTL.L
492	91 – 98	494.7242	987.4339	987.4332	0.0007	1	45	9.8e-05	1	Score **>** **17** indicates **identity**	U	L.HLDDQMTL.L<br>+ Oxidation (M)
647	91 – 99	543.2673	1084.5200	1084.5223	-0.0023	2	40	0.00035	1	Score **>** **18** indicates **identity**	U	L.HLDDQMTLL.Q
674	91 – 99	551.2656	1100.5167	1100.5172	-0.0005	2	32	0.0024	1	Score **>** **18** indicates **identity**	U	L.HLDDQMTLL.Q<br>+ Oxidation (M)
372	100 – 106	465.7045	929.3945	929.3953	-0.0008	2	21	0.035	1	Score **>** **19** indicates **identity**	U	L.QYSWMSL.M<br>+ Oxidation (M)
453	102 – 109	486.7177	971.4209	971.4245	-0.0036	2	23	0.015	1	Score **>** **17** indicates **identity**	U	Y.SWMSLMAF.A
491	102 – 109	494.7149	987.4152	987.4194	-0.0042	2	19	0.034	1	Score **>** **17** indicates **identity**	U	Y.SWMSLMAF.A<br>+ Oxidation (M)

Query	Start – End	Observed	Mr(expt)	Mr(calc)	Delta	M	Score	Expect	Rank	U	Peptide
516	102 – 109	502.7137	1003.4129	1003.4143	-0.0015	2	39	0.00049	1Score **> 18** indicates **identity**	U	Y.SWMSLMAF.A + 2 Oxidation (M)
145	107 – 113	398.1952	794.3758	794.3785	-0.0028	2	28	0.0016	1Score **> 13** indicates **identity**	U	L.MAFALGW.R
214	110 – 116	426.7221	851.4297	851.4290	0.0007	2	40	0.00011	1Score **> 13** indicates **identity**	U	F.ALGWRSY.R
283	117 – 124	444.7497	887.4848	887.4825	0.0023	1	43	0.00012	1Score **> 16** indicates **identity**	U	Y.RQSSANLL.C
284	117 – 124	444.7498	887.4851	887.4825	0.0026	1	39	0.0003	1Score **> 16** indicates **identity**	U	Y.RQSSANLL.C
444	117 – 124	484.7315	967.4485	967.4488	-0.0002	1	30	0.0026	1Score **> 16** indicates **identity**	U	Y.RQSSANLL.C + Phospho (ST)
1559	125 – 143	791.3660	2371.0762	2371.0833	-0.0071	2	25	0.0085	1Score **> 17** indicates **identity**	U	L.CFAPDLIINEQRMTLPCMY.D
1574	125 – 143	802.0310	2403.0711	2403.0731	-0.0020	2	32	0.0042	1Score **> 21** indicates **identity**	U	L.CFAPDLIINEQRMTLPCMY.D + 2 Oxidation (M)
1452	127 – 143	688.9983	2063.9729	2063.9842	-0.0113	1	22	0.024	1Score **> 18** indicates **identity**	U	F.APDLIINEQRMTLPCMY.D
1456	127 – 143	694.3323	2079.9750	2079.9791	-0.0041	1	21	0.029	1Score **> 18** indicates **identity**	U	F.APDLIINEQRMTLPCMY.D + Oxidation (M)
1461	127 – 143	699.6642	2095.9707	2095.9740	-0.0034	1	32	0.0023	1Score **> 20** indicates **identity** Score **> 18** indicates **homology**	U	F.APDLIINEQRMTLPCMY.D + 2 Oxidation (M)
664	144 – 151	547.7409	1093.4673	1093.4685	-0.0011	1	28	0.0032	1Score **> 16** indicates **identity**	U	Y.DQCKHMLY.V
688	144 – 151	555.7399	1109.4653	1109.4634	0.0019	1	28	0.0056	1Score **> 18** indicates **identity**	U	Y.DQCKHMLY.V + Oxidation (M)
399	152 – 159	470.7669	939.5193	939.5138	0.0056	1	39	0.00044	1Score **> 18** indicates **identity**	U	Y.VSSELHRL.Q
1048	152 – 163	473.2525	1416.7357	1416.7361	-0.0004	2	26	0.012	1Score **> 19** indicates **identity**	U	Y.VSSELHRLQVSY.E
310	157 – 163	451.7464	901.4782	901.4770	0.0012	1	31	0.002	1Score **> 16** indicates **identity**	U	L.HRLQVSY.E
948	157 – 166	662.3209	1322.6272	1322.6255	0.0017	2	49	5e-05	1Score **> 18** indicates **identity**	U	L.HRLQVSYEEY.L
347	160 – 166	459.2019	916.3893	916.3814	0.0079	1	19	0.046	1Score **> 18** indicates **identity**	U	L.QVSYEEY.L
264	167 – 173	439.7476	877.4807	877.4765	0.0041	2	36	0.00065	1Score **> 17** indicates **identity**	U	Y.LCMKTLL.L
291	167 – 173	447.7444	893.4743	893.4714	0.0029	2	40	0.00026	1Score **> 17** indicates **identity**	U	Y.LCMKTLL.L + Oxidation (M)
557	174 – 183	514.8025	1027.5905	1027.5914	-0.0008	2	56	8e-06	1Score **> 18** indicates **identity**	U	L.LLSSVPKDGL.K
343	175 – 183	458.2572	914.4998	914.5073	-0.0075	1	21	0.026	1Score **> 18** indicates **identity**	U	L.LSSVPKDGL.K
1121	175 – 188	750.9153	1499.8161	1499.8195	-0.0034	2	21	0.02	1Score **> 20** indicates **identity** Score **> 16** indicates **homology**	U	L.LSSVPKDGLKSQEL.F
153	176 – 183	401.7184	801.4223	801.4232	-0.0009	0	37	0.00027	1Score **> 13** indicates **identity**	U	L.SSVPKDGL.K
1275	184 – 196	553.9388	1658.7944	1658.7974	-0.0029	2	55	9e-06	1Score **> 20** indicates **identity** Score **> 17** indicates **homology**	U	L.KSQELFDEIRMTY.I
1291	184 – 196	559.2709	1674.7909	1674.7923	-0.0014	2	47	0.00012	1Score **> 21** indicates **identity** Score **> 20** indicates **homology**	U	L.KSQELFDEIRMTY.I + Oxidation (M)
631	189 – 196	537.7491	1073.4837	1073.4852	-0.0015	1	49	4.8e-05	1Score **> 18** indicates **identity**	U	L.FDEIRMTY.I

Query	Start	End	Observed	Mr(expt)	Mr(calc)	Delta	M	Score	Expect	Rank	U	Peptide
656	189	- 196	545.7457	1089.4769	1089.4801	-0.0032	1	44	0.00018	1 Score > 19 indicates identity	U	L.FDEIRMTY.I + Oxidation (M)
657	189	- 196	545.7482	1089.4818	1089.4801	0.0017	1	41	0.00034	1 Score > 19 indicates identity	U	L.FDEIRMTY.I + Oxidation (M)
366	190	- 196	464.2172	926.4199	926.4167	0.0032	0	34	0.0015	1 Score > 18 indicates identity	U	F.DEIRMTY.I
404	190	- 196	472.2136	942.4127	942.4117	0.0010	0	34	0.0013	1 Score > 17 indicates identity	U	F.DEIRMTY.I + Oxidation (M)
1028	225	- 236	699.8493	1397.6841	1397.6860	-0.0019	2	38	0.00059	1 Score > 18 indicates identity	U	L.LDSMHEVVENLL.N
1045	225	- 236	707.8472	1413.6799	1413.6810	-0.0011	2	57	1.1e-05	1 Score > 20 indicates identity	U	L.LDSMHEVVENLL.N + Oxidation (M)
898	226	- 236	643.3069	1284.5993	1284.6020	-0.0027	1	50	4.7e-05	1 Score > 19 indicates identity	U	L.DSMHEVVENLL.N
921	226	- 236	651.3061	1300.5976	1300.5969	0.0007	1	40	0.00073	1 Score > 21 indicates identity	U	L.DSMHEVVENLL.N + Oxidation (M)
1174	226	- 238	521.5757	1561.7053	1561.7082	-0.0029	2	47	9e-05	1 Score > 19 indicates identity	U	L.DSMHEVVENLLNY.C
1183	226	- 238	789.8584	1577.7023	1577.7032	-0.0009	2	59	4.1e-06	1 Score > 17 indicates identity	U	L.DSMHEVVENLLNY.C + Oxidation (M)
464	237	- 243	490.2022	978.3899	978.3906	-0.0007	2	24	0.014	1 Score > 18 indicates identity	U	L.NYCFQTF.L
1165	244	- 256	777.3820	1552.7494	1552.7517	-0.0023	1	49	4.7e-05	1 Score > 19 indicates identity	U	F.LDKTMSIEFPEML.A
1177	244	- 256	785.3793	1568.7440	1568.7466	-0.0026	1	31	0.0029	1 Score > 18 indicates identity	U	F.LDKTMSIEFPEML.A + Oxidation (M)
1191	244	- 256	529.2536	1584.7391	1584.7415	-0.0024	1	30	0.0059	1 Score > 20 indicates identity	U	F.LDKTMSIEFPEML.A + 2 Oxidation (M)
1670	244	- 267	942.1525	2823.4356	2823.4438	-0.0082	2	54	7.8e-06	1 Score > 16 indicates identity	U	F.LDKTMSIEFPEMLAEIITNQIPKY.S
1675	244	- 267	947.4847	2839.4324	2839.4387	-0.0063	2	58	5.6e-06	1 Score > 18 indicates identity	U	F.LDKTMSIEFPEMLAEIITNQIPKY.S + Oxidation (M)
1678	244	- 267	952.8147	2855.4222	2855.4336	-0.0115	2	62	1.5e-06	1 Score > 17 indicates identity	U	F.LDKTMSIEFPEMLAEIITNQIPKY.S + 2 Oxidation (M)
1083	245	- 256	728.8337	1455.6528	1455.6625	-0.0098	0	27	0.011	1 Score > 20 indicates identity	U	L.DKTMSIEFPEML.A + Oxidation (M)
905	257	- 267	645.3601	1288.7056	1288.7027	0.0029	0	62	2.3e-06	1 Score > 18 indicates identity	U	L.AEIITNQIPKY.S
484	268	- 276	493.8045	985.5944	985.5920	0.0024	1	40	0.00018	1 Score > 15 indicates identity	U	Y.SNGNIKKLL.F

2.2.2. Chymotrypsin digestion and data analysis using the SwissProtdata base.

MAP4K4 chymotrypsin digest

Glucocorticoid receptor OS=Homo sapiens OX=9606 GN=NR3C1 PE=1 SV=1

**Database:**	SwissProt
**Score:**	555
**Monoisotopic mass (M_r):**	86745
**Calculated pI:**	6.00
**Taxonomy:**	Homo sapiens

Sequence similarity is available as an NCBI BLAST search of GCR_HUMAN against nr.

Search parameters

**MS data file:**	D:\SCIEX OS Data\MGF files\May2018\300818 LBD GR MAP4K4 chymo.mgf
**Enzyme:**	Chymotrypsin: cuts C-term side of FLWY unless next residue is P.
**Fixed modifications:**	Carbamidomethyl (C)
**Variable modifications:**	Oxidation (M), Phospho (ST), Phospho (Y)

Protein sequence coverage: 23%

Matched peptides shown in ***bold red***.

```
  1 MDSKESLTPG REENPSSVLA QERGDVMDFY KTLRGGATVK VSASSPSLAV
 51 ASQSDSKQRR LLVDFPKGSV SNAQQPDLSK AVSLSMGLYM GETETKVMGN
101 DLGFPQQGQI SLSSGETDLK LLEESIANLN RSTSVPENPK SSASTAVSAA
151 PTEKEFPKTH SDVSSEQQHL KGQTGTNGGN VKLYTTDQST FDILQDLEFS
201 SGSPGKETNE SPWRSDLLID ENCLLSPLAG EDDSFLLEGN SNEDCKPLIL
251 PDTKPKIKDN GDLVLSSPSN VTLPQVKTEK EDFIELCTPG VIKQEKLGTV
301 YCQASFPGAN IIGNKMSAIS VHGVSTSGGQ MYHYDMNTAS LSQQQDQKPI
```

351 FNVIPPIPVG SENWNRCQGS GDDNLTSLGT LNFPGRTVFS NGYSSPSMRP

401 DVSSPPSSSS TATTGPPPKL CLVCSDEASG CHYGVLTCGS CKVFFKRAVE

451 GQHNYLCAGR NDCIIDKIRR KNCPACRYRK CLQAGMNLEA RKTKKKIKGI

501 QQATTGVSQE TSENPGNKTI VPATLPQLTP TLVSL**LEVIE** **PEVLYAGYDS**

551 **SVPDSTWRIM** **TTLNMLGGRQ** **VIAAVKWAKA** **IPGFRNLHLD** **DQMTLL**QYSW

601 MFL**MAFALGW** RSY**RQSSANL** **LCFAPDLIIN** **EQRMTLPCMY** DQCKHMLY**VS**

651 **SELHRLQVSY** **EEY**LCMKTLL **LLSSVPKDGL** **KSQELFDEIR** **MTY**IKELGKA

701 IVKREGNSSQ NWQRFYQLTK L**LDSMHEVVE** **NLLNY**CFQTF **LDKTMSIEFP**

751 **EMLAEIITNQ** **IPKYSNGNIK** **KLL**FHQK

Unformatted sequence string: 777 residues (for pasting into other applications).

Query	Start	–	End	Observed	Mr (expt)	Mr (calc)	Delta	M	Score	Expect	Rank	U	Peptide
725	536	–	544	520.7974	1039.5803	1039.5801	0.0002	1	28	0.68	1Score **>** **38** indicates **identity** Score **>** **27** indicates **homology**		L.LEVIEPEVL.Y
1029	536	–	545	602.3270	1202.6394	1202.6434	-0.0041	2	55	0.016	1Score **>** **39** indicates **identity** Score **>** **37** indicates **homology**		L.LEVIEPEVLY.A
469	537	–	544	464.2548	926.4951	926.4960	-0.0010	0	41	0.44	2Score **>** **37** indicates **identity**		L.EVIEPEVL.Y
816	537	–	545	545.7841	1089.5537	1089.5594	-0.0057	1	40	0.66	4Score **>** **38** indicates **identity**		L.EVIEPEVLY.A
1434	545	–	557	724.3022	1446.5899	1446.5939	-0.0040	2	34	0.043	1Score **>** **39** indicates **identity** Score **>** **20** indicates **homology**	U	L.YAGYDSSVPDSTW.R
1162	546	–	557	642.7706	1283.5267	1283.5306	-0.0040	1	23	0.41	1Score **>** **39** indicates **identity** Score **>** **20** indicates **homology**	U	Y.AGYDSSVPDSTW.R
617	549	–	557	497.2109	992.4072	992.4087	-0.0015	0	24	0.17	1Score **>** **38** indicates **identity** Score **>** **16** indicates **homology**	U	Y.DSSVPDSTW.R
853	558	–	566	554.7970	1107.5795	1107.5780	0.0015	1	31	0.29	1Score **>** **38** indicates **identity** Score **>** **26** indicates **homology**	U	W.RIMTTLNML.G + Oxidation (M)
980	558	–	566	586.7776	1171.5406	1171.5494	-0.0088	1	37	0.35	1Score **>** **38** indicates **identity** Score **>** **32** indicates **homology**	U	W.RIMTTLNML.G + Phospho (ST)
1566	564	–	577	514.9572	1541.8497	1541.8500	-0.0003	1	32	0.33	1Score **>** **39** indicates **identity** Score **>** **27** indicates **homology**	U	L.NMLGGRQVIAAVKW.A
1587	564	–	577	520.2853	1557.8341	1557.8450	-0.0109	1	37	0.015	1Score **>** **39** indicates **identity** Score **>** **19** indicates **homology**	U	L.NMLGGRQVIAAVKW.A + Oxidation (M)
997	567	–	577	592.8490	1183.6835	1183.6826	0.0009	0	48	0.11	1Score **>** **38** indicates **identity**	U	L.GGRQVIAAVKW.A
79	578	–	584	352.2079	702.4013	702.4064	-0.0051	0	41	0.053	1Score **>** **28** indicates **identity**		W.AKAIPGF.R
1284	585	–	595	452.5619	1354.6637	1354.6663	-0.0026	2	37	0.86	1Score **>** **39** indicates **identity** Score **>** **37** indicates **homology**		F.RNLHLDDQMTL.L
1305	585	–	595	686.3396	1370.6647	1370.6612	0.0034	2	29	0.54	1Score **>** **39** indicates **identity** Score **>** **26** indicates **homology**		F.RNLHLDDQMTL.L

+ Oxidation (M)

Query	Start	– End	Observed	Mr(expt)	Mr(calc)	Delta	M	Score	Expect	Rank	U	Peptide
568	588	– 595	486.7257	971.4369	971.4382	-0.0013	1	37	0.33	1Score > 38 indicates **identity** Score > 32 indicates **homology**		L.HLDDQMTL.L
610	588	– 595	494.7244	987.4343	987.4332	0.0011	1	40	0.4	1Score > 38 indicates **identity** Score > 36 indicates **homology**		L.HLDDQMTL.L + Oxidation (M)
802	588	– 596	543.2661	1084.5176	1084.5223	-0.0047	2	41	0.15	1Score > 38 indicates **identity** Score > 33 indicates **homology**		L.HLDDQMTLL.Q
842	588	– 596	551.2642	1100.5139	1100.5172	-0.0033	2	44	0.28	6Score > 38 indicates **identity**		L.HLDDQMTLL.Q + Oxidation (M)
176	604	– 610	398.1952	794.3759	794.3785	-0.0026	2	42	0.078	1Score > 33 indicates **identity** Score > 31 indicates **homology**	U	L.MAFALGW.R
202	604	– 610	406.1936	810.3727	810.3734	-0.0007	2	37	0.53	1Score > 34 indicates **identity** Score > 34 indicates **homology**	U	L.MAFALGW.R + Oxidation (M)
354	614	– 621	444.7491	887.4836	887.4825	0.0012	1	43	0.28	8Score > 37 indicates **identity**	U	Y.RQSSANLL.C
355	614	– 621	444.7496	887.4846	887.4825	0.0021	1	45	0.16	5Score > 37 indicates **identity**	U	Y.RQSSANLL.C
2449	622	– 640	802.0296	2403.0668	2403.0731	-0.0063	2	43	0.0041	1Score > 38 indicates **identity** Score > 19 indicates **homology**	U	L.CFAPDLIINEQRMTLPCMY.D + 2 Oxidation (M)
2493	622	– 640	818.0202	2451.0388	2451.0496	-0.0108	2	28	0.87	1Score > 38 indicates **identity** Score > 27 indicates **homology**	U	L.CFAPDLIINEQRMTLPCMY.D + Phospho (ST)
2170	624	– 640	694.3316	2079.9729	2079.9791	-0.0062	1	34	0.16	1Score > 38 indicates **identity** Score > 26 indicates **homology**	U	F.APDLIINEQRMTLPCMY.D + Oxidation (M)
503	649	– 656	470.7654	939.5162	939.5138	0.0024	1	52	0.017	1Score > 37 indicates **identity** Score > 34 indicates **homology**	U	Y.VSSELHRL.Q
1242	654	– 663	662.3187	1322.6228	1322.6255	-0.0027	2	55	0.0039	1Score > 39 indicates **identity** Score > 31 indicates **homology**	U	L.HRLQVSYEEY.L
694	671	– 680	514.8031	1027.5916	1027.5914	0.0003	2	48	0.04	1Score > 38 indicates **identity** Score > 34 indicates **homology**	U	L.LLSSVPKDGL.K
1339	673	– 685	694.3750	1386.7354	1386.7354	0.0000	1	32	0.56	1Score > 39 indicates **identity** Score > 30 indicates **homology**	U	L.SSVPKDGLKSQEL.F
1768	681	– 693	830.4025	1658.7904	1658.7974	-0.0069	2	51	0.0045	1Score > 39 indicates **identity** Score > 28 indicates **homology**	U	L.KSQELFDEIRMTY.I
1794	681	– 693	559.2715	1674.7927	1674.7923	0.0004	2	40	0.042	1Score > 39 indicates **identity** Score > 26 indicates **homology**	U	L.KSQELFDEIRMTY.I + Oxidation (M)
785	686	– 693	537.7481	1073.4817	1073.4852	-0.0035	1	52	0.012	1Score > 38 indicates **identity** Score > 33 indicates **homology**	U	L.FDEIRMTY.I
814	686	– 693	545.7476	1089.4807	1089.4801	0.0006	1	44	0.061	1Score > 38 indicates **identity** Score > 31 indicates **homology**	U	L.FDEIRMTY.I + Oxidation (M)
468	687	– 693	464.2156	926.4167	926.4167	0.0000	0	38	0.25	1Score > 37 indicates **identity** Score > 32 indicates **homology**	U	F.DEIRMTY.I
509	687	– 693	472.2127	942.4108	942.4117	-0.0009	0	37	0.32	1Score > 37 indicates **identity** Score > 32 indicates **homology**	U	F.DEIRMTY.I + Oxidation (M)
1356	722	– 733	699.8494	1397.6842	1397.6860	-0.0018	2	54	0.027	1Score > 39 indicates **identity**	U	L.LDSMHEVVENLL.N
1381	722	– 733	707.8458	1413.6770	1413.6810	-0.0039	2	43	0.33	2Score > 39 indicates **identity** Score > 38 indicates **homology**	U	L.LDSMHEVVENLL.N + Oxidation (M)

Query	Start – End	Observed	Mr(expt)	Mr(calc)	Delta	M	Score	Expect	Rank	U	Peptide
1167	723 – 733	643.3053	1284.5960	1284.6020	-0.0060	1	52	0.048	1 Score > 39 indicates identity	U	L.DSMHEVVENLL.N
1592	723 – 735	781.8585	1561.7024	1561.7082	-0.0058	2	51	0.015	1 Score > 39 indicates identity<br>Score > 33 indicates homology	U	L.DSMHEVVENLLNY.C
1612	723 – 735	789.8539	1577.6932	1577.7032	-0.0100	2	40	0.23	1 Score > 39 indicates identity<br>Score > 34 indicates homology	U	L.DSMHEVVENLLNY.C<br>+ Oxidation (M)
1581	741 – 753	777.3794	1552.7442	1552.7517	-0.0075	1	33	0.89	1 Score > 39 indicates identity<br>Score > 33 indicates homology	U	F.LDKTMSIEFPEML.A
1600	741 – 753	785.3778	1568.7410	1568.7466	-0.0056	1	35	0.23	1 Score > 39 indicates identity<br>Score > 29 indicates homology	U	F.LDKTMSIEFPEML.A<br>+ Oxidation (M)
2681	741 – 764	942.1494	2823.4263	2823.4438	-0.0175	2	54	0.0057	1 Score > 37 indicates identity<br>Score > 31 indicates homology	U	F.LDKTMSIEFPEMLAEIITNQIPKY.S
2692	741 – 764	947.4801	2839.4186	2839.4387	-0.0201	2	55	0.0047	1 Score > 37 indicates identity<br>Score > 32 indicates homology	U	F.LDKTMSIEFPEMLAEIITNQIPKY.S<br>+ Oxidation (M)
2693	741 – 764	947.4803	2839.4192	2839.4387	-0.0195	2	33	0.36	1 Score > 37 indicates identity<br>Score > 29 indicates homology	U	F.LDKTMSIEFPEMLAEIITNQIPKY.S<br>+ Oxidation (M)
2698	741 – 764	952.8115	2855.4125	2855.4336	-0.0211	2	58	0.0012	1 Score > 37 indicates identity<br>Score > 28 indicates homology	U	F.LDKTMSIEFPEMLAEIITNQIPKY.S<br>+ 2 Oxidation (M)
1176	754 – 764	645.3560	1288.6975	1288.7027	-0.0051	0	66	0.0003	1 Score > 39 indicates identity<br>Score > 31 indicates homology	U	L.AEIITNQIPKY.S
602	765 – 773	493.8038	985.5930	985.5920	0.0010	1	54	0.025	2 Score > 38 indicates identity	U	Y.SNGNIKKLL.F

MINK1 chymotrypsin digest

Glucocorticoid receptor OS=Homo sapiens OX=9606 GN=NR3C1 PE=1 SV=1

**Database:**	SwissProt
**Score:**	623
**Monoisotopic mass (M_r):**	86745
**Calculated pI:**	6.00
**Taxonomy:**	Homo sapiens

Sequence similarity is available as an NCBI BLAST search of GCR_HUMAN against nr.

Search parameters

**MS data file:**	D:\SCIEX OS Data\MGF files\May2018\300818 LBD GR MINK1 chymo.mgf
**Enzyme:**	Chymotrypsin: cuts C-term side of FLWY unless next residue is P.
**Fixed modifications:**	Carbamidomethyl (C)
**Variable modifications:**	Oxidation (M), Phospho (ST), Phospho (Y)

Protein sequence coverage: 25%

Matched peptides shown in ***bold red***.

```
  1 MDSKESLTPG REENPSSVLA QERGDVMDFY KTLRGGATVK VSASSPSLAV
 51 ASQSDSKQRR LLVDFPKGSV SNAQQPDLSK AVSLSMGLYM GETETKVMGN
101 DLGFPQQGQI SLSSGETDLK LLEESIANLN RSTSVPENPK SSASTAVSAA
151 PTEKEFPKTH SDVSSEQQHL KGQTGTNGGN VKLYTTDQST FDILQDLEFS
201 SGSPGKETNE SPWRSDLLID ENCLLSPLAG EDDSFLLEGN SNEDCKPLIL
251 PDTKPKIKDN GDLVLSSPSN VTLPQVKTEK EDFIELCTPG VIKQEKLGTV
301 YCQASFPGAN IIGNKMSAIS VHGVSTSGGQ MYHYDMNTAS LSQQQDQKPI
351 FNVIPPIPVG SENWNRCQGS GDDNLTSLGT LNFPGRTVFS NGYSSPSMRP
401 DVSSPPSSSS TATTGPPPKL CLVCSDEASG CHYGVLTCGS CKVFFKRAVE
451 GQHNYLCAGR NDCIIDKIRR KNCPACRYRK CLQAGMNLEA RKTKKKIKGI
501 QQATTGVSQE TSENPGNKTI VPATLPQLTP TLVSLLEVIE PEVLYAGYDS
551 SVPDSTWRIM TTLNMLGGRQ VIAAVKWAKA IPGFRNLHLD DQMTLLQYSW
601 MFLMAFALGW RSYRQSSANL LCFAPDLIIN EQRMTLPCMY DQCKHMLYVS
651 SELHRLQVSY EEYLCMKTLL LLSSVPKDGL KSQELFDEIR MTYIKELGKA
701 IVKREGNSSQ NWQRFYQLTK LLDSMHEVVE NLLNYCFQTF LDKTMSIEFP
751 EMLAEIITNQ IPKYSNGNIK KLLFHQK
```

Unformatted sequence string: 777 residues (for pasting into other applications).

Query	Start	– End	Observed	Mr(expt)	Mr(calc)	Delta	M	Score	Expect	Rank	U	Peptide
917	536	– 545	602.3267	1202.6388	1202.6434	-0.0046	2	67	0.0014	1Score **>** **38** indicates **identity**		L.LEVIEPEVLY.A
734	537	– 545	545.7848	1089.5551	1089.5594	-0.0042	1	41	0.5	2Score **>** **38** indicates **identity**<br>Score **>** **37** indicates **homology**		L.EVIEPEVLY.A
1299	545	– 557	724.2996	1446.5847	1446.5939	-0.0092	2	29	0.034	1Score **>** **38** indicates **identity**<br>Score **>** **14** indicates **homology**	U	L.YAGYDSSVPDSTW.R
1047	546	– 557	642.7665	1283.5185	1283.5306	-0.0121	1	19	0.59	1Score **>** **38** indicates **identity**<br>Score **>** **16** indicates **homology**	U	Y.AGYDSSVPDSTW.R
1048	546	– 557	642.7698	1283.5250	1283.5306	-0.0056	1	15	0.81	1Score **>** **38** indicates **identity**<br>Score **>** **13** indicates **homology**	U	Y.AGYDSSVPDSTW.R
544	549	– 557	497.2101	992.4056	992.4087	-0.0031	0	23	0.23	1Score **>** **37** indicates **identity**<br>Score **>** **16** indicates **homology**	U	Y.DSSVPDSTW.R
545	549	– 557	497.2104	992.4062	992.4087	-0.0025	0	24	0.87	1Score **>** **37** indicates **identity**<br>Score **>** **23** indicates **homology**	U	Y.DSSVPDSTW.R
875	558	– 566	586.7799	1171.5452	1171.5494	-0.0043	1	43	0.044	1Score **>** **38** indicates **identity**<br>Score **>** **29** indicates **homology**		W.RIMTTLNML.G<br>+ Phospho (ST)
893	558	– 566	594.7775	1187.5404	1187.5443	-0.0040	1	38	0.15	1Score **>** **38** indicates **identity**<br>Score **>** **29** indicates **homology**		W.RIMTTLNML.G<br>+ Oxidation (M); Phospho (ST)
2123	558	– 577	593.3087	2369.2055	2369.2113	-0.0058	2	23	0.85	1Score **>** **37** indicates **identity**<br>Score **>** **22** indicates **homology**		W.RIMTTLNMLGGRQVIAAVKW.A<br>+ 2 Oxidation (M); Phospho (ST)
1409	564	– 577	514.9528	1541.8367	1541.8500	-0.0133	1	32	0.16	1Score **>** **38** indicates **identity**<br>Score **>** **24** indicates **homology**		L.NMLGGRQVIAAVKW.A
1427	564	– 577	520.2852	1557.8337	1557.8450	-0.0112	1	41	0.023	1Score **>** **38** indicates **identity**<br>Score **>** **24** indicates **homology**		L.NMLGGRQVIAAVKW.A<br>+ Oxidation (M)
890	567	– 577	395.5679	1183.6820	1183.6826	-0.0006	0	50	0.068	1Score **>** **38** indicates **identity**		L.GGRQVIAAVKW.A
73	578	– 584	352.2074	702.4002	702.4064	-0.0063	0	41	0.054	1Score **>** **28** indicates **identity**		W.AKAIPGF.R
724	578	– 587	362.8849	1085.6328	1085.6345	-0.0017	1	32	0.27	2Score **>** **38** indicates **identity**<br>Score **>** **25** indicates **homology**		W.AKAIPGFRNL.H
1158	585	– 595	452.5599	1354.6579	1354.6663	-0.0084	2	42	0.15	1Score **>** **38** indicates **identity**<br>Score **>** **33** indicates **homology**		F.RNLHLDDQMTL.L
1180	585	– 595	686.3392	1370.6639	1370.6612	0.0027	2	58	0.0016	1Score **>** **38** indicates **identity**<br>Score **>** **29** indicates **homology**		F.RNLHLDDQMTL.L<br>+ Oxidation (M)
540	588	– 595	494.7233	987.4320	987.4332	-0.0012	1	45	0.12	1Score **>** **38** indicates **identity**<br>Score **>** **35** indicates **homology**		L.HLDDQMTL.L<br>+ Oxidation (M)
721	588	– 596	543.2656	1084.5166	1084.5223	-0.0057	2	42	0.4	1Score **>** **38** indicates **identity**		L.HLDDQMTLL.Q
162	604	– 610	398.1946	794.3747	794.3785	-0.0038	2	30	0.19	1Score **>** **33** indicates **identity**<br>Score **>** **23** indicates **homology**		L.MAFALGW.R
185	604	– 610	406.1914	810.3683	810.3734	-0.0051	2	42	0.1	1Score **>** **34** indicates **identity**<br>Score **>** **32** indicates **homology**		L.MAFALGW.R<br>+ Oxidation (M)
247	607	– 613	426.7216	851.4286	851.4290	-0.0004	2	34	0.29	1Score **>** **35** indicates **identity**<br>Score **>** **28** indicates **homology**		F.ALGWRSY.R
321	614	– 621	444.7483	887.4821	887.4825	-0.0004	1	50	0.05	1Score **>** **37** indicates **identity**	U	Y.RQSSANLL.C
2147	622	– 640	802.0271	2403.0594	2403.0731	-0.0137	2	35	0.056	1Score **>** **37** indicates **identity**<br>Score **>** **22** indicates **homology**	U	L.CFAPDLIINEQRMTLPCMY.D<br>+ 2 Oxidation (M)

Query	Start – End	Observed	Mr(expt)	Mr(calc)	Delta	M	Score	Expect	Rank	U	Peptide
2178	622 – 640	818.0198	2451.0374	2451.0496	-0.0122	2	29	0.16	1Score > 37 indicates **identity** Score > 20 indicates **homology**	U	L.CFAPDLIINEQRMTLPCMY.D + Phospho (ST)
2200	622 – 640	828.6825	2483.0258	2483.0394	-0.0137	2	39	0.078	1Score > 37 indicates **identity** Score > 28 indicates **homology**	U	L.CFAPDLIINEQRMTLPCMY.D + 2 Oxidation (M); Phospho (ST)
1932	624 – 640	694.3312	2079.9717	2079.9791	-0.0074	1	40	0.057	1Score > 38 indicates **identity** Score > 27 indicates **homology**	U	F.APDLIINEQRMTLPCMY.D + Oxidation (M)
444	649 – 656	470.7651	939.5156	939.5138	0.0018	1	61	0.0028	1Score > 37 indicates **identity** Score > 35 indicates **homology**	U	Y.VSSELHRL.Q
349	654 – 660	451.7463	901.4780	901.4770	0.0010	1	37	0.19	1Score > 37 indicates **identity** Score > 29 indicates **homology**	U	L.HRLQVSY.E
1115	654 – 663	662.3187	1322.6229	1322.6255	-0.0026	2	61	0.0014	1Score > 38 indicates **identity** Score > 32 indicates **homology**	U	L.HRLQVSYEEY.L
326	664 – 670	447.7430	893.4715	893.4714	0.0000	2	43	0.073	1Score > 36 indicates **identity** Score > 31 indicates **homology**		Y.LCMKTLL.L + Oxidation (M)
616	671 – 680	514.8026	1027.5907	1027.5914	-0.0007	2	39	0.89	2Score > 38 indicates **identity**	U	L.LLSSVPKDGL.K
1365	672 – 685	750.9111	1499.8077	1499.8195	-0.0118	2	35	0.037	1Score > 38 indicates **identity** Score > 20 indicates **homology**	U	L.LSSVPKDGLKSQEL.F
1622	681 – 693	559.2692	1674.7856	1674.7923	-0.0067	2	44	0.02	1Score > 38 indicates **identity** Score > 27 indicates **homology**		L.KSQELFDEIRMTY.I + Oxidation (M)
705	686 – 693	537.7474	1073.4802	1073.4852	-0.0050	1	52	0.012	1Score > 38 indicates **identity** Score > 32 indicates **homology**		L.FDEIRMTY.I
733	686 – 693	545.7470	1089.4795	1089.4801	-0.0006	1	44	0.061	1Score > 38 indicates **identity** Score > 31 indicates **homology**		L.FDEIRMTY.I + Oxidation (M)
409	687 – 693	464.2121	926.4097	926.4167	-0.0070	0	33	0.75	1Score > 37 indicates **identity** Score > 31 indicates **homology**		F.DEIRMTY.I
450	687 – 693	472.2129	942.4112	942.4117	-0.0005	0	35	0.085	1Score > 37 indicates **identity** Score > 24 indicates **homology**		F.DEIRMTY.I + Oxidation (M)
1959	694 – 712	540.0498	2156.1701	2156.1702	-0.0001	1	31	0.19	1Score > 38 indicates **identity** Score > 23 indicates **homology**		Y.IKELGKAIVKREGNSSQNW.Q
1225	722 – 733	699.8463	1397.6781	1397.6860	-0.0080	2	55	0.021	1Score > 38 indicates **identity**	U	L.LDSMHEVVENLL.N
1252	722 – 733	707.8464	1413.6783	1413.6810	-0.0027	2	40	0.7	2Score > 38 indicates **identity**	U	L.LDSMHEVVENLL.N + Oxidation (M)
1054	723 – 733	643.3051	1284.5957	1284.6020	-0.0063	1	42	0.51	5Score > 38 indicates **identity**	U	L.DSMHEVVENLL.N
1085	723 – 733	651.3050	1300.5955	1300.5969	-0.0014	1	44	0.24	1Score > 38 indicates **identity** Score > 37 indicates **homology**	U	L.DSMHEVVENLL.N + Oxidation (M)
1430	723 – 735	781.8576	1561.7006	1561.7082	-0.0076	2	53	0.0057	1Score > 38 indicates **identity** Score > 30 indicates **homology**	U	L.DSMHEVVENLLNY.C
1447	723 – 735	789.8570	1577.6995	1577.7032	-0.0036	2	45	0.038	1Score > 38 indicates **identity** Score > 31 indicates **homology**	U	L.DSMHEVVENLLNY.C + Oxidation (M)
1434	741 – 753	785.3771	1568.7397	1568.7466	-0.0069	1	41	0.084	1Score > 38 indicates **identity** Score > 29 indicates **homology**	U	F.LDKTMSIEFPEML.A + Oxidation (M)
1456	741 – 753	793.3745	1584.7344	1584.7415	-0.0071	1	29	0.1	1Score > 38 indicates **identity** Score > 19 indicates **homology**	U	F.LDKTMSIEFPEML.A + 2 Oxidation (M)

Query	Start – End	Observed	Mr(expt)	Mr(calc)	Delta	M	Score	Expect	Rank	U	Peptide
2350	741 – 764	947.4780	2839.4121	2839.4387	-0.0266	2	60	0.0016	1 Score **> 36** indicates **identity** Score **> 32** indicates **homology**	U	F.LDKTMSIEFPEMLAEIITNQIPKY.S + Oxidation (M)
2354	741 – 764	952.8113	2855.4121	2855.4336	-0.0215	2	37	0.11	1 Score **> 36** indicates **identity** Score **> 27** indicates **homology**	U	F.LDKTMSIEFPEMLAEIITNQIPKY.S + 2 Oxidation (M)
1308	742 – 753	728.8318	1455.6491	1455.6625	-0.0135	0	31	0.56	1 Score **> 38** indicates **identity** Score **> 28** indicates **homology**	U	L.DKTMSIEFPEML.A + Oxidation (M)
1064	754 – 764	645.3538	1288.6931	1288.7027	-0.0096	0	63	0.0034	1 Score **> 38** indicates **identity** Score **> 38** indicates **homology**	U	L.AEIITNQIPKY.S

MST1 chymotrypsin digest

Glucocorticoid receptor OS=Homo sapiens OX=9606 GN=NR3C1 PE=1 SV=1

**Database:**	SwissProt
**Score:**	601
**Monoisotopic mass (M_r):**	86745
**Calculated pI:**	6.00
**Taxonomy:**	Homo sapiens

Sequence similarity is available as an NCBI BLAST search of GCR_HUMAN against nr.

Search parameters

**MS data file:**	D:\SCIEX OS Data\MGF files\May2018\300818 LBD GR MST1 chymo.mgf
**Enzyme:**	Chymotrypsin: cuts C-term side of FLWY unless next residue is P.
**Fixed modifications:**	Carbamidomethyl (C)
**Variable modifications:**	Oxidation (M), Phospho (ST), Phospho (Y)

Protein sequence coverage: 24%

Matched peptides shown in ***bold red***.

1 MDSKESLTPG REENPSSVLA QERGDVMDFY KTLRGGATVK VSASSPSLAV

51 ASQSDSKQRR LLVDFPKGSV SNAQQPDLSK AVSLSMGLYM GETETKVMGN

101 DLGFPQQGQI SLSSGETDLK LLEESIANLN RSTSVPENPK SSASTAVSAA

151 PTEKEFPKTH SDVSSEQQHL KGQTGTNGGN VKLYTTDQST FDILQDLEFS

201 SGSPGKETNE SPWRSDLLID ENCLLSPLAG EDDSFLLEGN SNEDCKPLIL

251 PDTKPKIKDN GDLVLSSPSN VTLPQVKTEK EDFIELCTPG VIKQEKLGTV

301 YCQASFPGAN IIGNKMSAIS VHGVSTSGGQ MYHYDMNTAS LSQQQDQKPI

351 FNVIPPIPVG SENWNRCQGS GDDNLTSLGT LNFPGRTVFS NGYSSPSMRP

401 DVSSPPSSSS TATTGPPPKL CLVCSDEASG CHYGVLTCGS CKVFFKRAVE

451 GQHNYLCAGR NDCIIDKIRR KNCPACRYRK CLQAGMNLEA RKTKKKIKGI

501 QQATTGVSQE TSENPGNKTI VPATLPQLTP TL**VSLLEVIE** **PEVLYAGYDS**

551 **SVPDSTWRIM** **TTLNMLGGRQ** **VIAAVKWAKA** **IPGFRNLHLD** **DQMTLL**QYSW

601 MFL**MAFALGW** RSY**RQSSANL** **LCFAPDLIIN** **EQRMTLPCMY** **DQCKHMLYVS**

651 **SELHRLQVSY** **EEY**LCMKTLL **LLSSVPKDGL** **KSQELFDEIR** **MTY**IKELGKA

701 IVKREGNSSQ NWQRFYQLTK L**LDSMHEVVE** **NLLNY**CFQTF **LDKTMSIEFP**

751 **EMLAEIITNQ** **IPKYSNGNIK** **KLL**FHQK

Unformatted sequence string: 777 residues (for pasting into other applications).

Query	Start	– End	Observed	Mr(expt)	Mr(calc)	Delta	M	Score	Expect	Rank	U	Peptide
1096	533	– 544	670.3850	1338.7554	1338.7646	-0.0092	2	22	0.82	1Score **>** **38** indicates **identity**<br>Score **>** **21** indicates **homology**	U	L.VSLLEVIEPEVL.Y
607	536	– 544	520.7932	1039.5719	1039.5801	-0.0082	1	51	0.048	1Score **>** **38** indicates **identity**		L.LEVIEPEVL.Y
883	536	– 545	602.3250	1202.6355	1202.6434	-0.0079	2	57	0.013	1Score **>** **38** indicates **identity**		L.LEVIEPEVLY.A
695	537	– 545	545.7824	1089.5503	1089.5594	-0.0091	1	40	0.56	4Score **>** **38** indicates **identity**<br>Score **>** **37** indicates **homology**		L.EVIEPEVLY.A
1236	545	– 557	724.2996	1446.5847	1446.5939	-0.0093	2	25	0.14	1Score **>** **38** indicates **identity**<br>Score **>** **16** indicates **homology**	U	L.YAGYDSSVPDSTW.R
508	549	– 557	497.2099	992.4053	992.4087	-0.0034	0	25	0.26	1Score **>** **37** indicates **identity**<br>Score **>** **19** indicates **homology**	U	Y.DSSVPDSTW.R

Query	Start – End	Observed	Mr (expt)	Mr (calc)	Delta	M	Score	Expect	Rank	U	Peptide
509	549 – 557	497.2101	992.4057	992.4087	-0.0030	0	16	0.86	1 Score > 37 indicates identity Score > 14 indicates homology	U	Y.DSSVPDSTW.R
725	558 – 566	554.7942	1107.5739	1107.5780	-0.0041	1	55	0.016	1 Score > 38 indicates identity Score > 36 indicates homology	U	W.RIMTTLNML.G + Oxidation (M)
845	558 – 566	586.7805	1171.5464	1171.5494	-0.0031	1	36	0.22	1 Score > 38 indicates identity Score > 29 indicates homology	U	W.RIMTTLNML.G + Phospho (ST)
2115	558 – 577	593.3078	2369.2020	2369.2113	-0.0092	2	32	0.45	1 Score > 37 indicates identity Score > 28 indicates homology	U	W.RIMTTLNMLGGRQVIAAVKW.A + 2 Oxidation (M); Phospho (ST)
1364	564 – 577	514.9562	1541.8467	1541.8500	-0.0033	1	29	0.16	1 Score > 38 indicates identity Score > 20 indicates homology	U	L.NMLGGRQVIAAVKW.A
1380	564 – 577	779.9268	1557.8391	1557.8450	-0.0059	1	55	0.0086	1 Score > 38 indicates identity Score > 34 indicates homology	U	L.NMLGGRQVIAAVKW.A + Oxidation (M)
857	567 – 577	395.5676	1183.6809	1183.6826	-0.0017	0	55	0.021	1 Score > 38 indicates identity Score > 37 indicates homology	U	L.GGRQVIAAVKW.A
65	578 – 584	352.2070	702.3994	702.4064	-0.0071	0	41	0.053	1 Score > 28 indicates identity		W.AKAIPGF.R
1117	585 – 595	452.5617	1354.6634	1354.6663	-0.0030	2	43	0.26	1 Score > 38 indicates identity Score > 36 indicates homology		F.RNLHLDDQMTL.L
1130	585 – 595	686.3355	1370.6565	1370.6612	-0.0048	2	41	0.26	1 Score > 38 indicates identity Score > 34 indicates homology		F.RNLHLDDQMTL.L + Oxidation (M)
467	588 – 595	486.7247	971.4349	971.4382	-0.0033	1	45	0.045	1 Score > 38 indicates identity Score > 31 indicates homology		L.HLDDQMTL.L
504	588 – 595	494.7240	987.4335	987.4332	0.0003	1	42	0.042	1 Score > 38 indicates identity Score > 28 indicates homology		L.HLDDQMTL.L + Oxidation (M)
683	588 – 596	543.2644	1084.5142	1084.5223	-0.0081	2	40	0.19	1 Score > 38 indicates identity Score > 32 indicates homology		L.HLDDQMTLL.Q
135	604 – 610	398.1936	794.3726	794.3785	-0.0059	2	32	0.37	1 Score > 33 indicates identity Score > 28 indicates homology	U	L.MAFALGW.R
153	604 – 610	406.1914	810.3683	810.3734	-0.0051	2	42	0.17	1 Score > 34 indicates identity	U	L.MAFALGW.R + Oxidation (M)
285	614 – 621	444.7487	887.4829	887.4825	0.0004	1	46	0.12	1 Score > 37 indicates identity	U	Y.RQSSANLL.C
2137	622 – 640	796.6951	2387.0635	2387.0782	-0.0147	2	30	0.1	1 Score > 37 indicates identity Score > 20 indicates homology	U	L.CFAPDLIINEQRMTLPCMY.D + Oxidation (M)
2148	622 – 640	802.0277	2403.0613	2403.0731	-0.0118	2	33	0.12	1 Score > 37 indicates identity Score > 23 indicates homology	U	L.CFAPDLIINEQRMTLPCMY.D + 2 Oxidation (M)
2185	622 – 640	818.0169	2451.0287	2451.0496	-0.0209	2	19	0.69	2 Score > 37 indicates identity Score > 17 indicates homology	U	L.CFAPDLIINEQRMTLPCMY.D + Phospho (ST)
1886	624 – 640	694.3272	2079.9599	2079.9791	-0.0192	1	29	0.34	1 Score > 38 indicates identity Score > 24 indicates homology	U	F.APDLIINEQRMTLPCMY.D + Oxidation (M)
402	641 – 647	466.2102	930.4059	930.4052	0.0008	0	19	0.8	1 Score > 37 indicates identity Score > 17 indicates homology	U	Y.DQCKHML.Y
727	641 – 648	555.7389	1109.4632	1109.4634	-0.0002	1	32	0.82	2 Score > 38 indicates identity Score > 30 indicates homology	U	Y.DQCKHMLY.V + Oxidation (M)

Query	Start	– End	Observed	Mr(expt)	Mr(calc)	Delta	M	Score	Expect	Rank	U	Peptide
417	649	- 656	470.7635	939.5125	939.5138	-0.0012	1	39	0.07	1 Score > 37 indicates **identity** Score > 27 indicates **homology**	U	Y.VSSELHRL.Q
1190	649	- 660	473.2510	1416.7311	1416.7361	-0.0050	2	36	0.43	1 Score > 38 indicates **identity** Score > 32 indicates **homology**	U	Y.VSSELHRLQVSY.E
1070	654	- 663	662.3186	1322.6227	1322.6255	-0.0027	2	58	0.0025	1 Score > 38 indicates **identity** Score > 31 indicates **homology**	U	L.HRLQVSYEEY.L
1071	654	- 663	441.8815	1322.6228	1322.6255	-0.0027	2	26	0.46	1 Score > 38 indicates **identity** Score > 22 indicates **homology**	U	L.HRLQVSYEEY.L
580	671	- 680	514.8010	1027.5874	1027.5914	-0.0039	2	35	0.15	1 Score > 38 indicates **identity** Score > 26 indicates **homology**	U	L.LLSSVPKDGL.K
141	673	- 680	401.7185	801.4224	801.4232	-0.0009	0	37	0.23	1 Score > 35 indicates **identity** Score > 30 indicates **homology**	U	L.SSVPKDGL.K
1355	673	- 686	512.2738	1533.7995	1533.8039	-0.0043	2	27	0.92	1 Score > 38 indicates **identity** Score > 26 indicates **homology**	U	L.SSVPKDGLKSQELF.D
1546	681	- 693	830.4020	1658.7894	1658.7974	-0.0079	2	54	0.028	1 Score > 38 indicates **identity** Score > 38 indicates **homology**	U	L.KSQELFDEIRMTY.I
1572	681	- 693	559.2694	1674.7862	1674.7923	-0.0061	2	40	0.084	1 Score > 38 indicates **identity** Score > 29 indicates **homology**	U	L.KSQELFDEIRMTY.I + Oxidation (M)
666	686	- 693	537.7477	1073.4809	1073.4852	-0.0042	1	49	0.023	1 Score > 38 indicates **identity** Score > 32 indicates **homology**	U	L.FDEIRMTY.I
693	686	- 693	545.7461	1089.4776	1089.4801	-0.0025	1	44	0.061	1 Score > 38 indicates **identity** Score > 31 indicates **homology**	U	L.FDEIRMTY.I + Oxidation (M)
385	687	- 693	464.2143	926.4141	926.4167	-0.0026	0	36	0.32	1 Score > 37 indicates **identity** Score > 31 indicates **homology**	U	F.DEIRMTY.I
423	687	- 693	472.2121	942.4096	942.4117	-0.0021	0	23	0.85	1 Score > 37 indicates **identity** Score > 22 indicates **homology**	U	F.DEIRMTY.I + Oxidation (M)
1168	722	- 733	699.8454	1397.6762	1397.6860	-0.0099	2	64	0.0027	1 Score > 38 indicates **identity**	U	L.LDSMHEVVENLL.N
1188	722	- 733	707.8449	1413.6752	1413.6810	-0.0058	2	40	0.76	3 Score > 38 indicates **identity**	U	L.LDSMHEVVENLL.N + Oxidation (M)
1007	723	- 733	643.3054	1284.5963	1284.6020	-0.0057	1	42	0.5	3 Score > 38 indicates **identity**	U	L.DSMHEVVENLL.N
1383	723	- 735	781.8555	1561.6965	1561.7082	-0.0118	2	55	0.014	1 Score > 38 indicates **identity** Score > 36 indicates **homology**	U	L.DSMHEVVENLLNY.C
1401	723	- 735	789.8554	1577.6963	1577.7032	-0.0069	2	51	0.0068	1 Score > 38 indicates **identity** Score > 29 indicates **homology**	U	L.DSMHEVVENLLNY.C + Oxidation (M)
1411	741	- 753	529.2502	1584.7288	1584.7415	-0.0127	1	26	0.97	1 Score > 38 indicates **identity** Score > 25 indicates **homology**	U	F.LDKTMSIEFPEML.A + 2 Oxidation (M)
2352	741	- 764	942.1471	2823.4194	2823.4438	-0.0244	2	46	0.013	1 Score > 36 indicates **identity** Score > 26 indicates **homology**	U	F.LDKTMSIEFPEMLAEIITNQIPKY.S
2358	741	- 764	947.4760	2839.4062	2839.4387	-0.0325	2	34	0.25	1 Score > 36 indicates **identity** Score > 27 indicates **homology**	U	F.LDKTMSIEFPEMLAEIITNQIPKY.S + Oxidation (M)
2359	741	- 764	947.4764	2839.4073	2839.4387	-0.0314	2	37	0.12	1 Score > 36 indicates **identity** Score > 28 indicates **homology**	U	F.LDKTMSIEFPEMLAEIITNQIPKY.S + Oxidation (M)
2360	741	- 764	947.4771	2839.4094	2839.4387	-0.0293	2	56	0.012	1 Score > 36 indicates **identity** Score > 36 indicates **homology**	U	F.LDKTMSIEFPEMLAEIITNQIPKY.S + Oxidation (M)

Query	Start – End	Observed	Mr(expt)	Mr(calc)	Delta	M	Score	Expect	Rank	U	Peptide
2366	741 – 764	952.8061	2855.3965	2855.4336	-0.0371	2	60	0.00057	1Score > 36 indicates identity Score > 27 indicates homology	U	F.LDKTMSIEFPEMLAEIITNQIPKY.S + 2 Oxidation (M)
1246	742 – 753	728.8305	1455.6465	1455.6625	-0.0161	0	30	0.45	1Score > 38 indicates identity Score > 26 indicates homology	U	L.DKTMSIEFPEML.A + Oxidation (M)
1018	754 – 764	645.3568	1288.6990	1288.7027	-0.0037	0	51	0.0014	1Score > 38 indicates identity Score > 23 indicates homology	U	L.AEIITNQIPKY.S
497	765 – 773	493.8031	985.5917	985.5920	-0.0003	1	45	0.22	2Score > 38 indicates identity	U	Y.SNGNIKKLL.F

MST2 chymotrypsin digest

Glucocorticoid receptor OS=Homo sapiens OX=9606 GN=NR3C1 PE=1 SV=1

**Database:**	SwissProt
**Score:**	601
**Monoisotopic mass (M_r):**	86745
**Calculated pI:**	6.00
**Taxonomy:**	Homo sapiens

Sequence similarity is available as an NCBI BLAST search of GCR_HUMAN against nr.

Search parameters

**MS data file:**	D:\SCIEX OS Data\MGF files\May2018\300818 LBD GR MST2 chymo.mgf
**Enzyme:**	Chymotrypsin: cuts C-term side of FLWY unless next residue is P.
**Fixed modifications:**	Carbamidomethyl (C)
**Variable modifications:**	Oxidation (M), Phospho (ST), Phospho (Y)

Protein sequence coverage: 23%

Matched peptides shown in ***bold red***.

1 MDSKESLTPG REENPSSVLA QERGDVMDFY KTLRGGATVK VSASSPSLAV

51 ASQSDSKQRR LLVDFPKGSV SNAQQPDLSK AVSLSMGLYM GETETKVMGN

101 DLGFPQQGQI SLSSGETDLK LLEESIANLN RSTSVPENPK SSASTAVSAA

151 PTEKEFPKTH SDVSSEQQHL KGQTGTNGGN VKLYTTDQST FDILQDLEFS

201 SGSPGKETNE SPWRSDLLID ENCLLSPLAG EDDSFLLEGN SNEDCKPLIL

251 PDTKPKIKDN GDLVLSSPSN VTLPQVKTEK EDFIELCTPG VIKQEKLGTV

301 YCQASFPGAN IIGNKMSAIS VHGVSTSGGQ MYHYDMNTAS LSQQQDQKPI

351 FNVIPPIPVG SENWNRCQGS GDDNLTSLGT LNFPGRTVFS NGYSSPSMRP

401 DVSSPPSSSS TATTGPPPKL CLVCSDEASG CHYGVLTCGS CKVFFKRAVE

451 GQHNYLCAGR NDCIIDKIRR KNCPACRYRK CLQAGMNLEA RKTKKKIKGI

501 QQATTGVSQE TSENPGNKTI VPATLPQLTP TLVSL**LEVIE** **PEVLYAGYDS**

551 **SVPDSTWRIM** **TTLNMLGGRQ** **VIAAVKWAKA** **IPGFRNLHLD** **DQMTLL**QYSW

601 MFLMAF**ALGW** **RSYRQSSANL** **LCFAPDLIIN** **EQRMTLPCMY** **DQCKHMLYVS**

651 **SELHRLQVSY** **EEY**LCMKTLL **LLSSVPKDGL** **KSQELFDEIR** **MTY**IKELGKA

701 IVKREGNSSQ NWQRFYQLTK L**LDSMHEVVE** **NLLNY**CFQTF **LDKTMSIEFP**

751 **EMLAEIITNQ** **IPKY**SNGNIK KLLFHQK

Unformatted sequence string: 777 residues (for pasting into other applications).

Query	Start	–	End	Observed	Mr(expt)	Mr(calc)	Delta	M	Score	Expect	Rank		U	Peptide
578	536	–	544	520.7943	1039.5741	1039.5801	-0.0060	1	44	0.15	1	Score **>** **38** indicates **identity** Score **>** **36** indicates **homology**		L.LEVIEPEVL.Y
833	536	–	545	602.3261	1202.6376	1202.6434	-0.0059	2	69	0.00095	1	Score **>** **38** indicates **identity**		L.LEVIEPEVLY.A
666	537	–	545	545.7853	1089.5560	1089.5594	-0.0034	1	41	0.55	4	Score **>** **38** indicates **identity** Score **>** **38** indicates **homology**		L.EVIEPEVLY.A
1166	545	–	557	724.3004	1446.5863	1446.5939	-0.0077	2	23	0.26	1	Score **>** **38** indicates **identity** Score **>** **16** indicates **homology**	U	L.YAGYDSSVPDSTW.R

Query	Start	–	End	Observed	Mr(expt)	Mr(calc)	Delta	M	Score	Expect	Rank	U	Peptide
948	546	–	557	642.7708	1283.5270	1283.5306	-0.0036	1	24	0.29	1 Score **>** **38** indicates **identity** Score **>** **18** indicates **homology**	U	Y.AGYDSSVPDSTW.R
496	549	–	557	497.2104	992.4062	992.4087	-0.0025	0	27	0.81	1 Score **>** **37** indicates **identity** Score **>** **25** indicates **homology**	U	Y.DSSVPDSTW.R
798	558	–	566	586.7802	1171.5459	1171.5494	-0.0035	1	37	0.11	1 Score **>** **38** indicates **identity** Score **>** **27** indicates **homology**		W.RIMTTLNML.G + Phospho (ST)
1879	558	–	577	593.3088	2369.2062	2369.2113	-0.0051	2	36	0.064	1 Score **>** **37** indicates **identity** Score **>** **23** indicates **homology**		W.RIMTTLNMLGGRQVIAAVKW.A + 2 Oxidation (M); Phospho (ST)
1266	564	–	577	514.9561	1541.8464	1541.8500	-0.0036	1	37	0.019	1 Score **>** **38** indicates **identity** Score **>** **19** indicates **homology**		L.NMLGGRQVIAAVKW.A
1282	564	–	577	520.2874	1557.8404	1557.8450	-0.0045	1	35	0.031	1 Score **>** **38** indicates **identity** Score **>** **20** indicates **homology**		L.NMLGGRQVIAAVKW.A + Oxidation (M)
812	567	–	577	592.8490	1183.6835	1183.6826	0.0009	0	40	0.63	1 Score **>** **38** indicates **identity**		L.GGRQVIAAVKW.A
65	578	–	584	352.2100	702.4055	702.4064	-0.0010	0	41	0.052	1 Score **>** **28** indicates **identity**		W.AKAIPGF.R
655	578	–	587	543.8253	1085.6361	1085.6345	0.0016	1	30	0.66	2 Score **>** **38** indicates **identity** Score **>** **28** indicates **homology**		W.AKAIPGFRNL.H
1071	585	–	595	686.3402	1370.6658	1370.6612	0.0045	2	50	0.018	1 Score **>** **38** indicates **identity** Score **>** **32** indicates **homology**		F.RNLHLDDQMTL.L + Oxidation (M)
490	588	–	595	494.7248	987.4350	987.4332	0.0018	1	44	0.13	1 Score **>** **38** indicates **identity** Score **>** **35** indicates **homology**		L.HLDDQMTL.L + Oxidation (M)
653	588	–	596	543.2654	1084.5162	1084.5223	-0.0061	2	39	0.2	1 Score **>** **38** indicates **identity** Score **>** **32** indicates **homology**		L.HLDDQMTLL.Q
214	607	–	613	426.7213	851.4280	851.4290	-0.0010	2	36	0.65	1 Score **>** **35** indicates **identity** Score **>** **34** indicates **homology**		F.ALGWRSY.R
288	614	–	621	444.7490	887.4835	887.4825	0.0010	1	46	0.13	5 Score **>** **37** indicates **identity**	U	Y.RQSSANLL.C
1898	622	–	640	796.6968	2387.0687	2387.0782	-0.0095	2	31	0.16	1 Score **>** **37** indicates **identity** Score **>** **23** indicates **homology**	U	L.CFAPDLIINEQRMTLPCMY.D + Oxidation (M)
1910	622	–	640	802.0282	2403.0627	2403.0731	-0.0104	2	30	0.57	1 Score **>** **37** indicates **identity** Score **>** **27** indicates **homology**	U	L.CFAPDLIINEQRMTLPCMY.D + 2 Oxidation (M)
1944	622	–	640	818.0201	2451.0385	2451.0496	-0.0111	2	25	0.3	1 Score **>** **37** indicates **identity** Score **>** **20** indicates **homology**	U	L.CFAPDLIINEQRMTLPCMY.D + Phospho (ST)
1682	624	–	640	694.3307	2079.9704	2079.9791	-0.0088	1	31	0.22	1 Score **>** **38** indicates **identity** Score **>** **24** indicates **homology**	U	F.APDLIINEQRMTLPCMY.D + Oxidation (M)
697	641	–	648	555.7406	1109.4667	1109.4634	0.0033	1	34	0.97	1 Score **>** **38** indicates **identity** Score **>** **33** indicates **homology**	U	Y.DQCKHMLY.V + Oxidation (M)
405	649	–	656	470.7651	939.5156	939.5138	0.0018	1	48	0.039	1 Score **>** **37** indicates **identity** Score **>** **33** indicates **homology**	U	Y.VSSELHRL.Q
1015	654	–	663	441.8820	1322.6242	1322.6255	-0.0013	2	26	0.45	1 Score **>** **38** indicates **identity** Score **>** **22** indicates **homology**	U	L.HRLQVSYEEY.L
556	671	–	680	514.8020	1027.5894	1027.5914	-0.0019	2	44	0.029	1 Score **>** **38** indicates **identity** Score **>** **28** indicates **homology**	U	L.LLSSVPKDGL.K
1225	672	–	685	500.9475	1499.8207	1499.8195	0.0012	2	32	0.049	1 Score **>** **38** indicates **identity** Score **>** **19** indicates **homology**	U	L.LSSVPKDGLKSQEL.F
1093	673	–	685	694.3754	1386.7363	1386.7354	0.0009	1	26	0.44	1 Score **>** **38** indicates **identity** Score **>** **22** indicates **homology**	U	L.SSVPKDGLKSQEL.F

Query	Start – End	Observed	Mr(expt)	Mr(calc)	Delta	M	Score	Expect	Rank	U	Peptide
1427	681 – 693	553.9383	1658.7930	1658.7974	-0.0044	2	44	0.22	1Score > 38 indicates identity Score > 37 indicates homology		L.KSQELFDEIRMTY.I
1450	681 – 693	559.2706	1674.7900	1674.7923	-0.0023	2	64	0.00015	1Score > 38 indicates identity Score > 26 indicates homology		L.KSQELFDEIRMTY.I + Oxidation (M)
639	686 – 693	537.7478	1073.4810	1073.4852	-0.0042	1	49	0.023	1Score > 38 indicates identity Score > 32 indicates homology		L.FDEIRMTY.I
664	686 – 693	545.7467	1089.4789	1089.4801	-0.0012	1	44	0.059	1Score > 38 indicates identity Score > 31 indicates homology		L.FDEIRMTY.I + Oxidation (M)
665	686 – 693	545.7479	1089.4813	1089.4801	0.0012	1	46	0.17	1Score > 38 indicates identity		L.FDEIRMTY.I + Oxidation (M)
375	687 – 693	464.2152	926.4159	926.4167	-0.0009	0	38	0.18	1Score > 37 indicates identity Score > 30 indicates homology		F.DEIRMTY.I
410	687 – 693	472.2132	942.4119	942.4117	0.0002	0	37	0.18	1Score > 37 indicates identity Score > 29 indicates homology		F.DEIRMTY.I + Oxidation (M)
1109	722 – 733	699.8478	1397.6811	1397.6860	-0.0049	2	67	0.0015	1Score > 38 indicates identity	U	L.LDSMHEVVENLL.N
1128	722 – 733	707.8457	1413.6768	1413.6810	-0.0042	2	55	0.024	1Score > 38 indicates identity	U	L.LDSMHEVVENLL.N + Oxidation (M)
954	723 – 733	643.3039	1284.5932	1284.6020	-0.0088	1	43	0.39	1Score > 38 indicates identity	U	L.DSMHEVVENLL.N
985	723 – 733	651.3050	1300.5955	1300.5969	-0.0014	1	43	0.34	5Score > 38 indicates identity	U	L.DSMHEVVENLL.N + Oxidation (M)
1285	723 – 735	521.5738	1561.6996	1561.7082	-0.0087	2	47	0.031	1Score > 38 indicates identity Score > 32 indicates homology	U	L.DSMHEVVENLLNY.C
1304	723 – 735	789.8564	1577.6982	1577.7032	-0.0050	2	55	0.015	1Score > 38 indicates identity Score > 36 indicates homology	U	L.DSMHEVVENLLNY.C + Oxidation (M)
1276	741 – 753	777.3793	1552.7440	1552.7517	-0.0076	1	56	0.012	1Score > 38 indicates identity Score > 37 indicates homology	U	F.LDKTMSIEFPEML.A
1312	741 – 753	793.3736	1584.7327	1584.7415	-0.0088	1	41	0.035	1Score > 38 indicates identity Score > 26 indicates homology	U	F.LDKTMSIEFPEML.A + 2 Oxidation (M)
1396	741 – 753	817.3614	1632.7082	1632.7180	-0.0098	1	26	0.15	1Score > 38 indicates identity Score > 18 indicates homology	U	F.LDKTMSIEFPEML.A + Phospho (ST)
2072	741 – 764	942.1487	2823.4242	2823.4438	-0.0196	2	45	0.032	1Score > 36 indicates identity Score > 30 indicates homology	U	F.LDKTMSIEFPEMLAEIITNQIPKY.S
2077	741 – 764	947.4792	2839.4159	2839.4387	-0.0228	2	64	0.00066	1Score > 36 indicates identity Score > 32 indicates homology	U	F.LDKTMSIEFPEMLAEIITNQIPKY.S + Oxidation (M)
2081	741 – 764	952.8123	2855.4151	2855.4336	-0.0185	2	45	0.011	1Score > 36 indicates identity Score > 25 indicates homology	U	F.LDKTMSIEFPEMLAEIITNQIPKY.S + 2 Oxidation (M)
1174	742 – 753	728.8354	1455.6563	1455.6625	-0.0063	0	26	0.59	1Score > 38 indicates identity Score > 24 indicates homology	U	L.DKTMSIEFPEML.A + Oxidation (M)
964	754 – 764	645.3572	1288.6998	1288.7027	-0.0029	0	52	0.014	1Score > 38 indicates identity Score > 33 indicates homology		L.AEIITNQIPKY.S

Rock1 chymotrypsin digest

Glucocorticoid receptor OS=Homo sapiens OX=9606 GN=NR3C1 PE=1 SV=1

**Database:**	SwissProt
**Score:**	523
**Monoisotopic mass (M_r):**	86745
**Calculated pI:**	6.00
**Taxonomy:**	Homo sapiens

Sequence similarity is available as an NCBI BLAST search of GCR_HUMAN against nr.

Search parameters

**MS data file:**	D:\SCIEX OS Data\MGF files\May2018\300818 LBD GR Rock1 chymo.mgf
**Enzyme:**	Chymotrypsin: cuts C-term side of FLWY unless next residue is P.
**Fixed modifications:**	Carbamidomethyl (C)
**Variable modifications:**	Oxidation (M), Phospho (ST), Phospho (Y)

Protein sequence coverage: 24%

Matched peptides shown in ***bold red***.

```
  1 MDSKESLTPG REENPSSVLA QERGDVMDFY KTLRGGATVK VSASSPSLAV
 51 ASQSDSKQRR LLVDFPKGSV SNAQQPDLSK AVSLSMGLYM GETETKVMGN
101 DLGFPQQGQI SLSSGETDLK LLEESIANLN RSTSVPENPK SSASTAVSAA
151 PTEKEFPKTH SDVSSEQQHL KGQTGTNGGN VKLYTTDQST FDILQDLEFS
```

201 SGSPGKETNE SPWRSDLLID ENCLLSPLAG EDDSFLLEGN SNEDCKPLIL

251 PDTKPKIKDN GDLVLSSPSN VTLPQVKTEK EDFIELCTPG VIKQEKLGTV

301 YCQASFPGAN IIGNKMSAIS VHGVSTSGGQ MYHYDMNTAS LSQQQDQKPI

351 FNVIPPIPVG SENWNRCQGS GDDNLTSLGT LNFPGRTVFS NGYSSPSMRP

401 DVSSPPSSSS TATTGPPPKL CLVCSDEASG CHYGVLTCGS CKVFFKRAVE

451 GQHNYLCAGR NDCIIDKIRR KNCPACRYRK CLQAGMNLEA RKTKKKIKGI

501 QQATTGVSQE TSENPGNKTI VPATLPQLTP TL**VSLLEVIE** **PEVLYAGYDS**

551 **SVPDSTW**RIM TTL**NMLGGRQ** **VIAAVKWAKA** **IPGF**RNL**HLD** **DQMTLL**QYSW

601 MFL**MAFALGW** **RSYRQSSANL** **LCFAPDLIIN** **EQRMTLPCMY** DQCKHMLY**VS**

651 **SELHRLQVSY** **EEYLCMKTLL** **LLSSVPKDGL** **KSQELFDEIR** **MTY**IKELGKA

701 IVKREGNSSQ NWQRFYQLTK L**LDSMHEVVE** **NLLNYCFQTF** **LDKTMSIEFP**

751 **EMLAEIITNQ** **IPKYSNGNIK** **KLL**FHQK

Unformatted sequence string: 777 residues (for pasting into other applications).

Query	Start	End	Observed	Mr(expt)	Mr(calc)	Delta	M	Score	Expect	Rank	U	Peptide
967	533	544	670.3863	1338.7580	1338.7646	-0.0066	2	27	0.63	1Score > 38 indicates identity Score > 24 indicates homology	U	L.VSLLEVIEPEVL.Y
813	536	545	602.3275	1202.6405	1202.6434	-0.0030	2	40	0.72	4Score > 38 indicates identity	U	L.LEVIEPEVLY.A
658	537	545	545.7864	1089.5582	1089.5594	-0.0012	1	43	0.36	1Score > 38 indicates identity	U	L.EVIEPEVLY.A
1077	545	557	724.3015	1446.5884	1446.5939	-0.0055	2	25	0.06	1Score > 38 indicates identity Score > 13 indicates homology	U	L.YAGYDSSVPDSTW.R
891	546	557	642.7719	1283.5293	1283.5306	-0.0013	1	25	0.076	1Score > 38 indicates identity Score > 13 indicates homology	U	Y.AGYDSSVPDSTW.R
497	549	557	497.2112	992.4078	992.4087	-0.0009	0	25	0.71	1Score > 37 indicates identity Score > 23 indicates homology	U	Y.DSSVPDSTW.R
1160	564	577	514.9557	1541.8453	1541.8500	-0.0047	1	33	0.38	1Score > 38 indicates identity Score > 29 indicates homology	U	L.NMLGGRQVIAAVKW.A
1170	564	577	520.2885	1557.8437	1557.8450	-0.0013	1	32	0.041	1Score > 38 indicates identity Score > 18 indicates homology	U	L.NMLGGRQVIAAVKW.A + Oxidation (M)
1504	564	584	748.3640	2242.0702	2242.2408	-0.1707	2	24	0.74	1Score > 38 indicates identity Score > 22 indicates homology	U	L.NMLGGRQVIAAVKWAKAIPGF.R + Oxidation (M)
791	567	577	592.8490	1183.6835	1183.6826	0.0009	0	36	0.67	2Score > 38 indicates identity Score > 34 indicates homology	U	L.GGRQVIAAVKW.A
62	578	584	352.2097	702.4049	702.4064	-0.0015	0	42	0.038	1Score > 28 indicates identity	U	W.AKAIPGF.R

Query	Start	–	End	Observed	Mr(expt)	Mr(calc)	Delta	M	Score	Expect	Rank		U	Peptide
492	588	–	595	494.7242	987.4339	987.4332	0.0007	1	45	0.12	1	Score **>** **38** indicates **identity** Score **>** **35** indicates **homology**	U	L.HLDDQMTL.L + Oxidation (M)
647	588	–	596	543.2673	1084.5200	1084.5223	-0.0023	2	40	0.53	1	Score **>** **38** indicates **identity** Score **>** **37** indicates **homology**	U	L.HLDDQMTLL.Q
145	604	–	610	398.1952	794.3758	794.3785	-0.0028	2	28	0.68	1	Score **>** **33** indicates **identity** Score **>** **26** indicates **homology**	U	L.MAFALGW.R
214	607	–	613	426.7221	851.4297	851.4290	0.0007	2	40	0.3	1	Score **>** **35** indicates **identity** Score **>** **34** indicates **homology**	U	F.ALGWRSY.R
283	614	–	621	444.7497	887.4848	887.4825	0.0023	1	43	0.27	10	Score **>** **37** indicates **identity**	U	Y.RQSSANLL.C
284	614	–	621	444.7498	887.4851	887.4825	0.0026	1	39	0.64	4	Score **>** **37** indicates **identity**	U	Y.RQSSANLL.C
1574	622	–	640	802.0310	2403.0711	2403.0731	-0.0020	2	32	0.036	1	Score **>** **37** indicates **identity** Score **>** **17** indicates **homology**	U	L.CFAPDLIINEQRMTLPCMY.D + 2 Oxidation (M)
1461	624	–	640	699.6642	2095.9707	2095.9740	-0.0034	1	32	0.17	1	Score **>** **38** indicates **identity** Score **>** **24** indicates **homology**	U	F.APDLIINEQRMTLPCMY.D + 2 Oxidation (M)
399	649	–	656	470.7669	939.5193	939.5138	0.0056	1	39	0.22	1	Score **>** **37** indicates **identity** Score **>** **32** indicates **homology**	U	Y.VSSELHRL.Q
948	654	–	663	662.3209	1322.6272	1322.6255	0.0017	2	49	0.021	1	Score **>** **38** indicates **identity** Score **>** **31** indicates **homology**	U	L.HRLQVSYEEY.L
291	664	–	670	447.7444	893.4743	893.4714	0.0029	2	40	0.34	1	Score **>** **36** indicates **identity** Score **>** **35** indicates **homology**	U	Y.LCMKTLL.L + Oxidation (M)
557	671	–	680	514.8025	1027.5905	1027.5914	-0.0008	2	56	0.0051	1	Score **>** **38** indicates **identity** Score **>** **33** indicates **homology**	U	L.LLSSVPKDGL.K
153	673	–	680	401.7184	801.4223	801.4232	-0.0009	0	37	0.23	1	Score **>** **35** indicates **identity** Score **>** **30** indicates **homology**	U	L.SSVPKDGL.K
1275	681	–	693	553.9388	1658.7944	1658.7974	-0.0029	2	55	0.0017	1	Score **>** **38** indicates **identity** Score **>** **27** indicates **homology**	U	L.KSQELFDEIRMTY.I
1291	681	–	693	559.2709	1674.7909	1674.7923	-0.0014	2	47	0.019	1	Score **>** **38** indicates **identity** Score **>** **29** indicates **homology**	U	L.KSQELFDEIRMTY.I + Oxidation (M)
631	686	–	693	537.7491	1073.4837	1073.4852	-0.0015	1	49	0.025	1	Score **>** **38** indicates **identity** Score **>** **33** indicates **homology**	U	L.FDEIRMTY.I
656	686	–	693	545.7457	1089.4769	1089.4801	-0.0032	1	44	0.061	1	Score **>** **38** indicates **identity** Score **>** **31** indicates **homology**	U	L.FDEIRMTY.I + Oxidation (M)
657	686	–	693	545.7482	1089.4818	1089.4801	0.0017	1	41	0.11	1	Score **>** **38** indicates **identity** Score **>** **31** indicates **homology**	U	L.FDEIRMTY.I + Oxidation (M)
366	687	–	693	464.2172	926.4199	926.4167	0.0032	0	34	0.18	1	Score **>** **37** indicates **identity** Score **>** **26** indicates **homology**	U	F.DEIRMTY.I
404	687	–	693	472.2136	942.4127	942.4117	0.0010	0	34	0.63	1	Score **>** **37** indicates **identity** Score **>** **31** indicates **homology**	U	F.DEIRMTY.I + Oxidation (M)
1028	722	–	733	699.8493	1397.6841	1397.6860	-0.0019	2	38	0.72	1	Score **>** **38** indicates **identity** Score **>** **36** indicates **homology**	U	L.LDSMHEVVENLL.N
1045	722	–	733	707.8472	1413.6799	1413.6810	-0.0011	2	57	0.015	1	Score **>** **38** indicates **identity**	U	L.LDSMHEVVENLL.N + Oxidation (M)
898	723	–	733	643.3069	1284.5993	1284.6020	-0.0027	1	50	0.03	1	Score **>** **38** indicates **identity** Score **>** **34** indicates **homology**	U	L.DSMHEVVENLL.N
921	723	–	733	651.3061	1300.5976	1300.5969	0.0007	1	40	0.68	4	Score **>** **38** indicates **identity**	U	L.DSMHEVVENLL.N

Query	Start – End	Observed	Mr(expt)	Mr(calc)	Delta	M	Score	Expect	Rank	U	Peptide
1174	723 – 735	521.5757	1561.7053	1561.7082	-0.0029	2	47	0.091	1Score > 38 indicates identity<br>Score > 36 indicates homology	U	L.DSMHEVVENLLNY.C<br>+ Oxidation (M)
1183	723 – 735	789.8584	1577.7023	1577.7032	-0.0009	2	59	0.0099	1Score > 38 indicates identity	U	L.DSMHEVVENLLNY.C
464	734 – 740	490.2022	978.3899	978.3906	-0.0007	2	24	0.81	1Score > 37 indicates identity<br>Score > 22 indicates homology	U	L.NYCFQTF.L<br>+ Oxidation (M)
1165	741 – 753	777.3820	1552.7494	1552.7517	-0.0023	1	49	0.086	1Score > 38 indicates identity	U	F.LDKTMSIEFPEML.A
1177	741 – 753	785.3793	1568.7440	1568.7466	-0.0026	1	31	0.24	1Score > 38 indicates identity<br>Score > 24 indicates homology	U	F.LDKTMSIEFPEML.A
1191	741 – 753	529.2536	1584.7391	1584.7415	-0.0024	1	30	0.95	1Score > 38 indicates identity<br>Score > 30 indicates homology	U	F.LDKTMSIEFPEML.A<br>+ Oxidation (M)
1670	741 – 764	942.1525	2823.4356	2823.4438	-0.0082	2	54	0.018	1Score > 36 indicates identity	U	F.LDKTMSIEFPEMLAEIITNQIPKY.S<br>+ 2 Oxidation (M)
1675	741 – 764	947.4847	2839.4324	2839.4387	-0.0063	2	58	0.0014	1Score > 36 indicates identity<br>Score > 29 indicates homology	U	F.LDKTMSIEFPEMLAEIITNQIPKY.S
1678	741 – 764	952.8147	2855.4222	2855.4336	-0.0115	2	62	0.0002	1Score > 36 indicates identity<br>Score > 25 indicates homology	U	F.LDKTMSIEFPEMLAEIITNQIPKY.S<br>+ Oxidation (M)
905	754 – 764	645.3601	1288.7056	1288.7027	0.0029	0	62	0.00032	1Score > 38 indicates identity<br>Score > 27 indicates homology	U	L.AEIITNQIPKY.S<br>+ 2 Oxidation (M)
484	765 – 773	493.8045	985.5944	985.5920	0.0024	1	40	0.59	4Score > 38 indicates identity	U	Y.SNGNIKKLL.F

2.2.3. Chymotrypsin and trypsin digestion and data analysis using the internal database: UKCM.

MAP4K4 trypsin and chymotrypsin digest

Prot0560

**Database:**	UserUKCM
**Score:**	1603
**Monoisotopic mass (M_r):**	32690
**Calculated pI:**	6.83

Sequence similarity is available as an NCBI BLAST search of Prot0560 against nr.

Search parameters

**MS data file:**	D:\SCIEX OS Data\MGF files\May2018\300818 LBD GR MAP4K4 double.mgf
**Enzyme:**	TrypChymo: cuts C-term side of FKLRWY unless next residue is P.
**Fixed modifications:**	Carbamidomethyl (C)
**Variable modifications:**	Oxidation (M), Phospho (ST), Phospho (Y)

Protein sequence coverage: 68%

Matched peptides shown in ***bold red***.

```
  1 MHHHHHHGGE NLYFQGNPGN KTIVPATLPQ LTPTLVSLLE VIEPEVLYAG
 51 YDSSVPDSTW RIMTTLNMLG GRQVIAAVKW AKAIPGFRNL HLDDQMTLLQ
101 YSWMSLMAFA LGWRSYRQSS ANLLCFAPDL IINEQRMTLP CMYDQCKHML
151 YVSSELHRLQ VSYEEYLCMK TLLLLSSVPK DGLKSQELFD EIRMTYIKEL
201 GKAIVKREGN SSQNWQRFYQ LTKLLDSMHE VVENLLNYCF QTFLDKTMSI
251 EFPEMLAEII TNQIPKYSNG NIKKLLFHQK
```

Unformatted sequence string: 280 residues (for pasting into other applications).

Query	Start – End	Observed	Mr(expt)	Mr(calc)	Delta	M	Score	Expect	Rank		U	Peptide
236	40 – 47	464.2558	926.4971	926.4960	0.0011	0	44	0.00032	1	Score > 13 indicates **identity**	U	L.EVIEPEVL.Y
428	40 – 48	545.7868	1089.5591	1089.5594	-0.0003	1	40	0.00065	1	Score > 13 indicates **identity**	U	L.EVIEPEVLY.A
570	49 – 60	642.7725	1283.5304	1283.5306	-0.0003	1	20	0.012	1	Score > 13 indicates **identity** Score > 13 indicates **homology**	U	Y.AGYDSSVPDSTW.R
661	49 – 61	720.8230	1439.6314	1439.6317	-0.0003	2	41	0.00023	1	Score > 13 indicates **identity** Score > 13 indicates **homology**	U	Y.AGYDSSVPDSTWR.I
319	52 – 60	497.2110	992.4075	992.4087	-0.0012	0	18	0.068	1	Score > 13 indicates **identity** Score > 13 indicates **homology**	U	Y.DSSVPDSTW.R
320	52 – 60	497.2118	992.4091	992.4087	0.0003	0	20	0.078	1	Score > 13 indicates **identity**	U	Y.DSSVPDSTW.R

Query	Start	-	End	Observed	Mr(expt)	Mr(calc)	Delta	M	Score	Expect	Rank	U	Peptide
											Score > 13 indicates **homology**		
475	52	-	61	575.2617	1148.5088	1148.5098	-0.0010	1	47	6e-05	1Score > 13 indicates **identity** Score > 13 indicates **homology**	U	Y.DSSVPDSTWR.I
476	52	-	61	575.2630	1148.5114	1148.5098	0.0016	1	59	3.1e-06	1Score > 13 indicates **identity** Score > 13 indicates **homology**	U	Y.DSSVPDSTWR.I
340	62	-	69	508.7833	1015.5520	1015.4483	0.1037	1	19	0.085	1Score > 13 indicates **identity**	U	R.IMTTLNML.G + Phospho (ST)
517	62	-	72	603.8185	1205.6224	1205.6260	-0.0036	2	77	1e-07	1Score > 13 indicates **identity** Score > 13 indicates **homology**	U	R.IMTTLNMLGGR.Q
530	62	-	72	611.8178	1221.6210	1221.6210	0.0000	2	54	2.1e-05	1Score > 13 indicates **identity** Score > 13 indicates **homology**	U	R.IMTTLNMLGGR.Q + Oxidation (M)
539	62	-	72	619.8165	1237.6184	1237.6159	0.0025	2	76	1.4e-07	1Score > 13 indicates **identity**	U	R.IMTTLNMLGGR.Q + 2 Oxidation (M)
52	73	-	79	364.7380	727.4615	727.4592	0.0022	0	44	4e-05	1Score > 13 indicates **identity**	U	R.QVIAAVK.W
223	73	-	80	457.7752	913.5358	913.5385	-0.0027	1	57	1.1e-05	1Score > 13 indicates **identity**	U	R.QVIAAVKW.A
150	81	-	88	430.2615	858.5084	858.5076	0.0008	2	22	0.029	1Score > 13 indicates **identity**	U	W.AKAIPGFR.N
512	89	-	98	600.2898	1198.5650	1198.5652	-0.0003	2	39	0.00046	1Score > 13 indicates **identity** Score > 13 indicates **homology**	U	R.NLHLDDQMTL.L
526	89	-	98	608.2870	1214.5594	1214.5601	-0.0007	2	27	0.0087	1Score > 13 indicates **identity** Score > 13 indicates **homology**	U	R.NLHLDDQMTL.L + Oxidation (M)
289	91	-	98	486.7278	971.4411	971.4382	0.0029	1	36	0.00046	1Score > 13 indicates **identity** Score > 13 indicates **homology**	U	L.HLDDQMTL.L
317	91	-	98	494.7254	987.4362	987.4332	0.0031	1	39	0.00079	1Score > 13 indicates **identity** Score > 13 indicates **homology**	U	L.HLDDQMTL.L + Oxidation (M)
422	91	-	99	543.2675	1084.5205	1084.5223	-0.0018	2	42	0.00046	1Score > 13 indicates **identity**	U	L.HLDDQMTLL.Q
434	91	-	99	551.2659	1100.5172	1100.5172	0.0000	2	35	0.0015	1Score > 13 indicates **identity** Score > 13 indicates **homology**	U	L.HLDDQMTLL.Q + Oxidation (M)

Query	Start – End	Observed	Mr(expt)	Mr(calc)	Delta	M	Score	Expect	Rank	U	Peptide
241	100 – 106	465.7039	929.3932	929.3953	-0.0021	2	22	0.0082	1 Score > 13 indicates **identity** Score > 13 indicates **homology**	U	L.QYSWMSL.M + Oxidation (M)
316	102 – 109	494.7164	987.4182	987.4194	-0.0012	2	20	0.039	1 Score > 13 indicates **identity** Score > 13 indicates **homology**	U	Y.SWMSLMAF.A + Oxidation (M)
329	102 – 109	502.7140	1003.4135	1003.4143	-0.0008	2	24	0.041	1 Score > 13 indicates **identity**	U	Y.SWMSLMAF.A + 2 Oxidation (M)
108	107 – 113	406.1944	810.3743	810.3734	0.0008	2	30	0.0033	1 Score > 13 indicates **identity**	U	L.MAFALGW.R + Oxidation (M)
141	117 – 123	428.2589	854.5033	854.3647	0.1386	1	18	0.056	1 Score > 13 indicates **identity**	U	Y.RQSSANL.L + Phospho (ST)
184	117 – 124	444.7499	887.4852	887.4825	0.0028	2	43	0.00024	1 Score > 13 indicates **identity**	U	Y.RQSSANLL.C
55	118 – 124	366.6981	731.3817	731.3813	0.0004	1	21	0.021	1 Score > 13 indicates **identity**	U	R.QSSANLL.C
700	125 – 136	492.5814	1474.7224	1474.7238	-0.0014	2	41	0.00028	1 Score > 13 indicates **identity** Score > 13 indicates **homology**	U	L.CFAPDLIINEQR.M
492	127 – 136	584.8178	1167.6211	1167.6248	-0.0037	1	18	0.1	1 Score > 13 indicates **identity**	U	F.APDLIINEQR.M
493	127 – 136	584.8205	1167.6264	1167.6248	0.0016	1	50	2.8e-05	1 Score > 13 indicates **identity** Score > 13 indicates **homology**	U	F.APDLIINEQR.M
494	127 – 136	584.8208	1167.6270	1167.6248	0.0022	1	69	3.7e-07	1 Score > 13 indicates **identity** Score > 13 indicates **homology**	U	F.APDLIINEQR.M
224	137 – 143	458.1919	914.3693	914.3700	-0.0007	0	22	0.047	1 Score > 13 indicates **identity** Score > 13 indicates **homology**	U	R.MTLPCMY.D
247	137 – 143	466.1892	930.3638	930.3649	-0.0012	0	30	0.0078	1 Score > 13 indicates **identity** Score > 13 indicates **homology**	U	R.MTLPCMY.D + Oxidation (M)
248	137 – 143	466.1902	930.3659	930.3649	0.0010	0	29	0.0021	1 Score > 13 indicates **identity** Score > 13 indicates **homology**	U	R.MTLPCMY.D + Oxidation (M)
260	137 – 143	474.1870	946.3594	946.3598	-0.0005	0	22	0.01	1 Score > 13 indicates **identity** Score > 13 indicates **homology**	U	R.MTLPCMY.D + 2 Oxidation (M)
702	137 – 147	739.7942	1477.5738	1477.5710	0.0028	1	28	0.0032	1 Score > 13 indicates **identity** Score > 13 indicates **homology**	U	R.MTLPCMYDQCK.H + 2 Oxidation (M)

Query	Start – End	Observed	Mr(expt)	Mr(calc)	Delta	M	Score	Expect	Rank	U	Peptide
445	144 – 151	555.7413	1109.4680	1109.4634	0.0046	2	29	0.01	1 Score > 13 indicates **identity**	U	Y.DQCKHMLY.V + Oxidation (M)
354	159 – 166	515.7400	1029.4655	1029.4655	0.0000	2	20	0.014	1 Score > 13 indicates **identity** Score > 13 indicates **homology**	U	R.LQVSYEEY.L
65	174 – 180	372.2380	742.4615	742.4589	0.0026	2	40	0.00018	1 Score > 13 indicates **identity**	U	L.LLSSVPK.D
186	181 – 188	445.2367	888.4589	888.4552	0.0036	2	26	0.018	1 Score > 13 indicates **identity**	U	K.DGLKSQEL.F
461	185 – 193	568.6526	1135.2907	1135.5509	-0.2602	2	27	0.014	1 Score > 13 indicates **identity**	U	K.SQELFDEIR.M
462	185 – 193	568.7820	1135.5494	1135.5509	-0.0015	2	45	0.00021	1 Score > 13 indicates **identity**	U	K.SQELFDEIR.M
196	194 – 200	449.2090	896.4034	896.4677	-0.0643	2	13	0.3	2 Score > 13 indicates **identity**	U	R.MTYIKEL.G
413	207 – 215	539.2397	1076.4649	1076.4635	0.0014	1	25	0.008	1 Score > 13 indicates **identity** Score > 13 indicates **homology**	U	K.REGNSSQNW.Q
227	208 – 215	461.1900	920.3655	920.3624	0.0031	0	27	0.0027	1 Score > 13 indicates **identity** Score > 13 indicates **homology**	U	R.EGNSSQNW.Q
516	208 – 217	603.2689	1204.5233	1204.5221	0.0012	1	63	2.5e-06	1 Score > 13 indicates **identity** Score > 13 indicates **homology**	U	R.EGNSSQNWQR.F
572	226 – 236	643.3076	1284.6007	1284.6020	-0.0013	1	15	0.15	1 Score > 13 indicates **identity** Score > 13 indicates **homology**	U	L.DSMHEVVENLL.N
585	226 – 236	651.3080	1300.6015	1300.5969	0.0046	1	21	0.065	1 Score > 13 indicates **identity** Score > 13 indicates **homology**	U	L.DSMHEVVENLL.N + Oxidation (M)
773	226 – 238	781.8593	1561.7041	1561.7082	-0.0042	2	35	0.0017	1 Score > 13 indicates **identity**	U	L.DSMHEVVENLLNY.C
780	226 – 238	789.8564	1577.6983	1577.7032	-0.0048	2	17	0.03	1 Score > 13 indicates **identity** Score > 13 indicates **homology**	U	L.DSMHEVVENLLNY.C + Oxidation (M)
769	244 – 256	777.3836	1552.7527	1552.7517	0.0011	2	52	2.9e-05	1 Score > 13 indicates **identity**	U	F.LDKTMSIEFPEML.A
779	244 – 256	785.3795	1568.7445	1568.7466	-0.0021	2	36	0.0011	1 Score > 13 indicates **identity**	U	F.LDKTMSIEFPEML.A + Oxidation (M)
787	244 – 256	793.3777	1584.7408	1584.7415	-0.0007	2	37	0.00033	1 Score > 13 indicates **identity** Score > 13 indicates **homology**	U	F.LDKTMSIEFPEML.A + 2 Oxidation (M)

Query	Start – End	Observed	Mr(expt)	Mr(calc)	Delta	M	Score	Expect	Rank	U	Peptide
1020	245 – 266	855.4366	2563.2880	2563.2913	-0.0033	2	62	1e-06	1 Score > 13 indicates identity	U	L.DKTMSIEFPEMLAEIITNQIPK.Y + Oxidation (M)
1025	245 – 266	860.7680	2579.2822	2579.2862	-0.0040	2	63	1.1e-06	1 Score > 13 indicates identity	U	L.DKTMSIEFPEMLAEIITNQIPK.Y + 2 Oxidation (M)
509	247 – 256	599.2792	1196.5438	1196.5457	-0.0019	0	25	0.0066	1 Score > 13 indicates identity Score > 13 indicates homology	U	K.TMSIEFPEML.A
523	247 – 256	607.2775	1212.5404	1212.5406	-0.0002	0	26	0.025	1 Score > 13 indicates identity	U	K.TMSIEFPEML.A + Oxidation (M)
994	247 – 266	769.0625	2304.1656	2304.1745	-0.0089	1	78	4.4e-08	1 Score > 13 indicates identity	U	K.TMSIEFPEMLAEIITNQIPK.Y
998	247 – 266	774.3925	2320.1558	2320.1694	-0.0136	1	63	2.1e-06	1 Score > 13 indicates identity	U	K.TMSIEFPEMLAEIITNQIPK.Y + Oxidation (M)
999	247 – 266	774.3934	2320.1585	2320.1694	-0.0109	1	73	1.8e-07	1 Score > 13 indicates identity	U	K.TMSIEFPEMLAEIITNQIPK.Y + Oxidation (M)
1003	247 – 266	779.7259	2336.1559	2336.1643	-0.0085	1	59	2e-06	1 Score > 13 indicates identity	U	K.TMSIEFPEMLAEIITNQIPK.Y + 2 Oxidation (M)
454	257 – 266	563.8281	1125.6416	1125.6393	0.0022	0	54	1.9e-05	1 Score > 13 indicates identity	U	L.AEIITNQIPK.Y
576	257 – 267	645.3577	1288.7009	1288.7027	-0.0018	1	49	7.7e-05	1 Score > 13 indicates identity Score > 13 indicates homology	U	L.AEIITNQIPKY.S

MINK1 trypsin and chymotrypsin digest

Prot0560

Database: UserUKCM

Score: 1842

Monoisotopic mass (M_r): 32690

Calculated pI: 6.83

Sequence similarity is available as an NCBI BLAST search of Prot0560 against nr.

Search parameters

**MS data file:**	D:\SCIEX OS Data\MGF files\May2018\300818 LBD GR MINK1 double.mgf
**Enzyme:**	TrypChymo: cuts C-term side of FKLRWY unless next residue is P.
**Fixed modifications:**	Carbamidomethyl (C)
**Variable modifications:**	Oxidation (M), Phospho (ST), Phospho (Y)

Protein sequence coverage: 72%

Matched peptides shown in ***bold red***.

```
  1 MHHHHHHGGE NLYFQGNPGN KTIVPATLPQ LTPTLVSLLE VIEPEVLYAG
 51 YDSSVPDSTW RIMTTLNMLG GRQVIAAVKW AKAIPGFRNL HLDDQMTLLQ
101 YSWMSLMAFA LGWRSYRQSS ANLLCFAPDL IINEQRMTLP CMYDQCKHML
151 YVSSELHRLQ VSYEEYLCMK TLLLLSSVPK DGLKSQELFD EIRMTYIKEL
201 GKAIVKREGN SSQNWQRFYQ LTKLLDSMHE VVENLLNYCF QTFLDKTMSI
251 EFPEMLAEII TNQIPKYSNG NIKKLLFHQK
```

Unformatted sequence string: 280 residues (for pasting into other applications).

Query	Start – End	Observed	Mr(expt)	Mr(calc)	Delta	M	Score	Expect	Rank	U	Peptide
508	13 – 21	512.7480	1023.4814	1023.4774	0.0040	2	49	7.8e-05	1 Score > 21 indicates **identity**	U	L.YFQGNPGNK.T
794	39 – 48	602.3288	1202.6430	1202.6434	-0.0005	2	21	0.012	1 Score > 21 indicates **identity** Score > 14 indicates **homology**	U	L.LEVIEPEVLY.A
336	40 – 47	464.2553	926.4960	926.4960	0.0000	0	36	0.0018	1 Score > 21 indicates **identity**	U	L.EVIEPEVL.Y
632	40 – 48	545.7865	1089.5585	1089.5594	-0.0009	1	40	0.00061	1 Score > 21 indicates **identity**	U	L.EVIEPEVLY.A

Query	Start – End	Observed	Mr(expt)	Mr(calc)	Delta	M	Score	Expect	Rank	U	Peptide
1059	48 – 60	724.3027	1446.5909	1446.5939	-0.0031	2	25	0.013	1 Score **> 19** indicates **identity**	U	L.YAGYDSSVPDSTW.R
879	49 – 60	642.7732	1283.5318	1283.5306	0.0012	1	30	0.0014	1 Score **> 20** indicates **identity** Score **> 14** indicates **homology**	U	Y.AGYDSSVPDSTW.R
1047	49 – 61	720.8228	1439.6310	1439.6317	-0.0008	2	42	0.00021	1 Score **> 21** indicates **identity** Score **> 18** indicates **homology**	U	Y.AGYDSSVPDSTWR.I
1048	49 – 61	720.8228	1439.6310	1439.6317	-0.0007	2	55	9.3e-06	1 Score **> 21** indicates **identity** Score **> 17** indicates **homology**	U	Y.AGYDSSVPDSTWR.I
465	52 – 60	497.2108	992.4071	992.4087	-0.0016	0	23	0.0094	1 Score **> 21** indicates **identity** Score **> 15** indicates **homology**	U	Y.DSSVPDSTW.R
466	52 – 60	497.2115	992.4085	992.4087	-0.0002	0	22	0.032	1 Score **> 21** indicates **identity** Score **> 19** indicates **homology**	U	Y.DSSVPDSTW.R
715	52 – 61	575.2631	1148.5117	1148.5098	0.0019	1	48	3.8e-05	1 Score **> 22** indicates **identity** Score **> 16** indicates **homology**	U	Y.DSSVPDSTWR.I
716	52 – 61	575.2633	1148.5121	1148.5098	0.0023	1	49	3e-05	1 Score **> 22** indicates **identity** Score **> 17** indicates **homology**	U	Y.DSSVPDSTWR.I
753	61 – 69	586.7820	1171.5494	1171.5494	0.0000	2	28	0.012	1 Score **> 21** indicates **identity**	U	W.RIMTTLNML.G + Phospho (ST)
799	62 – 72	603.8196	1205.6247	1205.6260	-0.0013	2	32	0.0033	1 Score **> 20** indicates **identity** Score **> 19** indicates **homology**	U	R.IMTTLNMLGGR.Q
818	62 – 72	611.8144	1221.6142	1221.6210	-0.0067	2	64	2.2e-06	1 Score **> 20** indicates **identity**	U	R.IMTTLNMLGGR.Q + Oxidation (M)

Query	Start	–	End	Observed	Mr(expt)	Mr(calc)	Delta	M	Score	Expect	Rank	U	Peptide
832	62	-	72	619.8169	1237.6192	1237.6159	0.0033	2	33	0.0034	1 Score **> 20** indicates **identity**	U	R.IMTTLNMLGGR.Q + 2 Oxidation (M)
883	62	-	72	643.8042	1285.5939	1285.5924	0.0016	2	70	6.5e-07	1 Score **> 20** indicates **identity**	U	R.IMTTLNMLGGR.Q + Phospho (ST)
904	62	-	72	651.7990	1301.5834	1301.5873	-0.0038	2	62	2e-06	1 Score **> 21** indicates **identity** Score **> 18** indicates **homology**	U	R.IMTTLNMLGGR.Q + Oxidation (M); Phospho (ST)
905	62	-	72	651.7996	1301.5847	1301.5873	-0.0026	2	48	3.5e-05	1 Score **> 21** indicates **identity** Score **> 16** indicates **homology**	U	R.IMTTLNMLGGR.Q + Oxidation (M); Phospho (ST)
925	62	-	72	659.7994	1317.5843	1317.5822	0.0021	2	42	0.00015	1 Score **> 21** indicates **identity** Score **> 16** indicates **homology**	U	R.IMTTLNMLGGR.Q + 2 Oxidation (M); Phospho (ST)
72	73	-	79	364.7376	727.4607	727.4592	0.0015	0	41	7.9e-05	1 Score **> 13** indicates **identity**	U	R.QVIAAVK.W
73	73	-	79	364.7388	727.4631	727.4592	0.0039	0	43	5e-05	1 Score **> 13** indicates **identity**	U	R.QVIAAVK.W
309	73	-	80	457.7768	913.5391	913.5385	0.0006	1	48	8.7e-05	1 Score **> 20** indicates **identity**	U	R.QVIAAVKW.A
310	73	-	80	457.7776	913.5406	913.5385	0.0020	1	61	4.8e-06	1 Score **> 20** indicates **identity**	U	R.QVIAAVKW.A
213	81	-	88	430.2630	858.5114	858.5076	0.0039	2	28	0.0067	1 Score **> 19** indicates **identity**	U	W.AKAIPGFR.N
788	89	-	98	600.2871	1198.5597	1198.5652	-0.0055	2	36	0.0018	1 Score **> 21** indicates **identity**	U	R.NLHLDDQMTL.L
811	89	-	98	608.2866	1214.5587	1214.5601	-0.0015	2	33	0.0031	1 Score **> 21** indicates **identity** Score **> 21** indicates **homology**	U	R.NLHLDDQMTL.L + Oxidation (M)
812	89	-	98	608.2877	1214.5608	1214.5601	0.0007	2	37	0.0016	1 Score **> 21** indicates **identity**	U	R.NLHLDDQMTL.L + Oxidation (M)
461	91	-	98	494.7237	987.4329	987.4332	-0.0002	1	33	0.0034	1 Score **> 21** indicates **identity**	U	L.HLDDQMTL.L + Oxidation (M)

Query	Start - End	Observed	Mr(expt)	Mr(calc)	Delta	M	Score	Expect	Rank	U	Peptide
624	91 - 99	543.2675	1084.5205	1084.5223	-0.0018	2	43	0.00039	1 Score > 21 indicates identity	U	L.HLDDQMTLL.Q
645	91 - 99	551.2652	1100.5159	1100.5172	-0.0013	2	46	0.00013	1 Score > 21 indicates identity Score > 20 indicates homology	U	L.HLDDQMTLL.Q + Oxidation (M)
460	102 - 109	494.7171	987.4196	987.4194	0.0002	2	34	0.0031	1 Score > 21 indicates identity	U	Y.SWMSLMAF.A + Oxidation (M)
135	107 - 113	398.1947	794.3749	794.3785	-0.0036	2	29	0.0022	1 Score > 15 indicates identity	U	L.MAFALGW.R
150	107 - 113	406.1948	810.3750	810.3734	0.0016	2	43	0.00018	1 Score > 18 indicates identity	U	L.MAFALGW.R + Oxidation (M)
260	117 - 124	444.7495	887.4844	887.4825	0.0019	2	48	7.5e-05	1 Score > 19 indicates identity	U	Y.RQSSANLL.C
261	117 - 124	444.7504	887.4863	887.4825	0.0038	2	26	0.013	2 Score > 19 indicates identity	U	Y.RQSSANLL.C
76	118 - 124	366.6986	731.3826	731.3813	0.0013	1	22	0.015	1 Score > 16 indicates identity	U	R.QSSANLL.C
1109	125 - 136	492.5792	1474.7158	1474.7238	-0.0081	2	22	0.026	1 Score > 20 indicates identity Score > 19 indicates homology	U	L.CFAPDLIINEQR.M
1110	125 - 136	492.5810	1474.7210	1474.7238	-0.0028	2	58	9.3e-06	1 Score > 20 indicates identity	U	L.CFAPDLIINEQR.M
747	127 - 136	584.8192	1167.6238	1167.6248	-0.0010	1	28	0.0025	1 Score > 21 indicates identity Score > 14 indicates homology	U	F.APDLIINEQR.M
748	127 - 136	584.8197	1167.6248	1167.6248	0.0000	1	61	4.1e-06	1 Score > 21 indicates identity Score > 20 indicates homology	U	F.APDLIINEQR.M
749	127 - 136	584.8207	1167.6269	1167.6248	0.0021	1	67	8.4e-07	1 Score > 21 indicates identity Score > 19 indicates homology	U	F.APDLIINEQR.M
311	137 - 143	458.1918	914.3691	914.3700	-0.0009	0	22	0.0093	1 Score > 21 indicates identity	U	R.MTLPCMY.D

Query	Start – End	Observed	Mr (expt)	Mr (calc)	Delta	M	Score	Expect	Rank	U	Peptide
									Score > 15 indicates **homology**		
348	137 – 143	466.1894	930.3642	930.3649	-0.0007	0	31	0.0072	1Score > 22 indicates **identity** Score > 22 indicates **homology**	U	R.MTLPCMY.D + Oxidation (M)
349	137 – 143	466.1904	930.3662	930.3649	0.0013	0	29	0.0021	1Score > 22 indicates **identity** Score > 15 indicates **homology**	U	R.MTLPCMY.D + Oxidation (M)
377	137 – 143	474.1860	946.3574	946.3598	-0.0024	0	32	0.001	1Score > 22 indicates **identity** Score > 15 indicates **homology**	U	R.MTLPCMY.D + 2 Oxidation (M)
1058	137 – 147	723.7967	1445.5788	1445.5811	-0.0024	1	45	0.00014	1Score > 19 indicates **identity**	U	R.MTLPCMYDQCK.H
1090	137 – 147	731.7947	1461.5748	1461.5761	-0.0013	1	65	1.7e-06	1Score > 20 indicates **identity**	U	R.MTLPCMYDQCK.H + Oxidation (M)
1115	137 – 147	739.7956	1477.5766	1477.5710	0.0056	1	57	6.1e-06	1Score > 22 indicates **identity** Score > 17 indicates **homology**	U	R.MTLPCMYDQCK.H + 2 Oxidation (M)
659	144 – 151	555.7407	1109.4668	1109.4634	0.0034	2	28	0.012	1Score > 21 indicates **identity**	U	Y.DQCKHMLY.V + Oxidation (M)
463	151 – 158	495.7538	989.4931	989.4930	0.0001	2	50	7.5e-05	1Score > 21 indicates **identity**	U	L.YVSSELHR.L
171	152 – 158	414.2232	826.4318	826.4297	0.0021	1	31	0.0026	1Score > 17 indicates **identity**	U	Y.VSSELHR.L
520	159 – 166	515.7390	1029.4635	1029.4655	-0.0019	2	24	0.0098	1Score > 22 indicates **identity** Score > 17 indicates **homology**	U	R.LQVSYEEY.L
419	164 – 170	486.7113	971.4081	971.4092	-0.0011	2	35	0.00086	1Score > 21 indicates **identity** Score > 17 indicates **homology**	U	Y.EEYLCMK.T
86	174 – 180	372.2380	742.4615	742.4589	0.0026	2	45	6.2e-05	1Score > 15 indicates **identity**	U	L.LLSSVPK.D

Query	Start	-	End	Observed	Mr(expt)	Mr(calc)	Delta	M	Score	Expect	Rank	U	Peptide
694	185	-	193	568.7828	1135.5511	1135.5509	0.0001	2	46	0.00019	1 Score > 21 indicates identity	U	K.SQELFDEIR.M
695	185	-	193	568.7833	1135.5521	1135.5509	0.0012	2	41	0.00051	1 Score > 21 indicates identity	U	K.SQELFDEIR.M
613	207	-	215	539.2397	1076.4648	1076.4635	0.0013	1	42	0.00028	1 Score > 23 indicates identity Score > 19 indicates homology	U	K.REGNSSQNW.Q
322	208	-	215	461.1903	920.3660	920.3624	0.0036	0	23	0.0063	1 Score > 21 indicates identity Score > 14 indicates homology	U	R.EGNSSQNW.Q
796	208	-	217	603.2689	1204.5232	1204.5221	0.0011	1	63	2.7e-06	1 Score > 20 indicates identity	U	R.EGNSSQNWQR.F
1005	224	-	235	699.8478	1397.6810	1397.6860	-0.0051	2	37	0.00091	1 Score > 21 indicates identity Score > 19 indicates homology	U	K.LLDSMHEVVENL.L
1017	224	-	235	707.8468	1413.6790	1413.6810	-0.0020	2	42	0.00037	1 Score > 20 indicates identity	U	K.LLDSMHEVVENL.L + Oxidation (M)
1240	226	-	238	781.8584	1561.7022	1561.7082	-0.0060	2	46	0.00015	1 Score > 20 indicates identity	U	L.DSMHEVVENLLNY.C
1257	226	-	238	789.8586	1577.7026	1577.7032	-0.0005	2	33	0.00096	1 Score > 20 indicates identity Score > 15 indicates homology	U	L.DSMHEVVENLLNY.C + Oxidation (M)
1226	244	-	256	777.3812	1552.7479	1552.7517	-0.0038	2	44	0.00018	1 Score > 19 indicates identity	U	F.LDKTMSIEFPEML.A
1250	244	-	256	785.3794	1568.7443	1568.7466	-0.0023	2	36	0.001	1 Score > 19 indicates identity	U	F.LDKTMSIEFPEML.A + Oxidation (M)
1264	244	-	256	793.3740	1584.7334	1584.7415	-0.0082	2	22	0.035	1 Score > 20 indicates identity	U	F.LDKTMSIEFPEML.A + 2 Oxidation (M)
1719	245	-	266	850.1032	2547.2878	2547.2964	-0.0086	2	40	0.0003	1 Score > 17 indicates identity	U	L.DKTMSIEFPEMLAEIITNQIPK.Y
1720	245	-	266	855.4339	2563.2798	2563.2913	-0.0116	2	68	2.7e-07	1 Score > 15 indicates identity	U	L.DKTMSIEFPEMLAEIITNQIPK.Y + Oxidation (M)

Query	Start – End	Observed	Mr(expt)	Mr(calc)	Delta	M	Score	Expect	Rank	U	Peptide
1723	245 – 266	860.7687	2579.2844	2579.2862	-0.0019	2	56	4.4e-06	1 Score **> 15** indicates **identity**	U	L.DKTMSIEFPEMLAEIITNQIPK.Y + 2 Oxidation (M)
807	247 – 256	607.2752	1212.5359	1212.5406	-0.0047	0	22	0.048	1 Score **> 23** indicates **identity** Score **> 21** indicates **homology**	U	K.TMSIEFPEML.A + Oxidation (M)
1671	247 – 266	769.0621	2304.1644	2304.1745	-0.0101	1	63	1.3e-06	1 Score **> 17** indicates **identity**	U	K.TMSIEFPEMLAEIITNQIPK.Y
1672	247 – 266	769.0628	2304.1665	2304.1745	-0.0080	1	37	0.00054	1 Score **> 17** indicates **identity**	U	K.TMSIEFPEMLAEIITNQIPK.Y
1678	247 – 266	774.3952	2320.1638	2320.1694	-0.0056	1	63	1.8e-06	1 Score **> 18** indicates **identity**	U	K.TMSIEFPEMLAEIITNQIPK.Y + Oxidation (M)
1681	247 – 266	779.7256	2336.1550	2336.1643	-0.0093	1	29	0.002	1 Score **> 14** indicates **identity**	U	K.TMSIEFPEMLAEIITNQIPK.Y + 2 Oxidation (M)
1682	247 – 266	779.7263	2336.1570	2336.1643	-0.0073	1	54	5.2e-06	1 Score **> 14** indicates **identity**	U	K.TMSIEFPEMLAEIITNQIPK.Y + 2 Oxidation (M)
1706	247 – 267	823.4176	2467.2310	2467.2378	-0.0068	2	44	0.00011	1 Score **> 17** indicates **identity**	U	K.TMSIEFPEMLAEIITNQIPKY.S
1710	247 – 267	828.7490	2483.2250	2483.2327	-0.0077	2	29	0.0027	1 Score **> 16** indicates **identity**	U	K.TMSIEFPEMLAEIITNQIPKY.S + Oxidation (M)
681	257 – 266	563.8273	1125.6401	1125.6393	0.0007	0	48	8.4e-05	1 Score **> 19** indicates **identity**	U	L.AEIITNQIPK.Y
887	257 – 267	645.3587	1288.7029	1288.7027	0.0002	1	44	0.00022	1 Score **> 20** indicates **identity**	U	L.AEIITNQIPKY.S

MST1 trypsin and chymotrypsin digest

Prot0560

**Database:**	UserUKCM
**Score:**	1720
**Monoisotopic mass (M_r):**	32690
**Calculated pI:**	6.83

Sequence similarity is available as an NCBI BLAST search of Prot0560 against nr.

Search parameters

**MS data file:**	D:\SCIEX OS Data\MGF files\May2018\300818 LBD GR MST1 double.mgf
**Enzyme:**	TrypChymo: cuts C-term side of FKLRWY unless next residue is P.
**Fixed modifications:**	Carbamidomethyl (C)
**Variable modifications:**	Oxidation (M), Phospho (ST), Phospho (Y)

Protein sequence coverage: 65%

Matched peptides shown in ***bold red***.

```
  1 MHHHHHHGGE NLYFQGNPGN KTIVPATLPQ LTPTLVSLLE VIEPEVLYAG
 51 YDSSVPDSTW RIMTTLNMLG GRQVIAAVKW AKAIPGFRNL HLDDQMTLLQ
101 YSWMSLMAFA LGWRSYRQSS ANLLCFAPDL IINEQRMTLP CMYDQCKHML
151 YVSSELHRLQ VSYEEYLCMK TLLLLSSVPK DGLKSQELFD EIRMTYIKEL
201 GKAIVKREGN SSQNWQRFYQ LTKLLDSMHE VVENLLNYCF QTFLDKTMSI
```

251 **EFPEMLAEII** **TNQIPKY**SNG NIKKLLFHQK

Unformatted sequence string: [280 residues](#) (for pasting into other applications).

Query	Start	-	End	Observed	Mr(expt)	Mr(calc)	Delta	M	Score	Expect	Rank	U	Peptide
718	39	-	48	602.3276	1202.6407	1202.6434	-0.0028	2	70	7.9e-07	1 Score > **21** indicates **identity**	U	L.LEVIEPEVLY.A
295	40	-	47	464.2545	926.4945	926.4960	-0.0016	0	33	0.0033	1 Score > **21** indicates **identity**	U	L.EVIEPEVL.Y
554	40	-	48	545.7851	1089.5557	1089.5594	-0.0037	1	40	0.00063	1 Score > **21** indicates **identity**	U	L.EVIEPEVLY.A
806	49	-	60	642.7710	1283.5274	1283.5306	-0.0032	1	17	0.023	1 Score > **20** indicates **identity** Score > **14** indicates **homology**	U	Y.AGYDSSVPDSTW.R
982	49	-	61	720.8225	1439.6305	1439.6317	-0.0013	2	58	4.9e-06	1 Score > **21** indicates **identity** Score > **18** indicates **homology**	U	Y.AGYDSSVPDSTWR.I
1104	49	-	61	760.8062	1519.5978	1519.5981	-0.0003	2	47	4.3e-05	1 Score > **21** indicates **identity** Score > **15** indicates **homology**	U	Y.AGYDSSVPDSTWR.I + Phospho (ST)
395	52	-	60	497.2112	992.4079	992.4087	-0.0009	0	15	0.036	1 Score > **21** indicates **identity** Score > **13** indicates **homology**	U	Y.DSSVPDSTW.R
396	52	-	60	497.2117	992.4088	992.4087	0.0001	0	15	0.035	1 Score > **21** indicates **identity** Score > **13** indicates **homology**	U	Y.DSSVPDSTW.R
636	52	-	61	575.2655	1148.5165	1148.5098	0.0067	1	45	0.00027	1 Score > **22** indicates **identity**	U	Y.DSSVPDSTWR.I
673	61	-	69	586.7805	1171.5465	1171.5494	-0.0029	2	34	0.0028	1 Score > **21** indicates **identity**	U	W.RIMTTLNML.G + Phospho (ST)
692	61	-	69	594.7778	1187.5410	1187.5443	-0.0034	2	25	0.024	1 Score > **21** indicates **identity**	U	W.RIMTTLNML.G + Oxidation (M); Phospho (ST)

Query	Start – End	Observed	Mr(expt)	Mr(calc)	Delta	M	Score	Expect	Rank	U	Peptide
693	61 – 69	594.7778	1187.5411	1187.5443	-0.0033	2	33	0.0037	1 Score **>** **21** indicates **identity**	U	W.RIMTTLNML.G + Oxidation (M); Phospho (ST)
719	61 – 69	602.7756	1203.5367	1203.5393	-0.0026	2	41	0.00042	1 Score **>** **20** indicates **identity**	U	W.RIMTTLNML.G + 2 Oxidation (M); Phospho (ST)
424	62 – 69	508.7288	1015.4431	1015.4483	-0.0053	1	27	0.014	1 Score **>** **21** indicates **identity**	U	R.IMTTLNML.G + Phospho (ST)
453	62 – 69	516.7270	1031.4394	1031.4432	-0.0038	1	21	0.017	1 Score **>** **22** indicates **identity** Score **>** **16** indicates **homology**	U	R.IMTTLNML.G + Oxidation (M); Phospho (ST)
723	62 – 72	603.8197	1205.6248	1205.6260	-0.0013	2	71	3.8e-07	1 Score **>** **20** indicates **identity**	U	R.IMTTLNMLGGR.Q
737	62 – 72	611.8170	1221.6195	1221.6210	-0.0015	2	64	2.5e-06	1 Score **>** **20** indicates **identity** Score **>** **20** indicates **homology**	U	R.IMTTLNMLGGR.Q + Oxidation (M)
759	62 – 72	619.8157	1237.6169	1237.6159	0.0010	2	78	1.1e-07	1 Score **>** **20** indicates **identity**	U	R.IMTTLNMLGGR.Q + 2 Oxidation (M)
810	62 – 72	643.8024	1285.5903	1285.5924	-0.0020	2	65	1.3e-06	1 Score **>** **20** indicates **identity** Score **>** **19** indicates **homology**	U	R.IMTTLNMLGGR.Q + Phospho (ST)
830	62 – 72	651.7999	1301.5852	1301.5873	-0.0021	2	34	0.0019	1 Score **>** **21** indicates **identity** Score **>** **19** indicates **homology**	U	R.IMTTLNMLGGR.Q + Oxidation (M); Phospho (ST)
831	62 – 72	651.8002	1301.5858	1301.5873	-0.0015	2	50	3.4e-05	1 Score **>** **21** indicates **identity** Score **>** **18** indicates **homology**	U	R.IMTTLNMLGGR.Q + Oxidation (M); Phospho (ST)
847	62 – 72	659.7987	1317.5829	1317.5822	0.0007	2	47	3.6e-05	1 Score **>** **21** indicates **identity** Score **>** **15** indicates **homology**	U	R.IMTTLNMLGGR.Q + 2 Oxidation (M); Phospho (ST)
61	73 – 79	364.7374	727.4603	727.4592	0.0011	0	43	5e-05	1 Score **>** **13** indicates **identity**	U	R.QVIAAVK.W

Query	Start – End	Observed	Mr (expt)	Mr (calc)	Delta	M	Score	Expect	Rank	U	Peptide
271	73 – 80	457.7762	913.5378	913.5385	-0.0008	1	58	1e-05	1 Score > 20 indicates **identity**	U	R.QVIAAVKW.A
41	81 – 87	352.2094	702.4042	702.4064	-0.0022	1	26	0.0024	1 Score > 13 indicates **identity**	U	W.AKAIPGF.R
183	81 – 88	430.2627	858.5108	858.5076	0.0032	2	26	0.011	1 Score > 19 indicates **identity**	U	W.AKAIPGFR.N
712	89 – 98	600.2889	1198.5633	1198.5652	-0.0019	2	44	0.00014	1 Score > 21 indicates **identity** Score > 18 indicates **homology**	U	R.NLHLDDQMTL.L
732	89 – 98	608.2861	1214.5577	1214.5601	-0.0025	2	45	0.00023	1 Score > 21 indicates **identity**	U	R.NLHLDDQMTL.L + Oxidation (M)
362	91 – 98	486.7262	971.4378	971.4382	-0.0005	1	36	0.0018	1 Score > 21 indicates **identity**	U	L.HLDDQMTL.L
391	91 – 98	494.7237	987.4328	987.4332	-0.0003	1	39	0.00094	1 Score > 21 indicates **identity**	U	L.HLDDQMTL.L + Oxidation (M)
547	91 – 99	543.2653	1084.5160	1084.5223	-0.0062	2	34	0.0033	1 Score > 21 indicates **identity**	U	L.HLDDQMTLL.Q
562	91 – 99	551.2649	1100.5153	1100.5172	-0.0019	2	36	0.0017	1 Score > 21 indicates **identity**	U	L.HLDDQMTLL.Q + Oxidation (M)
408	102 – 109	502.7136	1003.4127	1003.4143	-0.0016	2	49	6.4e-05	1 Score > 22 indicates **identity** Score > 19 indicates **homology**	U	Y.SWMSLMAF.A + 2 Oxidation (M)
111	107 – 113	398.1931	794.3717	794.3785	-0.0068	2	36	0.00049	1 Score > 15 indicates **identity**	U	L.MAFALGW.R
123	107 – 113	406.1931	810.3716	810.3734	-0.0019	2	33	0.0015	1 Score > 18 indicates **identity**	U	L.MAFALGW.R + Oxidation (M)
228	117 – 124	444.7491	887.4836	887.4825	0.0011	2	43	0.00023	1 Score > 19 indicates **identity**	U	Y.RQSSANLL.C
64	118 – 124	366.6989	731.3833	731.3813	0.0019	1	21	0.019	1 Score > 16 indicates **identity**	U	R.QSSANLL.C
1039	125 – 136	738.3664	1474.7183	1474.7238	-0.0056	2	83	2.3e-08	1 Score > 20 indicates **identity** Score > 19 indicates **homology**	U	L.CFAPDLIINEQR.M

Query	Start	–	End	Observed	Mr(expt)	Mr(calc)	Delta	M	Score	Expect	Rank		U	Peptide
663	127	–	136	584.8190	1167.6235	1167.6248	-0.0013	1	73	1.7e-07	1	Score > 21 indicates identity Score > 18 indicates homology	U	F.APDLIINEQR.M
664	127	–	136	584.8195	1167.6245	1167.6248	-0.0002	1	72	1.9e-07	1	Score > 21 indicates identity Score > 17 indicates homology	U	F.APDLIINEQR.M
272	137	–	143	458.1912	914.3679	914.3700	-0.0021	0	18	0.022	1	Score > 21 indicates identity Score > 14 indicates homology	U	R.MTLPCMY.D
305	137	–	143	466.1899	930.3652	930.3649	0.0003	0	31	0.0068	1	Score > 22 indicates identity Score > 22 indicates homology	U	R.MTLPCMY.D + Oxidation (M)
306	137	–	143	466.1902	930.3659	930.3649	0.0010	0	39	0.00032	1	Score > 22 indicates identity Score > 16 indicates homology	U	R.MTLPCMY.D + Oxidation (M)
326	137	–	143	474.1860	946.3574	946.3598	-0.0025	0	31	0.0011	1	Score > 22 indicates identity Score > 14 indicates homology	U	R.MTLPCMY.D + 2 Oxidation (M)
398	137	–	143	498.1742	994.3339	994.3363	-0.0024	0	20	0.022	1	Score > 22 indicates identity Score > 16 indicates homology	U	R.MTLPCMY.D + Phospho (ST)
416	137	–	143	506.1710	1010.3274	1010.3313	-0.0038	0	18	0.029	1	Score > 22 indicates identity Score > 15 indicates homology	U	R.MTLPCMY.D + Oxidation (M); Phospho (ST)
580	144	–	151	555.7399	1109.4653	1109.4634	0.0019	2	30	0.0057	1	Score > 21 indicates identity Score > 20 indicates homology	U	Y.DQCKHMLY.V + Oxidation (M)
251	157	–	163	451.7476	901.4806	901.4770	0.0036	2	31	0.0042	1	Score > 20 indicates identity	U	L.HRLQVSY.E
449	159	–	166	515.7392	1029.4638	1029.4655	-0.0017	2	31	0.0029	1	Score > 22 indicates identity	U	R.LQVSYEEY.L

Query	Start – End	Observed	Mr(expt)	Mr(calc)	Delta	M	Score	Expect	Rank	U	Peptide
									Score > 18 indicates homology		
73	174 – 180	372.2367	742.4588	742.4589	-0.0001	2	46	4.3e-05	1 Score > 15 indicates identity	U	L.LLSSVPK.D
615	185 – 193	568.7822	1135.5499	1135.5509	-0.0010	2	52	4.6e-05	1 Score > 21 indicates identity	U	K.SQELFDEIR.M
616	185 – 193	568.7827	1135.5508	1135.5509	-0.0001	2	42	0.00048	1 Score > 21 indicates identity	U	K.SQELFDEIR.M
532	207 – 215	539.2397	1076.4648	1076.4635	0.0013	1	31	0.0078	1 Score > 23 indicates identity	U	K.REGNSSQNW.Q
278	208 – 215	461.1889	920.3632	920.3624	0.0008	0	30	0.0016	1 Score > 21 indicates identity Score > 15 indicates homology	U	R.EGNSSQNW.Q
720	208 – 217	603.2717	1204.5289	1204.5221	0.0068	1	64	2.3e-06	1 Score > 20 indicates identity	U	R.EGNSSQNWQR.F
807	208 – 217	643.2519	1284.4892	1284.4884	0.0008	1	24	0.0055	1 Score > 20 indicates identity Score > 14 indicates homology	U	R.EGNSSQNWQR.F + Phospho (ST)
932	224 – 235	699.8494	1397.6842	1397.6860	-0.0019	2	31	0.002	1 Score > 21 indicates identity Score > 16 indicates homology	U	K.LLDSMHEVVENL.L
808	226 – 236	643.3078	1284.6010	1284.6020	-0.0010	1	18	0.02	1 Score > 20 indicates identity Score > 14 indicates homology	U	L.DSMHEVVENLL.N
1154	226 – 238	781.8578	1561.7011	1561.7082	-0.0071	2	54	2.5e-05	1 Score > 20 indicates identity	U	L.DSMHEVVENLLNY.C
1172	226 – 238	789.8573	1577.7000	1577.7032	-0.0032	2	34	0.0013	1 Score > 20 indicates identity Score > 18 indicates homology	U	L.DSMHEVVENLLNY.C + Oxidation (M)
1146	244 – 256	777.3820	1552.7494	1552.7517	-0.0023	2	22	0.028	1 Score > 19 indicates identity	U	F.LDKTMSIEFPEML.A

Query	Start – End	Observed	Mr(expt)	Mr(calc)	Delta	M	Score	Expect	Rank	U	Peptide
1166	244 – 256	785.3778	1568.7411	1568.7466	-0.0055	2	36	0.001	1 Score **> 19** indicates **identity**	U	F.LDKTMSIEFPEML.A + Oxidation (M)
1178	244 – 256	793.3762	1584.7379	1584.7415	-0.0036	2	31	0.0013	1 Score **> 20** indicates **identity** Score **> 14** indicates **homology**	U	F.LDKTMSIEFPEML.A + 2 Oxidation (M)
1248	244 – 256	825.3638	1648.7130	1648.7129	0.0001	2	20	0.043	1 Score **> 19** indicates **identity**	U	F.LDKTMSIEFPEML.A + Oxidation (M); Phospho (ST)
1006	245 – 256	728.8394	1455.6643	1455.6625	0.0017	1	31	0.0041	1 Score **> 20** indicates **identity** Score **> 20** indicates **homology**	U	L.DKTMSIEFPEML.A + Oxidation (M)
1607	245 – 266	855.4362	2563.2868	2563.2913	-0.0045	2	57	3.3e-06	1 Score **> 15** indicates **identity**	U	L.DKTMSIEFPEMLAEIITNQIPK.Y + Oxidation (M)
1614	245 – 266	860.7660	2579.2762	2579.2862	-0.0100	2	28	0.003	1 Score **> 15** indicates **identity**	U	L.DKTMSIEFPEMLAEIITNQIPK.Y + 2 Oxidation (M)
705	247 – 256	599.2779	1196.5413	1196.5457	-0.0044	0	29	0.002	1 Score **> 21** indicates **identity** Score **> 14** indicates **homology**	U	K.TMSIEFPEML.A
728	247 – 256	607.2768	1212.5390	1212.5406	-0.0016	0	35	0.0016	1 Score **> 23** indicates **identity** Score **> 20** indicates **homology**	U	K.TMSIEFPEML.A + Oxidation (M)
1546	247 – 266	769.0642	2304.1706	2304.1745	-0.0039	1	46	7.1e-05	1 Score **> 17** indicates **identity**	U	K.TMSIEFPEMLAEIITNQIPK.Y
1554	247 – 266	774.3929	2320.1569	2320.1694	-0.0125	1	48	6.2e-05	1 Score **> 18** indicates **identity**	U	K.TMSIEFPEMLAEIITNQIPK.Y + Oxidation (M)
1555	247 – 266	774.3930	2320.1571	2320.1694	-0.0123	1	75	1.1e-07	1 Score **> 18** indicates **identity**	U	K.TMSIEFPEMLAEIITNQIPK.Y + Oxidation (M)
1562	247 – 266	779.7246	2336.1520	2336.1643	-0.0124	1	68	2.3e-07	1 Score **> 14** indicates **identity**	U	K.TMSIEFPEMLAEIITNQIPK.Y + 2 Oxidation (M)
1589	247 – 267	823.4191	2467.2356	2467.2378	-0.0023	2	35	0.00078	1 Score **> 17** indicates **identity**	U	K.TMSIEFPEMLAEIITNQIPKY.S
606	257 – 266	563.8276	1125.6406	1125.6393	0.0013	0	46	0.00012	1 Score **> 19** indicates **identity**	U	L.AEIITNQIPK.Y

Query	Start – End	Observed	Mr (expt)	Mr (calc)	Delta	M	Score	Expect	Rank	U	Peptide
814	257 – 267	645.3574	1288.7003	1288.7027	-0.0023	1	56	6e-06	1	U	L.AEIITNQIPKY.S

Score > **20** indicates **identity**
Score > **16** indicates **homology**

MST2 trypsin and chymotrypsin digest

Prot0560

**Database:**	UserUKCM
**Score:**	1580
**Monoisotopic mass (M_r):**	32690
**Calculated pI:**	6.83

Sequence similarity is available as an NCBI BLAST search of Prot0560 against nr.

Search parameters

**MS data file:**	D:\SCIEX OS Data\MGF files\May2018\300818 LBD GR MST2 double.mgf
**Enzyme:**	TrypChymo: cuts C-term side of FKLRWY unless next residue is P.
**Fixed modifications:**	Carbamidomethyl (C)
**Variable modifications:**	Oxidation (M), Phospho (ST), Phospho (Y)

Protein sequence coverage: 65%

Matched peptides shown in ***bold red***.

1 MHHHHHHGGE NLYFQGNPGN KTIVPATLPQ LTPTLVSL**LE** **VIEPEVLYAG**

51 **YDSSVPDSTW** **RIMTTLNMLG** **GRQVIAAVKW** **AKAIPGFRNL** **HLDDQMTLL**Q

101 Y**SWMSLMAFA** **LGW**RSY**RQSS** **ANLLCFAPDL** **IINEQRMTLP** **CMYDQCKHML**

151 **Y**VSSELHR**LQ** **VSYEEY**LCMK TLL**LLSSVPK** DGLK**SQELFD** **EIRMTY**IKEL

201 GKAIVK**REGN** **SSQNWQR**FYQ LTKLL**DSMHE** **VVENLLNY**CF QTF**LDKTMSI**

251 **EFPEMLAEII** **TNQIPKY**SNG NIKKLLFHQK

Unformatted sequence string: 280 residues (for pasting into other applications).

Query	Start	–	End	Observed	Mr(expt)	Mr(calc)	Delta	M	Score	Expect	Rank	U	Peptide
575	39	–	48	602.3293	1202.6440	1202.6434	0.0006	2	28	0.013	1 Score > 21 indicates **identity**	U	L.LEVIEPEVLY.A
252	40	–	47	464.2558	926.4971	926.4960	0.0011	0	30	0.0073	1 Score > 21 indicates **identity**	U	L.EVIEPEVL.Y
464	40	–	48	545.7859	1089.5573	1089.5594	-0.0021	1	41	0.0006	1 Score > 21 indicates **identity**	U	L.EVIEPEVLY.A
779	48	–	60	724.3035	1446.5924	1446.5939	-0.0015	2	21	0.031	1 Score > 19 indicates **identity**	U	L.YAGYDSSVPDSTW.R
641	49	–	60	642.7735	1283.5325	1283.5306	0.0019	1	17	0.024	1 Score > 20 indicates **identity** Score > 14 indicates **homology**	U	Y.AGYDSSVPDSTW.R
768	49	–	61	720.8241	1439.6336	1439.6317	0.0019	2	63	1.9e-06	1 Score > 21 indicates **identity** Score > 18 indicates **homology**	U	Y.AGYDSSVPDSTWR.I
869	49	–	61	760.8048	1519.5950	1519.5981	-0.0030	2	45	5.5e-05	1 Score > 21 indicates **identity** Score > 15 indicates **homology**	U	Y.AGYDSSVPDSTWR.I + Phospho (ST)
339	52	–	60	497.2126	992.4106	992.4087	0.0019	0	23	0.032	1 Score > 21 indicates **identity** Score > 21 indicates **homology**	U	Y.DSSVPDSTW.R
516	52	–	61	575.2631	1148.5117	1148.5098	0.0019	1	45	5.8e-05	1 Score > 22 indicates **identity**	U	Y.DSSVPDSTWR.I

Query	Start – End	Observed	Mr(expt)	Mr(calc)	Delta	M	Score	Expect	Rank	U	Peptide
									Score > **15** indicates **homology**		
542	61 – 69	586.7814	1171.5482	1171.5494	-0.0013	2	37	0.0014	1 Score > **21** indicates **identity**	U	W.RIMTTLNML.G + Phospho (ST)
555	61 – 69	594.7791	1187.5437	1187.5443	-0.0006	2	30	0.0062	1 Score > **21** indicates **identity** Score > **20** indicates **homology**	U	W.RIMTTLNML.G + Oxidation (M); Phospho (ST)
580	62 – 72	603.8210	1205.6275	1205.6260	0.0015	2	33	0.0024	1 Score > **20** indicates **identity**	U	R.IMTTLNMLGGR.Q
593	62 – 72	611.8184	1221.6223	1221.6210	0.0013	2	30	0.0021	1 Score > **20** indicates **identity** Score > **15** indicates **homology**	U	R.IMTTLNMLGGR.Q + Oxidation (M)
594	62 – 72	611.8187	1221.6228	1221.6210	0.0019	2	51	4.9e-05	1 Score > **20** indicates **identity**	U	R.IMTTLNMLGGR.Q + Oxidation (M)
603	62 – 72	619.8157	1237.6169	1237.6159	0.0010	2	43	9.5e-05	1 Score > **20** indicates **identity** Score > **15** indicates **homology**	U	R.IMTTLNMLGGR.Q + 2 Oxidation (M)
644	62 – 72	643.8041	1285.5937	1285.5924	0.0014	2	57	7.5e-06	1 Score > **20** indicates **identity** Score > **19** indicates **homology**	U	R.IMTTLNMLGGR.Q + Phospho (ST)
656	62 – 72	651.8011	1301.5877	1301.5873	0.0004	2	50	2.3e-05	1 Score > **21** indicates **identity** Score > **16** indicates **homology**	U	R.IMTTLNMLGGR.Q + Oxidation (M); Phospho (ST)
657	62 – 72	651.8017	1301.5888	1301.5873	0.0015	2	69	3.7e-07	1 Score > **21** indicates **identity** Score > **17** indicates **homology**	U	R.IMTTLNMLGGR.Q + Oxidation (M); Phospho (ST)
671	62 – 72	659.7988	1317.5831	1317.5822	0.0009	2	40	0.00016	1 Score > **21** indicates **identity** Score > **15** indicates **homology**	U	R.IMTTLNMLGGR.Q + 2 Oxidation (M); Phospho (ST)
54	73 – 79	364.7375	727.4604	727.4592	0.0012	0	41	7.3e-05	1 Score > **13** indicates **identity**	U	R.QVIAAVK.W

Query	Start	–	End	Observed	Mr(expt)	Mr(calc)	Delta	M	Score	Expect	Rank	U	Peptide
232	73	–	80	457.7776	913.5406	913.5385	0.0021	1	61	4.8e-06	1 Score > 20 indicates identity	U	R.QVIAAVKW.A
165	81	–	88	430.2616	858.5086	858.5076	0.0010	2	27	0.0086	1 Score > 19 indicates identity	U	W.AKAIPGFR.N
570	89	–	98	600.2906	1198.5667	1198.5652	0.0014	2	40	0.00075	1 Score > 21 indicates identity	U	R.NLHLDDQMTL.L
590	89	–	98	608.2878	1214.5611	1214.5601	0.0009	2	30	0.0038	1 Score > 21 indicates identity Score > 18 indicates homology	U	R.NLHLDDQMTL.L + Oxidation (M)
308	91	–	98	486.7271	971.4397	971.4382	0.0015	1	42	0.00039	1 Score > 21 indicates identity	U	L.HLDDQMTL.L
337	91	–	98	494.7259	987.4373	987.4332	0.0041	1	42	0.00042	1 Score > 21 indicates identity	U	L.HLDDQMTL.L + Oxidation (M)
456	91	–	99	543.2684	1084.5223	1084.5223	0.0000	2	32	0.0052	1 Score > 21 indicates identity	U	L.HLDDQMTLL.Q
351	102	–	109	502.7141	1003.4137	1003.4143	-0.0007	2	40	0.00079	1 Score > 22 indicates identity Score > 22 indicates homology	U	Y.SWMSLMAF.A + 2 Oxidation (M)
96	107	–	113	398.1968	794.3790	794.3785	0.0005	2	30	0.0017	1 Score > 15 indicates identity	U	L.MAFALGW.R
107	107	–	113	406.1939	810.3733	810.3734	-0.0001	2	37	0.00067	1 Score > 18 indicates identity	U	L.MAFALGW.R + Oxidation (M)
195	117	–	124	444.7491	887.4836	887.4825	0.0012	2	49	5.9e-05	1 Score > 19 indicates identity	U	Y.RQSSANLL.C
304	117	–	124	484.7324	967.4502	967.4488	0.0014	2	24	0.021	1 Score > 20 indicates identity	U	Y.RQSSANLL.C + Phospho (ST)
819	125	–	136	492.5818	1474.7235	1474.7238	-0.0004	2	39	0.00037	1 Score > 20 indicates identity Score > 18 indicates homology	U	L.CFAPDLIINEQR.M
536	127	–	136	584.8206	1167.6267	1167.6248	0.0019	1	41	0.00016	1 Score > 21 indicates identity Score > 16 indicates homology	U	F.APDLIINEQR.M
537	127	–	136	584.8217	1167.6288	1167.6248	0.0040	1	66	8.7e-07	1 Score > 21 indicates	U	F.APDLIINEQR.M

Query	Start – End	Observed	Mr(expt)	Mr(calc)	Delta	M	Score	Expect	Rank	U	Peptide
									identity Score > 18 indicates homology		
233	137 – 143	458.1930	914.3715	914.3700	0.0015	0	21	0.028	1Score > 21 indicates identity Score > 18 indicates homology	U	R.MTLPCMY.D
263	137 – 143	466.1903	930.3660	930.3649	0.0011	0	31	0.0074	1Score > 22 indicates identity Score > 22 indicates homology	U	R.MTLPCMY.D + Oxidation (M)
264	137 – 143	466.1906	930.3666	930.3649	0.0017	0	33	0.00075	1Score > 22 indicates identity Score > 15 indicates homology	U	R.MTLPCMY.D + Oxidation (M)
282	137 – 143	474.1872	946.3598	946.3598	0.0000	0	22	0.0091	1Score > 22 indicates identity Score > 14 indicates homology	U	R.MTLPCMY.D + 2 Oxidation (M)
342	137 – 143	498.1760	994.3375	994.3363	0.0011	0	34	0.0029	1Score > 22 indicates identity	U	R.MTLPCMY.D + Phospho (ST)
802	137 – 147	731.7948	1461.5751	1461.5761	-0.0010	1	38	0.001	1Score > 20 indicates identity	U	R.MTLPCMYDQCK.H + Oxidation (M)
823	137 – 147	739.7900	1477.5654	1477.5710	-0.0056	1	38	0.00042	1Score > 22 indicates identity Score > 17 indicates homology	U	R.MTLPCMYDQCK.H + 2 Oxidation (M)
477	144 – 151	555.7411	1109.4677	1109.4634	0.0043	2	32	0.0052	1Score > 21 indicates identity	U	Y.DQCKHMLY.V + Oxidation (M)
382	159 – 166	515.7419	1029.4692	1029.4655	0.0037	2	15	0.035	1Score > 22 indicates identity Score > 13 indicates homology	U	R.LQVSYEEY.L
63	174 – 180	372.2374	742.4603	742.4589	0.0014	2	44	7.5e-05	1Score > 15 indicates identity	U	L.LLSSVPK.D
503	185 – 193	568.7839	1135.5532	1135.5509	0.0022	2	44	0.00028	1Score > 21 indicates identity	U	K.SQELFDEIR.M

Query	Start	–	End	Observed	Mr(expt)	Mr(calc)	Delta	M	Score	Expect	Rank	U	Peptide
504	185	–	193	568.7852	1135.5559	1135.5509	0.0050	2	47	0.00014	1 Score > 21 indicates **identity**	U	K.SQELFDEIR.M
277	190	–	196	472.2128	942.4111	942.4117	-0.0006	1	21	0.039	1 Score > 20 indicates **identity**	U	F.DEIRMTY.I + Oxidation (M)
447	207	–	215	539.2380	1076.4615	1076.4635	-0.0020	1	31	0.0025	1 Score > 23 indicates **identity** Score > 18 indicates **homology**	U	K.REGNSSQNW.Q
240	208	–	215	461.1897	920.3649	920.3624	0.0025	0	25	0.0047	1 Score > 21 indicates **identity** Score > 14 indicates **homology**	U	R.EGNSSQNW.Q
577	208	–	217	603.2700	1204.5254	1204.5221	0.0033	1	63	2.8e-06	1 Score > 20 indicates **identity**	U	R.EGNSSQNWQR.F
643	226	–	236	643.3088	1284.6030	1284.6020	0.0010	1	22	0.019	1 Score > 20 indicates **identity** Score > 18 indicates **homology**	U	L.DSMHEVVENLL.N
654	226	–	236	651.3078	1300.6011	1300.5969	0.0042	1	22	0.025	1 Score > 21 indicates **identity** Score > 18 indicates **homology**	U	L.DSMHEVVENLL.N + Oxidation (M)
906	226	–	238	781.8559	1561.6973	1561.7082	-0.0110	2	42	0.00041	1 Score > 20 indicates **identity**	U	L.DSMHEVVENLLNY.C
907	226	–	238	781.8632	1561.7119	1561.7082	0.0036	2	66	1.7e-06	1 Score > 20 indicates **identity**	U	L.DSMHEVVENLLNY.C
918	226	–	238	789.8558	1577.6970	1577.7032	-0.0061	2	49	5.3e-05	1 Score > 20 indicates **identity** Score > 19 indicates **homology**	U	L.DSMHEVVENLLNY.C + Oxidation (M)
898	244	–	256	777.3823	1552.7500	1552.7517	-0.0017	2	38	0.00067	1 Score > 19 indicates **identity**	U	F.LDKTMSIEFPEML.A
914	244	–	256	785.3797	1568.7448	1568.7466	-0.0018	2	40	0.00048	1 Score > 19 indicates **identity**	U	F.LDKTMSIEFPEML.A + Oxidation (M)
924	244	–	256	793.3766	1584.7386	1584.7415	-0.0030	2	26	0.01	1 Score > 20 indicates **identity** Score > 19 indicates **homology**	U	F.LDKTMSIEFPEML.A + 2 Oxidation (M)

Query	Start – End	Observed	Mr(expt)	Mr(calc)	Delta	M	Score	Expect	Rank	U	Peptide
1231	245 – 266	855.4345	2563.2816	2563.2913	-0.0097	2	44	6.6e-05	1 Score **> 15** indicates **identity**	U	L.DKTMSIEFPEMLAEIITNQIPK.Y + Oxidation (M)
1234	245 – 266	860.7673	2579.2802	2579.2862	-0.0060	2	54	6.9e-06	1 Score **> 15** indicates **identity**	U	L.DKTMSIEFPEMLAEIITNQIPK.Y + 2 Oxidation (M)
564	247 – 256	599.2792	1196.5438	1196.5457	-0.0019	0	26	0.0058	1 Score **> 21** indicates **identity** Score **> 17** indicates **homology**	U	K.TMSIEFPEML.A
586	247 – 256	607.2767	1212.5389	1212.5406	-0.0017	0	35	0.0011	1 Score **> 23** indicates **identity** Score **> 18** indicates **homology**	U	K.TMSIEFPEML.A + Oxidation (M)
1190	247 – 266	769.0606	2304.1601	2304.1745	-0.0144	1	60	2.6e-06	1 Score **> 17** indicates **identity**	U	K.TMSIEFPEMLAEIITNQIPK.Y
1191	247 – 266	769.0617	2304.1634	2304.1745	-0.0111	1	50	2.8e-05	1 Score **> 17** indicates **identity**	U	K.TMSIEFPEMLAEIITNQIPK.Y
1195	247 – 266	774.3961	2320.1666	2320.1694	-0.0028	1	60	4.1e-06	1 Score **> 18** indicates **identity**	U	K.TMSIEFPEMLAEIITNQIPK.Y + Oxidation (M)
1196	247 – 266	774.3965	2320.1676	2320.1694	-0.0018	1	71	2.8e-07	1 Score **> 18** indicates **identity**	U	K.TMSIEFPEMLAEIITNQIPK.Y + Oxidation (M)
1201	247 – 266	779.7263	2336.1572	2336.1643	-0.0072	1	64	5.3e-07	1 Score **> 14** indicates **identity**	U	K.TMSIEFPEMLAEIITNQIPK.Y + 2 Oxidation (M)
1223	247 – 267	823.4168	2467.2286	2467.2378	-0.0093	2	56	5.9e-06	1 Score **> 17** indicates **identity**	U	K.TMSIEFPEMLAEIITNQIPKY.S
494	257 – 266	563.8278	1125.6410	1125.6393	0.0016	0	48	7.6e-05	1 Score **> 19** indicates **identity**	U	L.AEIITNQIPK.Y
646	257 – 267	645.3588	1288.7031	1288.7027	0.0005	1	40	0.00027	1 Score **> 20** indicates **identity** Score **> 17** indicates **homology**	U	L.AEIITNQIPKY.S

Rock1 trypsin and chymotrypsin digest

Prot0560

**Database:**	UserUKCM
**Score:**	1622
**Monoisotopic mass (M_r):**	32690
**Calculated pI:**	6.83

Sequence similarity is available as an NCBI BLAST search of Prot0560 against nr.

Search parameters

**MS data file:**	D:\SCIEX OS Data\MGF files\May2018\300818 LBD GR Rock1 double.mgf
**Enzyme:**	TrypChymo: cuts C-term side of FKLRWY unless next residue is P.
**Fixed modifications:**	Carbamidomethyl (C)
**Variable modifications:**	Oxidation (M), Phospho (ST), Phospho (Y)

Protein sequence coverage: 68%

Matched peptides shown in ***bold red***.

```
  1 MHHHHHHGGE NLYFQGNPGN KTIVPATLPQ LTPTLVSLLE VIEPEVLYAG
 51 YDSSVPDSTW RIMTTLNMLG GRQVIAAVKW AKAIPGFRNL HLDDQMTLLQ
101 YSWMSLMAFA LGWRSYRQSS ANLLCFAPDL IINEQRMTLP CMYDQCKHML
151 YVSSELHRLQ VSYEEYLCMK TLLLLSSVPK DGLKSQELFD EIRMTYIKEL
201 GKAIVKREGN SSQNWQRFYQ LTKLLDSMHE VVENLLNYCF QTFLDKTMSI
```

251 **EFPEMLAEII** **TNQIPKY**SNG NIKKLLFHQK

Unformatted sequence string: 280 residues (for pasting into other applications).

Query	Start	-	End	Observed	Mr(expt)	Mr(calc)	Delta	M	Score	Expect	Rank		U	Peptide
358	13	-	21	512.7455	1023.4764	1023.4774	-0.0010	2	33	0.0033	1	Score > 21 indicates identity	U	L.YFQGNPGNK.T
518	39	-	48	602.3299	1202.6452	1202.6434	0.0018	2	36	0.0017	1	Score > 21 indicates identity	U	L.LEVIEPEVLY.A
247	40	-	47	464.2559	926.4972	926.4960	0.0011	0	42	0.00048	1	Score > 21 indicates identity	U	L.EVIEPEVL.Y
439	40	-	48	545.7874	1089.5603	1089.5594	0.0009	1	40	0.00062	1	Score > 21 indicates identity	U	L.EVIEPEVLY.A
620	40	-	51	691.3472	1380.6798	1380.6813	-0.0015	2	48	0.00013	1	Score > 22 indicates identity	U	L.EVIEPEVLYAGY.D
568	49	-	60	642.7742	1283.5337	1283.5306	0.0031	1	31	0.0013	1	Score > 20 indicates identity Score > 14 indicates homology	U	Y.AGYDSSVPDSTW.R
650	49	-	61	720.8229	1439.6313	1439.6317	-0.0004	2	47	6.1e-05	1	Score > 21 indicates identity Score > 17 indicates homology	U	Y.AGYDSSVPDSTWR.I
331	52	-	60	497.2135	992.4125	992.4087	0.0038	0	22	0.02	1	Score > 21 indicates identity Score > 17 indicates homology	U	Y.DSSVPDSTW.R
478	52	-	61	575.2625	1148.5104	1148.5098	0.0006	1	51	1.8e-05	1	Score > 22 indicates identity Score > 17 indicates homology	U	Y.DSSVPDSTWR.I
479	52	-	61	575.2629	1148.5112	1148.5098	0.0014	1	50	2e-05	1	Score > 22 indicates identity Score > 16 indicates homology	U	Y.DSSVPDSTWR.I
278	62	-	69	476.7455	951.4764	951.4769	-0.0006	1	27	0.015	1	Score > 22 indicates identity Score > 22 indicates homology	U	R.IMTTLNML.G + Oxidation (M)
299	62	-	69	484.7434	967.4723	967.4718	0.0005	1	22	0.034	1	Score > 20 indicates identity	U	R.IMTTLNML.G + 2 Oxidation (M)
350	62	-	69	508.7829	1015.5512	1015.4483	0.1029	1	23	0.032	1	Score > 21 indicates identity	U	R.IMTTLNML.G + Phospho (ST)
522	62	-	72	603.8211	1205.6277	1205.6260	0.0017	2	56	1.2e-05	1	Score > 20 indicates identity	U	R.IMTTLNMLGGR.Q
531	62	-	72	611.8185	1221.6224	1221.6210	0.0015	2	71	4.9e-07	1	Score > 20 indicates identity	U	R.IMTTLNMLGGR.Q + Oxidation (M)

Query	Start – End	Observed	Mr(expt)	Mr(calc)	Delta	M	Score	Expect	Rank	U	Peptide
532	62 – 72	611.8187	1221.6228	1221.6210	0.0019	2	56	1.6e-05	1 Score > **20** indicates **identity**	U	R.IMTTLNMLGGR.Q + Oxidation (M)
538	62 – 72	619.8161	1237.6176	1237.6159	0.0017	2	66	1.4e-06	1 Score > **20** indicates **identity**	U	R.IMTTLNMLGGR.Q + 2 Oxidation (M)
54	73 – 79	364.7398	727.4650	727.4592	0.0057	0	43	5.1e-05	1 Score > **13** indicates **identity**	U	R.QVIAAVK.W
232	73 – 80	457.7766	913.5386	913.5385	0.0001	1	54	2.2e-05	1 Score > **20** indicates **identity**	U	R.QVIAAVKW.A
161	81 – 88	430.2627	858.5108	858.5076	0.0033	2	31	0.0036	1 Score > **19** indicates **identity**	U	W.AKAIPGFR.N
516	89 – 98	600.2905	1198.5664	1198.5652	0.0012	2	37	0.0009	1 Score > **21** indicates **identity** Score > **19** indicates **homology**	U	R.NLHLDDQMTL.L
529	89 – 98	608.2868	1214.5590	1214.5601	-0.0011	2	37	0.00053	1 Score > **21** indicates **identity** Score > **17** indicates **homology**	U	R.NLHLDDQMTL.L + Oxidation (M)
304	91 – 98	486.7272	971.4398	971.4382	0.0016	1	35	0.0021	1 Score > **21** indicates **identity**	U	L.HLDDQMTL.L
329	91 – 98	494.7254	987.4362	987.4332	0.0030	1	38	0.0012	1 Score > **21** indicates **identity**	U	L.HLDDQMTL.L + Oxidation (M)
434	91 – 99	543.2683	1084.5220	1084.5223	-0.0003	2	34	0.0029	1 Score > **21** indicates **identity**	U	L.HLDDQMTLL.Q
303	102 – 109	486.7199	971.4252	971.4245	0.0008	2	27	0.0031	1 Score > **21** indicates **identity** Score > **14** indicates **homology**	U	Y.SWMSLMAF.A
328	102 – 109	494.7173	987.4200	987.4194	0.0006	2	31	0.0059	1 Score > **21** indicates **identity**	U	Y.SWMSLMAF.A + Oxidation (M)
340	102 – 109	502.7152	1003.4159	1003.4143	0.0016	2	37	0.00087	1 Score > **22** indicates **identity** Score > **19** indicates **homology**	U	Y.SWMSLMAF.A + 2 Oxidation (M)
100	107 – 113	398.1968	794.3791	794.3785	0.0005	2	41	0.00014	1 Score > **15** indicates **identity**	U	L.MAFALGW.R
109	107 – 113	406.1939	810.3733	810.3734	-0.0002	2	35	0.00096	1 Score > **18** indicates **identity**	U	L.MAFALGW.R + Oxidation (M)
196	117 – 124	444.7484	887.4823	887.4825	-0.0001	2	41	0.0004	1 Score > **19** indicates **identity**	U	Y.RQSSANLL.C
298	117 – 124	484.7324	967.4502	967.4488	0.0014	2	21	0.046	1 Score > **20** indicates **identity**	U	Y.RQSSANLL.C + Phospho (ST)
56	118 – 124	366.6985	731.3825	731.3813	0.0012	1	19	0.033	1 Score > **16** indicates **identity**	U	R.QSSANLL.C
684	125 – 136	492.5818	1474.7237	1474.7238	-0.0001	2	46	6.2e-05	1 Score > **20** indicates **identity**	U	L.CFAPDLIINEQR.M

Query	Start	-	End	Observed	Mr(expt)	Mr(calc)	Delta	M	Score	Expect	Rank	U	Peptide
											Score > 17 indicates homology		
497	127	-	136	584.8204	1167.6263	1167.6248	0.0016	1	47	3.6e-05	1Score > 21 indicates identity Score > 15 indicates homology	U	F.APDLIINEQR.M
498	127	-	136	584.8209	1167.6272	1167.6248	0.0024	1	57	5e-06	1Score > 21 indicates identity Score > 17 indicates homology	U	F.APDLIINEQR.M
233	137	-	143	458.1937	914.3729	914.3700	0.0028	0	20	0.014	1Score > 21 indicates identity Score > 14 indicates homology	U	R.MTLPCMY.D
257	137	-	143	466.1902	930.3658	930.3649	0.0009	0	31	0.002	1Score > 22 indicates identity Score > 16 indicates homology	U	R.MTLPCMY.D + Oxidation (M)
258	137	-	143	466.1907	930.3669	930.3649	0.0020	0	26	0.019	1Score > 22 indicates identity Score > 21 indicates homology	U	R.MTLPCMY.D + Oxidation (M)
272	137	-	143	474.1883	946.3621	946.3598	0.0022	0	25	0.005	1Score > 22 indicates identity Score > 14 indicates homology	U	R.MTLPCMY.D + 2 Oxidation (M)
656	137	-	147	723.7979	1445.5812	1445.5811	0.0001	1	56	1.1e-05	1Score > 19 indicates identity	U	R.MTLPCMYDQCK.H
673	137	-	147	731.7971	1461.5797	1461.5761	0.0036	1	56	1.5e-05	1Score > 20 indicates identity	U	R.MTLPCMYDQCK.H + Oxidation (M)
688	137	-	147	739.7939	1477.5732	1477.5710	0.0022	1	52	5.5e-05	1Score > 22 indicates identity	U	R.MTLPCMYDQCK.H + 2 Oxidation (M)
450	144	-	151	555.7402	1109.4659	1109.4634	0.0025	2	25	0.025	1Score > 21 indicates identity	U	Y.DQCKHMLY.V + Oxidation (M)
366	159	-	166	515.7412	1029.4678	1029.4655	0.0023	2	25	0.0049	1Score > 22 indicates identity Score > 14 indicates homology	U	R.LQVSYEEY.L
65	174	-	180	372.2367	742.4589	742.4589	0.0000	2	44	7.6e-05	1Score > 15 indicates identity	U	L.LLSSVPK.D
467	185	-	193	568.6515	1135.2885	1135.5509	-0.2625	2	22	0.04	1Score > 21 indicates identity	U	K.SQELFDEIR.M
468	185	-	193	568.7821	1135.5497	1135.5509	-0.0012	2	56	1.9e-05	1Score > 21 indicates identity	U	K.SQELFDEIR.M
437	189	-	196	545.7477	1089.4808	1089.4801	0.0007	2	24	0.016	1Score > 21 indicates identity Score > 19 indicates homology	U	L.FDEIRMTY.I + Oxidation (M)
269	190	-	196	472.2142	942.4138	942.4117	0.0022	1	27	0.0071	1Score > 20 indicates identity	U	F.DEIRMTY.I + Oxidation (M)

Query	Start	–	End	Observed	Mr(expt)	Mr(calc)	Delta	M	Score	Expect	Rank	U	Peptide
											Score **> 18** indicates **homology**		
428	207	–	215	539.2403	1076.4661	1076.4635	0.0026	1	29	0.0048	1Score **> 23** indicates **identity** Score **> 19** indicates **homology**	U	K.REGNSSQNW.Q
238	208	–	215	461.1894	920.3642	920.3624	0.0018	0	25	0.0042	1Score **> 21** indicates **identity** Score **> 14** indicates **homology**	U	R.EGNSSQNW.Q
519	208	–	217	603.2678	1204.5210	1204.5221	-0.0011	1	63	2.7e-06	1Score **> 20** indicates **identity**	U	R.EGNSSQNWQR.F
606	208	–	218	676.8041	1351.5936	1351.5905	0.0031	2	44	7.1e-05	1Score **> 20** indicates **identity** Score **> 15** indicates **homology**	U	R.EGNSSQNWQRF.Y
747	226	–	238	781.8563	1561.6981	1561.7082	-0.0101	2	38	0.00099	1Score **> 20** indicates **identity**	U	L.DSMHEVVENLLNY.C
748	226	–	238	781.8570	1561.6994	1561.7082	-0.0088	2	23	0.031	1Score **> 20** indicates **identity**	U	L.DSMHEVVENLLNY.C
755	226	–	238	789.8602	1577.7059	1577.7032	0.0028	2	46	0.00013	1Score **> 20** indicates **identity**	U	L.DSMHEVVENLLNY.C + Oxidation (M)
743	244	–	256	777.3835	1552.7525	1552.7517	0.0009	2	29	0.0057	1Score **> 19** indicates **identity**	U	F.LDKTMSIEFPEML.A
753	244	–	256	785.3797	1568.7448	1568.7466	-0.0018	2	23	0.022	1Score **> 19** indicates **identity**	U	F.LDKTMSIEFPEML.A + Oxidation (M)
762	244	–	256	793.3775	1584.7405	1584.7415	-0.0010	2	45	0.00018	1Score **> 20** indicates **identity** Score **> 20** indicates **homology**	U	F.LDKTMSIEFPEML.A + 2 Oxidation (M)
973	245	–	266	855.4355	2563.2848	2563.2913	-0.0066	2	67	3.3e-07	1Score **> 15** indicates **identity**	U	L.DKTMSIEFPEMLAEIITNQIPK.Y + Oxidation (M)
974	245	–	266	860.7665	2579.2778	2579.2862	-0.0085	2	89	2.4e-09	1Score **> 15** indicates **identity**	U	L.DKTMSIEFPEMLAEIITNQIPK.Y + 2 Oxidation (M)
513	247	–	256	599.2785	1196.5425	1196.5457	-0.0032	0	25	0.0043	1Score **> 21** indicates **identity** Score **> 14** indicates **homology**	U	K.TMSIEFPEML.A
527	247	–	256	607.2771	1212.5396	1212.5406	-0.0010	0	21	0.023	1Score **> 23** indicates **identity** Score **> 17** indicates **homology**	U	K.TMSIEFPEML.A + Oxidation (M)
950	247	–	266	769.0654	2304.1743	2304.1745	-0.0002	1	59	3.4e-06	1Score **> 17** indicates **identity**	U	K.TMSIEFPEMLAEIITNQIPK.Y
954	247	–	266	774.3965	2320.1676	2320.1694	-0.0018	1	61	3.1e-06	1Score **> 18** indicates **identity**	U	K.TMSIEFPEMLAEIITNQIPK.Y + Oxidation (M)
955	247	–	266	774.3978	2320.1715	2320.1694	0.0021	1	75	1.4e-07	1Score **> 18** indicates **identity**	U	K.TMSIEFPEMLAEIITNQIPK.Y + Oxidation (M)

Query	Start	–	End	Observed	Mr(expt)	Mr(calc)	Delta	M	Score	Expect	Rank	U	Peptide
960	247	–	266	779.7288	2336.1645	2336.1643	0.0001	1	74	6.5e-08	1 Score > 14 indicates identity	U	K.TMSIEFPEMLAEIITNQIPK.Y
971	247	–	267	823.4170	2467.2292	2467.2378	-0.0087	2	38	0.00038	1 Score > 17 indicates identity	U	+ 2 Oxidation (M) K.TMSIEFPEMLAEIITNQIPKY.S
461	257	–	266	563.8287	1125.6429	1125.6393	0.0035	0	56	1.1e-05	1 Score > 19 indicates identity	U	L.AEIITNQIPK.Y
574	257	–	267	645.3596	1288.7047	1288.7027	0.0020	1	44	0.00013	1 Score > 20 indicates identity Score > 17 indicates homology	U	L.AEIITNQIPKY.S

2.2.4. Chymotrypsin and trypsin digestion and data analysis using the SwissProtdata base.

MAP4K4 trypsin and chymotrypsin digest

Glucocorticoid receptor OS=Homo sapiens OX=9606 GN=NR3C1 PE=1 SV=1

**Database:**	SwissProt
**Score:**	687
**Monoisotopic mass (M_r):**	86745
**Calculated pI:**	6.00
**Taxonomy:**	Homo sapiens

Sequence similarity is available as an NCBI BLAST search of GCR_HUMAN against nr.

Search parameters

**MS data file:**	D:\SCIEX OS Data\MGF files\May2018\300818 LBD GR MAP4K4 double.mgf
**Enzyme:**	TrypChymo: cuts C-term side of FKLRWY unless next residue is P.

Fixed modifications: Carbamidomethyl (C)

Variable modifications: Oxidation (M), Phospho (ST), Phospho (Y)

Protein sequence coverage: 16%

Matched peptides shown in ***bold red***.

```
  1 MDSKESLTPG REENPSSVLA QERGDVMDFY KTLRGGATVK VSASSPSLAV
 51 ASQSDSKQRR LLVDFPKGSV SNAQQPDLSK AVSLSMGLYM GETETKVMGN
101 DLGFPQQGQI SLSSGETDLK LLEESIANLN RSTSVPENPK SSASTAVSAA
151 PTEKEFPKTH SDVSSEQQHL KGQTGTNGGN VKLYTTDQST FDILQDLEFS
201 SGSPGKETNE SPWRSDLLID ENCLLSPLAG EDDSFLLEGN SNEDCKPLIL
251 PDTKPKIKDN GDLVLSSPSN VTLPQVKTEK EDFIELCTPG VIKQEKLGTV
301 YCQASFPGAN IIGNKMSAIS VHGVSTSGGQ MYHYDMNTAS LSQQQDQKPI
351 FNVIPPIPVG SENWNRCQGS GDDNLTSLGT LNFPGRTVFS NGYSSPSMRP
401 DVSSPPSSSS TATTGPPPKL CLVCSDEASG CHYGVLTCGS CKVFFKRAVE
451 GQHNYLCAGR NDCIIDKIRR KNCPACRYRK CLQAGMNLEA RKTKKKIKGI
```

501 QQATTGVSQE TSENPGNKTI VPATLPQLTP TLVSLL**EVIE** **PEVLYAGYDS**

551 **SVPDSTWRIM** **TTLNMLGGRQ** **VIAAVKW**AKA IPGFR**NLHLD** **DQMTLL**QYSW

601 MFLMAFALGW RSYRQSSANL L**CFAPDLIIN** **EQRMTLPCMY** **DQCK**HMLYVS

651 SELHRLQVSY EEYLCMKTLL **LLSSVPK**DGL K**SQELFDEIR** MTYIKELGKA

701 IVKR**EGNSSQ** **NWQR**FYQLTK LLDSMHEVVE NLLNYCFQTF **LDKTMSIEFP**

751 **EMLAEIITNQ** **IPKY**SNGNIK KLLFHQK

Unformatted sequence string: 777 residues (for pasting into other applications).

Query	Start	–	End	Observed	Mr(expt)	Mr(calc)	Delta	M	Score	Expect	Rank		U	Peptide
236	537	–	544	464.2558	926.4971	926.4960	0.0011	0	44	0.49	1	Score **> 40** indicates **identity**	U	L.EVIEPEVL.Y
428	537	–	545	545.7868	1089.5591	1089.5594	-0.0003	1	40	0.53	4	Score **> 40** indicates **identity**	U	L.EVIEPEVLY.A

Query	Start – End	Observed	Mr(expt)	Mr(calc)	Delta	M	Score	Expect	Rank	U	Peptide
									Score > 37 indicates homology		
570	546 - 557	642.7725	1283.5304	1283.5306	-0.0003	1	20	0.42	1 Score > 40 indicates identity Score > 16 indicates homology	U	Y.AGYDSSVPDSTW.R
661	546 - 558	720.8230	1439.6314	1439.6317	-0.0003	2	41	0.019	1 Score > 40 indicates identity Score > 23 indicates homology	U	Y.AGYDSSVPDSTWR.I
475	549 - 558	575.2617	1148.5088	1148.5098	-0.0010	1	47	0.032	1 Score > 40 indicates identity Score > 31 indicates homology	U	Y.DSSVPDSTWR.I
476	549 - 558	575.2630	1148.5114	1148.5098	0.0016	1	59	0.0084	1 Score > 40 indicates identity Score > 37 indicates homology	U	Y.DSSVPDSTWR.I
517	559 - 569	603.8185	1205.6224	1205.6260	-0.0036	2	77	2.6e-05	1 Score > 40 indicates identity Score > 31 indicates homology	U	R.IMTTLNMLGGR.Q
530	559 - 569	611.8178	1221.6210	1221.6210	0.0000	2	54	0.0032	1 Score > 40 indicates identity Score > 28 indicates homology	U	R.IMTTLNMLGGR.Q + Oxidation (M)
539	559 - 569	619.8165	1237.6184	1237.6159	0.0025	2	76	0.00018	1 Score > 40 indicates identity Score > 38 indicates homology	U	R.IMTTLNMLGGR.Q + 2 Oxidation (M)
52	570 - 576	364.7380	727.4615	727.4592	0.0022	0	44	0.082	1 Score > 33 indicates identity	U	R.QVIAAVK.W
223	570 - 577	457.7752	913.5358	913.5385	-0.0027	1	57	0.021	1 Score > 40 indicates identity	U	R.QVIAAVKW.A
512	586 - 595	600.2898	1198.5650	1198.5652	-0.0003	2	39	0.26	1 Score > 40 indicates identity Score > 33 indicates homology	U	R.NLHLDDQMTL.L
289	588 - 595	486.7278	971.4411	971.4382	0.0029	1	36	0.15	1 Score > 40 indicates identity Score > 27 indicates homology	U	L.HLDDQMTL.L
317	588 - 595	494.7254	987.4362	987.4332	0.0031	1	39	0.41	1 Score > 41 indicates identity Score > 35 indicates homology	U	L.HLDDQMTL.L + Oxidation (M)
422	588 - 596	543.2675	1084.5205	1084.5223	-0.0018	2	42	0.69	1 Score > 40 indicates identity Score > 40 indicates homology	U	L.HLDDQMTLL.Q
700	622 - 633	492.5814	1474.7224	1474.7238	-0.0014	2	41	0.043	1 Score > 39 indicates identity Score > 27 indicates homology	U	L.CFAPDLIINEQR.M

Query	Start – End	Observed	Mr(expt)	Mr(calc)	Delta	M	Score	Expect	Rank	U	Peptide
493	624 – 633	584.8205	1167.6264	1167.6248	0.0016	1	50	0.13	1Score **>** **40** indicates **identity**	U	F.APDLIINEQR.M
494	624 – 633	584.8208	1167.6270	1167.6248	0.0022	1	69	0.0017	1Score **>** **40** indicates **identity**	U	F.APDLIINEQR.M
224	634 – 640	458.1919	914.3693	914.3700	-0.0007	0	22	0.66	1Score **>** **40** indicates **identity** Score **>** **20** indicates **homology**	U	R.MTLPCMY.D
247	634 – 640	466.1892	930.3638	930.3649	-0.0012	0	30	0.49	1Score **>** **41** indicates **identity** Score **>** **26** indicates **homology**	U	R.MTLPCMY.D + Oxidation (M)
248	634 – 640	466.1902	930.3659	930.3649	0.0010	0	29	0.41	1Score **>** **41** indicates **identity** Score **>** **24** indicates **homology**	U	R.MTLPCMY.D + Oxidation (M)
702	634 – 644	739.7942	1477.5738	1477.5710	0.0028	1	28	0.42	1Score **>** **39** indicates **identity** Score **>** **24** indicates **homology**	U	R.MTLPCMYDQCK.H + 2 Oxidation (M)
65	671 – 677	372.2380	742.4615	742.4589	0.0026	2	40	0.29	1Score **>** **34** indicates **identity**	U	L.LLSSVPK.D
461	682 – 690	568.6526	1135.2907	1135.5509	-0.2602	2	27	0.32	2Score **>** **40** indicates **identity** Score **>** **21** indicates **homology**	U	K.SQELFDEIR.M
462	682 – 690	568.7820	1135.5494	1135.5509	-0.0015	2	45	0.062	1Score **>** **40** indicates **identity** Score **>** **33** indicates **homology**	U	K.SQELFDEIR.M
227	705 – 712	461.1900	920.3655	920.3624	0.0031	0	27	0.22	1Score **>** **40** indicates **identity** Score **>** **20** indicates **homology**	U	R.EGNSSQNW.Q
516	705 – 714	603.2689	1204.5233	1204.5221	0.0012	1	63	0.0053	1Score **>** **40** indicates **identity**	U	R.EGNSSQNWQR.F
769	741 – 753	777.3836	1552.7527	1552.7517	0.0011	2	52	0.0035	1Score **>** **39** indicates **identity** Score **>** **27** indicates **homology**	U	F.LDKTMSIEFPEML.A
779	741 – 753	785.3795	1568.7445	1568.7466	-0.0021	2	36	0.061	1Score **>** **39** indicates **identity** Score **>** **23** indicates **homology**	U	F.LDKTMSIEFPEML.A + Oxidation (M)
787	741 – 753	793.3777	1584.7408	1584.7415	-0.0007	2	37	0.029	1Score **>** **39** indicates **identity** Score **>** **21** indicates **homology**	U	F.LDKTMSIEFPEML.A + 2 Oxidation (M)
1020	742 – 763	855.4366	2563.2880	2563.2913	-0.0033	2	62	0.0017	1Score **>** **34** indicates **identity**	U	L.DKTMSIEFPEMLAEIITNQIPK.Y + Oxidation (M)
1025	742 – 763	860.7680	2579.2822	2579.2862	-0.0040	2	63	0.0015	1Score **>** **34** indicates **identity**	U	L.DKTMSIEFPEMLAEIITNQIPK.Y + 2 Oxidation (M)

Query	Start	–	End	Observed	Mr(expt)	Mr(calc)	Delta	M	Score	Expect	Rank		U	Peptide
523	744	–	753	607.2775	1212.5404	1212.5406	-0.0002	0	26	0.49	1	Score **> 40** indicates **identity** Score **> 23** indicates **homology**	U	K.TMSIEFPEML.A + Oxidation (M)
994	744	–	763	769.0625	2304.1656	2304.1745	-0.0089	1	78	2.7e-06	1	Score **> 35** indicates **identity** Score **> 21** indicates **homology**	U	K.TMSIEFPEMLAEIITNQIPK.Y
998	744	–	763	774.3925	2320.1558	2320.1694	-0.0136	1	63	0.0007	1	Score **> 35** indicates **identity** Score **> 31** indicates **homology**	U	K.TMSIEFPEMLAEIITNQIPK.Y + Oxidation (M)
999	744	–	763	774.3934	2320.1585	2320.1694	-0.0109	1	73	0.00018	1	Score **> 35** indicates **identity**	U	K.TMSIEFPEMLAEIITNQIPK.Y + Oxidation (M)
1003	744	–	763	779.7259	2336.1559	2336.1643	-0.0085	1	59	0.002	1	Score **> 35** indicates **identity** Score **> 31** indicates **homology**	U	K.TMSIEFPEMLAEIITNQIPK.Y + 2 Oxidation (M)
454	754	–	763	563.8281	1125.6416	1125.6393	0.0022	0	54	0.033	1	Score **> 40** indicates **identity** Score **> 39** indicates **homology**	U	L.AEIITNQIPK.Y
576	754	–	764	645.3577	1288.7009	1288.7027	-0.0018	1	49	0.0026	1	Score **> 40** indicates **identity** Score **> 23** indicates **homology**	U	L.AEIITNQIPKY.S

MINK1 trypsin and chymotrypsin digest

Glucocorticoid receptor OS=Homo sapiens OX=9606 GN=NR3C1 PE=1 SV=1

**Database:**	SwissProt
**Score:**	908
**Monoisotopic mass (M_r):**	86745
**Calculated pI:**	6.00
**Taxonomy:**	Homo sapiens

Sequence similarity is available as an NCBI BLAST search of GCR_HUMAN against nr.

Search parameters

MS data file: D:\SCIEX OS Data\MGF files\May2018\300818 LBD GR MINK1 double.mgf

Enzyme: TrypChymo: cuts C-term side of FKLRWY unless next residue is P.

Fixed modifications: Carbamidomethyl (C)

Variable modifications: Oxidation (M), Phospho (ST), Phospho (Y)

Protein sequence coverage: 22%

Matched peptides shown in ***bold red***.

1 MDSKESLTPG REENPSSVLA QERGDVMDFY KTLRGGATVK VSASSPSLAV

51 ASQSDSKQRR LLVDFPKGSV SNAQQPDLSK AVSLSMGLYM GETETKVMGN

101 DLGFPQQGQI SLSSGETDLK LLEESIANLN RSTSVPENPK SSASTAVSAA

151 PTEKEFPKTH SDVSSEQQHL KGQTGTNGGN VKLYTTDQST FDILQDLEFS

201 SGSPGKETNE SPWRSDLLID ENCLLSPLAG EDDSFLLEGN SNEDCKPLIL

251 PDTKPKIKDN GDLVLSSPSN VTLPQVKTEK EDFIELCTPG VIKQEKLGTV

301 YCQASFPGAN IIGNKMSAIS VHGVSTSGGQ MYHYDMNTAS LSQQQDQKPI

351 FNVIPPIPVG SENWNRCQGS GDDNLTSLGT LNFPGRTVFS NGYSSPSMRP

401 DVSSPPSSSS TATTGPPPKL CLVCSDEASG CHYGVLTCGS CKVFFKRAVE

451 GQHNYLCAGR NDCIIDKIRR KNCPACRYRK CLQAGMNLEA RKTKKKIKGI

501 QQATTGVSQE TSENPGNKTI VPATLPQLTP TLVSL**LEVIE** **PEVLYAGYDS**

551 **SVPDSTWRIM** **TTLNMLGGRQ** **VIAAVKW**AKA IPGFR**NLHLD** **DQMTLL**QYSW

601 MFL**MAFALGW** RSY**RQSSANL** **LCFAPDLIIN** **EQRMTLPCMY** **DQCK**HML**YVS**

651 **SELHRLQVSY** **EEYLCMK**TLL **LLSSVPK**DGL K**SQELFDEIR** MTYIKELGKA

701 IVK**REGNSSQ** **NWQR**FYQLTK **LLDSMHEVVE** **NLLNY**CFQTF **LDKTMSIEFP**

751 **EMLAEIITNQ** **IPKY**SNGNIK KLLFHQK

Unformatted sequence string: 777 residues (for pasting into other applications).

Query	Start	–	End	Observed	Mr (expt)	Mr (calc)	Delta	M	Score	Expect	Rank	U	Peptide
794	536	–	545	602.3288	1202.6430	1202.6434	-0.0005	2	21	0.85	1Score > 40 indicates **identity** Score > 19 indicates **homology**	U	L.LEVIEPEVLY.A
336	537	–	544	464.2553	926.4960	926.4960	0.0000	0	36	0.91	2Score > 40 indicates **identity** Score > 35 indicates **homology**	U	L.EVIEPEVL.Y
632	537	–	545	545.7865	1089.5585	1089.5594	-0.0009	1	40	0.53	4Score > 40 indicates **identity** Score > 37 indicates **homology**	U	L.EVIEPEVLY.A
1059	545	–	557	724.3027	1446.5909	1446.5939	-0.0031	2	25	0.073	1Score > 40 indicates **identity** Score > 13 indicates **homology**	U	L.YAGYDSSVPDSTW.R
879	546	–	557	642.7732	1283.5318	1283.5306	0.0012	1	30	0.19	1Score > 40 indicates **identity** Score > 23 indicates **homology**	U	Y.AGYDSSVPDSTW.R
1047	546	–	558	720.8228	1439.6310	1439.6317	-0.0008	2	42	0.032	1Score > 40 indicates **identity** Score > 27 indicates **homology**	U	Y.AGYDSSVPDSTWR.I
1048	546	–	558	720.8228	1439.6310	1439.6317	-0.0007	2	55	0.017	1Score > 40 indicates **identity** Score > 37 indicates **homology**	U	Y.AGYDSSVPDSTWR.I
715	549	–	558	575.2631	1148.5117	1148.5098	0.0019	1	48	0.049	1Score > 40 indicates **identity** Score > 35 indicates **homology**	U	Y.DSSVPDSTWR.I
716	549	–	558	575.2633	1148.5121	1148.5098	0.0023	1	49	0.031	1Score > 40 indicates **identity** Score > 34 indicates **homology**	U	Y.DSSVPDSTWR.I

Query	Start – End	Observed	Mr(expt)	Mr(calc)	Delta	M	Score	Expect	Rank	U	Peptide
818	559 – 569	611.8144	1221.6142	1221.6210	-0.0067	2	64	0.00017	1 Score > **40** indicates **identity** Score > **26** indicates **homology**	U	R.IMTTLNMLGGR.Q + Oxidation (M)
832	559 – 569	619.8169	1237.6192	1237.6159	0.0033	2	33	0.13	1 Score > **40** indicates **identity** Score > **23** indicates **homology**	U	R.IMTTLNMLGGR.Q + 2 Oxidation (M)
883	559 – 569	643.8042	1285.5939	1285.5924	0.0016	2	70	8.2e-05	1 Score > **40** indicates **identity** Score > **28** indicates **homology**	U	R.IMTTLNMLGGR.Q + Phospho (ST)
904	559 – 569	651.7990	1301.5834	1301.5873	-0.0038	2	62	0.00026	1 Score > **40** indicates **identity** Score > **26** indicates **homology**	U	R.IMTTLNMLGGR.Q + Oxidation (M); Phospho (ST)
905	559 – 569	651.7996	1301.5847	1301.5873	-0.0026	2	48	0.014	1 Score > **40** indicates **identity** Score > **29** indicates **homology**	U	R.IMTTLNMLGGR.Q + Oxidation (M); Phospho (ST)
925	559 – 569	659.7994	1317.5843	1317.5822	0.0021	2	42	0.018	1 Score > **40** indicates **identity** Score > **24** indicates **homology**	U	R.IMTTLNMLGGR.Q + 2 Oxidation (M); Phospho (ST)
72	570 – 576	364.7376	727.4607	727.4592	0.0015	0	41	0.14	1 Score > **33** indicates **identity** Score > **32** indicates **homology**	U	R.QVIAAVK.W
73	570 – 576	364.7388	727.4631	727.4592	0.0039	0	43	0.1	1 Score > **33** indicates **identity**	U	R.QVIAAVK.W
309	570 – 577	457.7768	913.5391	913.5385	0.0006	1	48	0.026	1 Score > **40** indicates **identity** Score > **32** indicates **homology**	U	R.QVIAAVKW.A
310	570 – 577	457.7776	913.5406	913.5385	0.0020	1	61	0.002	1 Score > **40** indicates **identity** Score > **33** indicates **homology**	U	R.QVIAAVKW.A
811	586 – 595	608.2866	1214.5587	1214.5601	-0.0015	2	33	0.56	1 Score > **40** indicates **identity**	U	R.NLHLDDQMTL.L + Oxidation (M)

Query	Start – End	Observed	Mr(expt)	Mr(calc)	Delta	M	Score	Expect	Rank	U	Peptide
									Score > 31 indicates **homology**		
812	586 – 595	608.2877	1214.5608	1214.5601	0.0007	2	37	0.64	1Score > 40 indicates **identity** Score > 34 indicates **homology**	U	R.NLHLDDQMTL.L + Oxidation (M)
461	588 – 595	494.7237	987.4329	987.4332	-0.0002	1	33	0.39	2Score > 41 indicates **identity** Score > 29 indicates **homology**	U	L.HLDDQMTL.L + Oxidation (M)
624	588 – 596	543.2675	1084.5205	1084.5223	-0.0018	2	43	0.61	1Score > 40 indicates **identity** Score > 40 indicates **homology**	U	L.HLDDQMTLL.Q
645	588 – 596	551.2652	1100.5159	1100.5172	-0.0013	2	46	0.28	2Score > 41 indicates **identity**	U	L.HLDDQMTLL.Q + Oxidation (M)
135	604 – 610	398.1947	794.3749	794.3785	-0.0036	2	29	0.69	1Score > 36 indicates **identity** Score > 27 indicates **homology**	U	L.MAFALGW.R
150	604 – 610	406.1948	810.3750	810.3734	0.0016	2	43	0.33	1Score > 37 indicates **identity**	U	L.MAFALGW.R + Oxidation (M)
260	614 – 621	444.7495	887.4844	887.4825	0.0019	2	48	0.18	6Score > 40 indicates **identity**	U	Y.RQSSANLL.C
1110	622 – 633	492.5810	1474.7210	1474.7238	-0.0028	2	58	0.0026	1Score > 39 indicates **identity** Score > 31 indicates **homology**	U	L.CFAPDLIINEQR.M
747	624 – 633	584.8192	1167.6238	1167.6248	-0.0010	1	28	0.46	1Score > 40 indicates **identity** Score > 24 indicates **homology**	U	F.APDLIINEQR.M
748	624 – 633	584.8197	1167.6248	1167.6248	0.0000	1	61	0.0091	1Score > 40 indicates **identity**	U	F.APDLIINEQR.M
749	624 – 633	584.8207	1167.6269	1167.6248	0.0021	1	67	0.0023	1Score > 40 indicates **identity**	U	F.APDLIINEQR.M
311	634 – 640	458.1918	914.3691	914.3700	-0.0009	0	22	0.64	1Score > 40 indicates **identity** Score > 20	U	R.MTLPCMY.D

Query	Start – End	Observed	Mr (expt)	Mr (calc)	Delta	M	Score	Expect	Rank	U	Peptide
									indicates **homology**		
348	634 – 640	466.1894	930.3642	930.3649	-0.0007	0	31	0.45	1 Score **> 41** indicates **identity** Score **> 27** indicates **homology**	U	R.MTLPCMY.D + Oxidation (M)
349	634 – 640	466.1904	930.3662	930.3649	0.0013	0	29	0.98	1 Score **> 41** indicates **identity** Score **> 28** indicates **homology**	U	R.MTLPCMY.D + Oxidation (M)
377	634 – 640	474.1860	946.3574	946.3598	-0.0024	0	32	0.38	2 Score **> 40** indicates **identity** Score **> 28** indicates **homology**	U	R.MTLPCMY.D + 2 Oxidation (M)
1058	634 – 644	723.7967	1445.5788	1445.5811	-0.0024	1	45	0.29	1 Score **> 40** indicates **identity**	U	R.MTLPCMYDQCK.H
1090	634 – 644	731.7947	1461.5748	1461.5761	-0.0013	1	65	0.00027	1 Score **> 39** indicates **identity** Score **> 29** indicates **homology**	U	R.MTLPCMYDQCK.H + Oxidation (M)
1115	634 – 644	739.7956	1477.5766	1477.5710	0.0056	1	57	0.0016	1 Score **> 39** indicates **identity** Score **> 28** indicates **homology**	U	R.MTLPCMYDQCK.H + 2 Oxidation (M)
463	648 – 655	495.7538	989.4931	989.4930	0.0001	2	50	0.0082	1 Score **> 40** indicates **identity** Score **> 28** indicates **homology**	U	L.YVSSELHR.L
171	649 – 655	414.2232	826.4318	826.4297	0.0021	1	31	0.86	2 Score **> 38** indicates **identity** Score **> 30** indicates **homology**	U	Y.VSSELHR.L
520	656 – 663	515.7390	1029.4635	1029.4655	-0.0019	2	24	0.7	1 Score **> 41** indicates **identity** Score **> 22** indicates **homology**	U	R.LQVSYEEY.L
419	661 – 667	486.7113	971.4081	971.4092	-0.0011	2	35	0.15	1 Score **> 40** indicates **identity** Score **> 26** indicates **homology**	U	Y.EEYLCMK.T

Query	Start	–	End	Observed	Mr (expt)	Mr (calc)	Delta	M	Score	Expect	Rank	U	Peptide
86	671	–	677	372.2380	742.4615	742.4589	0.0026	2	45	0.098	1	Score > **34** indicates **identity**	U	L.LLSSVPK.D
694	682	–	690	568.7828	1135.5511	1135.5509	0.0001	2	46	0.083	1	Score > **40** indicates **identity** Score > **34** indicates **homology**	U	K.SQELFDEIR.M
695	682	–	690	568.7833	1135.5521	1135.5509	0.0012	2	41	0.066	1	Score > **40** indicates **identity** Score > **29** indicates **homology**	U	K.SQELFDEIR.M
613	704	–	712	539.2397	1076.4648	1076.4635	0.0013	1	42	0.15	1	Score > **40** indicates **identity** Score > **34** indicates **homology**	U	K.REGNSSQNW.Q
322	705	–	712	461.1903	920.3660	920.3624	0.0036	0	23	0.44	1	Score > **40** indicates **identity** Score > **19** indicates **homology**	U	R.EGNSSQNW.Q
796	705	–	714	603.2689	1204.5232	1204.5221	0.0011	1	63	0.0057	1	Score > **40** indicates **identity**	U	R.EGNSSQNWQR.F
1005	721	–	732	699.8478	1397.6810	1397.6860	-0.0051	2	37	0.91	2	Score > **40** indicates **identity** Score > **36** indicates **homology**	U	K.LLDSMHEVVENL.L
1017	721	–	732	707.8468	1413.6790	1413.6810	-0.0020	2	42	0.67	1	Score > **40** indicates **identity**	U	K.LLDSMHEVVENL.L + Oxidation (M)
1240	723	–	735	781.8584	1561.7022	1561.7082	-0.0060	2	46	0.22	2	Score > **39** indicates **identity**	U	L.DSMHEVVENLLNY.C
1257	723	–	735	789.8586	1577.7026	1577.7032	-0.0005	2	33	0.37	1	Score > **39** indicates **identity** Score > **28** indicates **homology**	U	L.DSMHEVVENLLNY.C + Oxidation (M)
1226	741	–	753	777.3812	1552.7479	1552.7517	-0.0038	2	44	0.059	1	Score > **39** indicates **identity** Score > **31** indicates **homology**	U	F.LDKTMSIEFPEML.A
1250	741	–	753	785.3794	1568.7443	1568.7466	-0.0023	2	36	0.11	1	Score > **39** indicates **identity** Score > **27**	U	F.LDKTMSIEFPEML.A + Oxidation (M)

Query	Start – End	Observed	Mr(expt)	Mr(calc)	Delta	M	Score	Expect	Rank	U	Peptide
									indicates **homology**		
1719	742 – 763	850.1032	2547.2878	2547.2964	-0.0086	2	40	0.083	1 Score **> 34** indicates **identity** Score **> 29** indicates **homology**	U	L.DKTMSIEFPEMLAEIITNQIPK.Y
1720	742 – 763	855.4339	2563.2798	2563.2913	-0.0116	2	68	0.00043	1 Score **> 34** indicates **identity**	U	L.DKTMSIEFPEMLAEIITNQIPK.Y + Oxidation (M)
1723	742 – 763	860.7687	2579.2844	2579.2862	-0.0019	2	56	0.0025	1 Score **> 34** indicates **identity** Score **> 30** indicates **homology**	U	L.DKTMSIEFPEMLAEIITNQIPK.Y + 2 Oxidation (M)
1671	744 – 763	769.0621	2304.1644	2304.1745	-0.0101	1	63	0.00012	1 Score **> 35** indicates **identity** Score **> 23** indicates **homology**	U	K.TMSIEFPEMLAEIITNQIPK.Y
1672	744 – 763	769.0628	2304.1665	2304.1745	-0.0080	1	37	0.045	1 Score **> 35** indicates **identity** Score **> 23** indicates **homology**	U	K.TMSIEFPEMLAEIITNQIPK.Y
1678	744 – 763	774.3952	2320.1638	2320.1694	-0.0056	1	63	0.00016	1 Score **> 35** indicates **identity** Score **> 25** indicates **homology**	U	K.TMSIEFPEMLAEIITNQIPK.Y + Oxidation (M)
1681	744 – 763	779.7256	2336.1550	2336.1643	-0.0093	1	29	0.92	1 Score **> 35** indicates **identity** Score **> 28** indicates **homology**	U	K.TMSIEFPEMLAEIITNQIPK.Y + 2 Oxidation (M)
1682	744 – 763	779.7263	2336.1570	2336.1643	-0.0073	1	54	0.0036	1 Score **> 35** indicates **identity** Score **> 30** indicates **homology**	U	K.TMSIEFPEMLAEIITNQIPK.Y + 2 Oxidation (M)
1706	744 – 764	823.4176	2467.2310	2467.2378	-0.0068	2	44	0.097	1 Score **> 34** indicates **identity** Score **> 33** indicates **homology**	U	K.TMSIEFPEMLAEIITNQIPKY.S
1710	744 – 764	828.7490	2483.2250	2483.2327	-0.0077	2	29	0.24	1 Score **> 34** indicates **identity** Score **> 22** indicates **homology**	U	K.TMSIEFPEMLAEIITNQIPKY.S + Oxidation (M)

Query	Start – End	Observed	Mr(expt)	Mr(calc)	Delta	M	Score	Expect	Rank	U	Peptide
681	754 – 763	563.8273	1125.6401	1125.6393	0.0007	0	48	0.14	1 Score > 40 indicates **identity** Score > 39 indicates **homology**	U	L.AEIITNQIPK.Y
887	754 – 764	645.3587	1288.7029	1288.7027	0.0002	1	44	0.04	1 Score > 40 indicates **identity** Score > 30 indicates **homology**	U	L.AEIITNQIPKY.S

MST1 trypsin and chymotrypsin digest

Glucocorticoid receptor OS=Homo sapiens OX=9606 GN=NR3C1 PE=1 SV=1

**Database:**	SwissProt
**Score:**	866
**Monoisotopic mass (M_r):**	86745
**Calculated pI:**	6.00
**Taxonomy:**	Homo sapiens

Sequence similarity is available as an NCBI BLAST search of GCR_HUMAN against nr.

Search parameters

**MS data file:**	D:\SCIEX OS Data\MGF files\May2018\300818 LBD GR MST1 double.mgf
**Enzyme:**	TrypChymo: cuts C-term side of FKLRWY unless next residue is P.
**Fixed modifications:**	Carbamidomethyl (C)
**Variable modifications:**	Oxidation (M), Phospho (ST), Phospho (Y)

Protein sequence coverage: 20%

Matched peptides shown in ***bold red***.

1 MDSKESLTPG REENPSSVLA QERGDVMDFY KTLRGGATVK VSASSPSLAV

51 ASQSDSKQRR LLVDFPKGSV SNAQQPDLSK AVSLSMGLYM GETETKVMGN

101 DLGFPQQGQI SLSSGETDLK LLEESIANLN RSTSVPENPK SSASTAVSAA

151 PTEKEFPKTH SDVSSEQQHL KGQTGTNGGN VKLYTTDQST FDILQDLEFS

201 SGSPGKETNE SPWRSDLLID ENCLLSPLAG EDDSFLLEGN SNEDCKPLIL

251 PDTKPKIKDN GDLVLSSPSN VTLPQVKTEK EDFIELCTPG VIKQEKLGTV

301 YCQASFPGAN IIGNKMSAIS VHGVSTSGGQ MYHYDMNTAS LSQQQDQKPI

351 FNVIPPIPVG SENWNRCQGS GDDNLTSLGT LNFPGRTVFS NGYSSPSMRP

401 DVSSPPSSSS TATTGPPPKL CLVCSDEASG CHYGVLTCGS CKVFFKRAVE

451 GQHNYLCAGR NDCIIDKIRR KNCPACRYRK CLQAGMNLEA RKTKKKIKGI

501 QQATTGVSQE TSENPGNKTI VPATLPQLTP TLVSL**LEVIE** **PEVLYAGYDS**

551 **SVPDSTWRIM** **TTLNMLGGRQ** **VIAAVKWAKA** **IPGF**RNLHLD **DQMTL**LQYSW

601 MFL**MAFALGW** RSYRQSSANL L**CFAPDLIIN** **EQRMTLPCMY** DQCKHMLYVS

651 SELHR**LQVSY** **EEY**LCMKTLL **LLSSVPK**DGL K**SQELFDEIR** MTYIKELGKA

701 IVKR**EGNSSQ** **NWQR**FYQLTK LL**DSMHEVVE** **NLLNY**CFQTF **LDKTMSIEFP**

751 **EMLAEIITNQ** **IPKY**SNGNIK KLLFHQK

Unformatted sequence string: 777 residues (for pasting into other applications).

Query	Start - End	Observed	Mr(expt)	Mr(calc)	Delta	M	Score	Expect	Rank	U	Peptide
718	536 - 545	602.3276	1202.6407	1202.6434	-0.0028	2	70	0.00035	1 Score **> 40** indicates **identity** Score **> 35** indicates **homology**	U	L.LEVIEPEVLY.A
806	546 - 557	642.7710	1283.5274	1283.5306	-0.0032	1	17	0.59	1 Score **> 40** indicates **identity** Score **> 15**	U	Y.AGYDSSVPDSTW.R

Query	Start – End	Observed	Mr(expt)	Mr(calc)	Delta	M	Score	Expect	Rank	U	Peptide
									indicates **homology**		
982	546 – 558	720.8225	1439.6305	1439.6317	-0.0013	2	58	0.0009	1 Score **> 40** indicates **identity** Score **> 27** indicates **homology**	U	Y.AGYDSSVPDSTWR.I
1104	546 – 558	760.8062	1519.5978	1519.5981	-0.0003	2	47	0.034	1 Score **> 39** indicates **identity** Score **> 32** indicates **homology**	U	Y.AGYDSSVPDSTWR.I + Phospho (ST)
636	549 – 558	575.2655	1148.5165	1148.5098	0.0067	1	45	0.22	1 Score **> 40** indicates **identity** Score **> 38** indicates **homology**	U	Y.DSSVPDSTWR.I
673	558 – 566	586.7805	1171.5465	1171.5494	-0.0029	2	34	0.72	1 Score **> 40** indicates **identity** Score **> 32** indicates **homology**	U	W.RIMTTLNML.G + Phospho (ST)
693	558 – 566	594.7778	1187.5411	1187.5443	-0.0033	2	33	0.27	1 Score **> 40** indicates **identity** Score **> 27** indicates **homology**	U	W.RIMTTLNML.G + Oxidation (M); Phospho (ST)
719	558 – 566	602.7756	1203.5367	1203.5393	-0.0026	2	41	0.062	1 Score **> 40** indicates **identity** Score **> 29** indicates **homology**	U	W.RIMTTLNML.G + 2 Oxidation (M); Phospho (ST)
723	559 – 569	603.8197	1205.6248	1205.6260	-0.0013	2	71	0.00086	1 Score **> 40** indicates **identity**	U	R.IMTTLNMLGGR.Q
737	559 – 569	611.8170	1221.6195	1221.6210	-0.0015	2	64	0.00016	1 Score **> 40** indicates **identity** Score **> 25** indicates **homology**	U	R.IMTTLNMLGGR.Q + Oxidation (M)
759	559 – 569	619.8157	1237.6169	1237.6159	0.0010	2	78	6.3e-05	1 Score **> 40** indicates **identity** Score **> 35** indicates **homology**	U	R.IMTTLNMLGGR.Q + 2 Oxidation (M)
810	559 – 569	643.8024	1285.5903	1285.5924	-0.0020	2	65	0.00033	1 Score **> 40** indicates **identity** Score **> 30** indicates **homology**	U	R.IMTTLNMLGGR.Q + Phospho (ST)

Query	Start – End	Observed	Mr(expt)	Mr(calc)	Delta	M	Score	Expect	Rank	U	Peptide
830	559 – 569	651.7999	1301.5852	1301.5873	-0.0021	2	34	0.27	1 Score > 40 indicates identity Score > 27 indicates homology	U	R.IMTTLNMLGGR.Q + Oxidation (M); Phospho (ST)
831	559 – 569	651.8002	1301.5858	1301.5873	-0.0015	2	50	0.012	1 Score > 40 indicates identity Score > 31 indicates homology	U	R.IMTTLNMLGGR.Q + Oxidation (M); Phospho (ST)
847	559 – 569	659.7987	1317.5829	1317.5822	0.0007	2	47	0.012	1 Score > 40 indicates identity Score > 28 indicates homology	U	R.IMTTLNMLGGR.Q + 2 Oxidation (M); Phospho (ST)
61	570 – 576	364.7374	727.4603	727.4592	0.0011	0	43	0.1	1 Score > 33 indicates identity	U	R.QVIAAVK.W
271	570 – 577	457.7762	913.5378	913.5385	-0.0008	1	58	0.02	1 Score > 40 indicates identity	U	R.QVIAAVKW.A
41	578 – 584	352.2094	702.4042	702.4064	-0.0022	1	26	0.093	1 Score > 32 indicates identity Score > 15 indicates homology	U	W.AKAIPGF.R
712	586 – 595	600.2889	1198.5633	1198.5652	-0.0019	2	44	0.39	1 Score > 40 indicates identity Score > 40 indicates homology	U	R.NLHLDDQMTL.L
732	586 – 595	608.2861	1214.5577	1214.5601	-0.0025	2	45	0.043	1 Score > 40 indicates identity Score > 31 indicates homology	U	R.NLHLDDQMTL.L + Oxidation (M)
391	588 – 595	494.7237	987.4328	987.4332	-0.0003	1	39	0.19	1 Score > 41 indicates identity Score > 31 indicates homology	U	L.HLDDQMTL.L + Oxidation (M)
111	604 – 610	398.1931	794.3717	794.3785	-0.0068	2	36	0.18	1 Score > 36 indicates identity Score > 28 indicates homology	U	L.MAFALGW.R
1039	622 – 633	738.3664	1474.7183	1474.7238	-0.0056	2	83	3e-06	1 Score > 39 indicates identity Score > 27	U	L.CFAPDLIINEQR.M

Query	Start – End	Observed	Mr(expt)	Mr(calc)	Delta	M	Score	Expect	Rank	U	Peptide
									indicates **homology**		
663	624 – 633	584.8190	1167.6235	1167.6248	-0.0013	1	73	0.00058	1Score **> 40** indicates **identity**	U	F.APDLIINEQR.M
664	624 – 633	584.8195	1167.6245	1167.6248	-0.0002	1	72	0.00081	1Score **> 40** indicates **identity**	U	F.APDLIINEQR.M
305	634 – 640	466.1899	930.3652	930.3649	0.0003	0	31	0.44	1Score **> 41** indicates **identity** Score **> 27** indicates **homology**	U	R.MTLPCMY.D + Oxidation (M)
306	634 – 640	466.1902	930.3659	930.3649	0.0010	0	39	0.049	1Score **> 41** indicates **identity** Score **> 25** indicates **homology**	U	R.MTLPCMY.D + Oxidation (M)
326	634 – 640	474.1860	946.3574	946.3598	-0.0025	0	31	0.57	2Score **> 40** indicates **identity** Score **> 28** indicates **homology**	U	R.MTLPCMY.D + 2 Oxidation (M)
449	656 – 663	515.7392	1029.4638	1029.4655	-0.0017	2	31	0.52	1Score **> 41** indicates **identity** Score **> 28** indicates **homology**	U	R.LQVSYEEY.L
73	671 – 677	372.2367	742.4588	742.4589	-0.0001	2	46	0.069	1Score **> 34** indicates **identity**	U	L.LLSSVPK.D
615	682 – 690	568.7822	1135.5499	1135.5509	-0.0010	2	52	0.025	1Score **> 40** indicates **identity** Score **> 35** indicates **homology**	U	K.SQELFDEIR.M
616	682 – 690	568.7827	1135.5508	1135.5509	-0.0001	2	42	0.13	1Score **> 40** indicates **identity** Score **> 32** indicates **homology**	U	K.SQELFDEIR.M
278	705 – 712	461.1889	920.3632	920.3624	0.0008	0	30	0.1	1Score **> 40** indicates **identity** Score **> 20** indicates **homology**	U	R.EGNSSQNW.Q
720	705 – 714	603.2717	1204.5289	1204.5221	0.0068	1	64	0.005	1Score **> 40** indicates **identity**	U	R.EGNSSQNWQR.F

Query	Start – End	Observed	Mr(expt)	Mr(calc)	Delta	M	Score	Expect	Rank	U	Peptide
807	705 – 714	643.2519	1284.4892	1284.4884	0.0008	1	24	0.96	1 Score **> 40** indicates **identity** Score **> 23** indicates **homology**	U	R.EGNSSQNWQR.F + Phospho (ST)
1154	723 – 735	781.8578	1561.7011	1561.7082	-0.0071	2	54	0.0085	1 Score **> 39** indicates **identity** Score **> 33** indicates **homology**	U	L.DSMHEVVENLLNY.C
1146	741 – 753	777.3820	1552.7494	1552.7517	-0.0023	2	22	0.8	1 Score **> 39** indicates **identity** Score **> 21** indicates **homology**	U	F.LDKTMSIEFPEML.A
1166	741 – 753	785.3778	1568.7411	1568.7466	-0.0055	2	36	0.035	1 Score **> 39** indicates **identity** Score **> 21** indicates **homology**	U	F.LDKTMSIEFPEML.A + Oxidation (M)
1178	741 – 753	793.3762	1584.7379	1584.7415	-0.0036	2	31	0.28	1 Score **> 39** indicates **identity** Score **> 25** indicates **homology**	U	F.LDKTMSIEFPEML.A + 2 Oxidation (M)
1006	742 – 753	728.8394	1455.6643	1455.6625	0.0017	1	31	0.33	1 Score **> 39** indicates **identity** Score **> 26** indicates **homology**	U	L.DKTMSIEFPEML.A + Oxidation (M)
1607	742 – 763	855.4362	2563.2868	2563.2913	-0.0045	2	57	0.0025	1 Score **> 34** indicates **identity** Score **> 31** indicates **homology**	U	L.DKTMSIEFPEMLAEIITNQIPK.Y + Oxidation (M)
728	744 – 753	607.2768	1212.5390	1212.5406	-0.0016	0	35	0.97	1 Score **> 40** indicates **identity** Score **> 35** indicates **homology**	U	K.TMSIEFPEML.A + Oxidation (M)
1546	744 – 763	769.0642	2304.1706	2304.1745	-0.0039	1	46	0.022	1 Score **> 36** indicates **identity** Score **> 29** indicates **homology**	U	K.TMSIEFPEMLAEIITNQIPK.Y
1554	744 – 763	774.3929	2320.1569	2320.1694	-0.0125	1	48	0.021	1 Score **> 35** indicates **identity** Score **> 31** indicates **homology**	U	K.TMSIEFPEMLAEIITNQIPK.Y + Oxidation (M)

Query	Start - End	Observed	Mr(expt)	Mr(calc)	Delta	M	Score	Expect	Rank	U	Peptide
1555	744 - 763	774.3930	2320.1571	2320.1694	-0.0123	1	75	3.8e-05	1 Score > 35 indicates **identity** Score > 31 indicates **homology**	U	K.TMSIEFPEMLAEIITNQIPK.Y + Oxidation (M)
1562	744 - 763	779.7246	2336.1520	2336.1643	-0.0124	1	68	0.00024	1 Score > 35 indicates **identity** Score > 31 indicates **homology**	U	K.TMSIEFPEMLAEIITNQIPK.Y + 2 Oxidation (M)
1589	744 - 764	823.4191	2467.2356	2467.2378	-0.0023	2	35	0.055	1 Score > 34 indicates **identity** Score > 22 indicates **homology**	U	K.TMSIEFPEMLAEIITNQIPKY.S
606	754 - 763	563.8276	1125.6406	1125.6393	0.0013	0	46	0.087	1 Score > 40 indicates **identity** Score > 35 indicates **homology**	U	L.AEIITNQIPK.Y
814	754 - 764	645.3574	1288.7003	1288.7027	-0.0023	1	56	0.0016	1 Score > 40 indicates **identity** Score > 27 indicates **homology**	U	L.AEIITNQIPKY.S

MST2 trypsin and chymotrypsin digest

Protein View: GCR_HUMAN

Glucocorticoid receptor OS=Homo sapiens OX=9606 GN=NR3C1 PE=1 SV=1

**Database:**	SwissProt
**Score:**	788
**Monoisotopic mass (M_r):**	86745
**Calculated pI:**	6.00
**Taxonomy:**	Homo sapiens

Sequence similarity is available as an NCBI BLAST search of GCR_HUMAN against nr.

Search parameters

**MS data file:**	D:\SCIEX OS Data\MGF files\May2018\300818 LBD GR MST2 double.mgf
**Enzyme:**	TrypChymo: cuts C-term side of FKLRWY unless next residue is P.
**Fixed modifications:**	Carbamidomethyl (C)
**Variable modifications:**	Oxidation (M), Phospho (ST), Phospho (Y)

Protein sequence coverage: 19%

Matched peptides shown in ***bold red***.

```
  1 MDSKESLTPG REENPSSVLA QERGDVMDFY KTLRGGATVK VSASSPSLAV
 51 ASQSDSKQRR LLVDFPKGSV SNAQQPDLSK AVSLSMGLYM GETETKVMGN
101 DLGFPQQGQI SLSSGETDLK LLEESIANLN RSTSVPENPK SSASTAVSAA
151 PTEKEFPKTH SDVSSEQQHL KGQTGTNGGN VKLYTTDQST FDILQDLEFS
201 SGSPGKETNE SPWRSDLLID ENCLLSPLAG EDDSFLLEGN SNEDCKPLIL
251 PDTKPKIKDN GDLVLSSPSN VTLPQVKTEK EDFIELCTPG VIKQEKLGTV
301 YCQASFPGAN IIGNKMSAIS VHGVSTSGGQ MYHYDMNTAS LSQQQDQKPI
351 FNVIPPIPVG SENWNRCQGS GDDNLTSLGT LNFPGRTVFS NGYSSPSMRP
401 DVSSPPSSSS TATTGPPPKL CLVCSDEASG CHYGVLTCGS CKVFFKRAVE
451 GQHNYLCAGR NDCIIDKIRR KNCPACRYRK CLQAGMNLEA RKTKKKIKGI
501 QQATTGVSQE TSENPGNKTI VPATLPQLTP TLVSLLEVIE PEVLYAGYDS
551 SVPDSTWRIM TTLNMLGGRQ VIAAVKWAKA IPGFRNLHLD DQMTLLQYSW
601 MFLMAFALGW RSYRQSSANL LCFAPDLIIN EQRMTLPCMY DQCKHMLYVS
651 SELHRLQVSY EEYLCMKTLL LLSSVPKDGL KSQELFDEIR MTYIKELGKA
701 IVKREGNSSQ NWQRFYQLTK LLDSMHEVVE NLLNYCFQTF LDKTMSIEFP
751 EMLAEIITNQ IPKYSNGNIK KLLFHQK
```

Unformatted sequence string: 777 residues (for pasting into other applications).

Query	Start	–	End	Observed	Mr (expt)	Mr (calc)	Delta	M	Score	Expect	Rank	U	Peptide
464	537	–	545	545.7859	1089.5573	1089.5594	-0.0021	1	41	0.9	4Score > **40** indicates **identity** Score > **40** indicates **homology**	U	L.EVIEPEVLY.A
641	546	–	557	642.7735	1283.5325	1283.5306	0.0019	1	17	0.9	1Score > **40** indicates **identity** Score > **16** indicates **homology**	U	Y.AGYDSSVPDSTW.R
768	546	–	558	720.8241	1439.6336	1439.6317	0.0019	2	63	0.00033	1Score > **40** indicates **identity** Score > **28** indicates **homology**	U	Y.AGYDSSVPDSTWR.I
869	546	–	558	760.8048	1519.5950	1519.5981	-0.0030	2	45	0.017	1Score > **39** indicates **identity** Score > **27** indicates **homology**	U	Y.AGYDSSVPDSTWR.I + Phospho (ST)
516	549	–	558	575.2631	1148.5117	1148.5098	0.0019	1	45	0.058	1Score > **40** indicates **identity** Score > **32** indicates **homology**	U	Y.DSSVPDSTWR.I
542	558	–	566	586.7814	1171.5482	1171.5494	-0.0013	2	37	0.19	1Score > **40** indicates **identity** Score > **30** indicates **homology**	U	W.RIMTTLNML.G + Phospho (ST)
580	559	–	569	603.8210	1205.6275	1205.6260	0.0015	2	33	0.12	1Score > **40** indicates **identity** Score > **24** indicates **homology**	U	R.IMTTLNMLGGR.Q
593	559	–	569	611.8184	1221.6223	1221.6210	0.0013	2	30	0.59	1Score > **40** indicates **identity** Score > **27** indicates **homology**	U	R.IMTTLNMLGGR.Q + Oxidation (M)
594	559	–	569	611.8187	1221.6228	1221.6210	0.0019	2	51	0.012	1Score > **40** indicates **identity** Score > **31** indicates **homology**	U	R.IMTTLNMLGGR.Q + Oxidation (M)
603	559	–	569	619.8157	1237.6169	1237.6159	0.0010	2	43	0.0094	1Score > **40** indicates	U	R.IMTTLNMLGGR.Q

Query	Start – End	Observed	Mr (expt)	Mr (calc)	Delta	M	Score	Expect	Rank	U	Peptide
									identity Score > 22 indicates homology		+ 2 Oxidation (M)
644	559 – 569	643.8041	1285.5937	1285.5924	0.0014	2	57	0.007	1Score > 40 indicates identity Score > 35 indicates homology	U	R.IMTTLNMLGGR.Q + Phospho (ST)
656	559 – 569	651.8011	1301.5877	1301.5873	0.0004	2	50	0.059	1Score > 40 indicates identity Score > 37 indicates homology	U	R.IMTTLNMLGGR.Q + Oxidation (M); Phospho (ST)
657	559 – 569	651.8017	1301.5888	1301.5873	0.0015	2	69	4.5e-05	1Score > 40 indicates identity Score > 25 indicates homology	U	R.IMTTLNMLGGR.Q + Oxidation (M); Phospho (ST)
671	559 – 569	659.7988	1317.5831	1317.5822	0.0009	2	40	0.0077	1Score > 40 indicates identity Score > 19 indicates homology	U	R.IMTTLNMLGGR.Q + 2 Oxidation (M); Phospho (ST)
54	570 – 576	364.7375	727.4604	727.4592	0.0012	0	41	0.15	1Score > 33 indicates identity	U	R.QVIAAVK.W
232	570 – 577	457.7776	913.5406	913.5385	0.0021	1	61	0.0091	1Score > 40 indicates identity	U	R.QVIAAVKW.A
570	586 – 595	600.2906	1198.5667	1198.5652	0.0014	2	40	0.21	1Score > 40 indicates identity Score > 33 indicates homology		R.NLHLDDQMTL.L
590	586 – 595	608.2878	1214.5611	1214.5601	0.0009	2	30	0.7	1Score > 40 indicates identity Score > 28 indicates homology		R.NLHLDDQMTL.L + Oxidation (M)
308	588 – 595	486.7271	971.4397	971.4382	0.0015	1	42	0.13	1Score > 40 indicates identity Score > 33 indicates homology		L.HLDDQMTL.L
337	588 – 595	494.7259	987.4373	987.4332	0.0041	1	42	0.4	1Score > 41 indicates identity Score > 38 indicates homology		L.HLDDQMTL.L + Oxidation (M)

Query	Start – End	Observed	Mr(expt)	Mr(calc)	Delta	M	Score	Expect	Rank	U	Peptide
107	604 – 610	406.1939	810.3733	810.3734	-0.0001	2	37	0.68	1 Score > **37** indicates **identity** Score > **35** indicates **homology**	U	L.MAFALGW.R + Oxidation (M)
195	614 – 621	444.7491	887.4836	887.4825	0.0012	2	49	0.14	1 Score > **40** indicates **identity**	U	Y.RQSSANLL.C
819	622 – 633	492.5818	1474.7235	1474.7238	-0.0004	2	39	0.26	1 Score > **39** indicates **identity** Score > **33** indicates **homology**	U	L.CFAPDLIINEQR.M
536	624 – 633	584.8206	1167.6267	1167.6248	0.0019	1	41	0.23	1 Score > **40** indicates **identity** Score > **34** indicates **homology**	U	F.APDLIINEQR.M
537	624 – 633	584.8217	1167.6288	1167.6248	0.0040	1	66	0.0032	1 Score > **40** indicates **identity**	U	F.APDLIINEQR.M
263	634 – 640	466.1903	930.3660	930.3649	0.0011	0	31	0.44	1 Score > **41** indicates **identity** Score > **27** indicates **homology**	U	R.MTLPCMY.D + Oxidation (M)
264	634 – 640	466.1906	930.3666	930.3649	0.0017	0	33	0.18	1 Score > **41** indicates **identity** Score > **26** indicates **homology**	U	R.MTLPCMY.D + Oxidation (M)
342	634 – 640	498.1760	994.3375	994.3363	0.0011	0	34	0.48	1 Score > **40** indicates **identity** Score > **31** indicates **homology**	U	R.MTLPCMY.D + Phospho (ST)
802	634 – 644	731.7948	1461.5751	1461.5761	-0.0010	1	38	0.35	1 Score > **39** indicates **identity** Score > **33** indicates **homology**	U	R.MTLPCMYDQCK.H + Oxidation (M)
823	634 – 644	739.7900	1477.5654	1477.5710	-0.0056	1	38	0.16	1 Score > **39** indicates **identity** Score > **30** indicates **homology**	U	R.MTLPCMYDQCK.H + 2 Oxidation (M)
63	671 – 677	372.2374	742.4603	742.4589	0.0014	2	44	0.12	1 Score > **34** indicates **identity**	U	L.LLSSVPK.D
503	682 – 690	568.7839	1135.5532	1135.5509	0.0022	2	44	0.082	1 Score > **40** indicates	U	K.SQELFDEIR.M

Query	Start – End	Observed	Mr(expt)	Mr(calc)	Delta	M	Score	Expect	Rank	U	Peptide
									identity Score > 33 indicates homology		
504	682 – 690	568.7852	1135.5559	1135.5509	0.0050	2	47	0.032	1Score > 40 indicates identity Score > 32 indicates homology	U	K.SQELFDEIR.M
447	704 – 712	539.2380	1076.4615	1076.4635	-0.0020	1	31	0.4	1Score > 40 indicates identity Score > 27 indicates homology	U	K.REGNSSQNW.Q
240	705 – 712	461.1897	920.3649	920.3624	0.0025	0	25	0.23	1Score > 40 indicates identity Score > 18 indicates homology	U	R.EGNSSQNW.Q
577	705 – 714	603.2700	1204.5254	1204.5221	0.0033	1	63	0.0059	1Score > 40 indicates identity	U	R.EGNSSQNWQR.F
906	723 – 735	781.8559	1561.6973	1561.7082	-0.0110	2	42	0.24	1Score > 39 indicates identity Score > 35 indicates homology	U	L.DSMHEVVENLLNY.C
907	723 – 735	781.8632	1561.7119	1561.7082	0.0036	2	66	0.0025	1Score > 39 indicates identity	U	L.DSMHEVVENLLNY.C
918	723 – 735	789.8558	1577.6970	1577.7032	-0.0061	2	49	0.027	1Score > 39 indicates identity Score > 33 indicates homology	U	L.DSMHEVVENLLNY.C + Oxidation (M)
898	741 – 753	777.3823	1552.7500	1552.7517	-0.0017	2	38	0.16	1Score > 39 indicates identity Score > 30 indicates homology	U	F.LDKTMSIEFPEML.A
914	741 – 753	785.3797	1568.7448	1568.7466	-0.0018	2	40	0.098	1Score > 39 indicates identity Score > 29 indicates homology	U	F.LDKTMSIEFPEML.A + Oxidation (M)
924	741 – 753	793.3766	1584.7386	1584.7415	-0.0030	2	26	0.45	1Score > 39 indicates identity Score > 23 indicates homology	U	F.LDKTMSIEFPEML.A + 2 Oxidation (M)

Query	Start	–	End	Observed	Mr(expt)	Mr(calc)	Delta	M	Score	Expect	Rank	U	Peptide
1231	742	–	763	855.4345	2563.2816	2563.2913	-0.0097	2	44	0.11	1	Score > **34** indicates **identity**	U	L.DKTMSIEFPEMLAEIITNQIPK.Y + Oxidation (M)
1234	742	–	763	860.7673	2579.2802	2579.2862	-0.0060	2	54	0.0096	1	Score > **34** indicates **identity**	U	L.DKTMSIEFPEMLAEIITNQIPK.Y + 2 Oxidation (M)
586	744	–	753	607.2767	1212.5389	1212.5406	-0.0017	0	35	0.11	1	Score > **40** indicates **identity** Score > **25** indicates **homology**	U	K.TMSIEFPEML.A + Oxidation (M)
1190	744	–	763	769.0606	2304.1601	2304.1745	-0.0144	1	60	0.00023	1	Score > **35** indicates **identity** Score > **23** indicates **homology**	U	K.TMSIEFPEMLAEIITNQIPK.Y
1191	744	–	763	769.0617	2304.1634	2304.1745	-0.0111	1	50	0.0021	1	Score > **35** indicates **identity** Score > **22** indicates **homology**	U	K.TMSIEFPEMLAEIITNQIPK.Y
1195	744	–	763	774.3961	2320.1666	2320.1694	-0.0028	1	60	0.0012	1	Score > **35** indicates **identity** Score > **30** indicates **homology**	U	K.TMSIEFPEMLAEIITNQIPK.Y + Oxidation (M)
1196	744	–	763	774.3965	2320.1676	2320.1694	-0.0018	1	71	0.00028	1	Score > **35** indicates **identity**	U	K.TMSIEFPEMLAEIITNQIPK.Y + Oxidation (M)
1201	744	–	763	779.7263	2336.1572	2336.1643	-0.0072	1	64	0.00056	1	Score > **35** indicates **identity** Score > **31** indicates **homology**	U	K.TMSIEFPEMLAEIITNQIPK.Y + 2 Oxidation (M)
1223	744	–	764	823.4168	2467.2286	2467.2378	-0.0093	2	56	0.00047	1	Score > **34** indicates **identity** Score > **23** indicates **homology**	U	K.TMSIEFPEMLAEIITNQIPKY.S
494	754	–	763	563.8278	1125.6410	1125.6393	0.0016	0	48	0.19	1	Score > **40** indicates **identity**		L.AEIITNQIPK.Y
646	754	–	764	645.3588	1288.7031	1288.7027	0.0005	1	40	0.073	1	Score > **40** indicates **identity** Score > **28** indicates **homology**	U	L.AEIITNQIPKY.S

Rock1 trypsin and chymotrypsin digest

Glucocorticoid receptor OS=Homo sapiens OX=9606 GN=NR3C1 PE=1 SV=1

**Database:**	SwissProt
**Score:**	817
**Monoisotopic mass (M_r):**	86745
**Calculated pI:**	6.00
**Taxonomy:**	Homo sapiens

Sequence similarity is available as an NCBI BLAST search of GCR_HUMAN against nr.

Search parameters

**MS data file:**	D:\SCIEX OS Data\MGF files\May2018\300818 LBD GR Rock1 double.mgf
**Enzyme:**	TrypChymo: cuts C-term side of FKLRWY unless next residue is P.
**Fixed modifications:**	Carbamidomethyl (C)
**Variable modifications:**	Oxidation (M), Phospho (ST), Phospho (Y)

Protein sequence coverage: 20%

Matched peptides shown in ***bold red***.

```
  1 MDSKESLTPG REENPSSVLA QERGDVMDFY KTLRGGATVK VSASSPSLAV
 51 ASQSDSKQRR LLVDFPKGSV SNAQQPDLSK AVSLSMGLYM GETETKVMGN
101 DLGFPQQGQI SLSSGETDLK LLEESIANLN RSTSVPENPK SSASTAVSAA
151 PTEKEFPKTH SDVSSEQQHL KGQTGTNGGN VKLYTTDQST FDILQDLEFS
201 SGSPGKETNE SPWRSDLLID ENCLLSPLAG EDDSFLLEGN SNEDCKPLIL
```

251 PDTKPKIKDN GDLVLSSPSN VTLPQVKTEK EDFIELCTPG VIKQEKLGTV

301 YCQASFPGAN IIGNKMSAIS VHGVSTSGGQ MYHYDMNTAS LSQQQDQKPI

351 FNVIPPIPVG SENWNRCQGS GDDNLTSLGT LNFPGRTVFS NGYSSPSMRP

401 DVSSPPSSSS TATTGPPPKL CLVCSDEASG CHYGVLTCGS CKVFFKRAVE

451 GQHNYLCAGR NDCIIDKIRR KNCPACRYRK CLQAGMNLEA RKTKKKIKGI

501 QQATTGVSQE TSENPGNKTI VPATLPQLTP TLVSL**LEVIE** **PEVLYAGYDS**

551 **SVPDSTWRIM** **TTLNMLGGRQ** **VIAAVKW**AKA IPGFR**NLHLD** **DQMTL**LQYSW

601 MFL**MAFALGW** RSYRQSSANL L**CFAPDLIIN** **EQRMTLPCMY** **DQCK**HMLYVS

651 SELHR**LQVSY** **EEY**LCMKTLL **LLSSVPK**DGL K**SQELFDEIR** **MTY**IKELGKA

701 IVKR**EGNSSQ** **NWQRF**YQLTK LL**DSMHEVVE** **NLLNY**CFQTF **LDKTMSIEFP**

751 **EMLAEIITNQ** **IPKY**SNGNIK KLLFHQK

Unformatted sequence string: 777 residues (for pasting into other applications).

Query	Start	–	End	Observed	Mr(expt)	Mr(calc)	Delta	M	Score	Expect	Rank	U	Peptide
518	536	–	545	602.3299	1202.6452	1202.6434	0.0018	2	36	0.39	1 Score **>** **40** indicates **identity** Score **>** **32** indicates **homology**	U	L.LEVIEPEVLY.A
247	537	–	544	464.2559	926.4972	926.4960	0.0011	0	42	0.75	2 Score **>** **40** indicates **identity**	U	L.EVIEPEVL.Y
439	537	–	545	545.7874	1089.5603	1089.5594	0.0009	1	40	0.55	4 Score **>** **40** indicates **identity** Score **>** **37** indicates **homology**	U	L.EVIEPEVLY.A
620	537	–	548	691.3472	1380.6798	1380.6813	-0.0015	2	48	0.016	1 Score **>** **40** indicates **identity** Score **>** **30** indicates **homology**	U	L.EVIEPEVLYAGY.D
657	545	–	557	724.3037	1446.5928	1446.5939	-0.0012	2	19	0.65	1 Score **>** **40** indicates **identity** Score **>** **16** indicates **homology**	U	L.YAGYDSSVPDSTW.R
568	546	–	557	642.7742	1283.5337	1283.5306	0.0031	1	31	0.25	1 Score **>** **40** indicates **identity** Score **>** **24** indicates **homology**	U	Y.AGYDSSVPDSTW.R
650	546	–	558	720.8229	1439.6313	1439.6317	-0.0004	2	47	0.16	1 Score **>** **40** indicates **identity** Score **>** **39** indicates **homology**	U	Y.AGYDSSVPDSTWR.I

Query	Start – End	Observed	Mr(expt)	Mr(calc)	Delta	M	Score	Expect	Rank	U	Peptide
331	549 – 557	497.2135	992.4125	992.4087	0.0038	0	22	0.87	1 Score **>** **40** indicates **identity** Score **>** **20** indicates **homology**	U	Y.DSSVPDSTW.R
478	549 – 558	575.2625	1148.5104	1148.5098	0.0006	1	51	0.036	1 Score **>** **40** indicates **identity** Score **>** **37** indicates **homology**	U	Y.DSSVPDSTWR.I
479	549 – 558	575.2629	1148.5112	1148.5098	0.0014	1	50	0.042	1 Score **>** **40** indicates **identity** Score **>** **36** indicates **homology**	U	Y.DSSVPDSTWR.I
278	559 – 566	476.7455	951.4764	951.4769	-0.0006	1	27	0.62	2 Score **>** **40** indicates **identity** Score **>** **25** indicates **homology**	U	R.IMTTLNML.G + Oxidation (M)
522	559 – 569	603.8211	1205.6277	1205.6260	0.0017	2	56	0.019	1 Score **>** **40** indicates **identity** Score **>** **39** indicates **homology**	U	R.IMTTLNMLGGR.Q
531	559 – 569	611.8185	1221.6224	1221.6210	0.0015	2	71	4.5e-05	1 Score **>** **40** indicates **identity** Score **>** **27** indicates **homology**	U	R.IMTTLNMLGGR.Q + Oxidation (M)
532	559 – 569	611.8187	1221.6228	1221.6210	0.0019	2	56	0.004	1 Score **>** **40** indicates **identity** Score **>** **31** indicates **homology**	U	R.IMTTLNMLGGR.Q + Oxidation (M)
538	559 – 569	619.8161	1237.6176	1237.6159	0.0017	2	66	0.00028	1 Score **>** **40** indicates **identity** Score **>** **30** indicates **homology**	U	R.IMTTLNMLGGR.Q + 2 Oxidation (M)
54	570 – 576	364.7398	727.4650	727.4592	0.0057	0	43	0.071	1 Score **>** **33** indicates **identity** Score **>** **31** indicates **homology**	U	R.QVIAAVK.W
232	570 – 577	457.7766	913.5386	913.5385	0.0001	1	54	0.042	1 Score **>** **40** indicates **identity**	U	R.QVIAAVKW.A
529	586 – 595	608.2868	1214.5590	1214.5601	-0.0011	2	37	0.2	1 Score **>** **40** indicates **identity** Score **>** **30** indicates **homology**	U	R.NLHLDDQMTL.L + Oxidation (M)
304	588 – 595	486.7272	971.4398	971.4382	0.0016	1	35	0.46	1 Score **>** **40** indicates **identity** Score **>** **31** indicates **homology**	U	L.HLDDQMTL.L
329	588 – 595	494.7254	987.4362	987.4332	0.0030	1	38	0.98	1 Score **>** **41** indicates **identity** Score **>** **37** indicates **homology**	U	L.HLDDQMTL.L + Oxidation (M)
100	604 – 610	398.1968	794.3791	794.3785	0.0005	2	41	0.21	1 Score **>** **36** indicates **identity** Score **>** **34** indicates **homology**	U	L.MAFALGW.R

Query	Start – End	Observed	Mr(expt)	Mr(calc)	Delta	M	Score	Expect	Rank	U	Peptide
109	604 – 610	406.1939	810.3733	810.3734	-0.0002	2	35	0.5	2 Score **>** **37** indicates **identity** Score **>** **32** indicates **homology**	U	L.MAFALGW.R + Oxidation (M)
684	622 – 633	492.5818	1474.7237	1474.7238	-0.0001	2	46	0.017	1 Score **>** **39** indicates **identity** Score **>** **28** indicates **homology**	U	L.CFAPDLIINEQR.M
497	624 – 633	584.8204	1167.6263	1167.6248	0.0016	1	47	0.13	1 Score **>** **40** indicates **identity** Score **>** **38** indicates **homology**	U	F.APDLIINEQR.M
498	624 – 633	584.8209	1167.6272	1167.6248	0.0024	1	57	0.023	1 Score **>** **40** indicates **identity**	U	F.APDLIINEQR.M
257	634 – 640	466.1902	930.3658	930.3649	0.0009	0	31	0.25	1 Score **>** **41** indicates **identity** Score **>** **24** indicates **homology**	U	R.MTLPCMY.D + Oxidation (M)
656	634 – 644	723.7979	1445.5812	1445.5811	0.0001	1	56	0.0014	1 Score **>** **40** indicates **identity** Score **>** **27** indicates **homology**	U	R.MTLPCMYDQCK.H
673	634 – 644	731.7971	1461.5797	1461.5761	0.0036	1	56	0.0028	1 Score **>** **39** indicates **identity** Score **>** **30** indicates **homology**	U	R.MTLPCMYDQCK.H + Oxidation (M)
688	634 – 644	739.7939	1477.5732	1477.5710	0.0022	1	52	0.0066	1 Score **>** **39** indicates **identity** Score **>** **30** indicates **homology**	U	R.MTLPCMYDQCK.H + 2 Oxidation (M)
366	656 – 663	515.7412	1029.4678	1029.4655	0.0023	2	25	0.89	1 Score **>** **41** indicates **identity** Score **>** **24** indicates **homology**	U	R.LQVSYEEY.L
65	671 – 677	372.2367	742.4589	742.4589	0.0000	2	44	0.12	1 Score **>** **34** indicates **identity**	U	L.LLSSVPK.D
468	682 – 690	568.7821	1135.5497	1135.5509	-0.0012	2	56	0.0061	1 Score **>** **40** indicates **identity** Score **>** **33** indicates **homology**	U	K.SQELFDEIR.M
437	686 – 693	545.7477	1089.4808	1089.4801	0.0007	2	24	0.96	2 Score **>** **40** indicates **identity** Score **>** **24** indicates **homology**	U	L.FDEIRMTY.I + Oxidation (M)
269	687 – 693	472.2142	942.4138	942.4117	0.0022	1	27	0.4	1 Score **>** **40** indicates **identity** Score **>** **22** indicates **homology**	U	F.DEIRMTY.I + Oxidation (M)
238	705 – 712	461.1894	920.3642	920.3624	0.0018	0	25	0.13	1 Score **>** **40** indicates **identity** Score **>** **16** indicates **homology**	U	R.EGNSSQNW.Q
519	705 – 714	603.2678	1204.5210	1204.5221	-0.0011	1	63	0.0057	1 Score **>** **40** indicates **identity**	U	R.EGNSSQNWQR.F

Query	Start – End	Observed	Mr(expt)	Mr(calc)	Delta	M	Score	Expect	Rank	U	Peptide
606	705 – 715	676.8041	1351.5936	1351.5905	0.0031	2	44	0.056	1Score **> 40** indicates **identity** Score **> 31** indicates **homology**	U	R.EGNSSQNWQRF.Y
747	723 – 735	781.8563	1561.6981	1561.7082	-0.0101	2	38	0.21	1Score **> 39** indicates **identity** Score **> 30** indicates **homology**	U	L.DSMHEVVENLLNY.C
755	723 – 735	789.8602	1577.7059	1577.7032	0.0028	2	46	0.22	1Score **> 39** indicates **identity**	U	L.DSMHEVVENLLNY.C + Oxidation (M)
743	741 – 753	777.3835	1552.7525	1552.7517	0.0009	2	29	0.26	1Score **> 39** indicates **identity** Score **> 23** indicates **homology**	U	F.LDKTMSIEFPEML.A
753	741 – 753	785.3797	1568.7448	1568.7466	-0.0018	2	23	0.66	1Score **> 39** indicates **identity** Score **> 21** indicates **homology**	U	F.LDKTMSIEFPEML.A + Oxidation (M)
762	741 – 753	793.3775	1584.7405	1584.7415	-0.0010	2	45	0.0045	1Score **> 39** indicates **identity** Score **> 21** indicates **homology**	U	F.LDKTMSIEFPEML.A + 2 Oxidation (M)
973	742 – 763	855.4355	2563.2848	2563.2913	-0.0066	2	67	0.00025	1Score **> 34** indicates **identity** Score **> 31** indicates **homology**	U	L.DKTMSIEFPEMLAEIITNQIPK.Y + Oxidation (M)
974	742 – 763	860.7665	2579.2778	2579.2862	-0.0085	2	89	3.3e-06	1Score **> 34** indicates **identity**	U	L.DKTMSIEFPEMLAEIITNQIPK.Y + 2 Oxidation (M)
513	744 – 753	599.2785	1196.5425	1196.5457	-0.0032	0	25	0.46	1Score **> 40** indicates **identity** Score **> 21** indicates **homology**	U	K.TMSIEFPEML.A
527	744 – 753	607.2771	1212.5396	1212.5406	-0.0010	0	21	0.58	1Score **> 40** indicates **identity** Score **> 18** indicates **homology**	U	K.TMSIEFPEML.A + Oxidation (M)
950	744 – 763	769.0654	2304.1743	2304.1745	-0.0002	1	59	0.001	1Score **> 36** indicates **identity** Score **> 29** indicates **homology**	U	K.TMSIEFPEMLAEIITNQIPK.Y
954	744 – 763	774.3965	2320.1676	2320.1694	-0.0018	1	61	0.0012	1Score **> 35** indicates **identity** Score **> 31** indicates **homology**	U	K.TMSIEFPEMLAEIITNQIPK.Y + Oxidation (M)
955	744 – 763	774.3978	2320.1715	2320.1694	0.0021	1	75	0.00011	1Score **> 35** indicates **identity** Score **> 34** indicates **homology**	U	K.TMSIEFPEMLAEIITNQIPK.Y + Oxidation (M)
960	744 – 763	779.7288	2336.1645	2336.1643	0.0001	1	74	0.00012	1Score **> 35** indicates **identity** Score **> 34** indicates **homology**	U	K.TMSIEFPEMLAEIITNQIPK.Y + 2 Oxidation (M)
971	744 – 764	823.4170	2467.2292	2467.2378	-0.0087	2	38	0.16	1Score **> 34** indicates **identity**	U	K.TMSIEFPEMLAEIITNQIPKY.S

Query	Start – End	Observed	Mr(expt)	Mr(calc)	Delta	M	Score	Expect	Rank	U	Peptide
									Score > 30 indicates homology		
461	754 – 763	563.8287	1125.6429	1125.6393	0.0035	0	56	0.021	1Score > 40 indicates identity Score > 39 indicates homology	U	L.AEIITNQIPK.Y
574	754 – 764	645.3596	1288.7047	1288.7027	0.0020	1	44	0.044	1Score > 40 indicates identity Score > 30 indicates homology	U	L.AEIITNQIPKY.S

2.3. Cell culture, transfection and stimulation

Cells were treated as followed:

Compound	Stock concentration	Conditions
**IGF1**	100 µg/mL	100 ng/mL for 20 min
**PI103**	10 mM	1 µM 30 min
**Forskolin**	20 mM	20 µM for 30 min
**H89**	20 mM	30 µM for 30 min
**Calyculin A**	10 µM	50 nM for 10 min

Treatment name	Procedure
**IGF1**	Treatment with IGF1
**PI103**	Pre-treatment with PI103 then treatment with IGF1
**Forskolin**	Treatment with forskolin
**H89**	Pre-treatment with H89 then treatment with forskolin
**Calyculin A**	Treatment with calyculin A

3. Supporting figures

A

Peptide name	K_d with 14-3-3σ by FP (µM)
**GR_pS83**	114 ± 24
**GR_pS134**	113 ± 22
**GR_pT493**	> 150
**GR_pT524**	88 ± 1
**GR_pT561**	94 ± 46
**GR_pS617**	20 ± 16
**GR_pT635**	62 ± 25
**GR_pT524-pS617**	0.053 ± 0.026

B

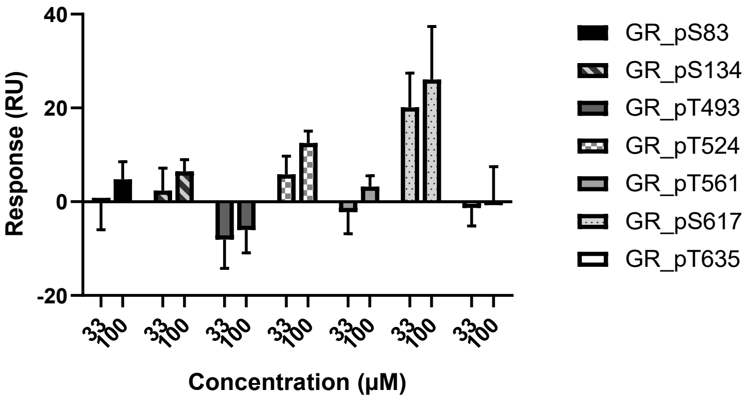

C

Peptide name	K_d with 14-3-3σ by SPR (µM)
**GR_pT524**	41 ± 35
**GR_pS617**	54 ± 21
**GR_pT524-pS617**	13 ± 18

Supporting Fig. S1. Interaction of GR peptides with 14-3-3σ. (**A**), Binding affinity of the monophosphorylated GR peptides centered on the key residue and GR_pT524-pS617 measured by FP. (**B**), Affinity of monophosphorylated GR peptides with 14-3-3ζ measured by SPR at two different peptide concentrations (33 and 100 µM). (**C**), Binding affinity of GR_pT524, GR_pS617 and GR_pT524-pS617 measured by SPR. Measurements were performed as triplicates and the error bars represent the standard deviation of these three independent experiments.

A

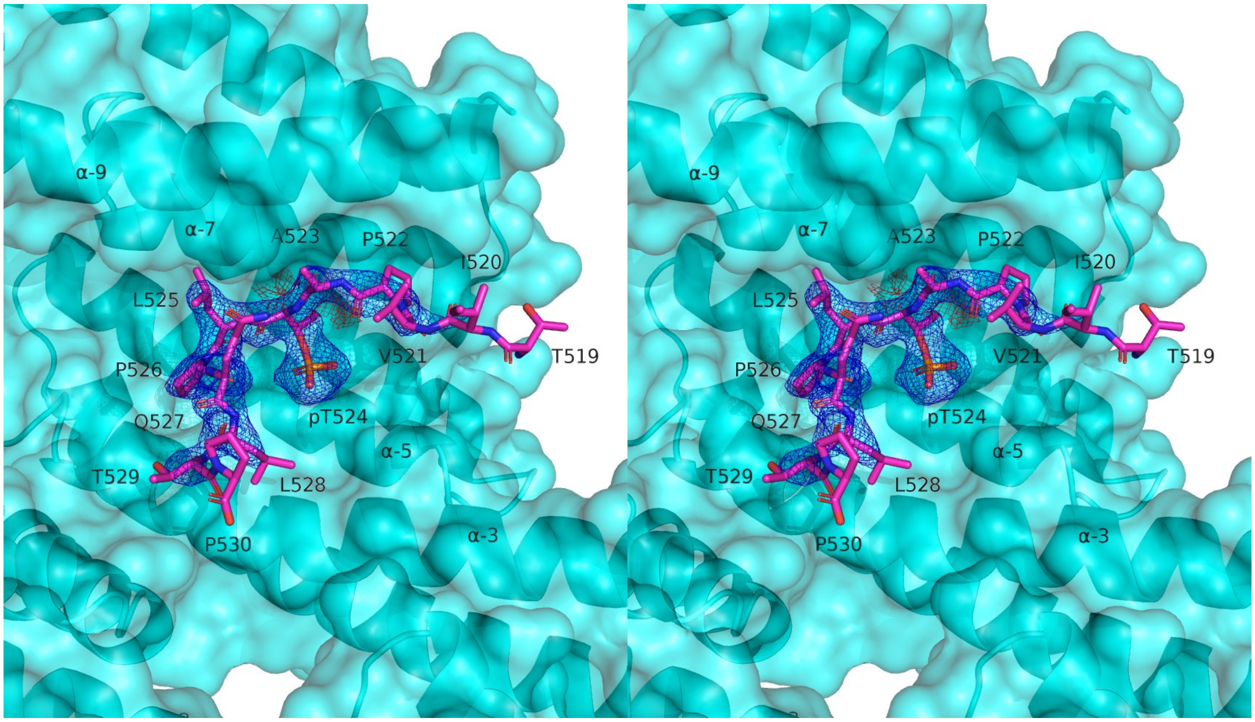
α-9
α-7
A523
P522
I520
L525
V521
T519
P526
pT524
Q527
α-5
T529
L528
α-3
P530

B

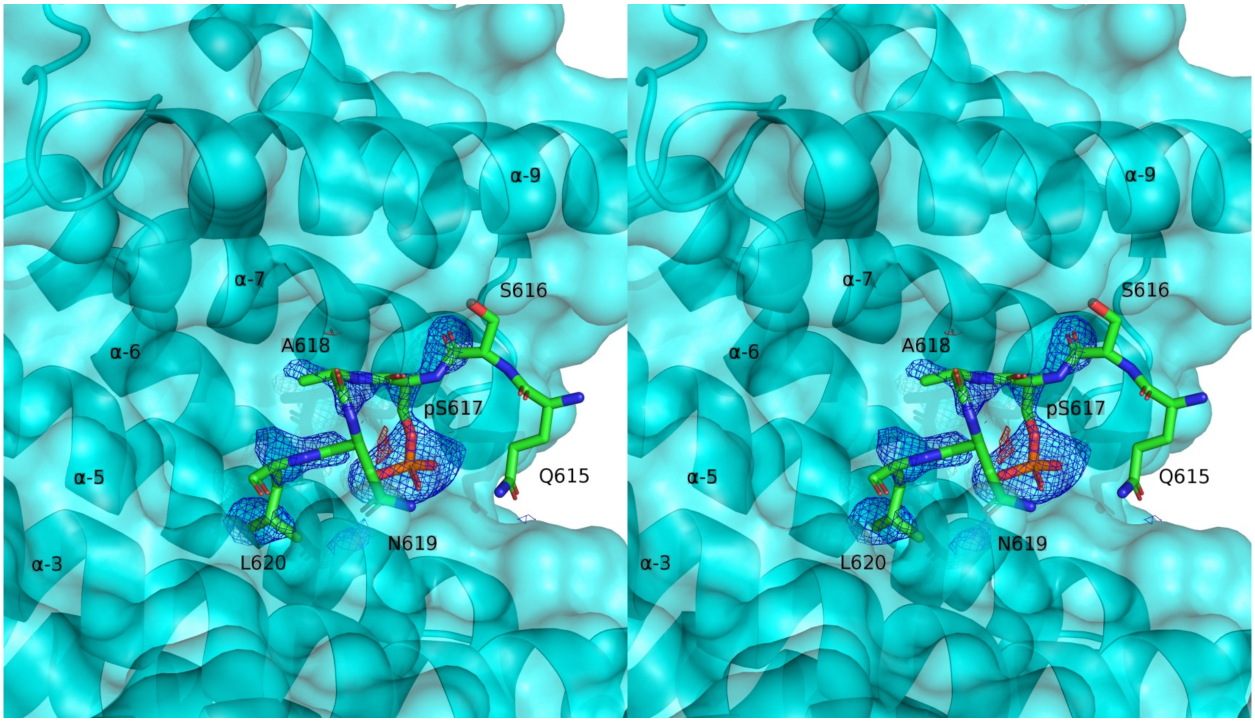
α-9
α-7
S616
α-6
A618
pS617
Q615
α-5
N619
α-3
L620

C

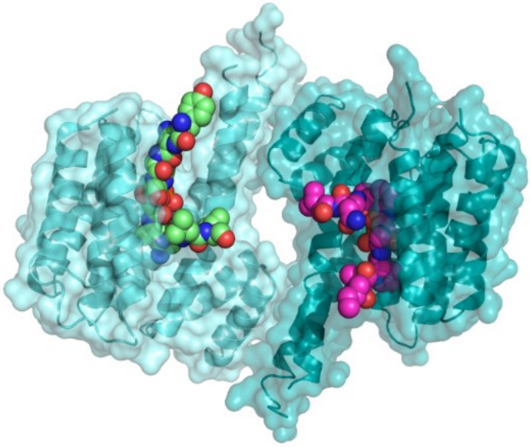

D

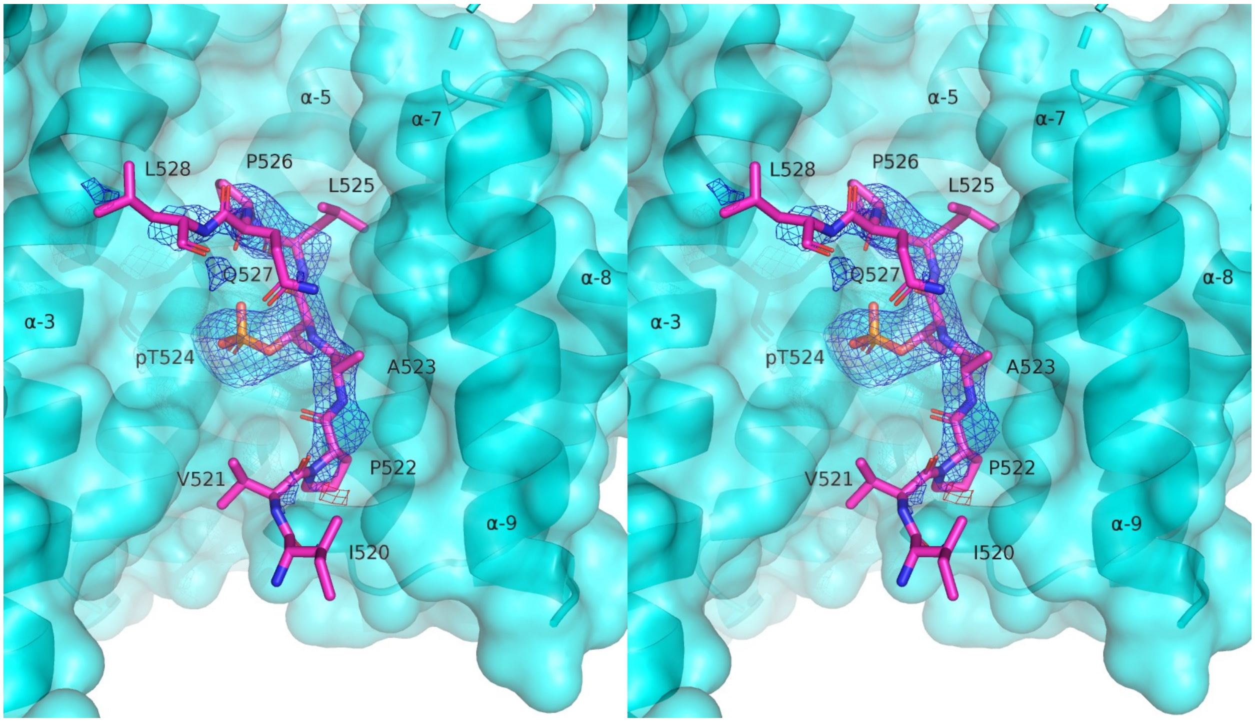

E

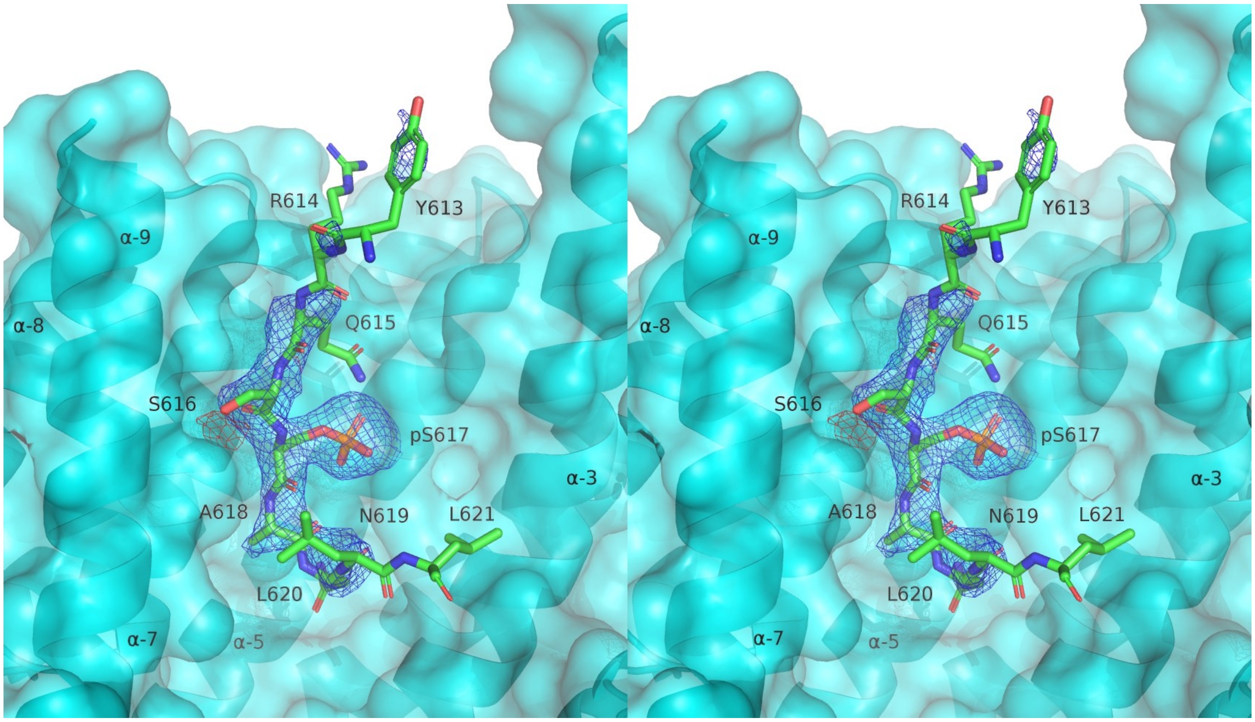

Supporting Fig. S2. GR peptides co-crystallized with 14-3-3ζ in stereo view. (**A**), Omit map of GR_pT524 (magenta, red and blue sticks) bound to 14-3-3ζ (aquamarine ribbons) with positive and negative regions of the map (F_o–Fc, contoured at ±3 σ and within 2Å of GR peptide). (**B**), Omit map of GR_pS617 (green, red and blue sticks) bound to 14-3-3ζ (aquamarine ribbons) with positive and negative regions of the map (F_o–Fc, contoured at ±3 σ and within 2Å of GR peptide). (**C**), Surface representation of 14-3-3ζ dimer (aquamarine solid surface and ribbons) complexed with GR_pT524-pS617, residues 520-528 are depicted in pink and residues 613-621 in green. (**D**), Omit map of GR_pT524_pS617 residues 520-528 (magenta, red and blue sticks) bound to 14-3-3ζ (aquamarine ribbons) with positive and negative regions of the map (F_o–Fc, contoured at ±3 σ and within 2Å of GR peptide). (**E**), Omit map of GR_pT524_pS617 residues 613-621 (green, red and blue sticks) bound to 14-3-3ζ (aquamarine ribbons) with positive and negative regions of the map (F_o–Fc, contoured at ±3 σ and within 2Å of GR peptide).

List of Kinases for the KinaseFinder – Ser/Thr protein kinases

ProQinase TARGETING CANCER

No.	Kinase	No.	Kinase	No.	Kinase	No.	Kinase	No.	Kinase	No.	Kinase
1	ACV-R1	45	CDK7CycH	89	GSK3-beta	133	MKK7	177	PKC-gamma	221	STK17A
2	ACV-R1B	46	CDK8/CycC	90	HIPK1	134	MKNK1	178	PKC-delta	222	STK23
3	ACV-R2A	47	CDK9/CycK	91	HIPK2	135	MKNK2	179	PKC-epsilon	223	STK25
4	ACV-R2B	48	CDK9/CycT	92	HIPK3	136	MLK4	180	PKC-eta	224	STK33
5	ACV-RL1	49	CHK1	93	HIPK4	137	MST1	181	PKC-iota	225	STK39
6	AKT1	50	CHK2	94	HRI	138	MST2	182	PKC-mu	226	TAOK2
7	AKT2	51	CK1-alpha1	95	IKK-alpha	139	MST3	183	PKC-nu	227	TAOK3
8	AKT3	52	CK1-gamma1	96	IKK-beta	140	MST4	184	PKC-theta	228	TBK1
9	AMPK-alpha1	53	CK1-gamma2	97	IKK-epsilon	141	mTOR	185	PKC-zeta	229	TGFB-R1
10	ARK5	54	CK1-gamma3	98	IRAK1	142	MYLK	186	PKC-zeta wt aa184-592	230	TGFB-R2
11	ASK1	55	CK1-delta	99	IRAK4	143	MYLK2	187	PKMYT1	231	TLK1
12	Aurora-A	56	CK1-epsilon	100	JNK1	144	MYLK3	188	PKN3	232	TLK2
13	Aurora-B	57	CK2-alpha1	101	JNK2	145	NEK1	189	PLK1	233	TSF1
14	Aurora-C	58	CK2-alpha2	102	JNK3	146	NEK11	190	PLK3	234	TSK2
15	BMPR1A	59	CLK1	103	LIMK1	147	NEK2	191	PRK1	235	TSSK1
16	B-RAF wt	60	CLK2	104	LIMK2	148	NEK3	192	PRK2	236	TTBK1
17	B-RAF VE	61	CLK3	105	LRRK G2019S	149	NEK4	193	PRKD2	237	TTBK2
18	BRSK1	62	CLK4	106	LRRK2 I2020T	150	NEK6	194	PRKG1	238	TTK
19	BRSK2	63	COT	107	LRRK2 R1441C	151	NEK7	195	PRKG2	239	VRK1
20	BUB1B	64	DAPK1	108	LRRK2 wt	152	NEK9	196	PRKX	240	VRK2
21	CAMK1D	65	DAPK2	109	MAP3K1	153	NIK	197	RAF1 YDYD	241	WEE1
22	CAMK2A	66	DAPK3	110	MAP3K10	154	NLK	198	RIPK2	242	WNK1
23	CAMK2B	67	DCAMKL2	111	MAP3K11	155	p38-alpha	199	RIPK5	243	WNK2
24	CAMK2D	68	DMPK	112	MAP3K7/MAP3K7IP1	156	p38-beta	200	ROCK1	244	WNK3
25	CAMK2G	69	DNA-PK	113	MAP3K9	157	p38-gamma	201	ROCK2	245	ZAK
26	CAMK4	70	DYRK1A	114	MAP4K2	158	p38-delta	202	RPS6KA1		
27	CAMKK1	71	DYRK1B	115	MAP4K4	159	PAK1	203	RPS6KA2		
28	CAMKK2	72	DYRK2	116	MAP4K5	160	PAK2	204	RPS6KA3		
29	CDC42BPA	73	DYRK3	117	MAPKAPK2	161	PAK3	205	RPS6KA4		
30	CDC42BPB	74	DYRK4	118	MAPKAPK3	162	PAK4	206	RPS6KA5		
31	CDC7/ASK	75	EEF2K	119	MAPKAPK5	163	PAK6	207	RPS6KA6		
32	CDK1/CycA	76	EIF2AK2	120	MARK1	164	PAK7	208	S6K		
33	CDK1/CycE	77	EIF2AK3	121	MARK2	165	PASK	209	S6K-beta		
34	CDK16/CycK	78	ERK1	122	MARK3	166	PBK	210	SAK		
35	CDK1CycB1	79	ERK2	123	MARK4	167	PDK1	211	SGK1		
36	CDK2/CycA	80	ERK7	124	MEK1 wt	168	PHKG1	212	SGK2		
37	CDK2/CycE	81	GRK2	125	MEK2	169	PHKG2	213	SGK3		
38	CDK3/CycE	82	GRK3	126	MEK5	170	PIM1	214	SIK1 aa1-350		
39	CDK4/CycD1	83	GRK4	127	MEKK2	171	PIM2	215	SIK2		
40	CDK4/CycD3	84	GRK5	128	MEKK3	172	PIM3	216	SLK		
41	CDK5/p25NCK	85	GRK6	129	MELK	173	PKA	217	SNARK		
42	CDK5/p35NCK	86	GRK7	130	MINK1	174	PKC-alpha	218	SNK		
43	CDK6/CycD1	87	GSG2	131	MKK4	175	PKC-beta1	219	SRPK1		
44	CDK6/CycD3	88	GSK3-alpha	132	MKK6SDTD	176	PKC-beta2	220	SRPK2		

Protein Kinases

info@proqinase.com

List of Kinases for the KinaseFinder – Ser/Thr protein kinases | V.001

Supporting Fig. S3. List of the 245 serine/threonine kinases tested in the initial kinase screening.

Serine/ Threonine Kinases #1a

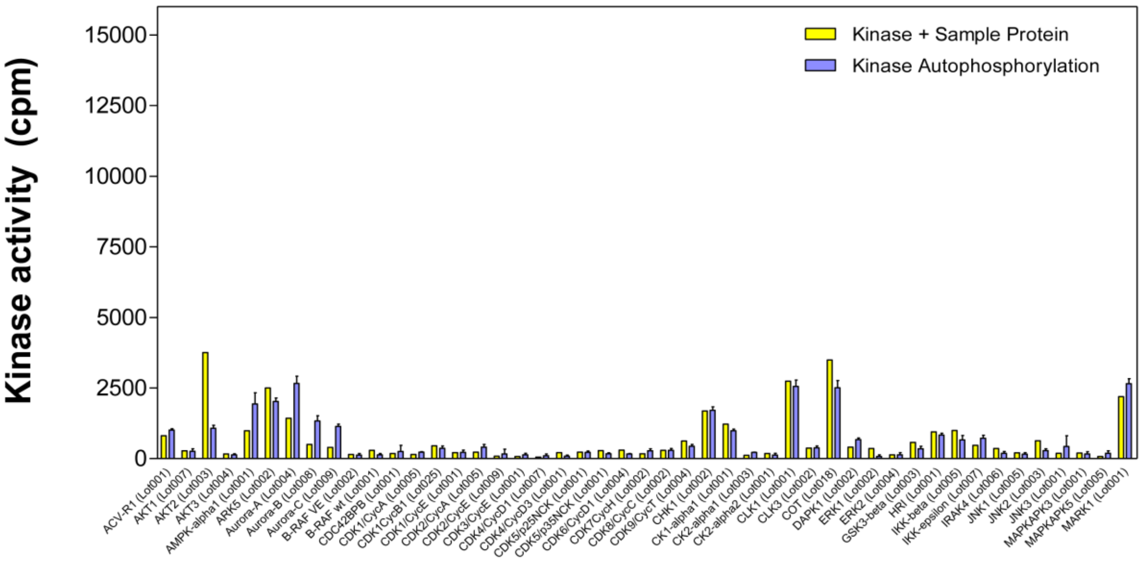

Serine/ Threonine Kinases #1b

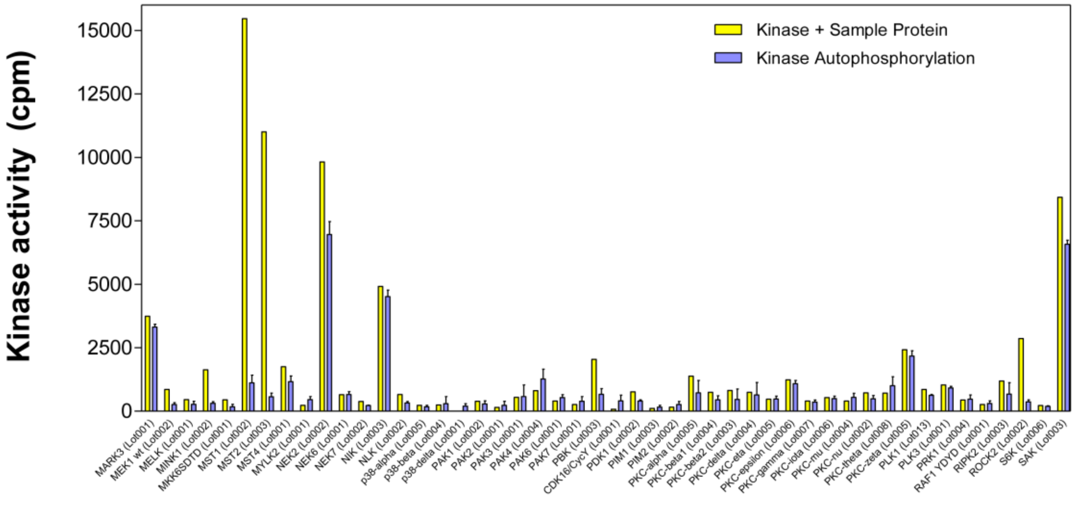

Serine/ Threonine Kinases #2a

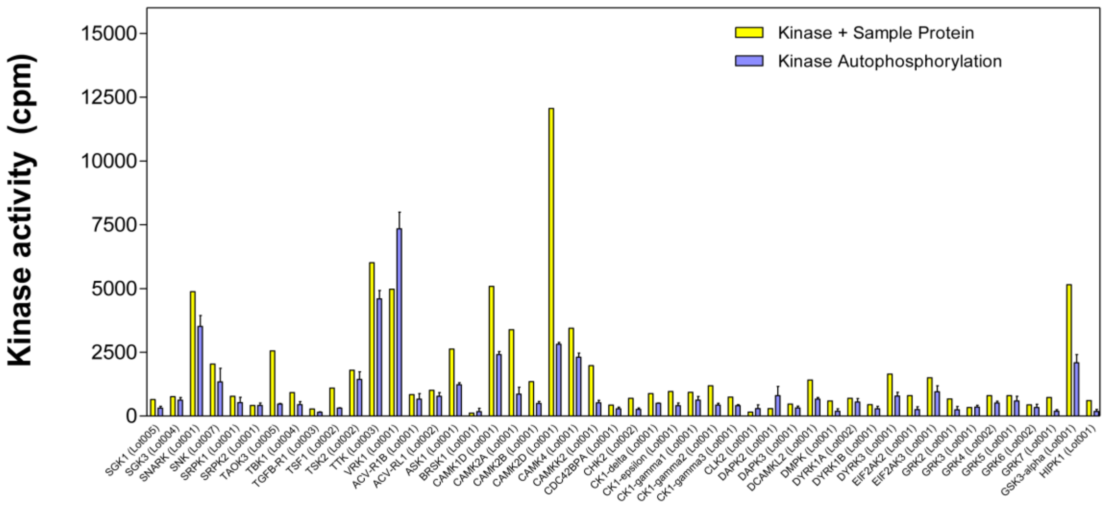

Serine/ Threonine Kinases #2b

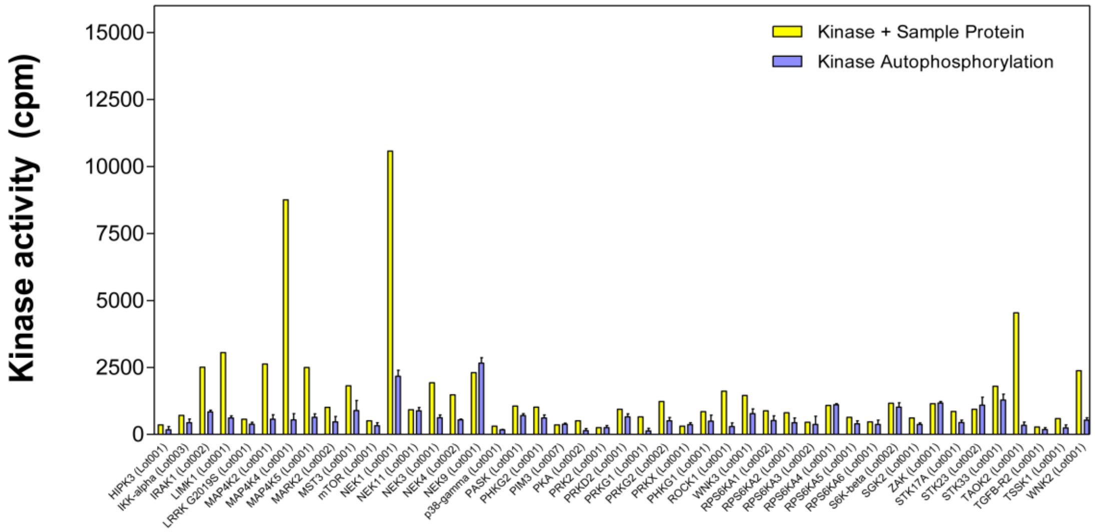

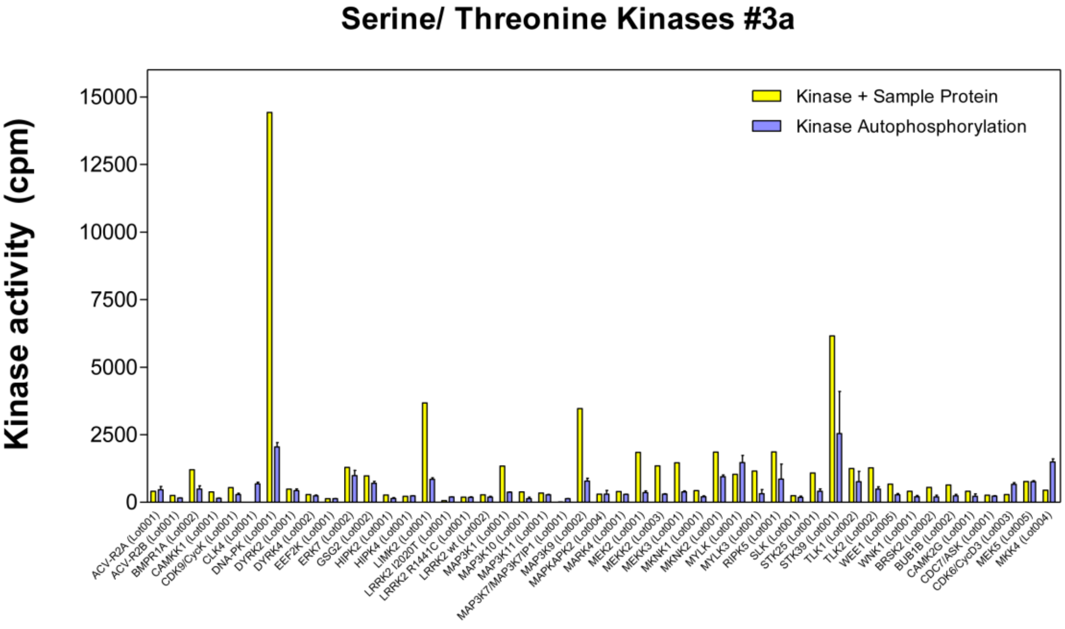

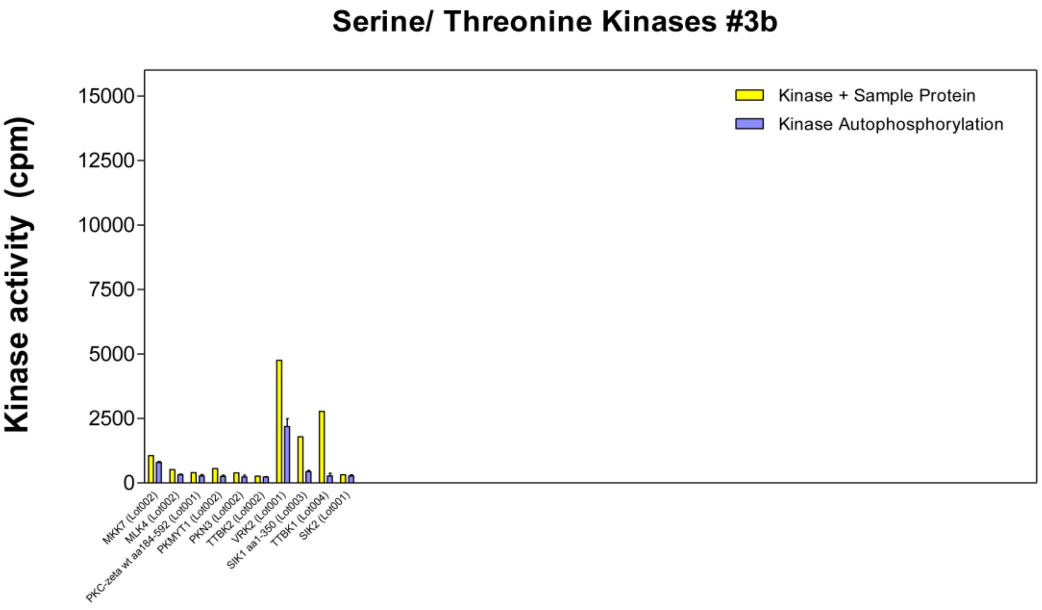

Supporting Fig. S4. Activity of 245 serine/threonine kinases with GR LBD. Corrected activity values (raw value minus sample protein background) with GR LBD at one concentration in the 245 serine/threonine protein kinase assay. Kinase with substrate is depicted in yellow and kinase apparent autophosphorylation in blue.

A

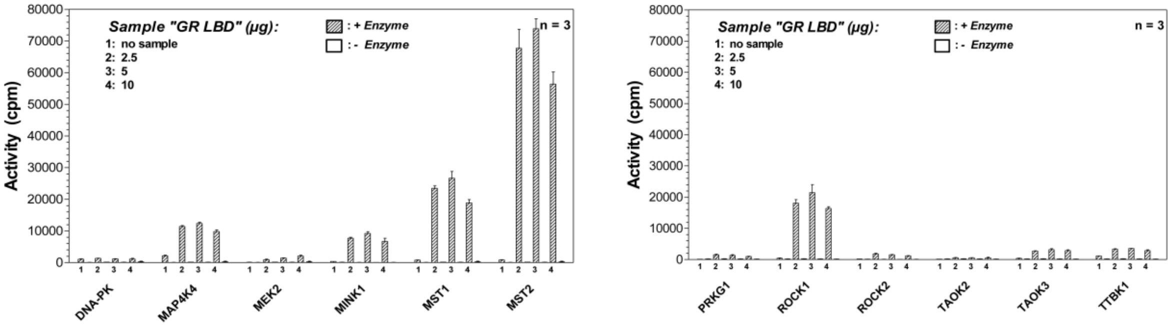

B

	Enzyme	+		-		+		-		+		-		+		-	
	**Sample (µg/50µL)**	**0.00**		**0.00**		**2.50**		**2.50**		**5.00**		**5.00**		**10.00**		**10.00**	
	**Enzyme (ng/50 µL)**	**Mean**	**SD**	**Mean**	**SD**	**Mean**	**SD**	**Mean**	**SD**	**Mean**	**SD**	**Mean**	**SD**	**Mean**	**SD**	**Mean**	**SD**
**DNA-PK**	20	1066	113	64	35	1292	77	122	15	1141	83	105	24	1125	231	228	242
**MAP4K4**	50	2172	184	64	35	11413	265	122	15	12338	407	105	24	9824	554	228	242
**MEK2**	50	114	19	64	35	907	188	122	15	1420	101	105	24	2084	266	228	242
**MINK1**	10	358	16	113	17	7669	326	69	28	9185	572	53	19	6685	1017	71	34
**MST1**	5	758	63	64	35	23494	744	122	15	26682	2122	105	24	18850	1042	228	242
**MST2**	10	773	99	64	35	67752	5956	122	15	73858	3161	105	24	56369	3882	228	242
**PRKG1**	12.5	87	33	177	70	1549	152	221	96	1364	236	176	39	991	87	145	27
**ROCK1**	4	415	142	177	70	18051	1164	221	96	21444	2560	176	39	16371	570	145	27
**ROCK2**	2.5	178	21	113	17	1774	269	69	28	1564	73	53	19	1180	112	71	34
**TAOK2**	10	132	23	177	70	603	49	221	96	591	17	176	39	559	191	145	27
**TAOK3**	50	363	81	177	70	2702	108	221	96	3154	368	176	39	2835	293	145	27
**TTBK1**	10	1144	42	177	70	3339	152	221	96	3555	67	176	39	2891	224	145	27

Supporting Fig. S5. Results from the hit confirmation assay of the 12 selected kinases with GR LBD. (**A**), Phosphorylation profile of GR LBD displayed as a bar graph. (**B**), Activity values by the selected kinases with GR LBD. The radiometric filter assays were performed in triplicate at three substrate concentrations (2.5, 5 and 10 µg/50 µl final assay concentration). Kinase activity in cpm. SD means standard deviation.

A

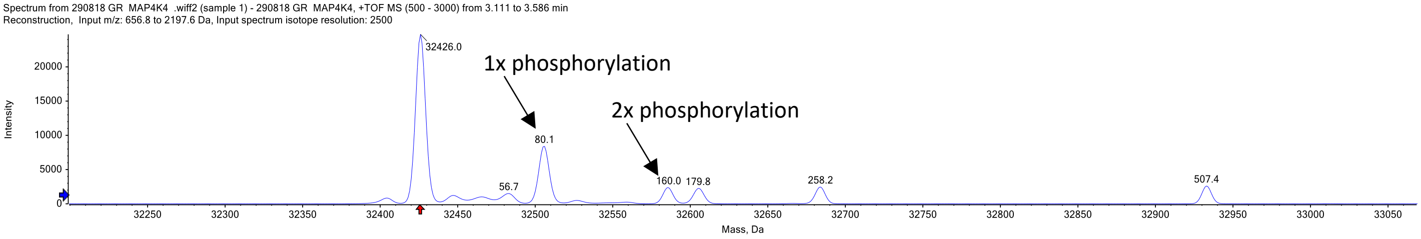

1 MHHHHHHGGE NLYFQGNPGN KTIVPATLPQ LTPTLVSLLE VIEPEVLYAG

51 YDSSVPDSTW RIMTTLNMLG GRQVIAAVKW AKAIPGFRNL HLDDQMTLLQ

101 YSWMSLMAFA LGWRSYRQSS ANLLCFAPDL IINEQRMTLP CMYDQCKHML

151 YVSSELHRLQ VSYEEYLCMK TLLLLSSVPK DGLKSQELFD EIRMTYIKEL

201 GKAIVKREGN SSQNWQRFYQ LTKLLDSMHE VVENLLNYCF QTFLDKTMSI

251 EFPEMLAEII TNQIPKYSNG NIKKLLFHQK

B

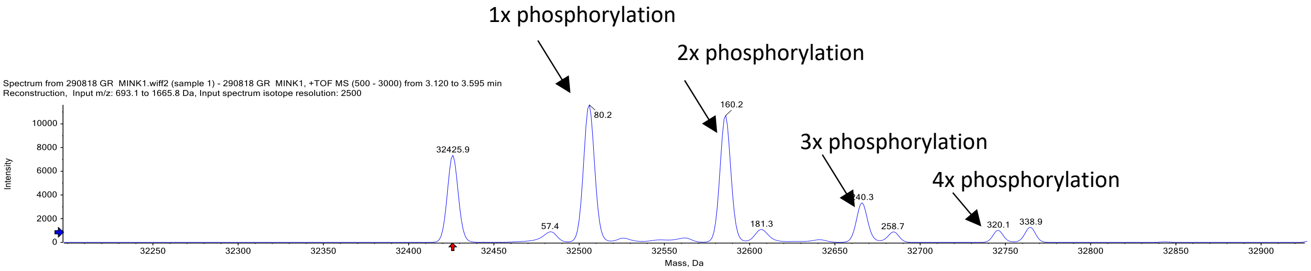

1 MHHHHHHGGE NLYFQGNPGN KTIVPATLPQ LTPTLVSLLE VIEPEVLYAG

51 YDSSVPDSTW RIMTTLNMLG GRQVIAAVKW AKAIPGFRNL HLDDQMTLLQ

101 YSWMSLMAFA LGWRSYRQSS ANLLCFAPDL IINEQRMTLP CMYDQCKHML

151 YVSSELHRLQ VSYEEYLCMK TLLLLSSVPK DGLKSQELFD EIRMTYIKEL

201 GKAIVKREGN SSQNWQRFYQ LTKLLDSMHE VVENLLNYCF QTFLDKTMSI

251 EFPEMLAEII TNQIPKYSNG NIKKLLFHQK

C

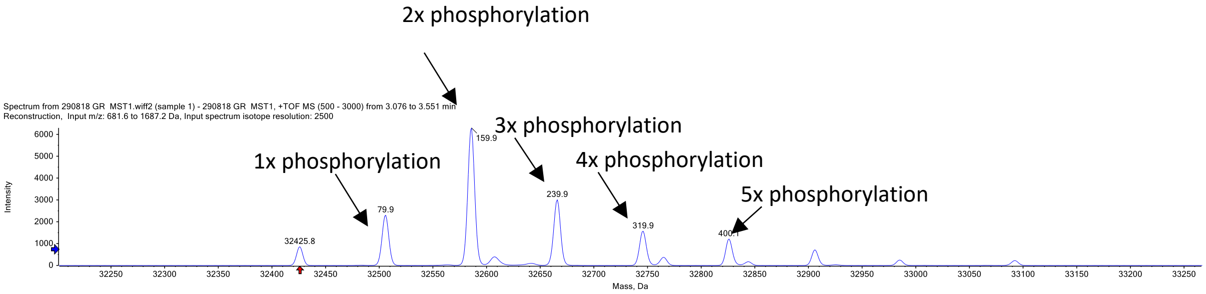

1 MHHHHHHGGE NLYFQGNPGN KTIVPATLPQ LTPTLVSLLE VIEPEVLYAG

51 YDSSVPDSTW RIMTTLNMLG GRQVIAAVKW AKAIPGFRNL HLDDQMTLLQ

101 YSWMSLMAFA LGWRSYRQSS ANLLCFAPDL IINEQRMTLP CMYDQCKHML

151 YVSSELHRLQ VSYEEYLCMK TLLLLSSVPK DGLKSQELFD EIRMTYIKEL

201 GKAIVKREGN SSQNWQRFYQ LTKLLDSMHE VVENLLNYCF QTFLDKTMSI

251 EFPEMLAEII TNQIPKYSNG NIKKLLFHQK

D

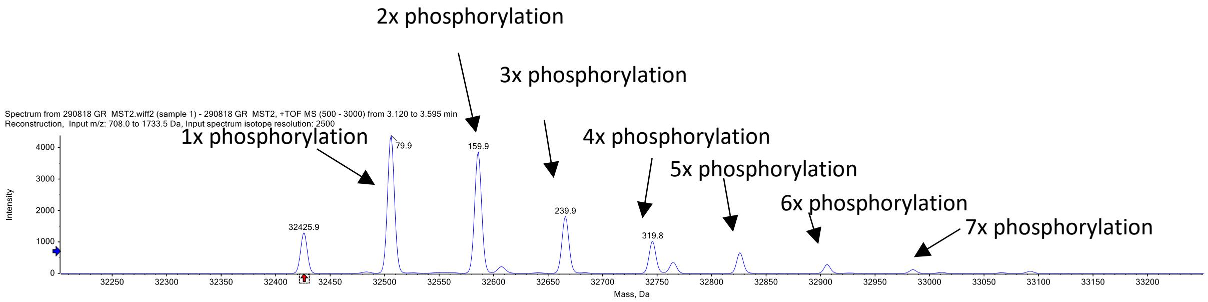

1 MHHHHHHGGE NLYFQGNPGN KTIVPATLPQ LTPTLVSLLE VIEPEVLYAG

51 YDSSVPDSTW RIMTTLNMLG GRQVIAAVKW AKAIPGFRNL HLDDQMTLLQ

101 YSWMSLMAFA LGWRSYRQSS ANLLCFAPDL IINEQRMTLP CMYDQCKHML

151 YVSSELHRLQ VSYEEYLCMK TLLLLSSVPK DGLKSQELFD EIRMTYIKEL

201 GKAIVKREGN SSQNWQRFYQ LTKLLDSMHE VVENLLNYCF QTFLDKTMSI

251 EFPEMLAEII TNQIPKYSNG NIKKLLFHQK

E

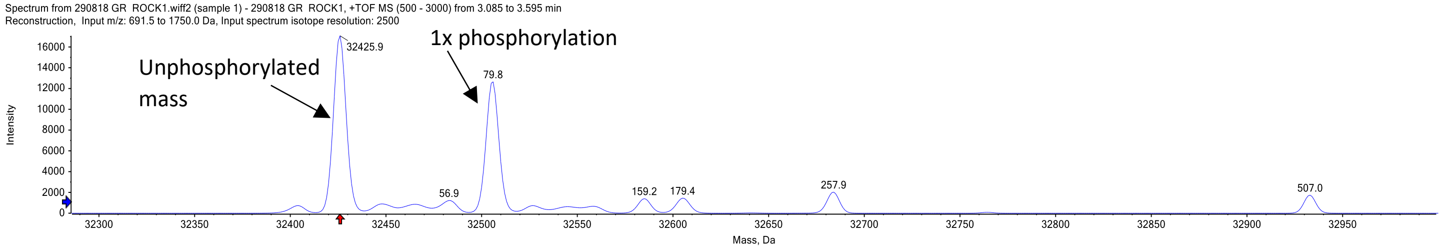

1 MHHHHHHGGE NLYFQGNPGN KTIVPATLPQ LTPTLVSLLE VIEPEVLYAG

51 YDSSVPDSTW RIMTTLNMLG GRQVIAAVKW AKAIPGFRNL HLDDQMTLLQ

101 YSWMSLMAFA LGWRSYRQSS ANLLCFAPDL IINEQRMTLP CMYDQCKHML

151 YVSSELHRLQ VSYEEYLCMK TLLLLSSVPK DGLKSQELFD EIRMTYIKEL

201 GKAIVKREGN SSQNWQRFYQ LTKLLDSMHE VVENLLNYCF QTFLDKTMSI

251 EFPEMLAEII TNQIPKYSNG NIKKLLFHQK

Supporting Fig. S6. MS spectra and peptide mapping of GR LBD phosphorylated by 5 kinases. (**A**), MAP4K4. GR LBD was unphosphorylated (61%), monophosphorylated (27%) and diphosphorylated (12%). Phosphorylations detected at T519 and T562. (**B**), MINK1. 20% was unphosphorylated, 32% had one site, 35% two sites and 13% three sites phosphorylated. Phosphorylation sites identified at T524, T562 and weakly at T635. (**C**), MST1. Only 5% of the protein was unphosphorylated, 14% had one phosphorylation, 33% 2, 15% 3, 11% 4, 9% 5, 7% 6 and 6% had 7. Phosphorylations found at: T562, T668, S682 and S746. Possible phosphorylations at T524, T594 and T635. (**D**), MST2. GR LBD showed 6 phosphorylation sites, possibly 7. 8% was unphosphorylated, 29% monophosphorylated, 25% twice, 12% 3 times, 9% 4 times, 7% 5 times, 6% 6 times and 4% 7 times. Phosphorylations detected at T562, T668, S682 and S746. Weaker indications of phosphorylations as T519, T556 and T635. (**E**), ROCK1. 54% of the protein was unphosphorylated and 46% had one phosphorylation. Phosphorylation sites detected at T519 and S617. The amount of each phosphorylation state was estimated using the areas under the trace. Sequence coverage is depicted in red and phosphorylation sites are highlighted in yellow.

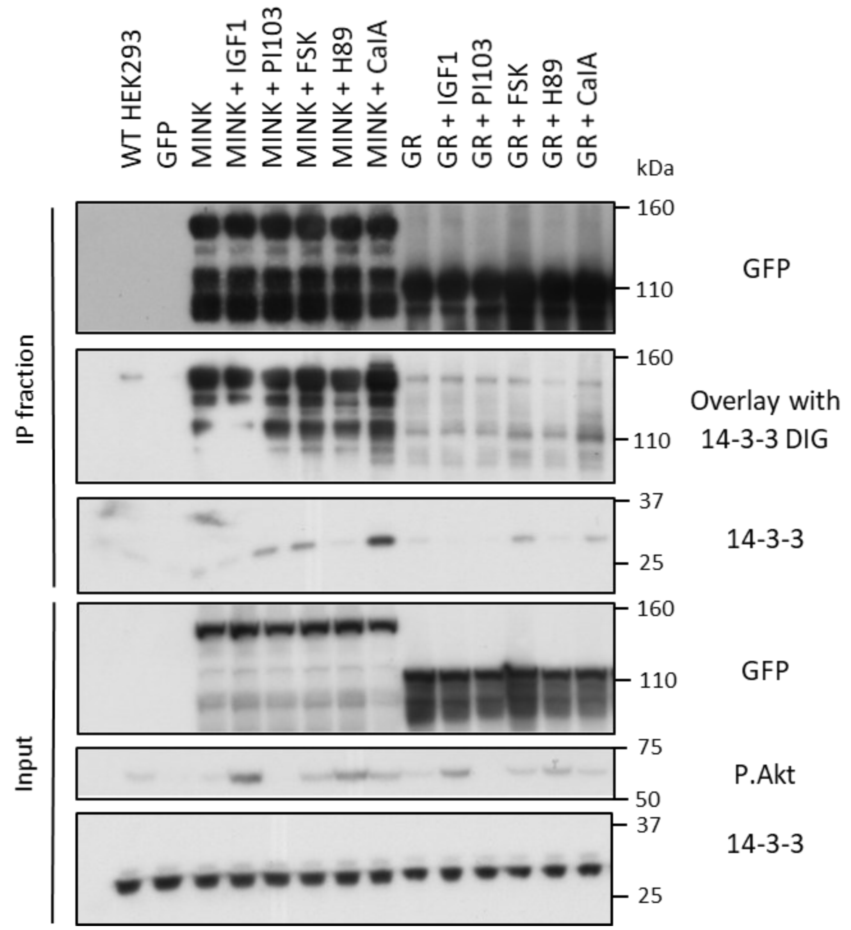

Supporting Fig. S7. Initial Co-IP assay revealing the same stimuli trigger the GR–14-3-3 interaction as those that lead to phosphorylation of MINK1. HEK293 cells were transiently transfected with GFP-MINK1 or GFP-GR plasmids followed by overnight starvation and by a panel of stimulation. Cell lysates were immunoprecipitated with GFP-Trap beads. GFP-MINK1 and GFP-GR were detected using anti-GFP antibody, MINK1-associated 14-3-3 and GR-associated 14-3-3 were detected using anti-pan 14-3-3 antibody and far-western blotting overlay was done by incubation of the MINK1-containing membrane and GR-containing membrane with BMH1-BMH2-DIG and subsequent detection of MINK1 or GR-bound 14-3-3 proteins using anti-DIG antibody.

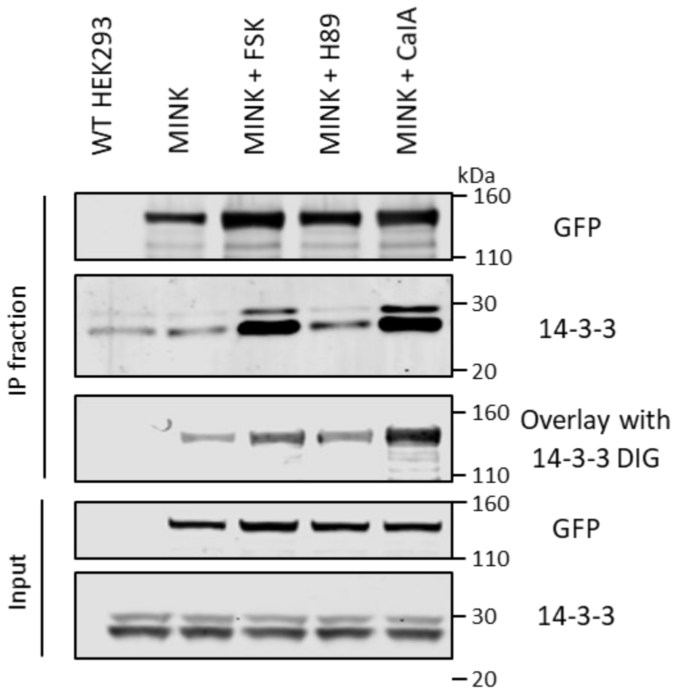

Supporting Fig. S8. MINK1–14-3-3 protein–protein interaction upon forskolin and calyculin A cell stimulation. HEK293 cells were transiently transfected with GFP-MINK1 plasmid followed by overnight starvation and by a panel of stimuli. Cell lysates were immunoprecipitated with GFP-Trap beads. GFP-MINK1 was detected using anti-GFP antibody, MINK1-associated 14-3-3 was detected using anti-pan 14-3-3 antibody and far-western blotting overlay was done by incubation of the MINK1-containing membrane with BMH1-BMH2-DIG and subsequent detection of MINK1-bound 14-3-3 proteins using anti-DIG antibody.

A

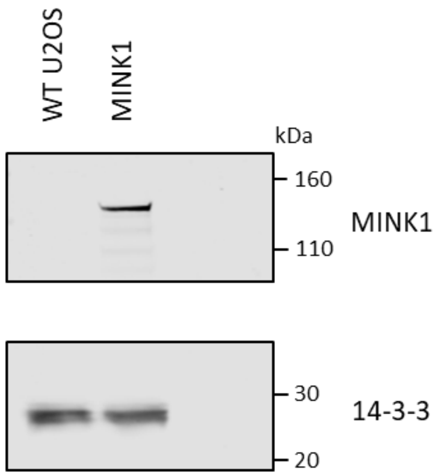

B

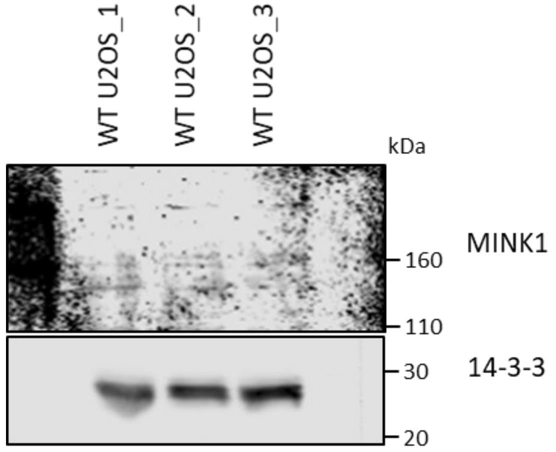

Supporting Fig. S9. Very low endogenous expression of MINK1 in U2OS cells. (**A**), Proteins from the cell lysates of U2OS cells and U2OS cells transiently transfected with GFP-MINK1 plasmid were resolved on a NuPAGE and transferred to a membrane. MINK1 was detected using anti-MINK1 antibody. (**B**), Proteins from three independent cell lysates of U2OS cells were resolved on a NuPAGE and transferred to a membrane. No endogenous MINK1 was detected using anti-MINK1 antibody even at the highest contrast offered by the Image Studio (LI-COR) software.

4. Supporting tables

Supporting Table S1. Binding affinity of the GR peptides from the alanine scan and mutations with 14-3-3ζ measured by FP

Peptide name	Sequence	PK_d with 14-3-3ζ
**GR_pT524-pS617**	K T I V P A pT L P Q L T P G G G G G R S Y R Q S pS A N L L C F	7.7
**GR_K518A**	**A** T I V P A pT L P Q L T P G G G G G R S Y R Q S pS A N L L C F	7.0
**GR_T519A**	K **A** I V P A pT L P Q L T P G G G G G R S Y R Q S pS A N L L C F	7.2
**GR_I520A**	K T **A** V P A pT L P Q L T P G G G G G R S Y R Q S pS A N L L C F	7.3
**GR_V521A**	K T I **A** P A pT L P Q L T P G G G G G R S Y R Q S pS A N L L C F	7.4
**GR_P522A**	K T I V **A** A pT L P Q L T P G G G G G R S Y R Q S pS A N L L C F	7.2
**GR_T524A-pS617**	K T I V P A **A** L P Q L T P G G G G G R S Y R Q S pS A N L L C F	3.9
**GR_L525A**	K T I V P A pT **A** P Q L T P G G G G G R S Y R Q S pS A N L L C F	7.2
**GR_P526A**	K T I V P A pT L **A** Q L T P G G G G G R S Y R Q S pS A N L L C F	5.9
**GR_Q527A**	K T I V P A pT L P **A** L T P G G G G G R S Y R Q S pS A N L L C F	7.5
**GR_L528A**	K T I V P A pT L P Q **A** T P G G G G G R S Y R Q S pS A N L L C F	7.3
**GR_T529A**	K T I V P A pT L P Q L **A** P G G G G G R S Y R Q S pS A N L L C F	7.3
**GR_P530A**	K T I V P A pT L P Q L T **A** G G G G G R S Y R Q S pS A N L L C F	7.5
**GR_R611A**	K T I V P A pT L P Q L T P G G G G G **A** S Y R Q S pS A N L L C F	7.3
**GR_S612A**	K T I V P A pT L P Q L T P G G G G G R **A** Y R Q S pS A N L L C F	7.5
**GR_Y613A**	K T I V P A pT L P Q L T P G G G G G R S **A** R Q S pS A N L L C F	7.1
**GR_R614A**	K T I V P A pT L P Q L T P G G G G G R S Y **A** Q S pS A N L L C F	6.7
**GR_Q615A**	K T I V P A pT L P Q L T P G G G G G R S Y R **A** S pS A N L L C F	7.4
**GR_S616A**	K T I V P A pT L P Q L T P G G G G G R S Y R Q **A** pS A N L L C F	7.2
**GR_pT524-S617A**	K T I V P A pT L P Q L T P G G G G G R S Y R Q S **A** A N L L C F	5.9
**GR_N619A**	K T I V P A pT L P Q L T P G G G G G R S Y R Q S pS A **A** L L C F	7.4
**GR_L620A**	K T I V P A pT L P Q L T P G G G G G R S Y R Q S pS A N **A** L C F	7.3
**GR_L621A**	K T I V P A pT L P Q L T P G G G G G R S Y R Q S pS A N L **A** C F	7.4
**GR_C622A**	K T I V P A pT L P Q L T P G G G G G R S Y R Q S pS A N L L **A** F	7.2
**GR_F623A**	K T I V P A pT L P Q L T P G G G G G R S Y R Q S pS A N L L C **A**	7.3
**GR_pS524-pS617**	K T I V P A **pS** L P Q L T P G G G G G R S Y R Q S pS A N L L C F	6.9

Sites of mutations are depicted in bold black. Measurements were performed as triplicates.

Supporting Table S2. Activity values by the selected kinases for hit confirmation

Kinase #	Kinase (ProQinase Lot #)	Enzyme, ng/well	Activity raw values	Kinase autophos., normalized mean n=3	Kinase autophos., SD	Substrate-BG, median n=3	Activity values, corrected (A-C)	Activity Ratio (A-C)/B
194	**PRKG1 (Lot001)**	12.5	801	130	103	145	656	**5.04**
125	**MEK2 (Lot001)**	100	1994	359	63	145	1849	**5.14**
130	**MINK1 (Lot002)**	10	1772	314	63	145	1627	**5.18**
227	**TAOK3 (Lot005)**	50	2698	469	27	145	2553	**5.45**
200	**ROCK1 (Lot001)**	4	1760	293	139	145	1615	**5.51**
69	**DNA-PK (Lot001)**	10	14574	2047	160	145	14429	**7.05**
201	**ROCK2 (Lot002)**	2.5	3007	370	80	145	2862	**7.73**
236	**TTBK1 (Lot004)**	10	2919	269	107	145	2774	**10.30**
226	**TAOK2 (Lot001)**	20	4685	342	123	145	4540	**13.28**
137	**MST1 (Lot002)**	5	15611	1117	304	145	15466	**13.84**
115	**MAP4K4 (Lot001)**	5	8905	546	232	145	8760	**16.04**
138	**MST2 (Lot003)**	10	11149	570	143	145	11004	**19.31**

All the kinases display an activity ratio above 5. A ratio value between substrate phosphorylation and apparent autophosphorylation > 3 may be considered as significant. Kinase activity in cpm. SD means standard deviation.

5. Supporting equation

Supporting Equation S1. Mass balance equation for the interaction ditopic host−guest systems

$$K_{dimer} = 2 \times K_{monomer1} \times K_{monomer2} \times EM \quad (1)$$

K_{dimer} is the affinity constant of the doubly phosphorylated peptide, $K_{monomer1}$ and $K_{monomer2}$ are the affinity constants of the single phosphorylated peptides and EM is the effective molarity.

6. GR_pT524-pS617 orientation in the crystal structure with 14-3-3ζ

We have refined GR_pT524-pS617 bound to 14-3-3ζ in the pose corresponding to an inverted U (GR_U). However, because of the relative symmetry of this GR peptide and for comparison, another structure of GR_pT524-pS617 adopting an alternative N binding pose was also refined (GR_N) (Fig. **1** and **2**). The orientation, GR_N, of GR_pT524-pS617 with respect to 14-3-3 differed from all the previous published crystal structures of a doubly phosphorylated peptide bound to 14-3-3 (PBD code 4IHL, 5D2D, 5D3E and 5D3F). Modelling studies and scoring were performed to seek for evidence of a preferred orientation. Both GR peptide orientations, GR_U and GR_N, are compatible with the unobserved pentaglycine section and the linker residues could be constructed using the ROSETTA remodel application (Fig. **3** and **4**). The B-factors from the C□ atoms of the bound peptides were calculated for both orientations and were found to be higher than the B-factors from previous published structures, in-line with the resolution. The average values of the corresponding GR peptides of the preferred (GR_U) and alternative (GR_N) structures were comparable (Fig **5** and Table **1**). ROSETTA was used to score the structures of each GR monophosphorylated peptide–14-3-3 complex without structure refinement (score function ref2015, www.rosettacommons.org/docs/latest/rosetta_basics/scoring/score-types). The positive values denoted unfavorable scorings and the trend with respect to resolution was as expected. Using this scoring function, the average score for the GR monophosphorylated peptide section centred on pS617 was lower in the GR_N orientation structure than in the GR_U orientation. Nevertheless, the phosphorylated serine showed poorer internal conformation energy in the GR_N orientation compared to the GR_U orientation (Fig **5** and Table **2**). Together, these data do not provide sufficient evidence to rule out the GR_N orientation in an unambiguous manner.

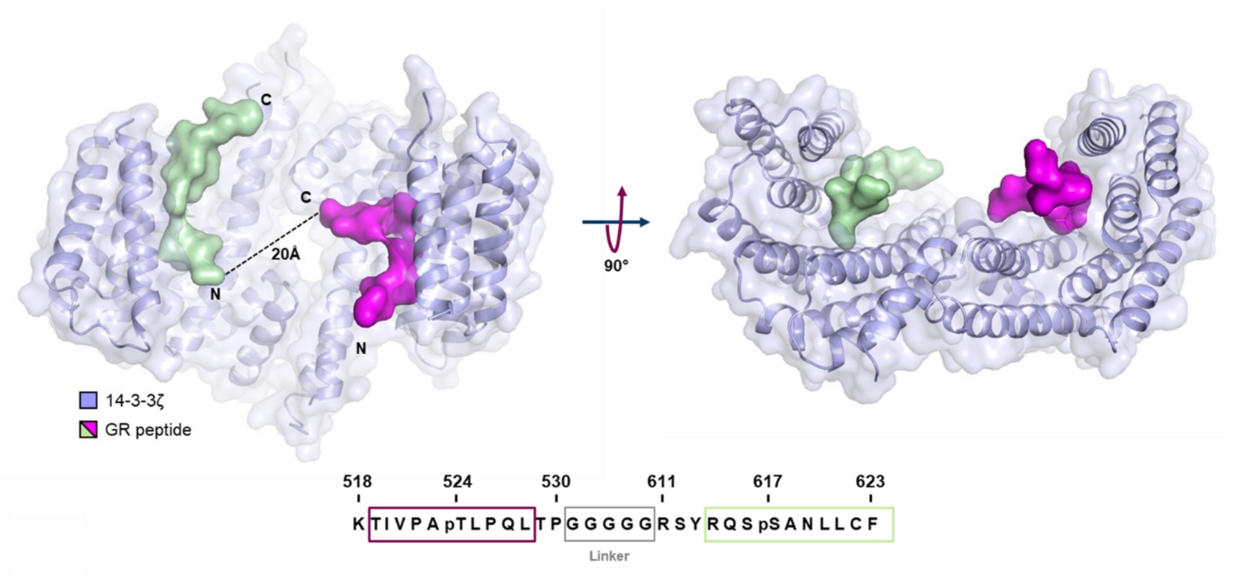

Fig. 1. Schematic representation of the alternative GR peptide binding pose, GR_N.

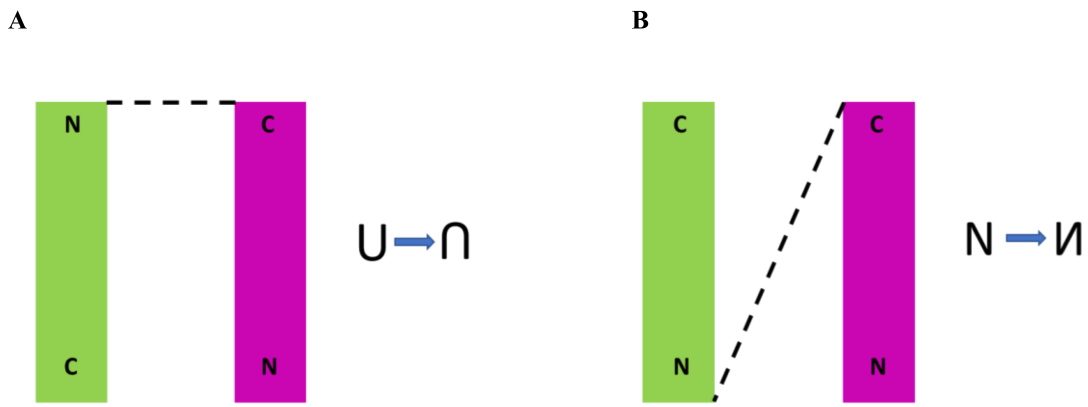

Fig. 2. Binding pose nomenclature. (**A**), Inverted U. (**B**), Alternative N binding pose.

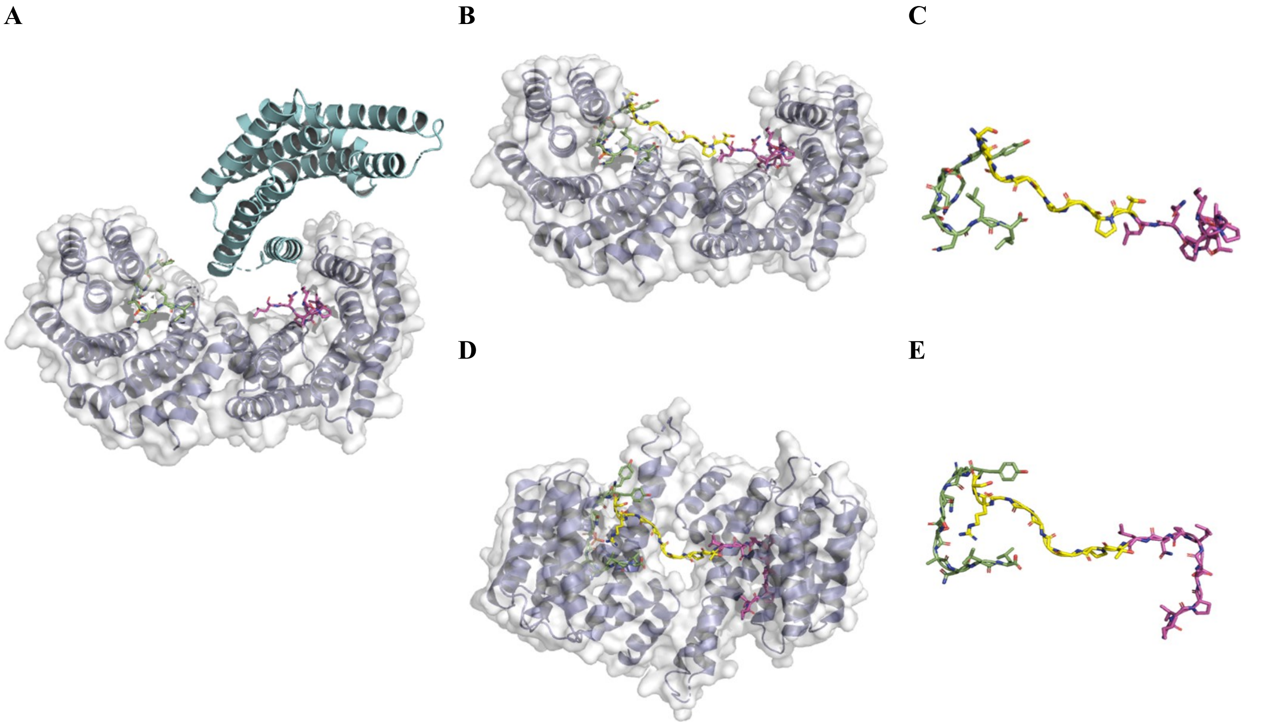

Fig. 3. GR_U Missing loop modelling. (**A**), Crystal copy included in the modelling of the missing loop. (**B**), Side view of modelled missing loop depicted in yellow. (**C**), Peptide side-view. (**D**), Top view of modelled missing loop depicted in yellow. (**E**), Peptide top-view.

A B C D E

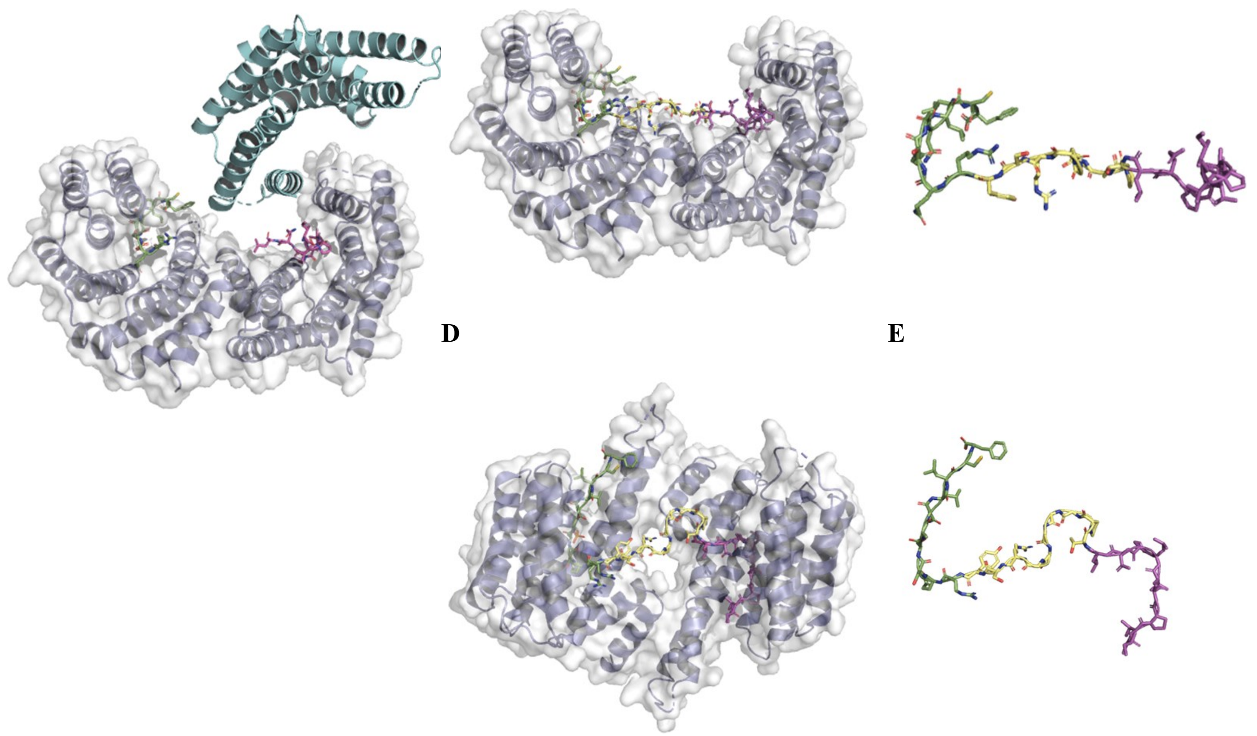

Fig. 4. GR_N Missing loop modelling. (**A**), Crystal copy included in the modelling of the missing loop. (**B**), Side view of modelled missing loop depicted in yellow. (**C**), Peptide side-view. (**D**), Top view of modelled missing loop depicted in yellow. (**E**), Peptide top-view.

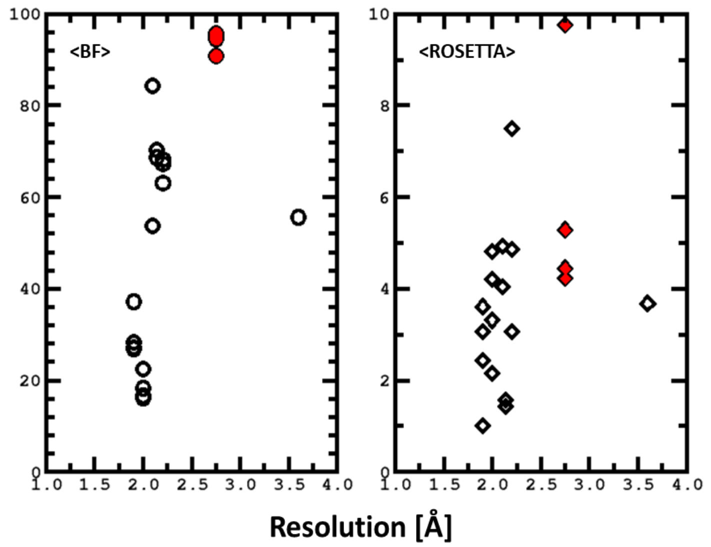

Fig. 5. Average B-factors and average ROSETTA scores versus resolution. Graphical display of the data from Table **1** and Table **2**.

Table 1: Peptide Cα B-factors

PDB	Resolution	Sequence in PDB	Protein Chain	Peptide Chain	-5	-4	-3	-2	-1	0	1	2	3	4	5	6	AV
**1A37**	3,6	--RSTsTPN---	A	P	0,00	0,00	61,55	57,21	60,41	**46,83**	44,65	64,75	54,54	0,00	0,00	0,00	55,71
**1A37**	3,6	--RSTsTPN---	B	Q	0,00	0,00	61,55	57,21	60,41	**46,83**	44,65	64,75	54,54	0,00	0,00	0,00	55,71
**1QJA**	2	DRLYHsLPA---	B	Q	0,00	30,22	20,15	14,60	12,07	**4,73**	10,00	14,56	23,65	0,00	0,00	0,00	16,25
**1QJA**	2	-RLYHsLPA---	A	R	0,00	38,90	28,17	19,33	11,52	**3,90**	6,67	7,95	17,34	0,00	0,00	0,00	16,72
**1QJB**	2	-ARSHsYPA---	A	Q	0,00	41,32	31,76	21,76	16,80	**12,02**	13,62	15,46	27,00	0,00	0,00	0,00	22,47
**1QJB**	2	--RSHsYPA---	B	S	0,00	0,00	35,62	20,29	10,48	**7,35**	10,23	16,50	28,19	0,00	0,00	0,00	18,38
**4HKC**	2,2	KRQYKsIL----	A	B	85,19	76,12	62,90	53,09	47,78	**42,72**	50,90	86,37	0,00	0,00	0,00	0,00	63,13
**4IHL**	2,2	-QRSTsTPNV--	A	P	102,98	93,79	67,13	52,12	40,22	**43,12**	48,62	67,50	89,54	0,00	0,00	0,00	67,22
**4IHL**	2,2	--HRYsTPHA--	B	P	0,00	93,41	73,07	66,33	43,61	**40,19**	55,50	81,51	92,06	0,00	0,00	0,00	68,21
**5D2D**	2,1	---RIsVISTG-	A	C	0,00	0,00	100,91	95,67	66,78	**70,98**	80,04	86,67	90,11	83,95	0,00	0,00	84,39
**5D2D**	2,1	-RRRQsVLNLMT	B	C	0,00	80,54	64,37	50,07	45,47	**37,50**	33,65	35,55	45,20	44,03	68,95	84,77	53,65
**5ULO**	2,14	---SPsFP----	A	C	0,00	0,00	0,00	84,82	73,17	**62,72**	62,82	68,36	0,00	0,00	0,00	0,00	70,38
**5ULO**	2,14	--RSPsFP----	B	D	0,00	0,00	83,38	77,29	66,02	**58,70**	58,92	68,48	0,00	0,00	0,00	0,00	68,80
**6F08**	1,9	-RRPEsAPAES-	A	Q	0,00	48,14	36,28	26,86	21,75	**16,37**	16,36	19,28	23,99	34,13	0,00	0,00	27,02
**6F08**	1,9	-RRPEsAPAE--	B	D	0,00	37,73	22,11	17,97	19,55	**15,46**	17,76	33,47	53,55	0,00	0,00	0,00	27,20
**6F08**	1,9	RRRPEsAPAE--	I	K	83,36	61,46	38,81	29,13	24,19	**20,76**	18,51	24,88	34,48	0,00	0,00	0,00	37,29
**6F08**	1,9	-RRPEsAPA---	J	N	0,00	39,34	25,67	22,54	22,25	**21,74**	24,27	34,69	36,23	0,00	0,00	0,00	28,34
**GR_U**	2,75	-IVPAtLPQL--	B	C	0,00	94,72	94,00	91,85	89,15	**86,53**	88,87	91,31	93,91	121,88	0,00	0,00	94,69
**GR_U**	2,75	-YRQSsANLL--	A	D	0,00	97,79	**94,84**	**89,93**	**84,69**	**81,32**	**84,24**	**87,49**	**88,48**	**109,49**	0,00	0,00	90,06 (90,92)
**GR_N**	2,75	-IVPAtLPQL--	B	C	0,00	95,51	94,25	92,05	89,52	**87,52**	89,74	91,90	94,57	119,97	0,00	0,00	95,00
**GR_N**	2,75	--RQSsANLLCF	A	D	0,00	0,00	**87,99**	**86,77**	**83,90**	**80,97**	**85,21**	**91,48**	**97,20**	**102,31**	105,16	136,72	89,48 (95,77)

Values from the PDB entries. Column 0 has the value of the phosphorylated residue in the binding pocket. The column AV has the average value. For GR_U and GR_N, this AV number is the average of the intervals in bold whereas the value in parenthesis corresponds to all values.

Table 2: ROSETTA residue score

PDB	Resolution	Sequence in PDB	Protein Chain	Peptide Chain	-5	-4	-3	-2	-1	0	1	2	3	4	5	6	AV
**1A37**	3,6	--RSTsTPN---	A	P	0,00	0,00	2,51	3,63	1,20	**12,51**	3,10	-0,88	0,00	0,00	0,00	0,00	3,68
**1A37**	3,6	--RSTsTPN---	B	Q	0,00	0,00	2,51	3,64	1,20	**12,50**	3,10	-0,87	0,00	0,00	0,00	0,00	3,68
**1QJA**	2	DRLYHsLPA---	B	Q	0,00	5,29	2,65	4,52	2,27	**0,80**	2,26	14,55	1,33	0,00	0,00	0,00	4,21
**1QJA**	2	-RLYHsLPA---	A	R	0,00	5,06	6,15	5,73	2,53	**1,47**	3,41	0,83	1,33	0,00	0,00	0,00	3,31
**1QJB**	2	-ARSHsYPA---	A	Q	0,00	1,34	5,06	0,88	3,29	**1,59**	3,22	0,54	1,33	0,00	0,00	0,00	2,15
**1QJB**	2	--RSHsYPA---	B	S	0,00	0,00	23,99	0,67	2,84	**1,58**	3,05	0,29	1,33	0,00	0,00	0,00	4,82
**4HKC**	2,2	KRQYKsIL----	A	B	4,55	8,99	6,71	5,39	4,91	**0,87**	4,68	2,78	0,00	0,00	0,00	0,00	4,86
**4IHL**	2,2	-QRSTsTPNV--	A	P	0,00	4,62	2,04	1,16	8,83	**1,29**	4,50	-1,44	0,87	5,80	0,00	0,00	3,07
**4IHL**	2,2	--HRYsTPHA--	B	P	0,00	0,00	12,44	12,09	6,16	**1,17**	3,37	0,20	12,93	11,56	0,00	0,00	7,49
**5D2D**	2,1	---RIsVISTG-	A	C	0,00	0,00	0,00	1,58	5,01	**1,07**	3,50	10,11	3,12	6,45	1,59	0,00	4,05
**5D2D**	2,1	-RRRQsVLNLMT	B	C	0,00	8,82	5,19	11,20	5,62	**2,77**	4,78	2,19	3,11	2,04	3,63	4,87	4,93
**5ULO**	2,14	---SPsFP----	A	C	0,00	0,00	0,00	1,95	-1,25	**1,46**	3,60	1,34	0,00	0,00	0,00	0,00	1,42
**5ULO**	2,14	--RSPsFP----	B	D	0,00	0,00	2,48	0,96	-1,91	**1,54**	3,49	2,84	0,00	0,00	0,00	0,00	1,56
**6F08**	1,9	-RRPEsAPAES-	A	Q	0,00	17,88	2,50	0,51	3,57	**2,10**	1,31	1,19	1,10	5,18	0,74	0,00	3,61
**6F08**	1,9	-RRPEsAPAE--	B	D	0,00	5,47	3,07	0,15	1,46	**1,51**	1,60	1,52	1,39	11,44	0,00	0,00	3,07
**6F08**	1,9	RRRPEsAPAE--	I	K	4,55	2,40	4,03	0,72	0,96	**2,64**	1,46	4,80	1,64	1,06	0,00	0,00	2,43
**6F08**	1,9	-RRPEsAPA---	J	N	0,00	2,02	3,60	-2,06	0,79	**1,99**	1,39	-1,09	1,33	0,00	0,00	0,00	1,00
**GR_U**	2,75	-IVPAtLPQL--	B	C	0,00	6,15	4,49	-0,39	1,43	7,84	7,24	-0,92	6,38	7,79	0,00	0,00	4,45
**GR_U**	2,75	-YRQSsANLL--	A	D	0,00	10,80	**16,12**	**22,46**	**6,04**	2,41	**-0,35**	**7,83**	**8,30**	**14,28**	0,00	0,00	9,64 (9,77)
**GR_N**	2,75	-IVPAtLPQL--	B	C	0,00	6,41	4,25	-0,61	1,49	7,57	6,20	-0,21	5,65	7,33	0,00	0,00	4,23
**GR_N**	2,75	--RQSsANLLCF	A	D	0,00	0,00	**5,88**	**4,39**	**0,95**	**16,63**	**1,67**	**1,71**	**5,82**	**6,04**	6,13	3,67	5,39 (5,29)

Values obtained directly from the PDB coordinates without refining. Column 0 has the value of the phosphorylated residue in the binding pocket. The column AV has the average value. For GR_U and GR_N, this AV number is the average of the intervals in bold whereas the value in parenthesis corresponds to all values.

7. GR peptide analysis

Whenever possible, only a fraction of crude material was purified to isolate about 10 mg of pure peptide.

FITC labelled GR_pT8:

FITC labelled GR_pT8 peptide was prepared following General Protocol for SPPS. The crude peptide was purified by reverse-phase HPLC (gradient: 5% B for 1 min, 5-19% B in 3 min, 19-24% B in 15 min, Waters CSH C18 column) to afford the desired peptide as a white fluffy solid. Yield (28 mg) in > 95% purity according to analytical UPLC. Rt 4.32 min (3-60% B in 10 min).

$[M + 2H]^{2+}$ calculated for $C_{85}H_{119}N_{20}O_{35}PS$, 1022.5; found 1022.7.

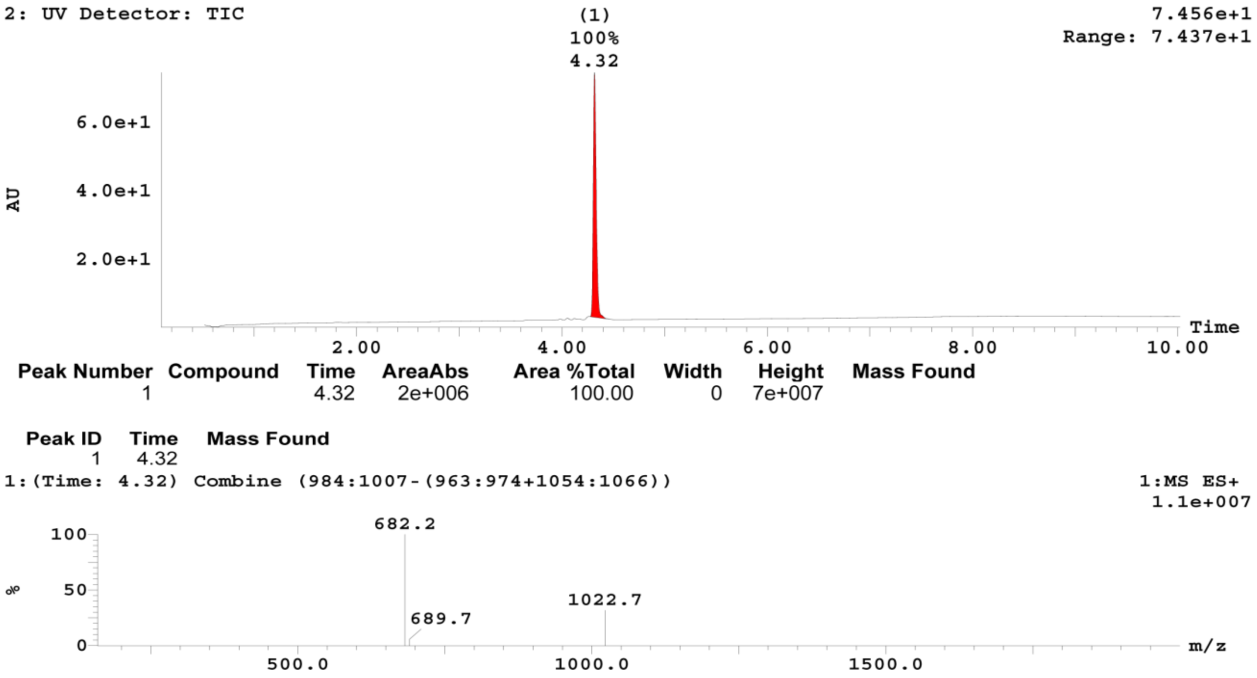

FITC labelled GR_pS45:

FITC labelled GR_pS45 peptide was prepared following General Protocol for SPPS. The crude peptide was purified by reverse-phase HPLC (gradient: 5% B for 1 min, 5-25% B in 3 min, 25-30% B in 15 min, Waters CSH C18 column) to afford the desired peptide as a white fluffy solid. Yield (11 mg) in > 95% purity according to analytical UPLC. Rt 6.00 min (3-60% B in 10 min).

$[M + 2H]^{2+}$ calculated for $C_{80}H_{117}N_{16}O_{27}PS$, 899.5; found 899.7.

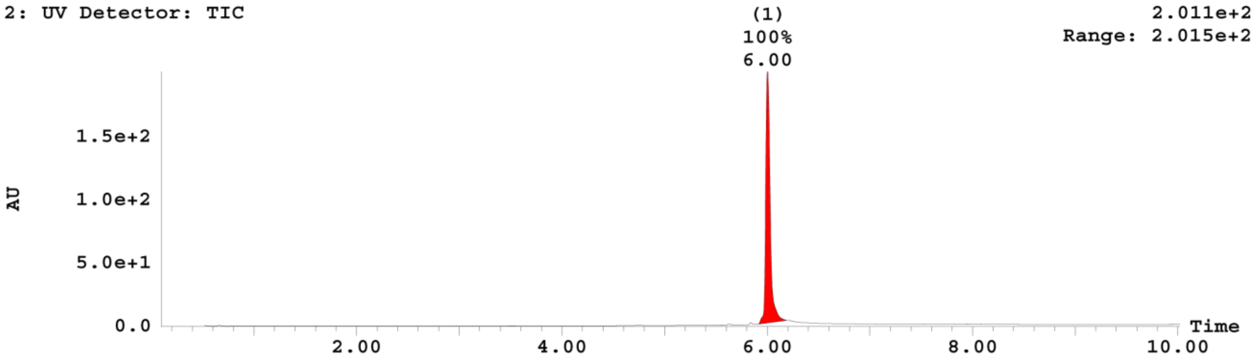

Peak Number	Compound	Time	AreaAbs	Area %Total	Width	Height	Mass Found
1		6.00	1e+007	100.00	0	2e+008	

Peak ID	Time	Mass Found
1	5.99	

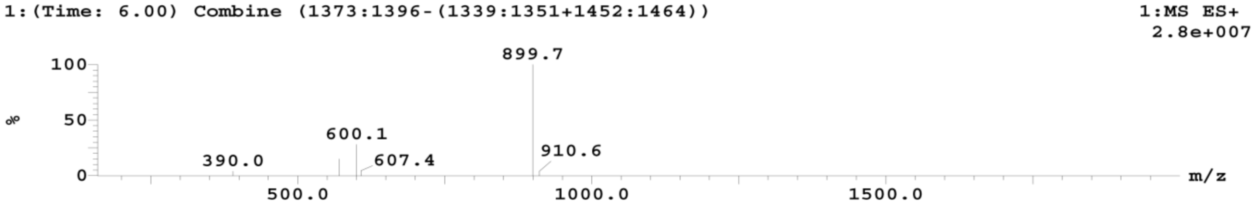

Acetylated GR_pS83:

Acetylated GR_pS83 peptide was prepared following General Protocol for SPPS. The crude peptide was purified by reverse-phase HPLC (gradient: 5% B for 1 min, 5-24% B in 3 min, 24-29% B in 15 min, Waters Xselect CSH column) to afford the desired peptide as a white fluffy solid. Yield (12 mg) in > 95% purity according to analytical UPLC. Rt 5.69 min (3-60% B in 10 min).

$[M + H]^{2+}$ calculated for $C_{63}H_{105}N_{14}O_{24}PS$, 1506.6; found 1506.4.

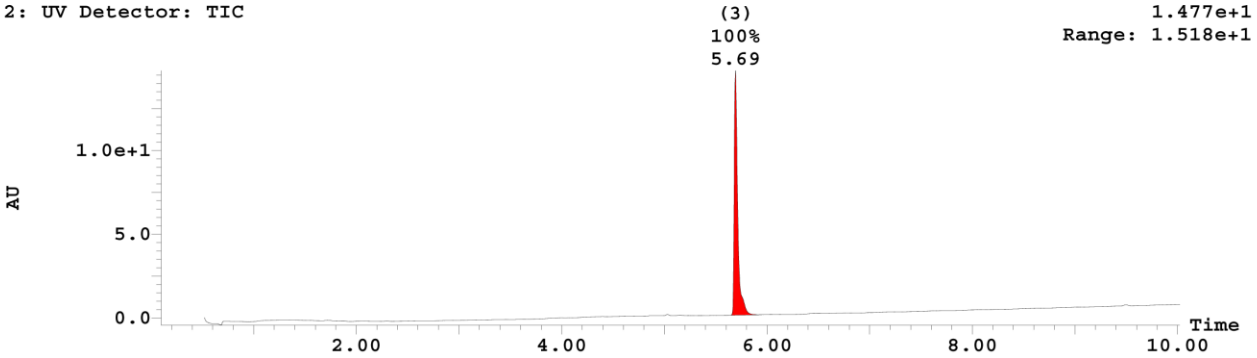

Peak Number	Compound	Time	AreaAbs	Area %Total	Width	Height	Mass Found
3		5.69	6e+005	100.00	0	1e+007	

Peak ID	Time	Mass Found
3	5.70	

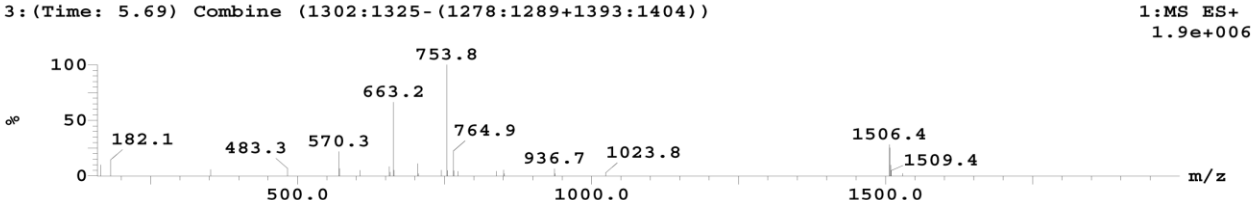

FITC labelled GR_pS83:

FITC labelled GR_pS83 peptide was prepared following General Protocol for SPPS. The crude peptide was purified by reverse-phase HPLC (gradient: 5% B for 1 min, 5-32%B in 3min, 32-37% in 15min, Waters Xselect CSH column) to afford the desired peptide as a white fluffy solid. Yield (12 mg) in > 95% purity according to analytical UPLC. Rt 7.13 min (3-60% B in 10 min).

$[M + 2H]^{2+}$ calculated for $C_{88}H_{125}N_{16}O_{29}PS_2$, 983.6; found 984.0.

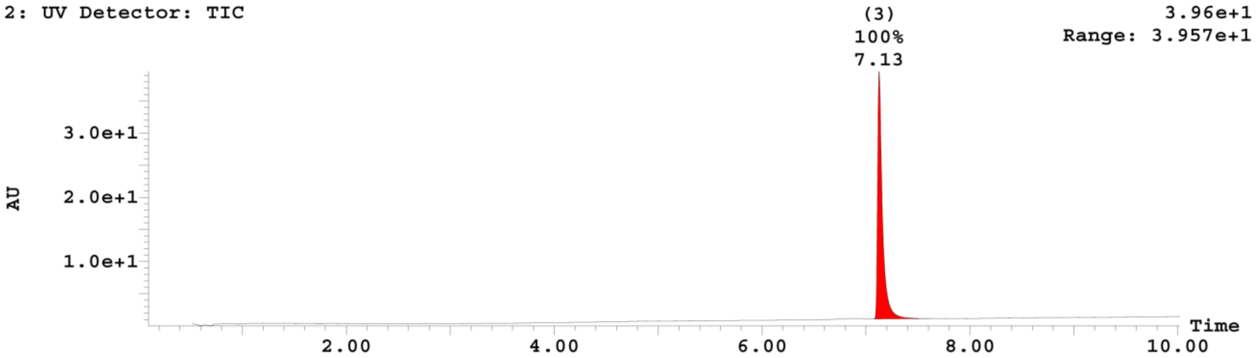

Peak Number	Compound	Time	AreaAbs	Area %Total	Width	Height	Mass Found
3		7.13	2e+006	100.00	1	4e+007	

MS Spectrum (+) for selected peak

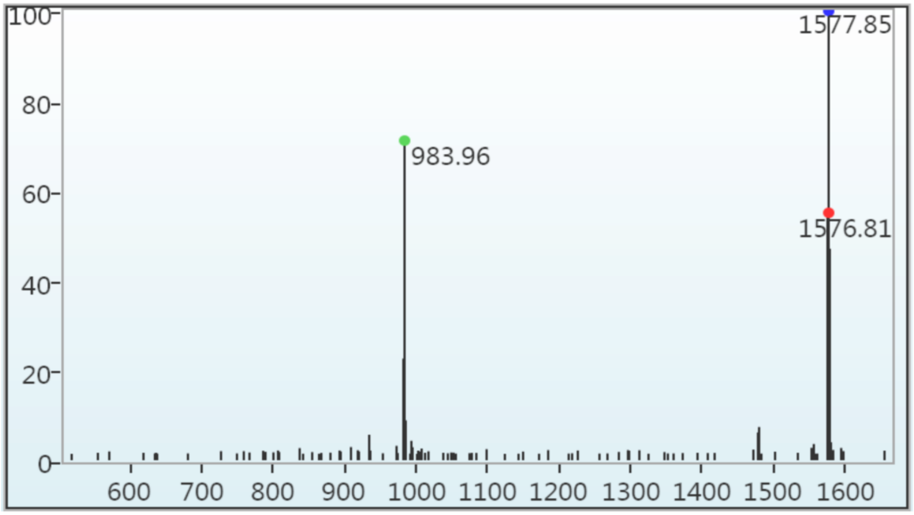

FAM labelled GR_pS113:

Project: BC100475.1 MW:1773.70
[Fluo(6FAM)]QGQISL[pS]SGETDL[COOH]

ID:GR_S113
P7635-1 2 mg

Thermo SCIENTIFIC
800-874-3723 or 815-968-0747 • FOR RESEARCH USE ONLY
3747 N. Meridian Rd., Rockford, IL 61101 U.S.A.
www.thermoscientific.com/pierce

HPLC Report

Sample ID:	P7635-1
Date File:	D:\32Karat\Projects\Default\Data\P7635-1
Method:	D:\32Karat\Projects\Default\Methods\PEP-CD071-C18.met
Column:	Onyx Monlithic C18, 100x3.0 mm 3u
Solvent:	A: 0.05% TFA in H_2O; B: 0.05% TFA in ACN
Wavelenght:	220 nm; Detector lamp: UV
Run Time:	3/27/2018 2:32:25 PM
HPLC ID#:	CD-071/EQ-024

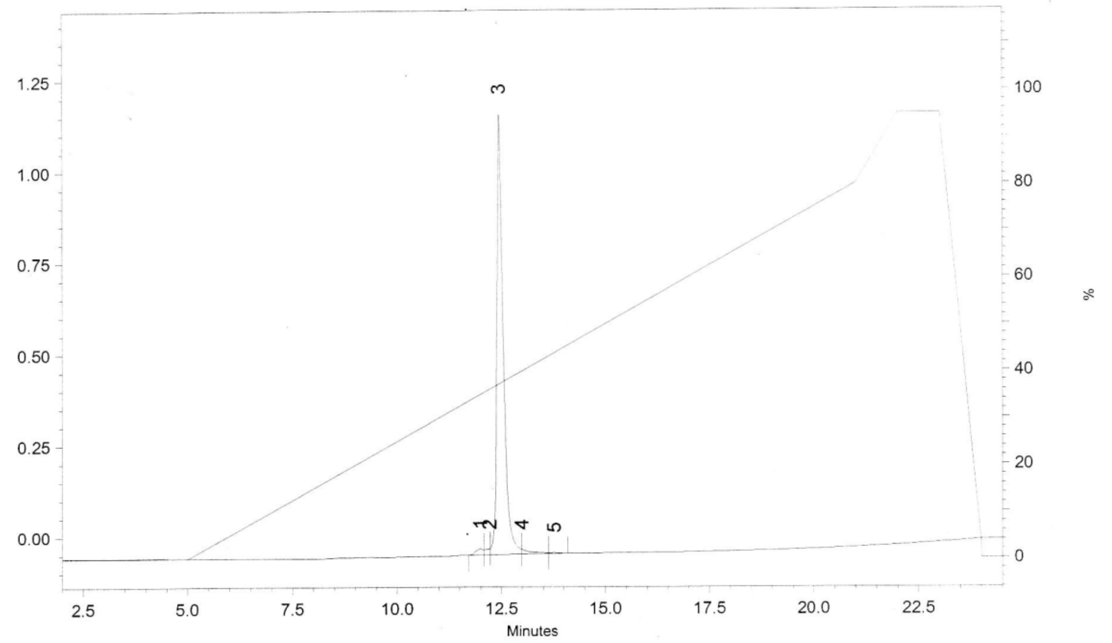

Pk #	Retention Time	Height	Height Percent	Area	Area Percent
1	12.000	16815	1.342	200810	1.502
2	12.217	15573	1.243	131452	0.983
3	12.450	1205322	96.178	12807464	95.800
4	12.983	13258	1.058	198936	1.488
5	13.767	2247	0.179	30291	0.227

Totals					
		1253215	100.000	13368953	100.000

Applied Biosystems Voyager System 1099

Voyager Spec #1=>SM5[BP = 1773.7, 1237]

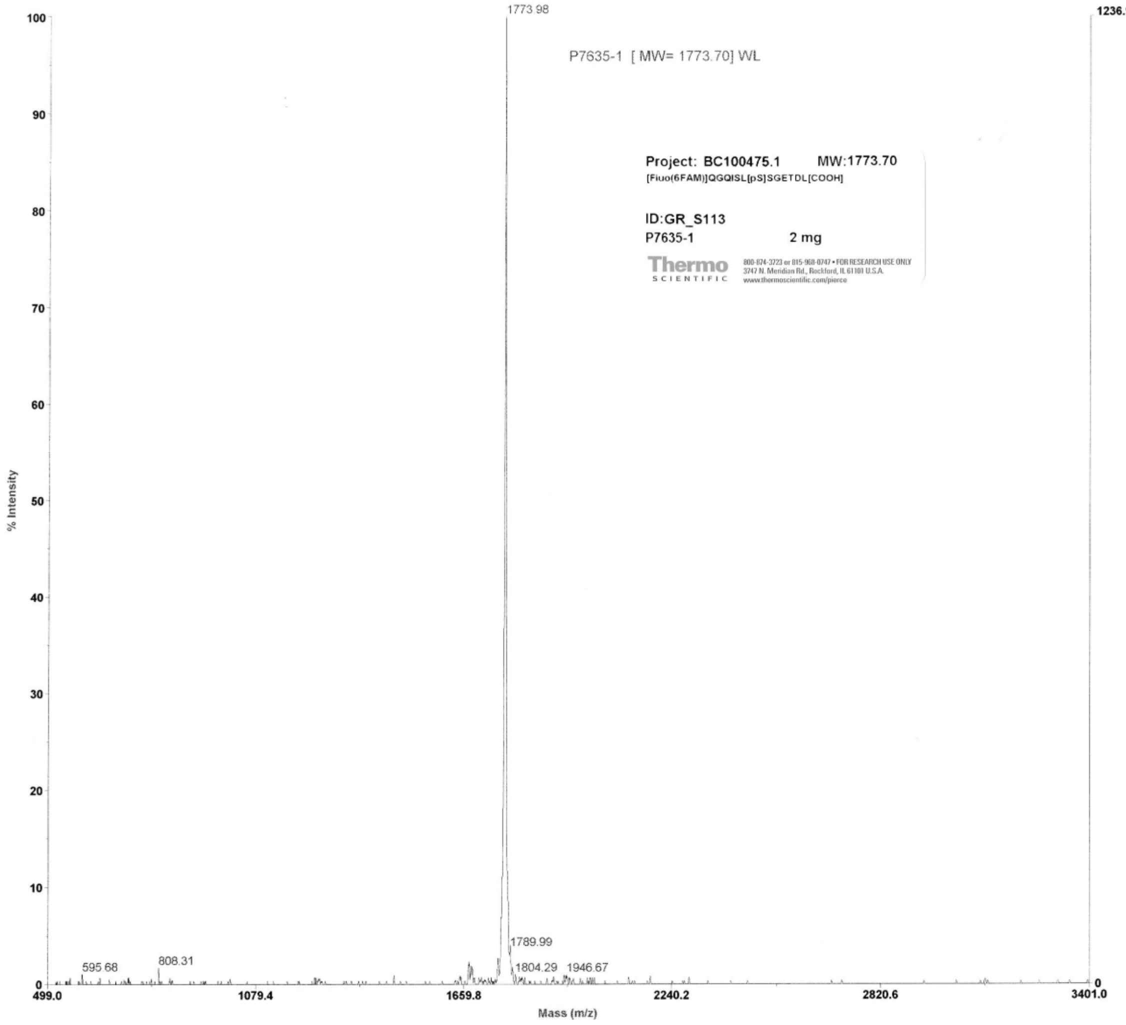

Mode of operation:	Linear
Extraction mode:	Delayed
Polarity:	Negative
Acquisition control:	Manual
Accelerating voltage:	25000 V
Grid voltage:	91%
Guide wire 0:	0.05%
Extraction delay time:	100 nsec
Acquisition mass range:	500 -- 3400 Da
Number of laser shots:	100/spectrum
Laser intensity:	1901
Laser Rep Rate:	3.0 Hz
Calibration type:	Default
Calibration matrix:	a-Cyano-4-hydroxycinnamic acic
Low mass gate:	Off
Digitizer start time:	13.008
Bin size:	4 nsec
Number of data points:	5188
Vertical scale:	200 mV
Vertical offset:	0%
Input bandwidth:	150 MHz
Sample well:	07
Plate ID:	100 WELL PLATE
Serial number:	1099
Instrument name:	Voyager-DE
Plate type filename:	C:\VOYAGER\100 well plate.plt
Lab name:	BioSynthesis, Inc
Absolute x-position:	32943.1
Absolute y-position:	47447.3
Relative x-position:	875.551
Relative y-position:	139.827
Shots in spectrum:	12
Source pressure:	7.08e-007
Mirror pressure:	0
TC2 pressure:	0.001
TIS gate width:	30
TIS flight length:	940

Acquired: 11:52:00, March 27, 2018

Printed: 11:54, March 27, 2018

C:\VOYAGER\Data\Biosyn2018\P7635-1_0003.dat

Acetylated GR_pS134:

Acetylated GR_pS134 peptide was prepared following General Protocol for SPPS. The crude peptide was purified by reverse-phase HPLC (gradient: 5% B for 1 min, 5-25% B in 25 min, Kromasil C18 column) to afford the desired peptide as a white fluffy solid. Yield (15 mg) in > 95% purity according to analytical UPLC. Rt 2.40 min (3-60% B in 10 min).

$[M + 2H]^{2+}$ calculated for $C_{62}H_{105}N_{20}O_{26}P$, 789.3; found 789.9.

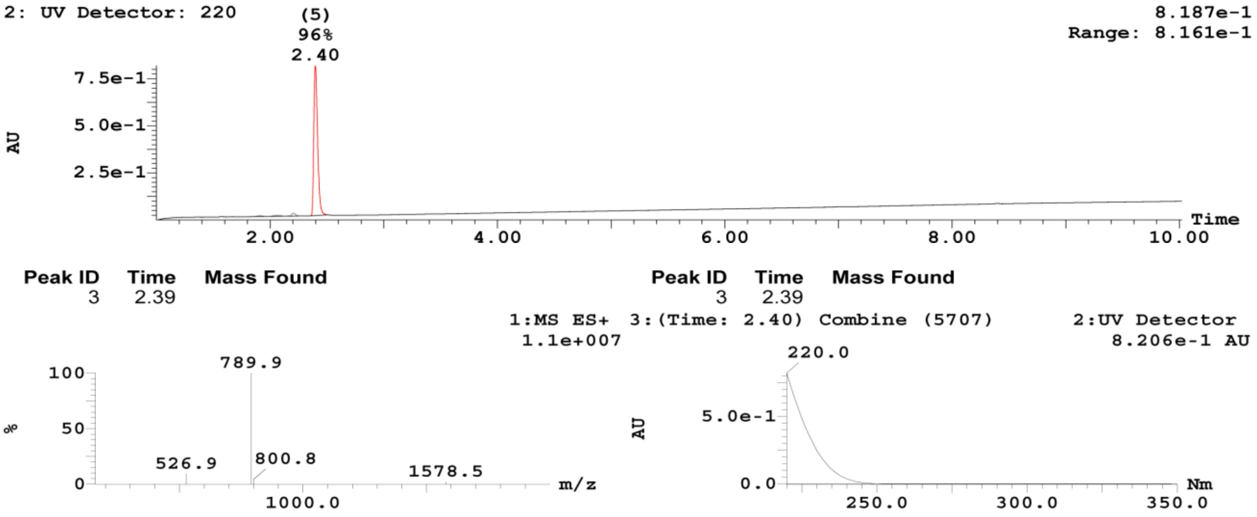

FITC labelled GR_pS134:

FITC labelled GR_pS134 peptide was prepared following General Protocol for SPPS. The crude peptide was purified by reverse-phase HPLC (gradient: 5% B for 1 min, 20-35% B in 30 min, Kromasil C18 column) to afford the desired peptide as a white fluffy solid. Yield (42 mg) in > 95% purity according to analytical UPLC. Rt 4.50 min (3-60% B in 10 min).

$[M + 2H]^{2+}$ calculated for $C_{87}H_{125}N_{22}O_{31}PS$, 1019.6; found 1020.2.

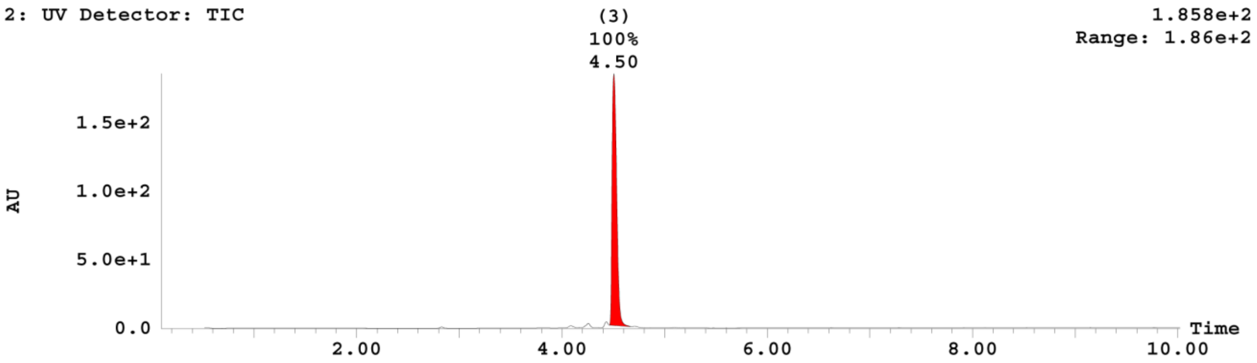

Peak Number	Compound	Time	AreaAbs	Area %Total	Width	Height	Mass Found
3		4.50	1e+007	100.00	0	2e+008	

Peak ID	Time	Mass Found
3	4.50	

Peak ID	Time	Mass Found
3	4.50	

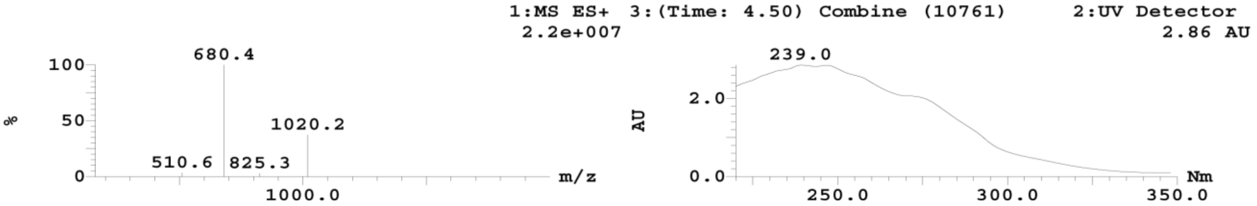

FITC labelled GR_pS203:

FITC labelled GR_pS203 peptide was prepared following General Protocol for SPPS. The crude peptide was purified by reverse-phase HPLC (gradient: 5% B for 1 min, 5-26% B in 3 min, 26-31% B in 15 min, Waters CSH C18 column) to afford the desired peptide as a white fluffy solid. Yield (26 mg) in > 95% purity according to analytical UPLC. Rt 6.10 min (3-60% B in 10 min).

$[M + 2H]^{2+}$ calculated for $C_{84}H_{112}N_{17}O_{32}PS$, 968.0; found 968.2.

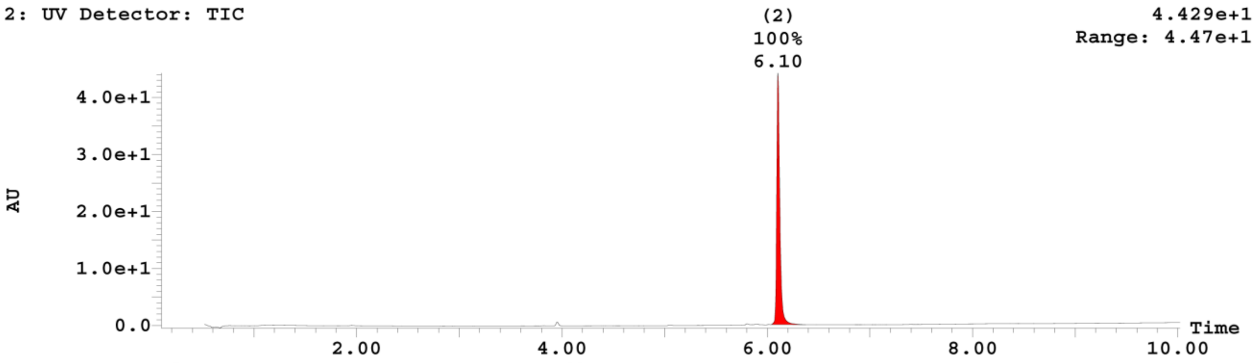

Peak Number	Compound	Time	AreaAbs	Area %Total	Width	Height	Mass Found
2		6.10	2e+006	100.00	0	4e+007	

Peak ID	Time	Mass Found
2	6.10	

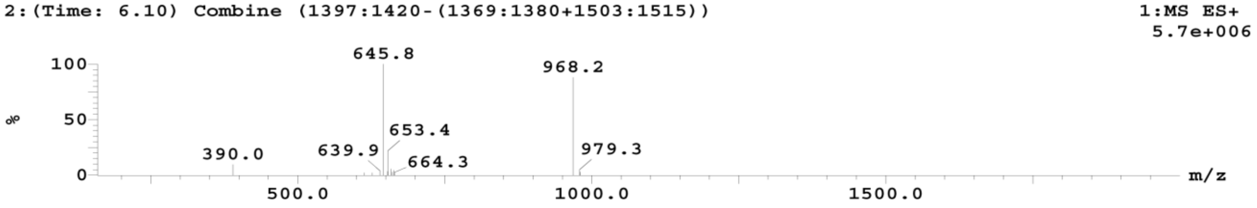

FITC labelled GR_pS211:

FITC labelled GR_pS211 peptide was prepared following General Protocol for SPPS. The crude peptide was purified by reverse-phase HPLC (gradient: 5% B for 1 min, 5-23% B in 3 min, 23-28% B in 15 min, Waters CSH C18 column) to afford the desired peptide as a white fluffy solid. Yield (13 mg) in > 95% purity according to analytical UPLC. Rt 5.13 min (3-60% B in 10 min).

[M + 2H]$^{2+}$ calculated for $C_{84}H_{112}N_{17}O_{32}PS$, 1051.1; found 1051.1.

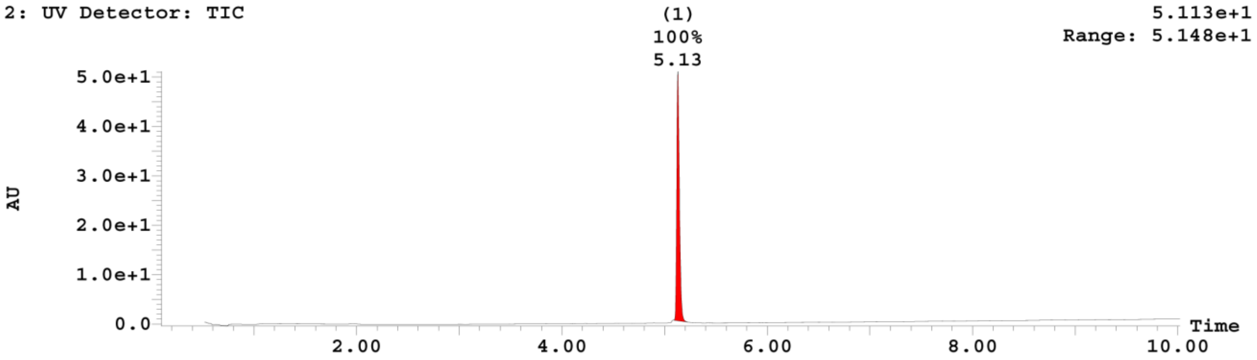

Peak Number	Compound	Time	AreaAbs	Area %Total	Width	Height	Mass Found
1		5.13	2e+006	100.00	0	5e+007	

Peak ID	Time	Mass Found
1	5.13	

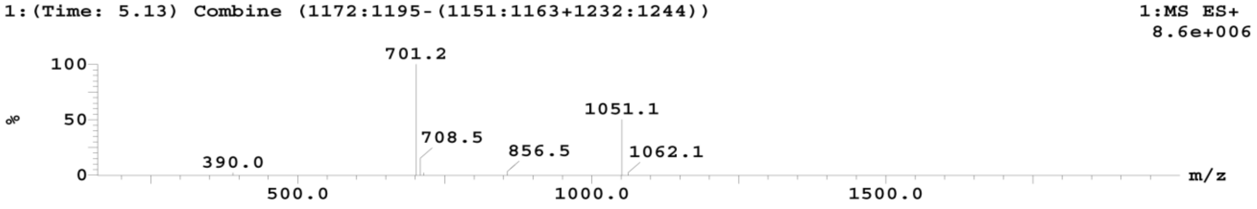

FITC labelled GR_pS226:

FITC labelled GR_pS226 peptide was prepared following General Protocol for SPPS. The crude peptide was purified by reverse-phase HPLC (gradient: 5% B for 1 min, 5-29% B in 3 min, 29-34% B in 15 min, Waters CSH C18 column) to afford the desired peptide as a white fluffy solid. Yield (17 mg) in > 95% purity according to analytical UPLC. Rt 6.95 min (3-60% B in 10 min).

[M + 2H]$^{2+}$ calculated for $C_{83}H_{113}N_{16}O_{33}PS_2$, 979.5; found 979.6.

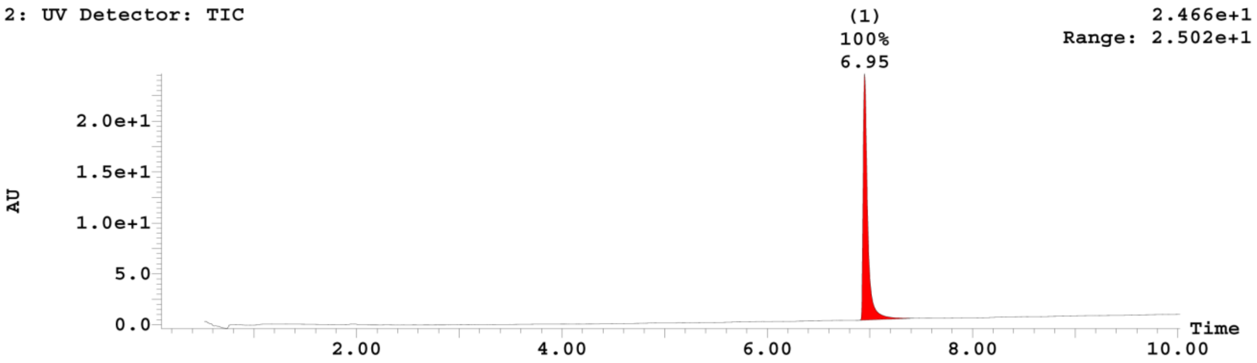

Peak Number	Compound	Time	AreaAbs	Area %Total	Width	Height	Mass Found
1		6.95	1e+006	100.00	1	2e+007	

Peak ID	Time	Mass Found
1	6.95	

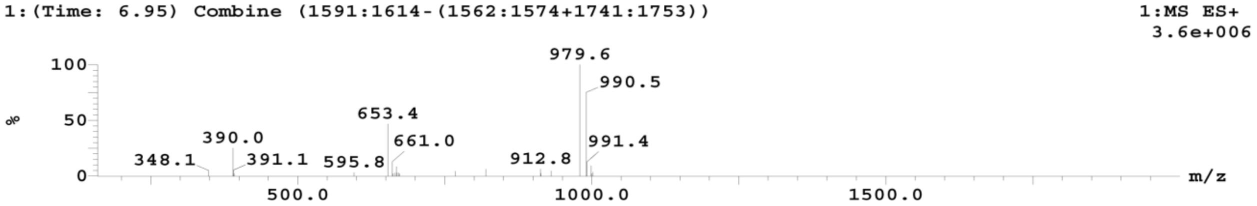

FITC labelled GR_pS234:

FITC labelled GR_pS234 peptide was prepared following General Protocol for SPPS. The crude peptide was purified by reverse-phase HPLC (gradient: 5% B for 1 min, 5-30% B in 3 min, 30-35% B in 15 min, Waters CSH C18 column) to afford the desired peptide as a white fluffy solid. Yield (27 mg) in > 95% purity according to analytical UPLC. Rt 7.28 min (3-60% B in 10 min).

$[M + 2H]^{2+}$ calculated for $C_{86}H_{113}N_{16}O_{33}PS$, 981.5; found 981.6.

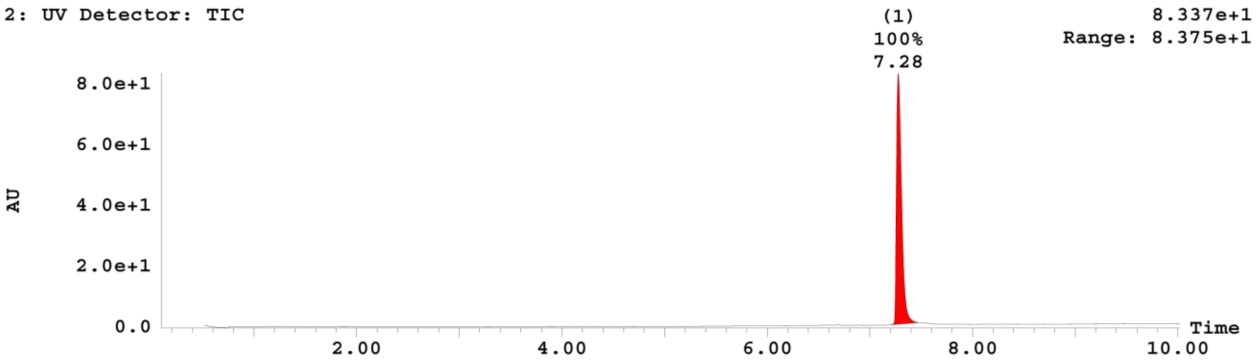

Peak Number	Compound	Time	AreaAbs	Area %Total	Width	Height	Mass Found
1		7.28	5e+006	100.00	0	8e+007	

Peak ID	Time	Mass Found
1	7.29	

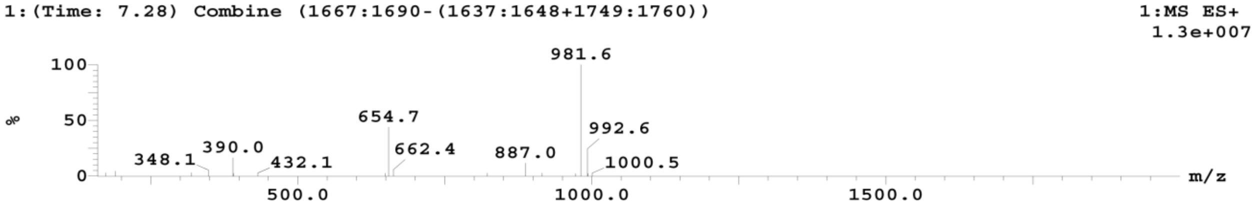

FITC labelled GR_pS267:

FITC labelled GR_pS267 peptide was prepared following General Protocol for SPPS. The crude peptide was purified by reverse-phase HPLC (gradient: 5% B for 1 min, 5-29% B in 3 min, 29-34% B in 15 min, Waters CSH C18 column) to afford the desired peptide as a white fluffy solid. Yield (8 mg) in > 95% purity according to analytical UPLC. Rt 7.07 min (3-60% B in 10 min).

$[M + 2H]^{2+}$ calculated for $C_{83}H_{119}N_{16}O_{30}PS$, 942.5; found 942.7.

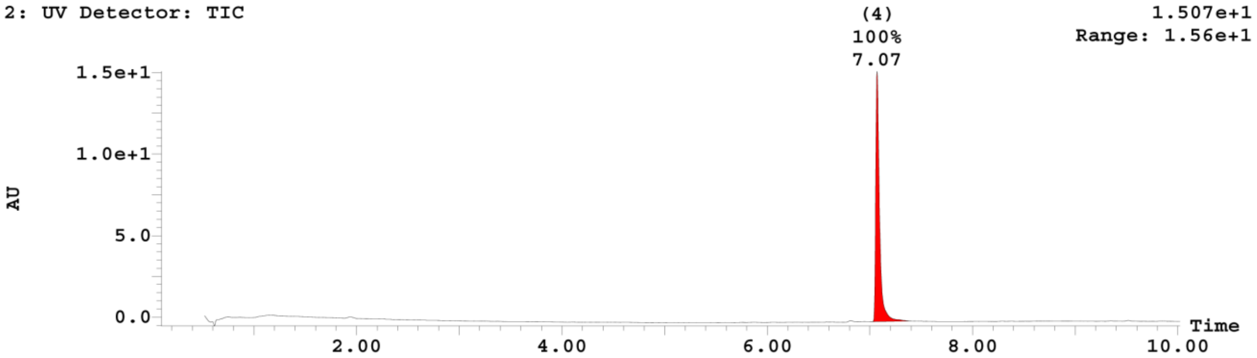

Peak Number	Compound	Time	AreaAbs	Area %Total	Width	Height	Mass Found
4		7.07	7e+005	100.00	0	2e+007	

Peak ID	Time	Mass Found
4	7.07	

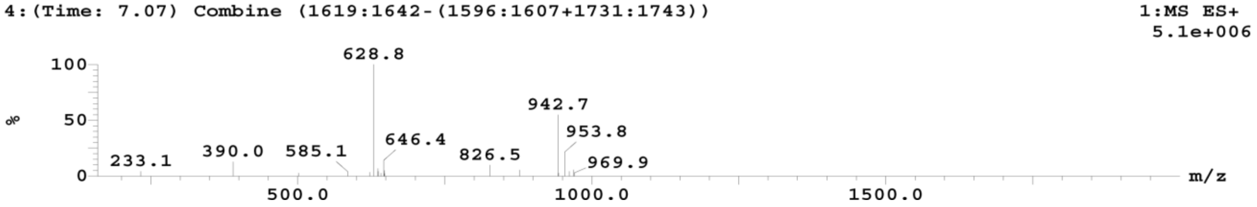

FITC labelled GR_pS404:

FITC labelled GR_pS404 peptide was prepared following General Protocol for SPPS. The crude peptide was purified by reverse-phase HPLC (gradient: 5% B for 1 min, 5-22% B in 3 min, 22-27% B in 15 min, Waters CSH C18 column) to afford the desired peptide as a white fluffy solid. Yield (16 mg) in > 95% purity according to analytical UPLC. Rt 5.33 min (3-60% B in 10 min).

$[M + 2H]^{2+}$ calculated for $C_{80}H_{111}N_{18}O_{31}PS_2$, 958.5; found 958.6.

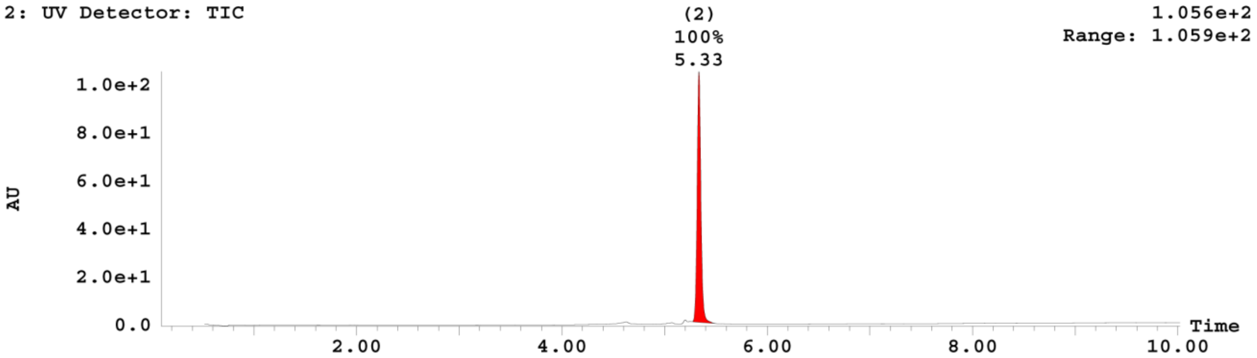

Peak Number	Compound	Time	AreaAbs	Area %Total	Width	Height	Mass Found
2		5.33	5e+006	100.00	0	1e+008	

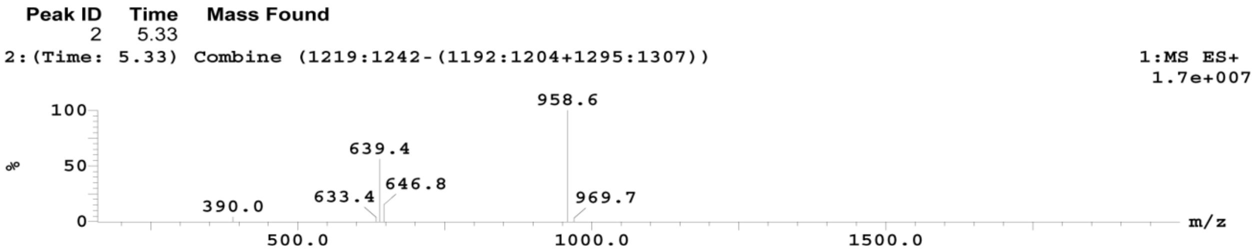

Acetylated GR_pT493:

Acetylated GR_pT493 peptide was prepared following General Protocol for SPPS. The crude peptide was purified by reverse-phase HPLC (gradient: 0% B for 4min, 0-15% B in 15min, Waters Atlantis T3 column) to afford the desired peptide as a white fluffy solid. Yield (61 mg) in > 95% purity according to analytical UPLC. Rt 1.41 min (3-60% B in 10 min).

$[M + 2H]^{2+}$ calculated for $C_{68}H_{127}N_{22}O_{22}P$, 818.4; found 818.6.

Sample ID: EN08354-03-001 SN1060315207 **Weight:** <has no weight> **Purity:** 98,02 %

UV Chromatogram

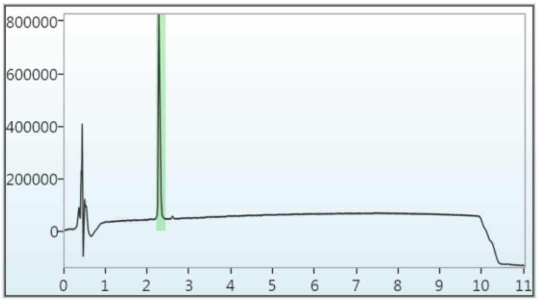

MS Spectrum (+) for selected peak

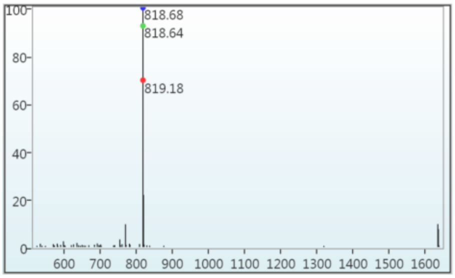

MS Spectrum (-) for selected peak

N/A

FITC labelled GR_pT493:

FITC labelled GR_pT493 peptide was prepared following General Protocol for SPPS. The crude peptide was purified by reverse-phase HPLC (gradient: 5% B for 1 min, 5-18% B in 3 min, 18-23% B in 15 min, Waters Atlantis T3 column) to afford the desired peptide as a white fluffy solid. Yield (25 mg) in > 95% purity according to analytical UPLC. Rt 3.33 min (3-60% B in 10 min).

$[M + 2H]^{2+}$ calculated for $C_{93}H_{147}N_{24}O_{27}PS$, 1048.7; found 1049.3.

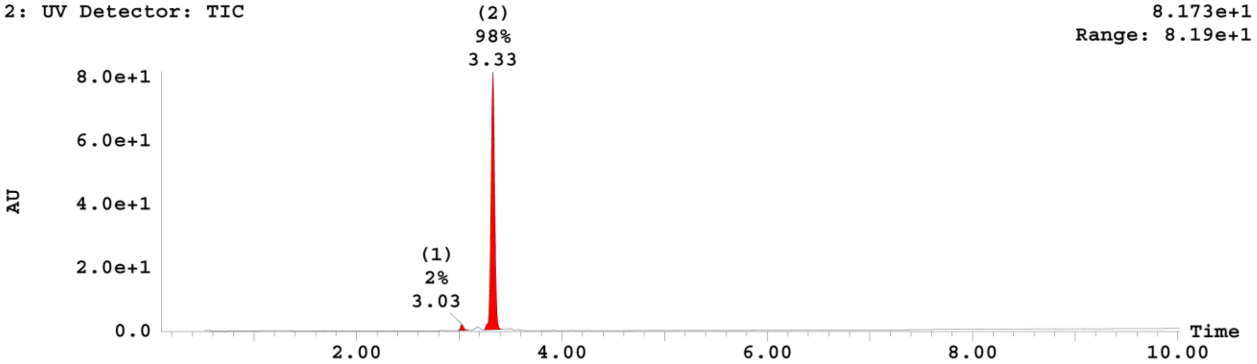

Peak Number	Compound	Time	AreaAbs	Area %Total	Width	Height	Mass Found
1		3.03	7e+004	2.03	0	2e+006	
2		3.33	4e+006	97.97	0	8e+007	

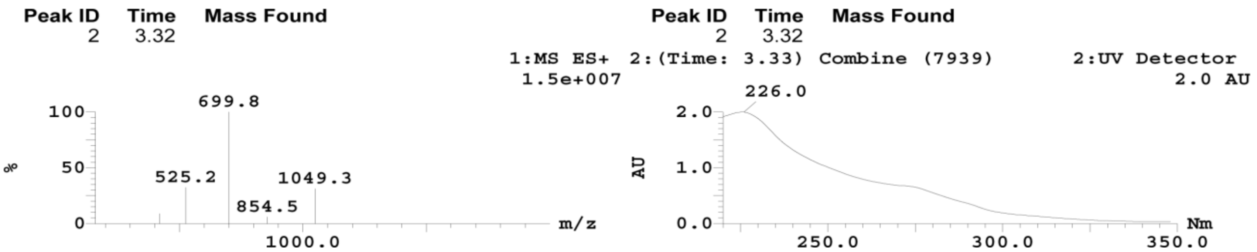

Acetylated GR_pT524:

Acetylated GR_pT524 peptide was prepared following General Protocol for SPPS. The crude peptide was purified by reverse-phase HPLC (gradient: 5% B for 1 min, 22-40% B in 25 min, Kromasil C18 column) to afford the desired peptide as a white fluffy solid. Yield (34 mg) in > 95% purity according to analytical UPLC. Rt 4.96 min (3-60% B in 10 min).

$[M + H]^+$ calculated for $C_{66}H_{114}N_{15}O_{22}P$, 1501.7; found 1501.7.

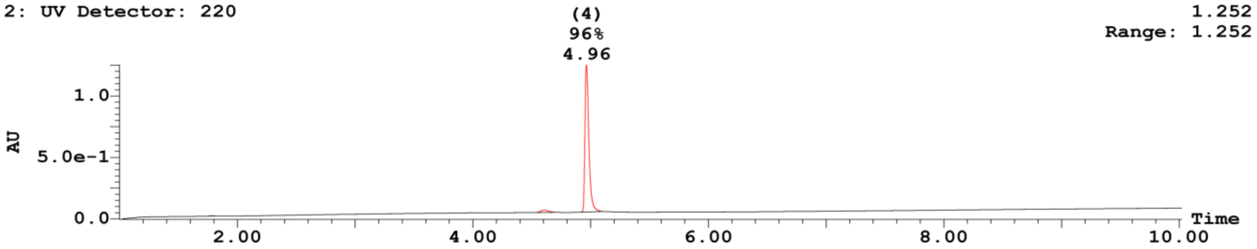

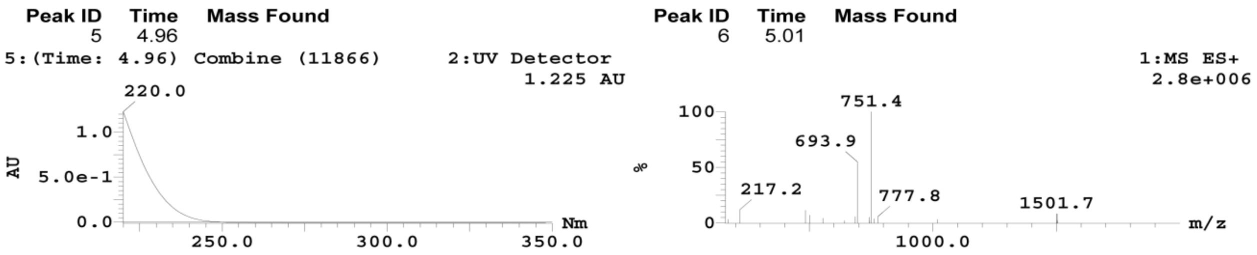

FITC labelled GR_pT524:

FITC labelled GR_pT524 peptide was prepared following General Protocol for SPPS. The crude peptide was purified by reverse-phase HPLC (gradient: 5% B for 1 min, 31-45% B in 30 min, Kromasil C18 column) to afford the desired peptide as a white fluffy solid. Yield (19 mg) in > 95% purity according to analytical UPLC. Rt 6.31 min (3-60% B in 10 min).

$[M + H]^+$ calculated for $C_{91}H_{134}N_{17}O_{27}PS$, 1962.2; found 1962.8.

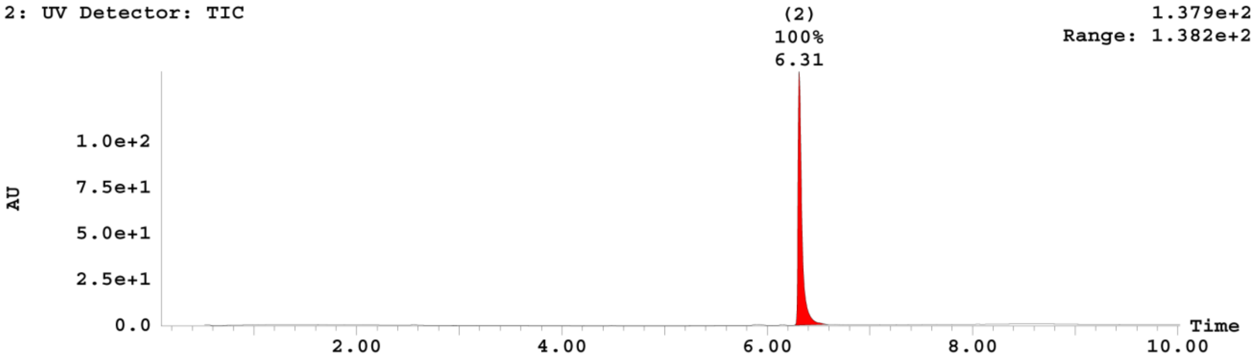

Peak Number	Compound	Time	AreaAbs	Area %Total	Width	Height	Mass Found
2		6.31	6e+006	100.00	0	1e+008	

Peak ID	Time	Mass Found
2	6.31	

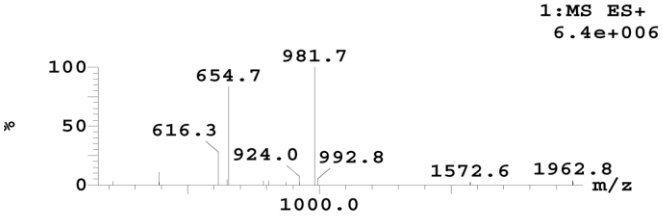

Acetylated GR_pT561:

Acetylated GR_pT561 peptide was prepared following General Protocol for SPPS. The crude peptide was purified by reverse-phase HPLC (gradient: 5% B for 1 min, 10-50% B in 25 min, Kromasil C18 column) to afford the desired peptide as a white fluffy solid. Yield (18 mg) in 94% purity according to analytical UPLC. Rt 6.39 min (3-60% B in 10 min).

$[M + H]^+$ calculated for $C_{66}H_{114}N_{15}O_{22}P$, 1646.8; found 1646.7.

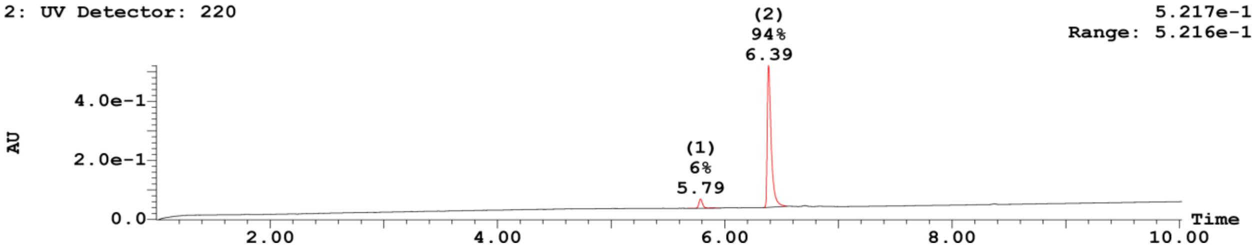

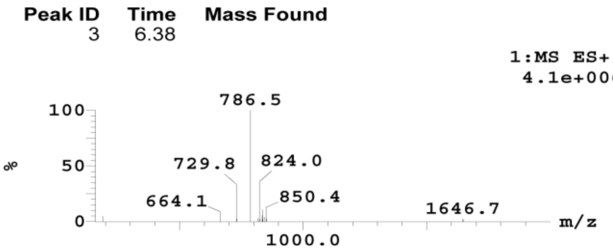

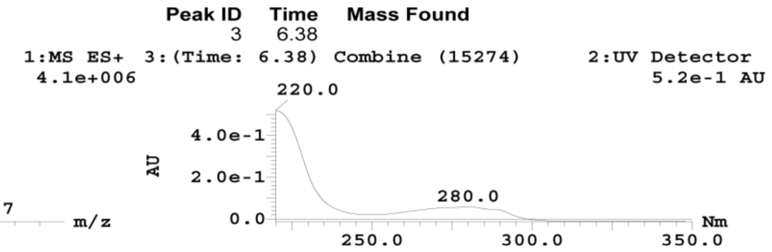

FITC labelled GR_pT561:

FITC labelled GR_pT561 peptide was prepared following General Protocol for SPPS. The crude peptide was purified by reverse-phase HPLC (gradient: 5% B for 1 min, 5-33% B in 3 min, 33-38% B in 15 min, Waters Xselect CSH C18 column) to afford the desired peptide as a white fluffy solid. Yield (4 mg) in > 95% purity according to analytical UPLC. Rt 7.55 min (3-60% B in 10 min).

$[M + 2H]^{2+}$ calculated for $C_{93}H_{133}N_{20}O_{28}PS_3$, 1053.7; found 1053.7.

Sample ID: EN08354-04-001 <has no SN number> **Weight:** <has no weight> **Purity:** 95,01 %

UV Chromatogram

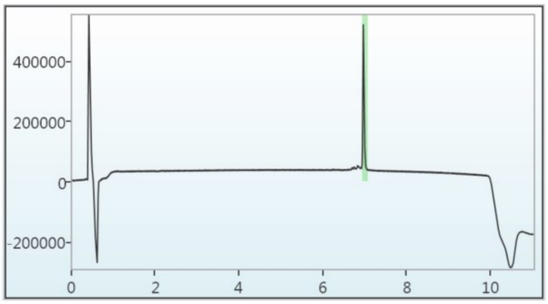

MS Spectrum (+) for selected peak

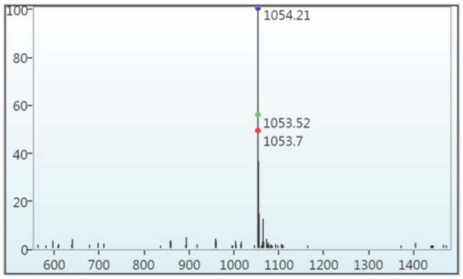

MS Spectrum (-) for selected peak

N/A

FITC labelled GR_pT562:

FITC labelled GR_pT562 peptide was prepared following General Protocol for SPPS. The crude peptide was purified by reverse-phase HPLC (gradient: 5% B for 1 min, 5-33% B in 3 min, 33-38% B in 15 min, Waters Xselect CSH C18 column) to afford the desired peptide as a white fluffy solid. Yield (5 mg) in 83% purity according to analytical UPLC. Rt 4.14 min (3-93% B in 9 min).

$[M + 2H]^{2+}$ calculated for $C_{92}H_{130}N_{19}O_{28}PS_3$, 1039.2; found 1039.4.

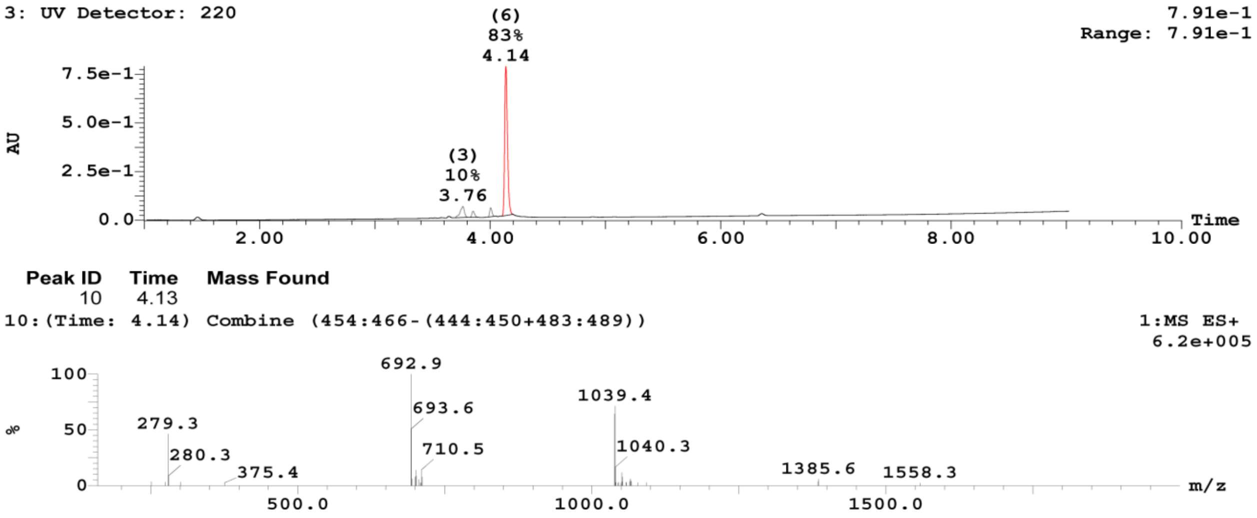

Acetylated GR_pS617:

Acetylated GR_pS617 peptide was prepared following General Protocol for SPPS. The crude peptide was purified by reverse-phase HPLC (gradient: 5% B for 1 min, 20-28% B in 35 min, Kromasil C18 column) to afford the desired peptide as a white fluffy solid. Yield (10 mg) in > 95% purity according to analytical UPLC. Rt 4.46 min (3-60% B in 10 min).

$[M + H]^+$ calculated for $C_{68}H_{108}N_{21}O_{24}PS$, 1667.8; found 1667.3.

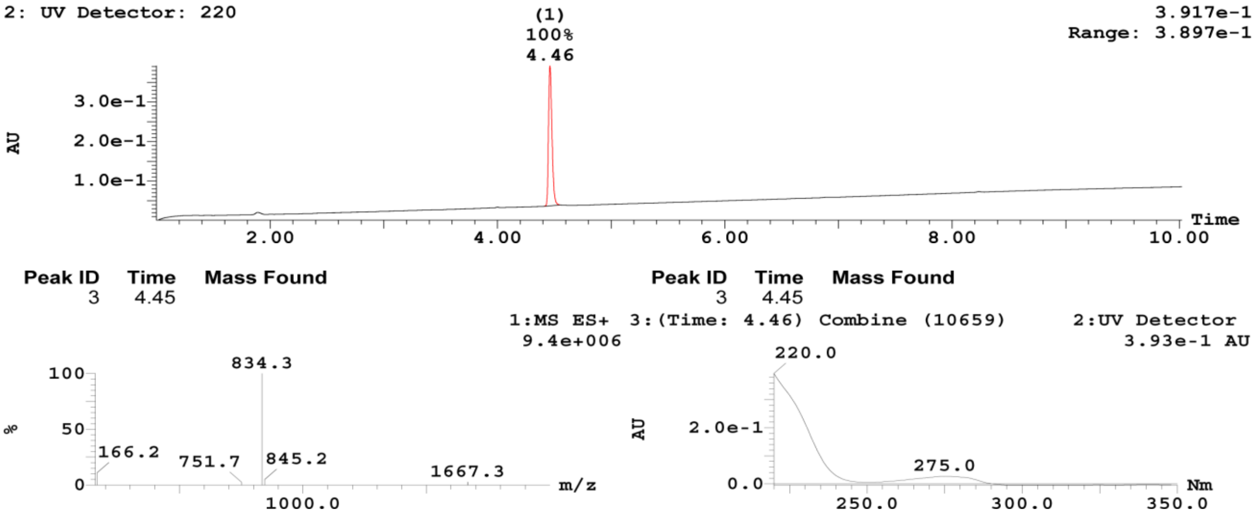

FITC labelled GR_pS617:

FITC labelled GR_pS617 peptide was prepared following General Protocol for SPPS. The crude peptide was purified by reverse-phase HPLC (gradient: 5% B for 1 min, 26-35 % B in 25 min, Kromasil C18 column) to afford the desired peptide as a white fluffy solid. Yield (32 mg) in > 95% purity according to analytical UPLC. Rt 5.82 min (3-60% B in 10 min).

$[M + 2H]^{2+}$ calculated for $C_{93}H_{128}N_{23}O_{29}PS_2$, 1064.1; found 1064.7.

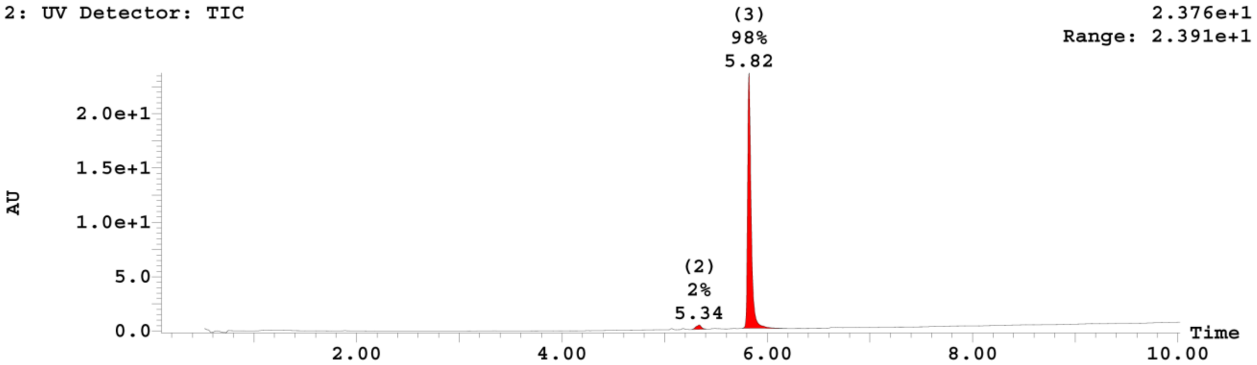

Peak Number	Compound	Time	AreaAbs	Area %Total	Width	Height	Mass Found
2		5.34	3e+004	2.49	0	4e+005	
3		5.82	1e+006	97.51	1	2e+007	

Peak ID	Time	Mass Found
3	5.81	

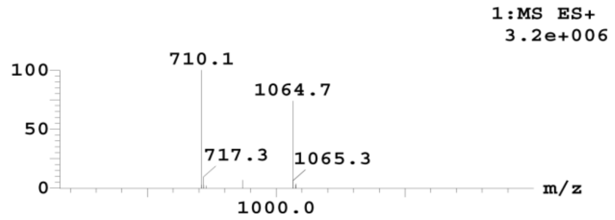

Acetylated GR_pT635:

Acetylated GR_pT635 peptide was prepared following General Protocol for SPPS. The crude peptide was purified by reverse-phase HPLC (gradient: 5% B for 1 min, 5-15% B in 3 min, 15-20% B in 15 min, Waters Xselect CSH C18 column) to afford the desired peptide as a white fluffy solid. Yield (17 mg) in > 95% purity according to analytical UPLC. Rt 4.89 min (3-60% B in 10 min).

$[M + H]^+$ calculated for $C_{69}H_{111}N_{18}O_{26}PS_3$, 1736.9; found 1735.6.

Sample ID: EN08354-10-001 <has no SN number> **Weight:** <has no weight> **Purity:** 99,92 %

UV Chromatogram

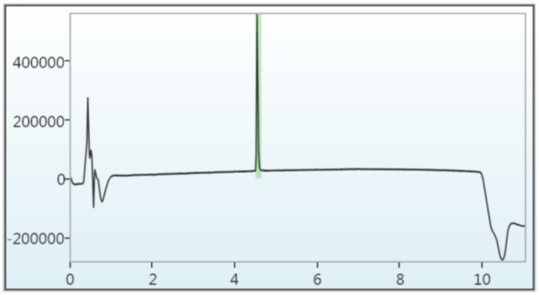

MS Spectrum (+) for selected peak

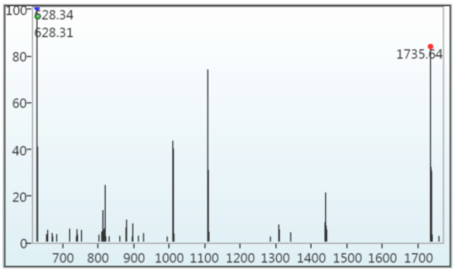

MS Spectrum (-) for selected peak

N/A

FITC labelled GR_pT635:

FITC labelled GR_pT635 peptide was prepared following General Protocol for SPPS. The crude peptide was purified by reverse-phase HPLC (gradient: 5% B for 1 min, 31-33 % B in 15 min, Kromasil C18 column) to afford the desired peptide as a white fluffy solid. Yield (10 mg) in 95% purity according to analytical UPLC. Rt 6.48 min (3-60% B in 10 min).

$[M + 2H]^{2+}$ calculated for $C_{94}H_{131}N_{20}O_{31}PS_4$, 1098.7; found 1099.2.

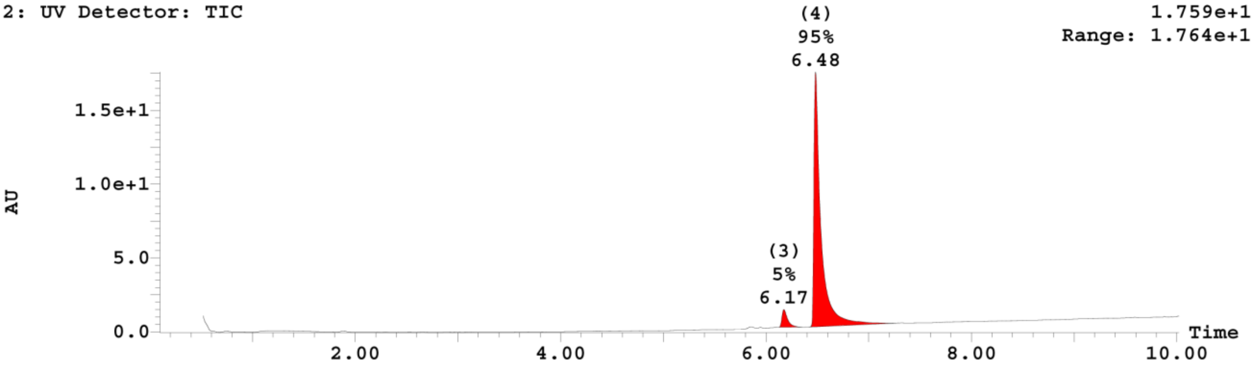

Peak Number	Compound	Time	AreaAbs	Area %Total	Width	Height	Mass Found
3		6.17	8e+004	5.22	0	1e+006	
4		6.48	1e+006	94.78	1	2e+007	

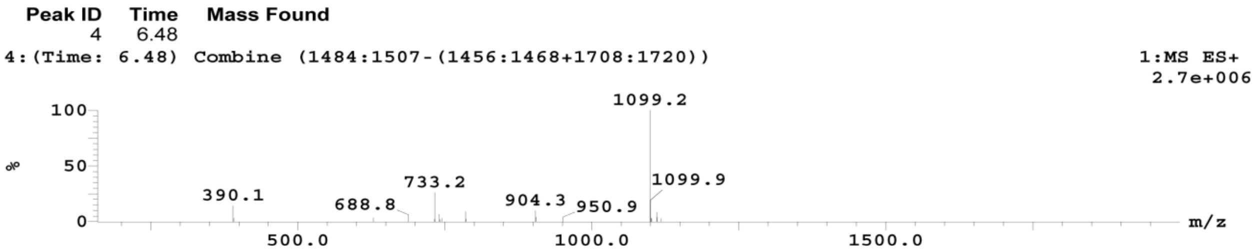

Acetylated GR_pT524-pS617:

Acetylated GR_pT524-pS617 peptide was prepared following General Protocol for SPPS. The crude peptide was purified by reverse-phase HPLC (gradient: 5% B for 1 min, 5-23% B in 3min, 23-28% B in 15min, Waters Xselect CSH C18 column) to afford the desired peptide as a white fluffy solid. Yield (38 mg) in > 95% purity according to analytical UPLC. Rt 5.40 min (3-60% B in 10 min).

[M + 3H]$^{3+}$ calculated for $C_{142}H_{233}N_{41}O_{49}P_2S$, 1131.6; found 1131.4.

Sample ID: EN08354-46-001 <has no SN number> **Weight:** <has no weight> **Purity:** 98,09 %

UV Chromatogram

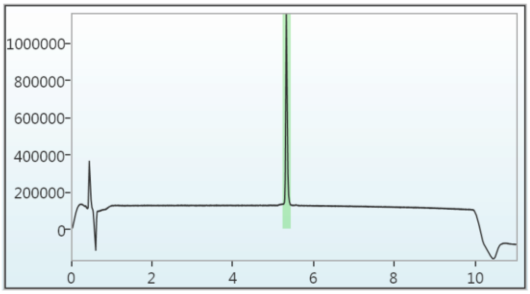

MS Spectrum (+) for selected peak

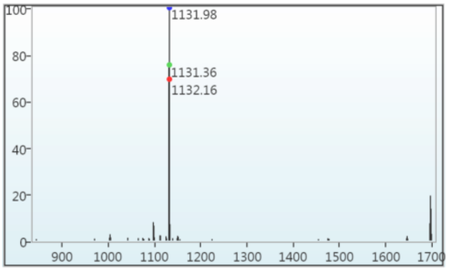

MS Spectrum (-) for selected peak

N/A

FITC labelled GR_pT524-pS617:

FITC labelled GR_pT524-pS617 peptide was prepared following General Protocol for SPPS. The crude peptide was purified by reverse-phase HPLC (gradient: 5% B for 1 min, 5-29% B in 3min, 29-34% B in 15min, Waters Atlantis T3 column) to afford the desired peptide as a white fluffy solid. Yield (30 mg) in > 95% purity according to analytical UPLC. Rt 4.18 min (5-95% B in 10 min).

[M + 3H]$^{3+}$ calculated for $C_{167}H_{253}N_{43}O_{54}P_2S_2$, 1285.1; found 1285.3.

Sample ID: EN08354-46-002 <has no SN number> **Weight:** <has no weight> **Purity:** 99,07 %

UV Chromatogram

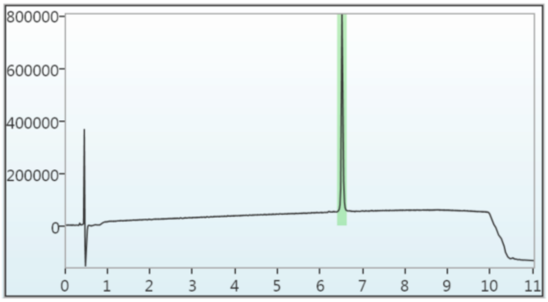

MS Spectrum (+) for selected peak

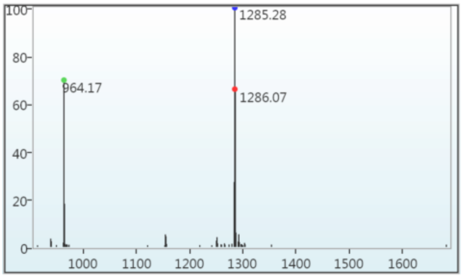

MS Spectrum (-) for selected peak

N/A

FITC labelled GR_pS83-pT524:

FITC labelled GR_pS83-pT524 peptide was prepared following General Protocol for SPPS. The crude peptide was purified by reverse-phase HPLC (gradient: 5% B for 1 min, 5-27% B in 3min, 27-32% B in 15min, Waters Xselect CSH C18 column) to afford the desired peptide as a white fluffy solid. Yield (7 mg) in > 95% purity according to analytical UPLC. Rt 7.28 min (10-50% B in 10 min).

$[M + 3H]^{3+}$ calculated for $C_{162}H_{250}N_{36}O_{54}P_2S_2$, 1231.3; found 1230.8.

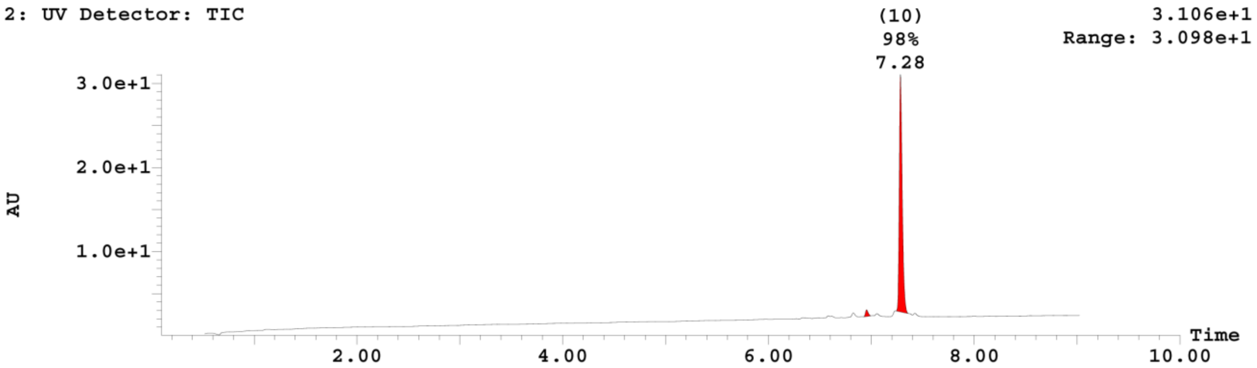

Peak Number	Compound	Time	AreaAbs	Area %Total	Width	Height	Mass Found
8		6.95	2e+004	2.42	0	8e+005	
10		7.28	1e+006	97.58	0	3e+007	

Peak ID	Time	Mass Found
10	7.28	

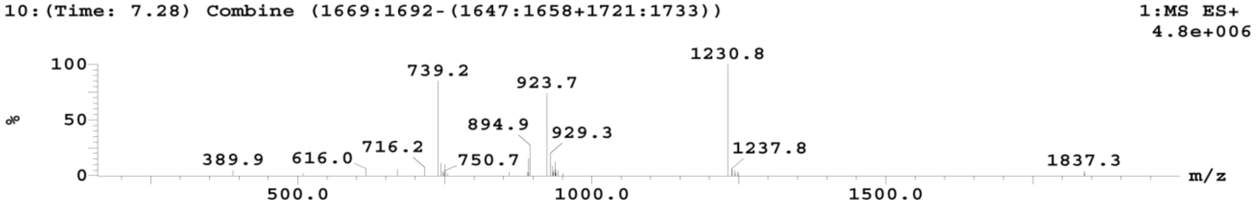

FAM labelled GR_pS134-pT524:

Sample Information

Acquired by : Admin
Sample Name : P7192-1
Sample ID : P7192-1
Data Filename : P7192-1a.lcd
Method Filename : ANAPEP_2.lcm
Date Acquired : 1/17/2018 1:18:01 AM
Data Processed : 1/17/2018 1:53:27 AM

Project: BC100046.1 MW:3620.79
[Fluo(6FAM)]NLNRST[pS]VPENPKGGGGGKTIVPA[p
T]LPQLTP[COOH]

ID:GR_134_524
P7192-1 2 mg

Thermo SCIENTIFIC
800-874-3723 or 815-968-0747 • FOR RESEARCH USE ONLY
3747 N. Meridian Rd., Rockford, IL 61101 U.S.A.
www.thermoscientific.com/pierce

Column: 100X3.0mm 5u C18 120A
Solvent: A:0.1%TFA in Water; B: 0.08 % TFA in ACN
Flow Rate: 1 ml min

Chromatogram
P7192-1 C:\LabSolutions\Data\ANA1\P7192-1a.lcd

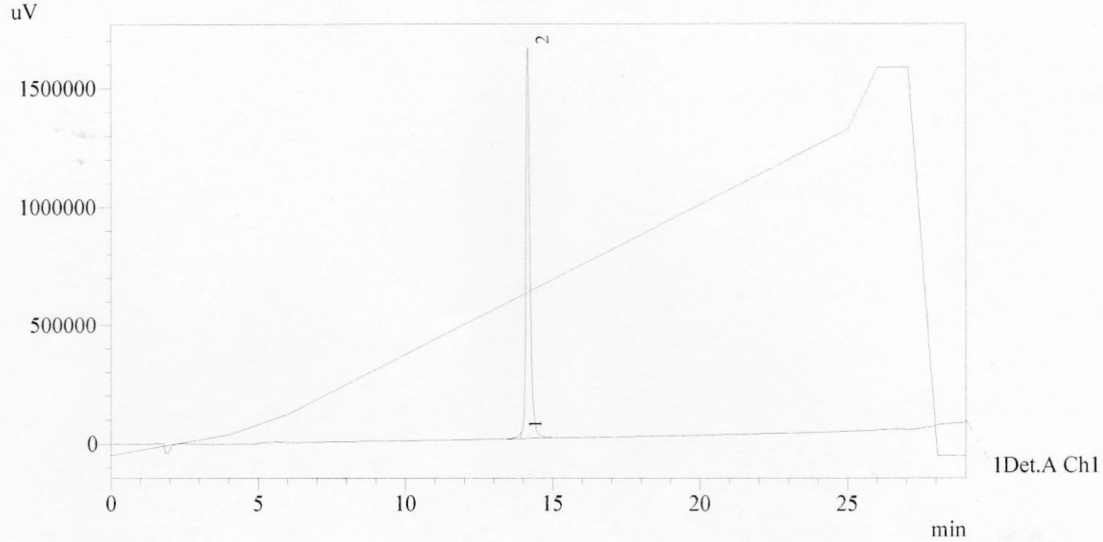

1 Det.A Ch1 / 220nm

PeakTable

Detector A Ch1 220nm

Peak#	Ret. Time	Area	Height	Height %	Area %
1	13.917	174473	22102	1.321	1.058
2	14.153	16314123	1651139	98.679	98.942
Total				100.000	100.000

Applied Biosystems Voyager System 1099

Voyager Spec #1=>SM5[BP = 3621.5, 1041]

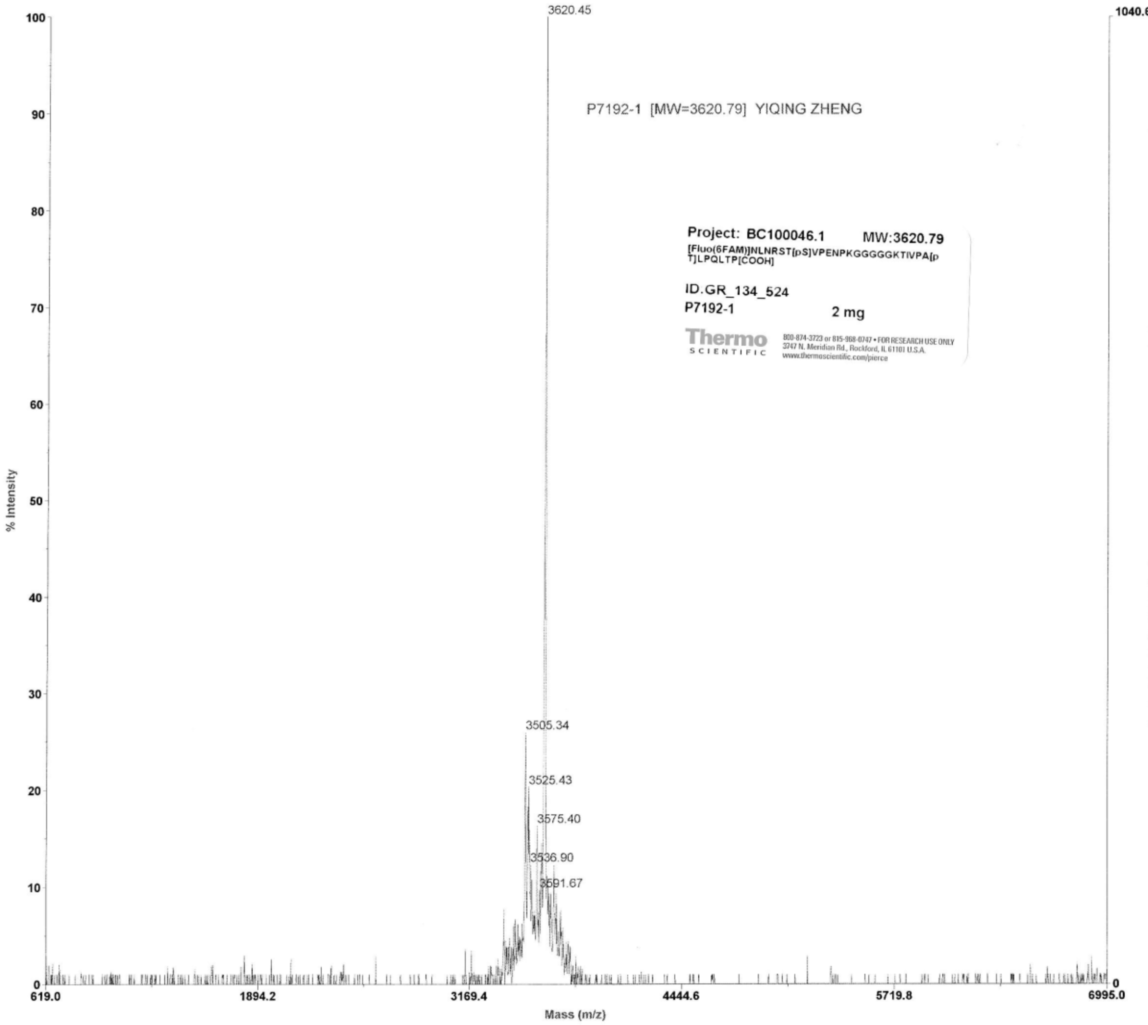

Mode of operation:	Linear
Extraction mode:	Delayed
Polarity:	Positive
Acquisition control:	Manual
Accelerating voltage:	20000 V
Grid voltage:	93%
Guide wire 0:	0.015%
Extraction delay time:	100 nsec
Acquisition mass range:	620 -- 7000 Da
Number of laser shots:	100/spectrum
Laser intensity:	2187
Laser Rep Rate:	3.0 Hz
Calibration type:	Default
Calibration matrix:	a-Cyano-4-hydroxycinnamic acic
Low mass gate:	Off
Digitizer start time:	16.214
Bin size:	2 nsec
Number of data points:	18994
Vertical scale:	200 mV
Vertical offset:	0%
Input bandwidth:	150 MHz
Sample well:	03
Plate ID:	100 WELL PLATE
Serial number:	1099
Instrument name:	Voyager-DE
Plate type filename:	C:\VOYAGER\100 well plate.plt
Lab name:	BioSynthesis, Inc
Absolute x-position:	11747.5
Absolute y-position:	47306.7
Relative x-position:	-0.000244521
Relative y-posititon:	-0.786014
Shots in spectrum:	5
Source pressure:	3.621e-007
Mirror pressure:	0
TC2 pressure:	0.001
TIS gate width:	30
TIS flight length:	940

Acquired: 02:09:00, January 17, 2018

Printed: 02:10, January 17, 2018

C:\VOYAGER\Data\Biosyn2018\P7192-1_0004.dat

FAM labelled GR_pS83-pS134:

Sample Information

Acquired by	: Admin
Sample Name	: P7192-3
Sample ID	: P7192-3
Data Filename	: P7192-3k.lcd
Method Filename	: ANAPEP1.lcm
Date Acquired	: 1/17/2018 1:44:52 AM
Data Processed	: 1/17/2018 9:28:51 PM

Project: BC100046.3 MW:3625.73
[Fluo(6FAM)]DLSKAV[pS]LSMGLYGGGGGNLNRST[pS]VPENPK[COOH]

ID:GR_83_134
P7192-3 2 mg

Thermo SCIENTIFIC 800-874-3723 or 815-968-0747 • FOR RESEARCH USE ONLY
3747 N. Meridian Rd., Rockford, IL 61101 U.S.A.
www.thermoscientific.com/pierce

Column:	100X3.0mm 5u C18 120A
Solvent:	A:0.1%TFA in Water; B: 0.08 % TFA in ACN
Flow Rate:	1 ml min

Chromatogram
P7192-3 C:\LabSolutions\Data\ANA1\P7192-3k.lcd

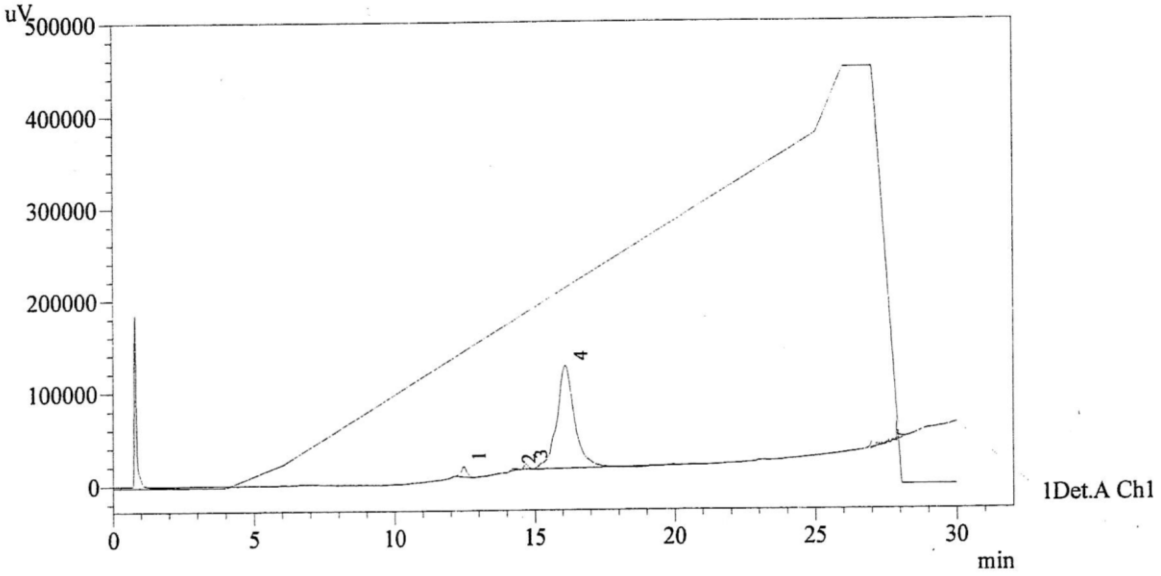

1 Det.A Ch1 / 220nm

PeakTable

Detector A Ch1 220nm

Peak#	Ret. Time	Area	Height	Height %	Area %
1	12.459	156743	11156	8.660	3.159
2	14.213	21346	1903	1.477	0.430
3	14.668	52413	5236	4.064	1.056
4	16.102	4730590	110532	85.799	95.354
Total				100.000	100.000

Applied Biosystems Voyager System 1099

Voyager Spec #1=>SM9=>SM11[BP = 3625.2, 155]

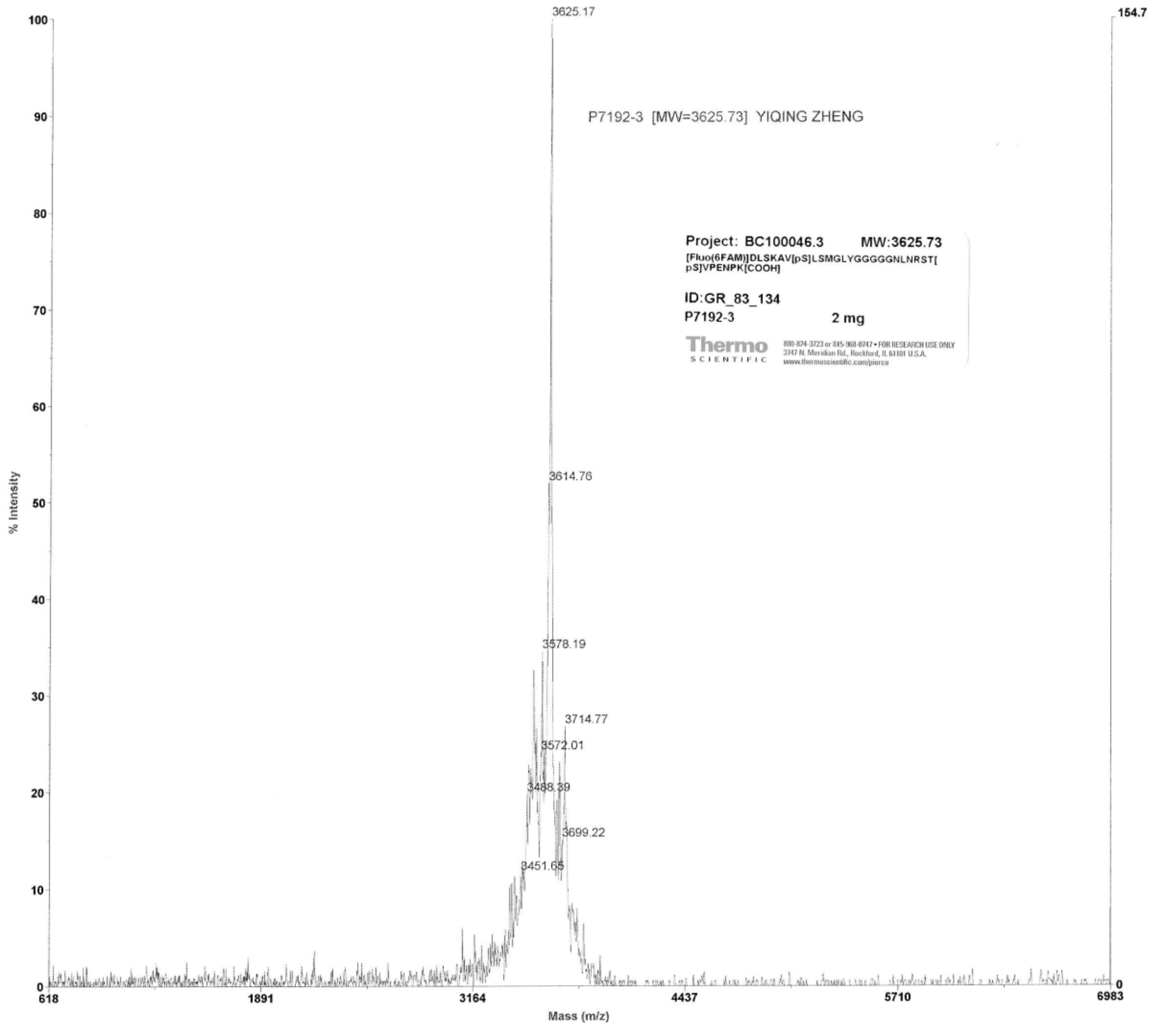

Mode of operation:	Linear
Extraction mode:	Delayed
Polarity:	Negative
Acquisition control:	Manual
Accelerating voltage:	20000 V
Grid voltage:	93%
Guide wire 0:	0.015%
Extraction delay time:	100 nsec
Acquisition mass range:	620 -- 7000 Da
Number of laser shots:	100/spectrum
Laser intensity:	2287
Laser Rep Rate:	3.0 Hz
Calibration type:	Default
Calibration matrix:	a-Cyano-4-hydroxycinnamic acic
Low mass gate:	Off
Digitizer start time:	16.212
Bin size:	2 nsec
Number of data points:	18991
Vertical scale:	200 mV
Vertical offset:	0%
Input bandwidth:	150 MHz
Sample well:	28
Plate ID:	100 WELL PLATE
Serial number:	1099
Instrument name:	Voyager-DE
Plate type filename:	C:\VOYAGER\100 well plate.plt
Lab name:	BioSynthesis, Inc
Absolute x-position:	37665.1
Absolute y-position:	37129.5
Relative x-position:	517.555
Relative y-posititon:	-18.0192
Shots in spectrum:	11
Source pressure:	2.103e-007
Mirror pressure:	0
TC2 pressure:	0.001
TIS gate width:	30
TIS flight length:	940

Acquired: 01:46:00, January 17, 2018

Printed: 01:46, January 17, 2018

C:\VOYAGER\Data\Biosyn2018\P7192-3_0006.dat

FITC labelled GR_K518A:

FITC labelled GR_K518A peptide was prepared following General Protocol for SPPS. The crude peptide was purified by reverse-phase HPLC (gradient: 5% B for 1 min, 5-30% B in 3min, 30-35% B in 15min, Waters Xselect CSH C18 column) to afford the desired peptide as a white fluffy solid. Yield (11 mg) in > 95% purity according to analytical UPLC. Rt 7.09 min (3-60% B in 10 min).

$[M + 3H]^{3+}$ calculated for $C_{164}H_{246}N_{42}O_{54}P_2S_2$, 1266.0; found 1266.0.

Sample ID: EN08354-60-001 <has no SN number> **Weight:** <has no weight> **Purity:** 95,07 %

UV Chromatogram

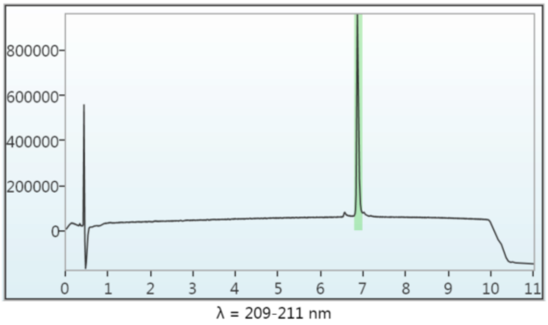

MS Spectrum (+) for selected peak

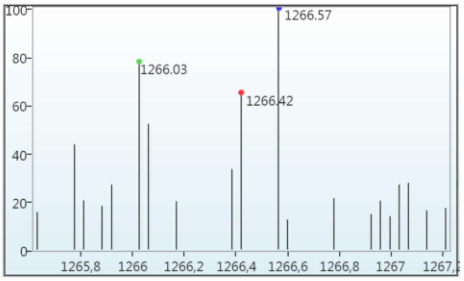

MS Spectrum (-) for selected peak

N/A

FITC labelled GR_T519A:

FITC labelled GR_T519A peptide was prepared following General Protocol for SPPS. The crude peptide was purified by reverse-phase HPLC (gradient: 5% B for 1 min, 31-40% B in 30min, Kromasil C18 column) to afford the desired peptide as a white fluffy solid. Yield (3 mg) in > 95% purity according to analytical UPLC. Rt 4.30 min (5-95% B in 10 min).

$[M + 3H]^{3+}$ calculated for $C_{166}H_{251}N_{43}O_{53}P_2S_2$, 1275.0; found 1276.3.

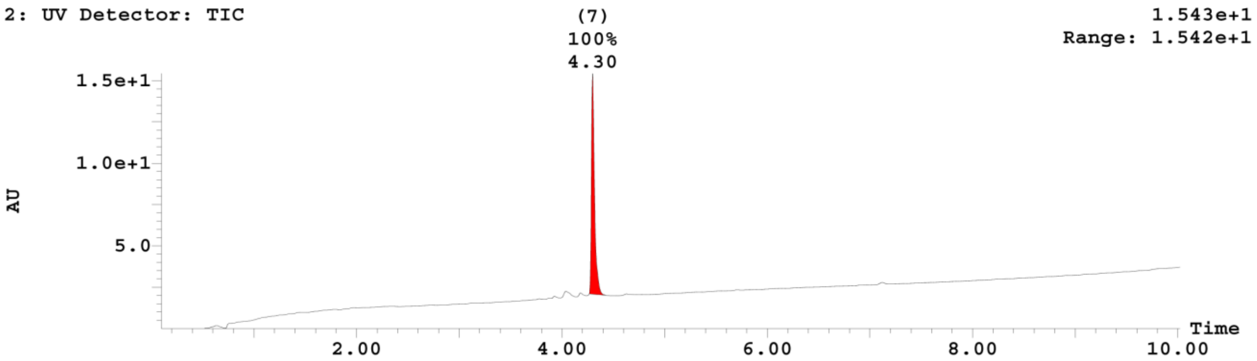

Peak Number	Compound	Time	AreaAbs	Area %Total	Width	Height	Mass Found
7		4.30	5e+005	100.00	0	1e+007	

Peak ID	Time	Mass Found
7	4.30	

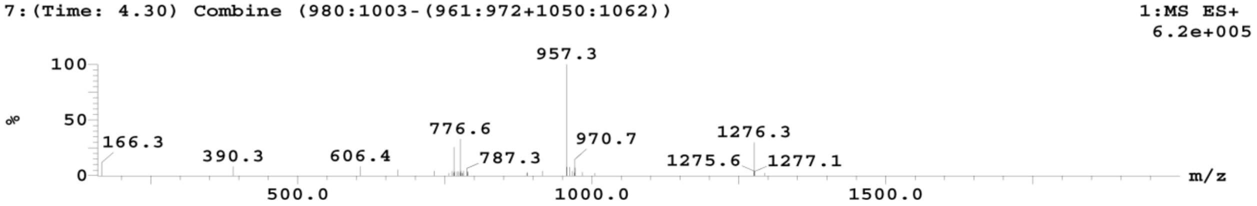

FITC labelled GR_I520A:

FITC labelled GR_I520A peptide was prepared following General Protocol for SPPS. The crude peptide was purified by reverse-phase HPLC (gradient: 5% B for 1 min, 5-29% B in 3 min, 29-34% B in 15min, Waters Atlantis T3 column) to afford the desired peptide as a white fluffy solid. Yield (13 mg) in > 95% purity according to analytical UPLC. Rt 6.14 min (3-60% B in 10 min).

$[M + 3H]^{3+}$ calculated for $C_{164}H_{247}N_{43}O_{54}P_2S_2$, 1271.0; found 1271.1.

Sample ID: EN08354-60-003 <has no SN number> **Weight:** <has no weight> **Purity:** 99,81 %

UV Chromatogram

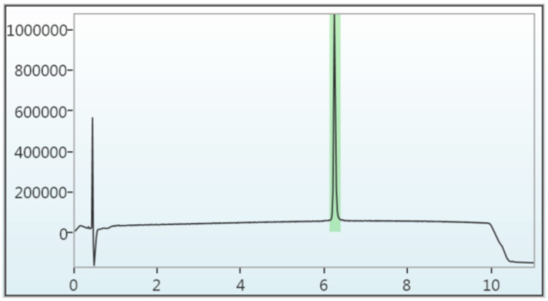

MS Spectrum (+) for selected peak

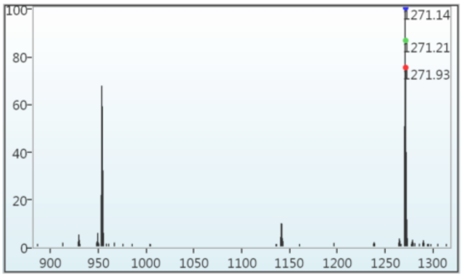

MS Spectrum (-) for selected peak

N/A

FITC labelled GR_V521A:

FITC labelled GR_V521A peptide was prepared following General Protocol for SPPS. The crude peptide was purified by reverse-phase HPLC (gradient: 5% B for 1 min, 5-29% B in 3 min, 29-34% B in 15min, Waters Atlantis T3 column) to afford the desired peptide as a white fluffy solid. Yield (2 mg) in > 95% purity according to analytical UPLC. Rt 6.24 min (3-60% B in 10 min).

$[M + 3H]^{3+}$ calculated for $C_{165}H_{249}N_{43}O_{54}P_2S_2$, 1275.7; found 1275.1.

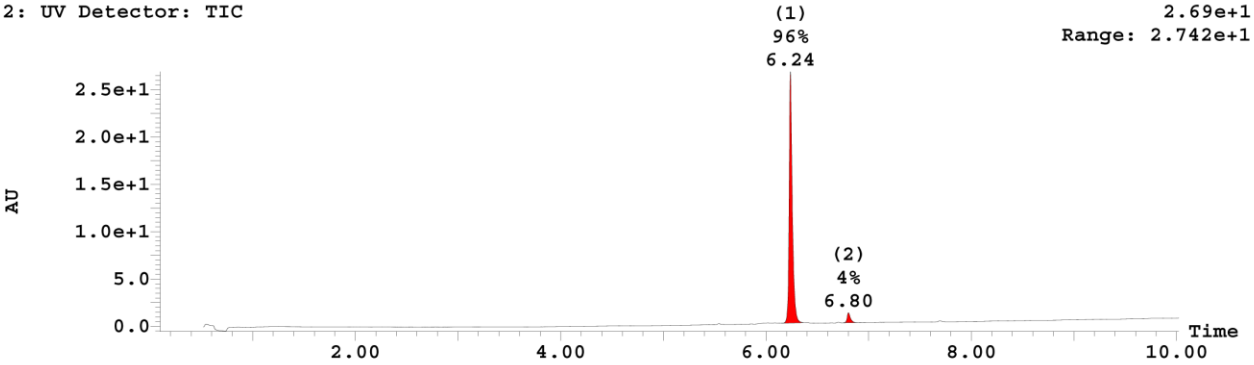

Peak Number	Compound	Time	AreaAbs	Area %Total	Width	Height	Mass Found
1		6.24	1e+006	96.03	0	3e+007	
2		6.80	4e+004	3.97	0	1e+006	

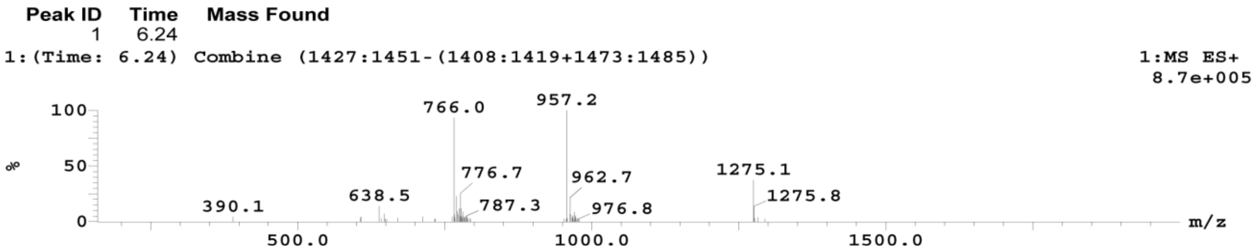

FITC labelled GR_P522A:

FITC labelled GR_P522A peptide was prepared following General Protocol for SPPS. The crude peptide was purified by reverse-phase HPLC (gradient: 5% B for 1 min, 5-29% B in 3 min, 29-34% B in 15min, Waters Atlantis T3 column) to afford the desired peptide as a white fluffy solid. Yield (13 mg) in > 95% purity according to analytical UPLC. Rt 4.22 min (5-95% B in 10 min).

$[M + 3H]^{3+}$ calculated for $C_{165}H_{251}N_{43}O_{54}P_2S_2$, 1276.4; found 1276.4.

Sample ID: EN08354-57-001 <has no SN number> **Weight:** <has no weight> **Purity:** 97,91 %

UV Chromatogram

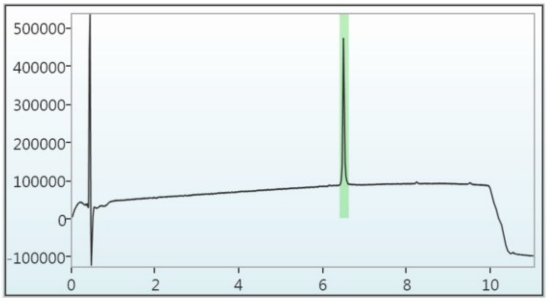

MS Spectrum (+) for selected peak

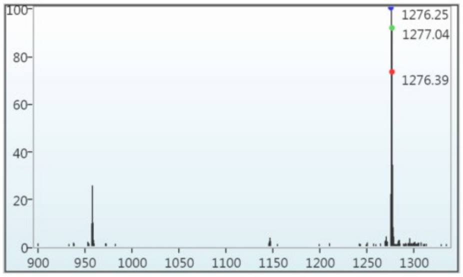

MS Spectrum (-) for selected peak

N/A

FITC labelled GR_T524A-pS617:

FITC labelled GR_T524A-pS617 peptide was prepared following General Protocol for SPPS. The crude peptide was purified by reverse-phase HPLC (gradient: 5% B for 1 min, 34-37% B in 25 min, Kromasil C18 column) to afford the desired peptide as a white fluffy solid. Yield (8 mg) in > 95% purity according to analytical UPLC. Rt 6.11 min (3-60% B in 10 min).

$[M + 3H]^{3+}$ calculated for $C_{166}H_{250}N_{43}O_{50}PS_2$, 1248.4; found 1249.2.

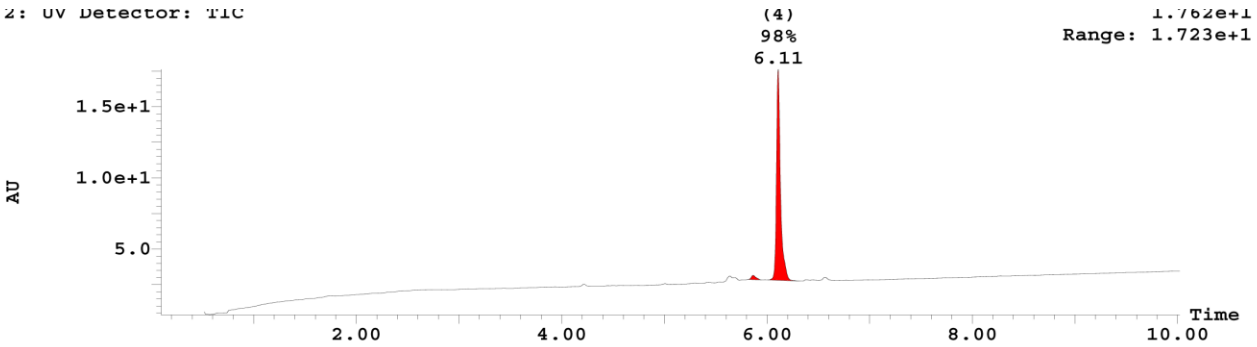

Peak Number	Compound	Time	AreaAbs	Area %Total	Width	Height	Mass Found
3		5.86	2e+004	2.33	0	3e+005	
4		6.11	7e+005	97.67	0	1e+007	

Peak ID	Time	Mass Found
4	6.10	

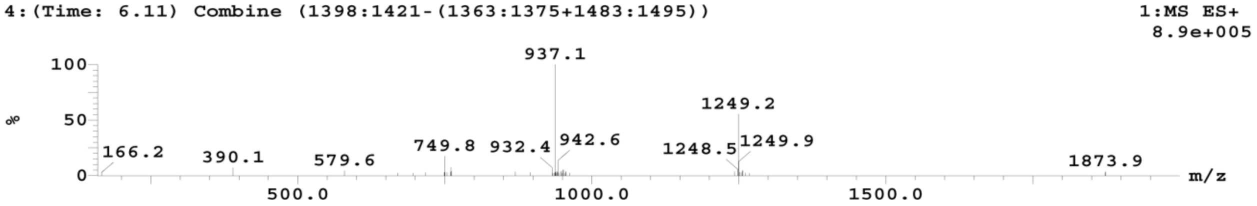

FITC labelled GR_L525A:

FITC labelled GR_L525A peptide was prepared following General Protocol for SPPS. The crude peptide was purified by reverse-phase HPLC (gradient: 5% B for 1 min, 5-28% B in 3 min, 28-33% B in 15min, Waters Atlantis T3 column) to afford the desired peptide as a white fluffy solid. Yield (20 mg) in > 95% purity according to analytical UPLC. Rt 4.05 min (5-95% B in 10 min).

$[M + 3H]^{3+}$ calculated for $C_{164}H_{247}N_{43}O_{54}P_2S_2$, 1271.0; found 1271.1.

Sample ID: EN08354-57-003 <has no SN number> **Weight:** <has no weight> **Purity:** 99,03 %

UV Chromatogram

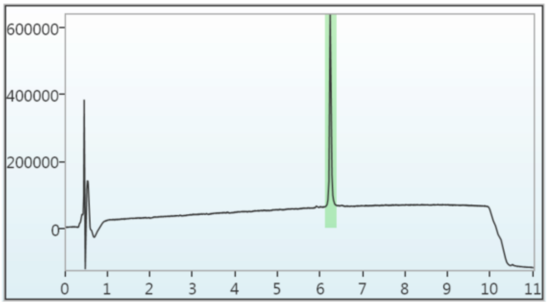

MS Spectrum (+) for selected peak

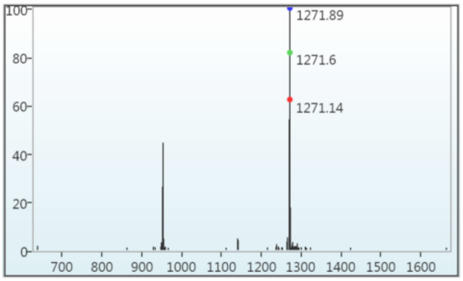

MS Spectrum (-) for selected peak

N/A

FITC labelled GR_P526A:

FITC labelled GR_P526A peptide was prepared following General Protocol for SPPS. The crude peptide was purified by reverse-phase HPLC (gradient: 5% B for 1 min, 5-30% B in 3 min, 30-35% B in 15min, Waters Atlantis T3 column) to afford the desired peptide as a white fluffy solid. Yield (3 mg) in > 95% purity according to analytical UPLC. Rt 6.49 min (3-60% B in 10 min).

$[M + 3H]^{3+}$ calculated for $C_{165}H_{251}N_{43}O_{54}P_2S_2$, 1276.4; found 1276.4.

Sample ID: EN08354-58-002 <has no SN number> **Weight:** <has no weight> **Purity:** 97,47 %

UV Chromatogram

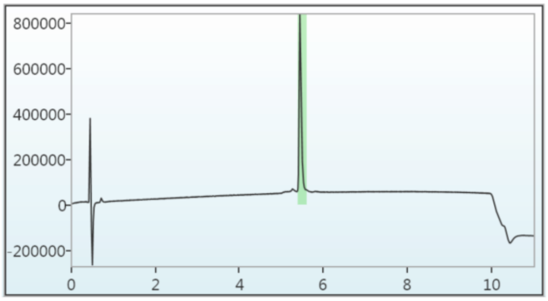

λ = 209-211 nm

MS Spectrum (+) for selected peak

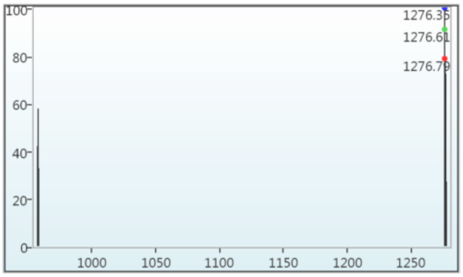

MS Spectrum (-) for selected peak

N/A

FITC labelled GR_Q527A:

FITC labelled GR_Q527A peptide was prepared following General Protocol for SPPS. The crude peptide was purified by reverse-phase HPLC (gradient: 5% B for 1 min, 5-31% B in 3 min, 31-36% B in 15min, Waters Atlantis T3 column) to afford the desired peptide as a white fluffy solid. Yield (5 mg) in > 95% purity according to analytical UPLC. Rt 6.53 min (3-60% B in 10 min).

$[M + 3H]^{3+}$ calculated for $C_{165}H_{250}N_{42}O_{53}P_2S_2$, 1266.0; found 1266.1.

Sample ID: EN08354-60-004 <has no SN number> **Weight:** <has no weight> **Purity:** 98,31 %

UV Chromatogram

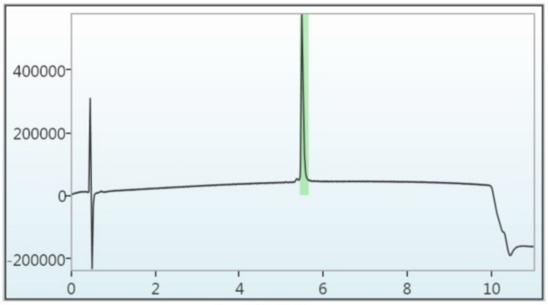

λ = 209-211 nm

MS Spectrum (+) for selected peak

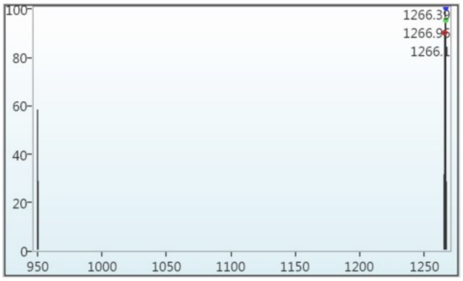

MS Spectrum (-) for selected peak

N/A

FITC labelled GR_L528A:

FITC labelled GR_L528A peptide was prepared following General Protocol for SPPS. The crude peptide was purified by reverse-phase HPLC (gradient: 5% B for 1 min, 5-27% B in 3 min, 27-32% B in 15min, Waters XSelect CSH C18 column) to afford the desired peptide as a white fluffy solid. Yield (18 mg) in > 95% purity according to analytical UPLC. Rt 6.16 min (3-60% B in 10 min).

$[M + 3H]^{3+}$ calculated for $C_{164}H_{247}N_{43}O_{54}P_2S_2$, 1271.0; found 1270.5.

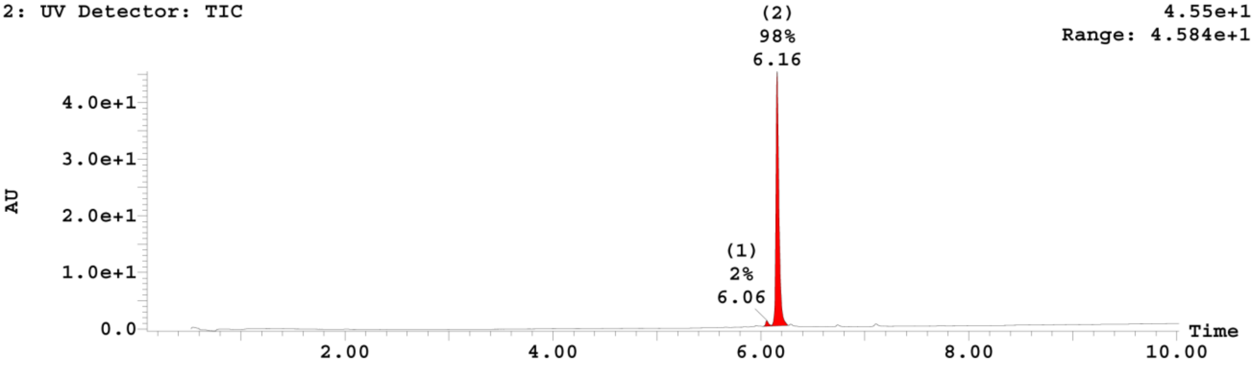

Peak Number	Compound	Time	AreaAbs	Area %Total	Width	Height	Mass Found
1		6.06	3e+004	2.11	0	1e+006	
2		6.16	2e+006	97.89	0	4e+007	

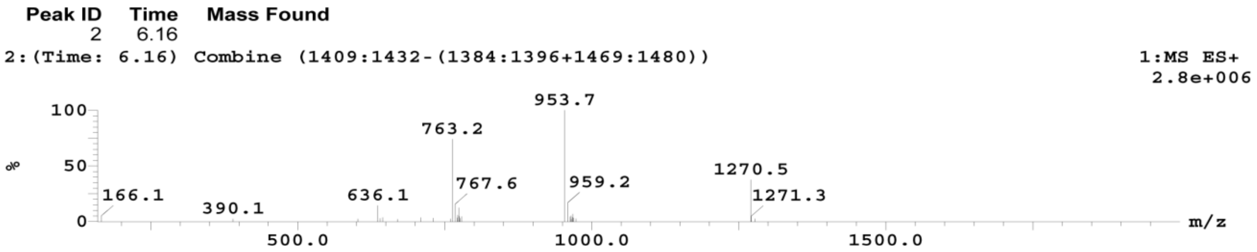

FITC labelled GR_T529A:

FITC labelled GR_T529A peptide was prepared following General Protocol for SPPS. The crude peptide was purified by reverse-phase HPLC (gradient: 5% B for 1 min, 5-30% B in 3 min, 30-35% B in 15min, Waters Atlantis T3 column) to afford the desired peptide as a white fluffy solid. Yield (13 mg) in > 95% purity according to analytical UPLC. Rt 6.43 min (3-60% B in 10 min).

$[M + 3H]^{3+}$ calculated for $C_{166}H_{251}N_{43}O_{53}P_2S_2$, 1275.0; found 1275.6.

Sample ID: EN08354-60-006 <has no SN number> **Weight:** <has no weight> **Purity:** 99,96 %

UV Chromatogram

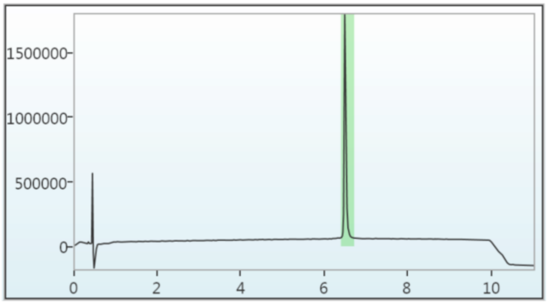

MS Spectrum (+) for selected peak

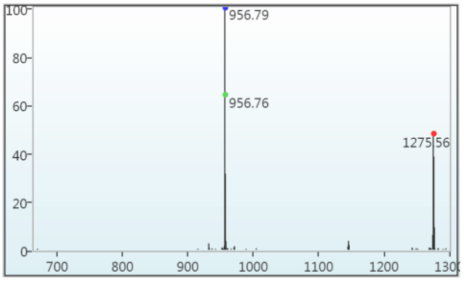

MS Spectrum (-) for selected peak

N/A

FITC labelled GR_P530A:

FITC labelled GR_P530A peptide was prepared following General Protocol for SPPS. The crude peptide was purified by reverse-phase HPLC (gradient: 5% B for 1 min, 5-30% B in 3 min, 30-35% B in 15min, Waters CSH C18 column) to afford the desired peptide as a white fluffy solid. Yield (8 mg) in 95% purity according to analytical UPLC. Rt 6.30 min (3-60% B in 10 min).

$[M + 3H]^{3+}$ calculated for $C_{165}H_{251}N_{43}O_{54}P_2S_2$, 1276.4; found 1275.9.

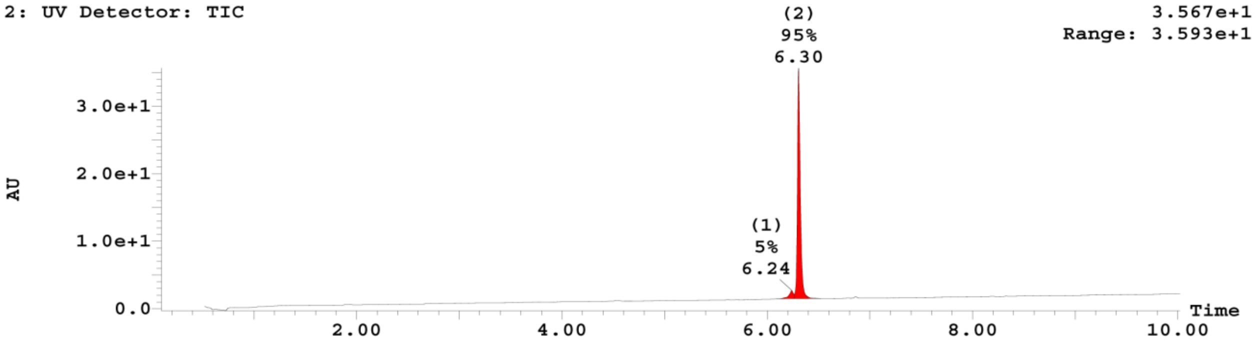

Peak Number	Compound	Time	AreaAbs	Area %Total	Width	Height	Mass Found
1		6.24	6e+004	5.03	0	1e+006	
2		6.30	1e+006	94.97	0	3e+007	

Peak ID	Time	Mass Found
2	6.30	

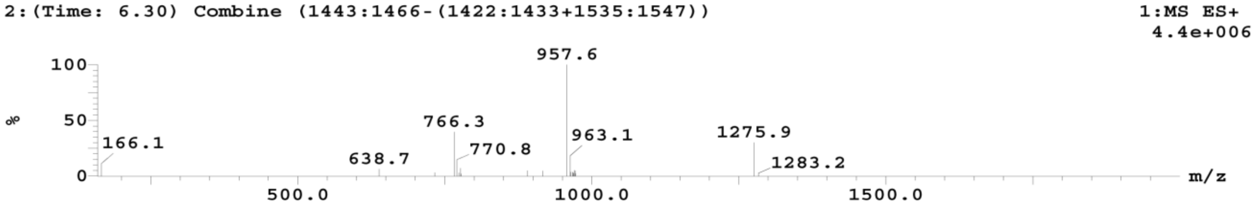

FITC labelled GR_R611A:

FITC labelled GR_R611A peptide was prepared following General Protocol for SPPS. The crude peptide was purified by reverse-phase HPLC (gradient: 5% B for 1 min, 5-29% B in 3 min, 29-34% B in 15min, Waters CSH C18 column) to afford the desired peptide as a white fluffy solid. Yield (2 mg) in > 95% purity according to analytical UPLC. Rt 6.74 min (3-60% B in 10 min).

$[M + 3H]^{3+}$ calculated for $C_{164}H_{246}N_{40}O_{54}P_2S_2$, 1256.7; found 1256.1.

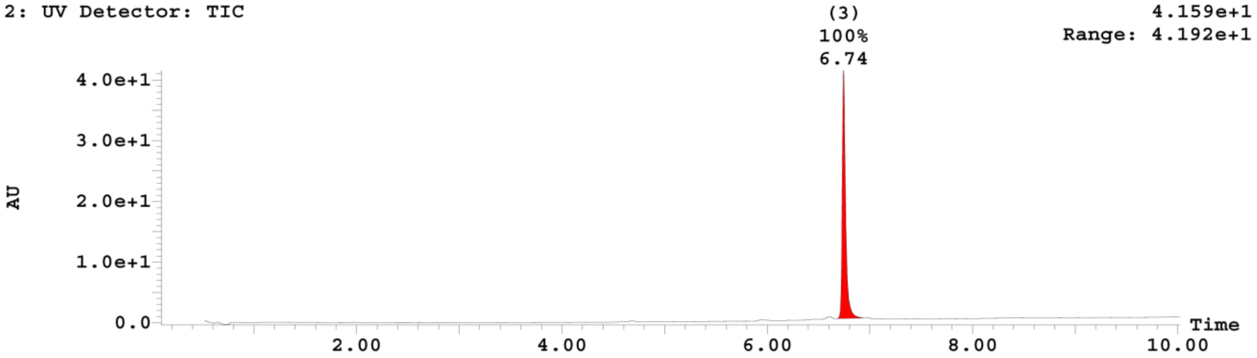

Peak Number	Compound	Time	AreaAbs	Area %Total	Width	Height	Mass Found
3		6.74	2e+006	100.00	0	4e+007	

Peak ID	Time	Mass Found
3	6.74	

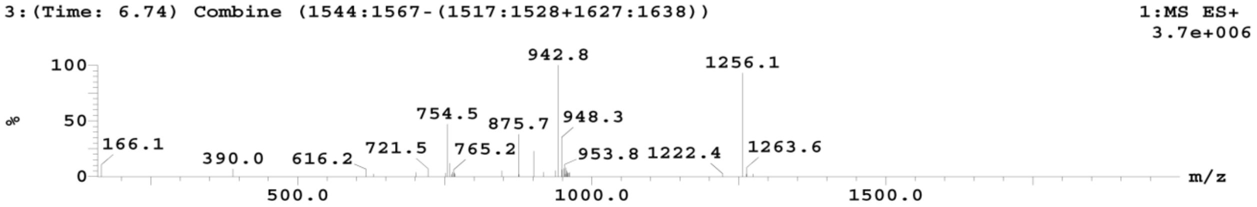

FITC labelled GR_S612A:

FITC labelled GR_S612A peptide was prepared following General Protocol for SPPS. The crude peptide was purified by reverse-phase HPLC (gradient: 5% B for 1 min, 5-28% B in 3 min, 28-33% B in 15min, Waters CSH C18 column) to afford the desired peptide as a white fluffy solid. Yield (8 mg) in > 95% purity according to analytical UPLC. Rt 6.36 min (3-60% B in 10 min).

[M + 3H]$^{3+}$ calculated for $C_{167}H_{253}N_{43}O_{53}P_2S_2$, 1279.7; found 1279.2.

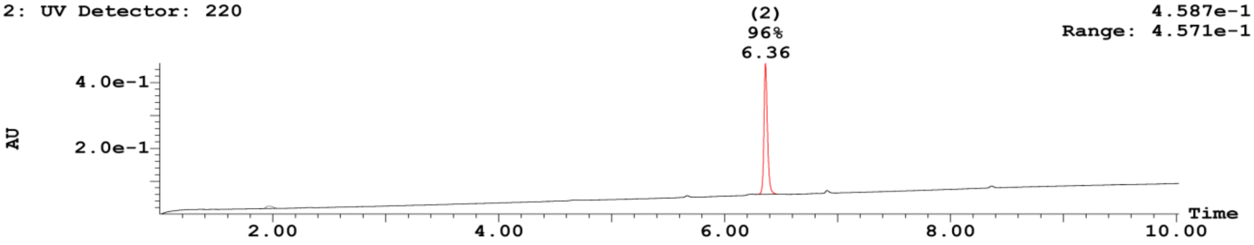

Peak ID	Time	Mass Found
2	6.36	

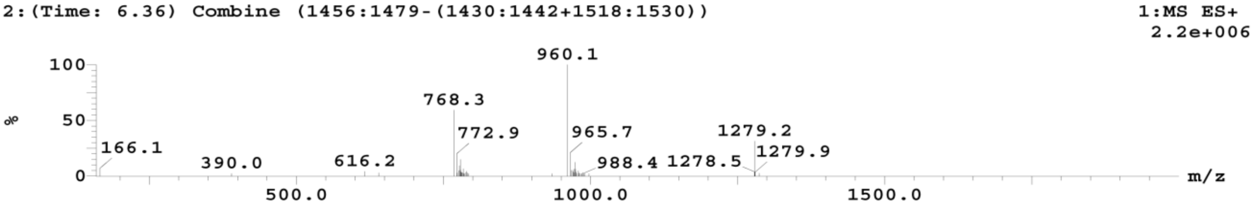

FITC labelled GR_Y613A:

FITC labelled GR_Y613A peptide was prepared following General Protocol for SPPS. The crude peptide was purified by reverse-phase HPLC (gradient: 5% B for 1 min, 5-27% B in 3 min, 27-32% B in 15min, Waters CSH C18 column) to afford the desired peptide as a white fluffy solid. Yield (6 mg) in > 95% purity according to analytical UPLC. Rt 6.30 min (3-60% B in 10 min).

[M + 3H]$^{3+}$ calculated for $C_{161}H_{249}N_{43}O_{53}P_2S_2$, 1254.3; found 1253.7.

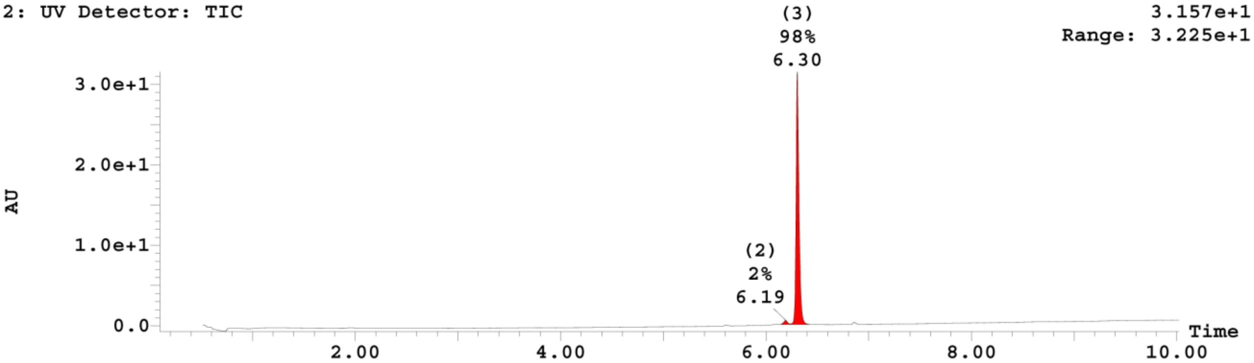

Peak Number	Compound	Time	AreaAbs	Area %Total	Width	Height	Mass Found
2		6.19	2e+004	2.09	0	5e+005	
3		6.30	1e+006	97.91	0	3e+007	

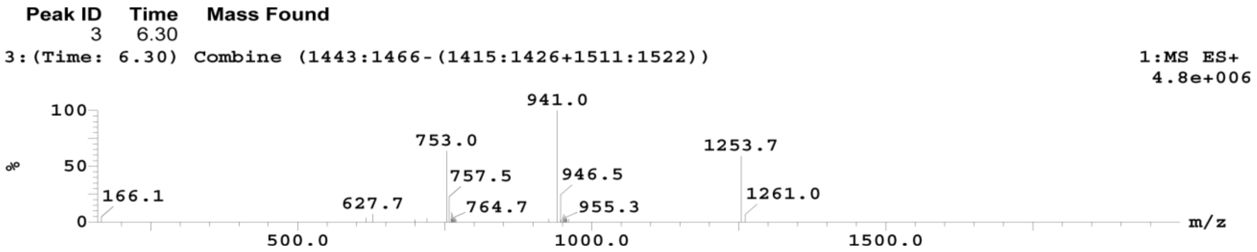

FITC labelled GR_R614A:

FITC labelled GR_R614A peptide was prepared following General Protocol for SPPS. The crude peptide was purified by reverse-phase HPLC (gradient: 5% B for 1 min, 5-31% B in 3 min, 31-36% B in 15min, Waters Atlantis T3 column) to afford the desired peptide as a white fluffy solid. Yield (1 mg) in > 95% purity according to analytical UPLC. Rt 6.83 min (3-60% B in 10 min).

$[M + 3H]^{3+}$ calculated for $C_{164}H_{246}N_{40}O_{54}P_2S_2$, 1256.7; found 1256.9.

Sample ID: EN08354-58-003 <has no SN number> **Weight:** <has no weight> **Purity:** 97 %

UV Chromatogram

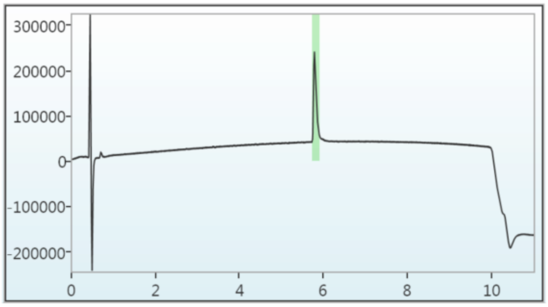

λ = 209-211 nm

MS Spectrum (+) for selected peak

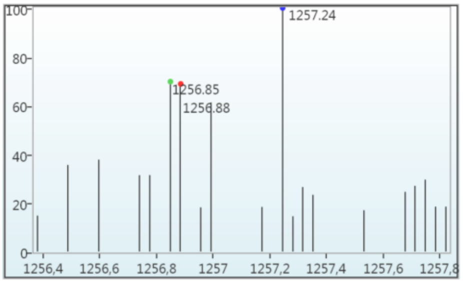

MS Spectrum (-) for selected peak

N/A

FITC labelled GR_Q615A:

FITC labelled GR_Q615A peptide was prepared following General Protocol for SPPS. The crude peptide was purified by reverse-phase HPLC (gradient: 5% B for 1 min, 25-35% B in 35 min, Kromasil C18 column) to afford the desired peptide as a white fluffy solid. Yield (7 mg) in > 95% purity according to analytical UPLC. Rt 6.39 min (3-60% B in 10 min).

$[M + 3H]^{3+}$ calculated for $C_{165}H_{250}N_{42}O_{53}P_2S_2$, 1266.0; found 1266.9.

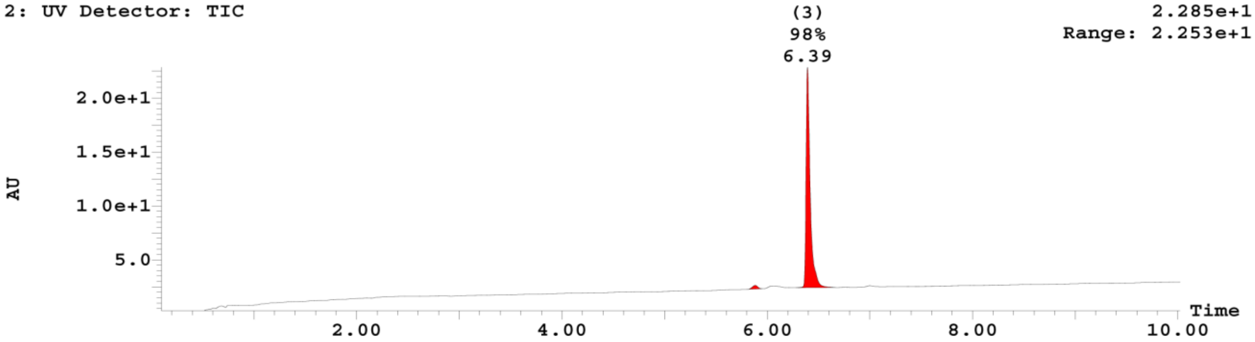

Peak Number	Compound	Time	AreaAbs	Area %Total	Width	Height	Mass Found
2		5.88	2e+004	2.14	0	3e+005	
3		6.39	1e+006	97.86	0	2e+007	

Peak ID	Time	Mass Found
3	6.39	

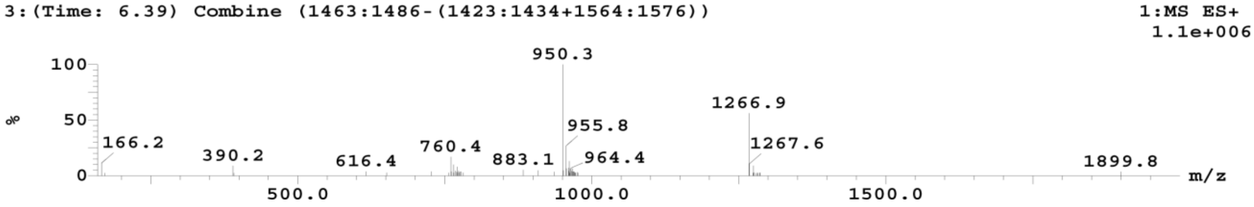

FITC labelled GR_S616A:

FITC labelled GR_S616A peptide was prepared following General Protocol for SPPS. The crude peptide was purified by reverse-phase HPLC (gradient: 5% B for 1 min, 5-29% B in 3 min, 29-34% B in 15min, Waters Atlantis T3 column) to afford the desired peptide as a white fluffy solid. Yield (16 mg) in > 95% purity according to analytical UPLC. Rt 4.29 min (5-95% B in 10 min).

$[M + 3H]^{3+}$ calculated for $C_{167}H_{253}N_{43}O_{53}P_2S_2$, 1279.7; found 1279.8.

Sample ID: EN08354-57-004 <has no SN number> **Weight:** <has no weight> **Purity:** 96,03 %

UV Chromatogram

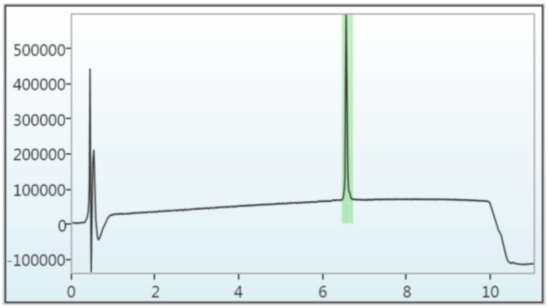

MS Spectrum (+) for selected peak

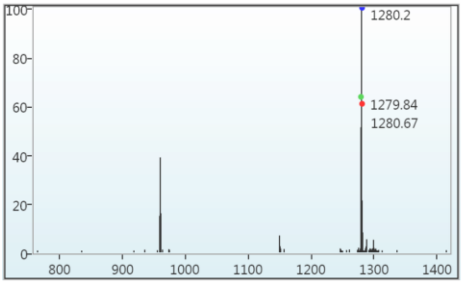

MS Spectrum (-) for selected peak

N/A

FITC labelled GR_pT524-S617A:

FITC labelled GR_pT524-S617A peptide was prepared following General Protocol for SPPS. The crude peptide was purified by reverse-phase HPLC (gradient: 5% B for 1 min, 34-37% B in 25 min, Kromasil C18 column) to afford the desired peptide as a white fluffy solid. Yield (2 mg) in > 95% purity according to analytical UPLC. Rt 6.17 min (3-60% B in 10 min).

$[M + 3H]^{3+}$ calculated for $C_{167}H_{252}N_{43}O_{50}PS_2$, 1253.1; found 1253.8.

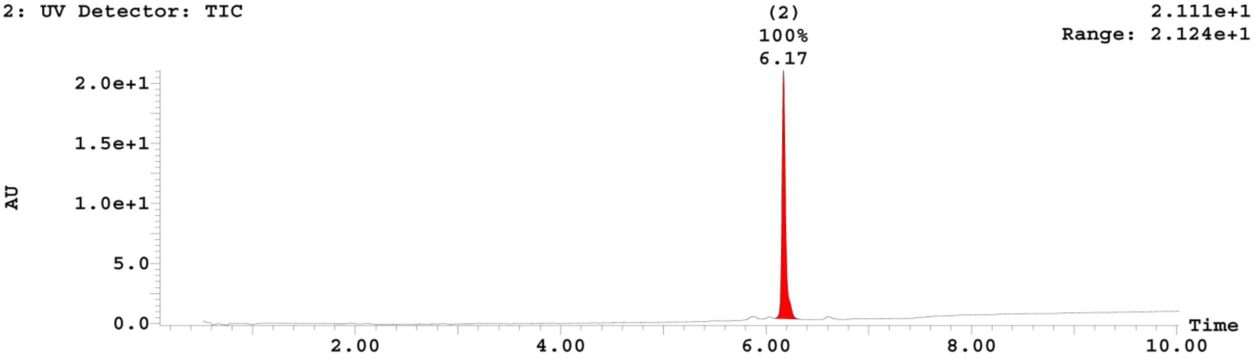

Peak Number	Compound	Time	AreaAbs	Area %Total	Width	Height	Mass Found
2		6.17	9e+005	100.00	0	2e+007	

Peak ID	Time	Mass Found
2	6.16	

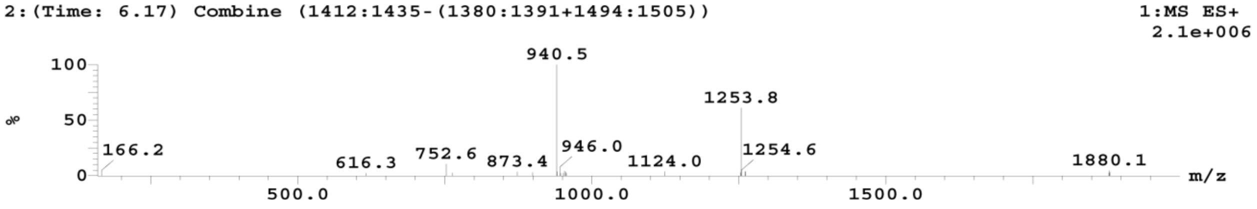

FITC labelled GR_N619A:

FITC labelled GR_N619A peptide was prepared following General Protocol for SPPS. The crude peptide was purified by reverse-phase HPLC (gradient: 5% B for 1 min, 26-34% B in 35min, Kromasil C18 column) to afford the desired peptide as a white fluffy solid. Yield (9 mg) in > 95% purity according to analytical UPLC. Rt 6.41 min (3-60% B in 10 min).

$[M + 3H]^{3+}$ calculated for $C_{166}H_{252}N_{42}O_{53}P_2S_2$, 1270.7; found 1271.4.

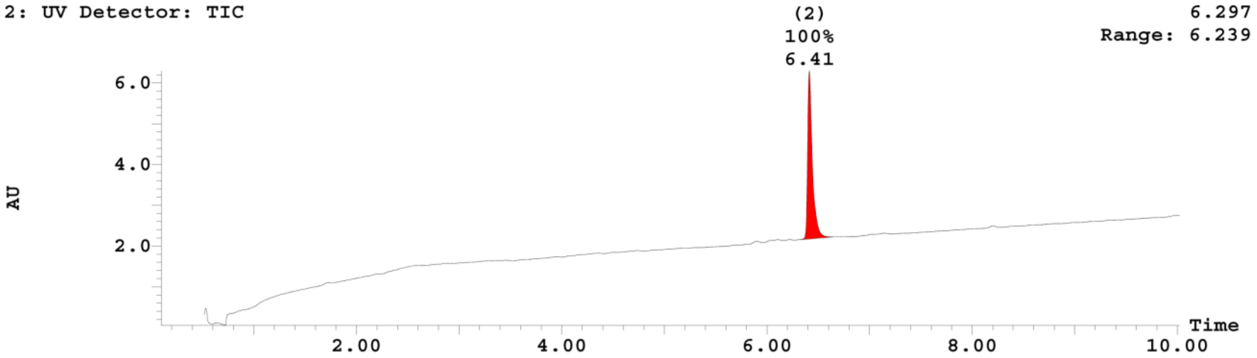

Peak Number	Compound	Time	AreaAbs	Area %Total	Width	Height	Mass Found
2		6.41	2e+005	100.00	0	4e+006	

Peak ID	Time	Mass Found
2	6.41	

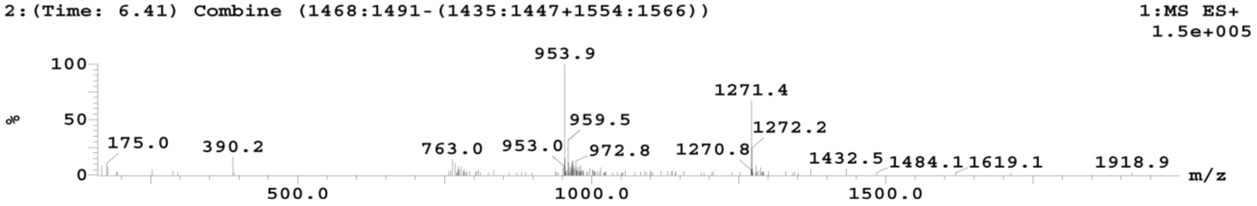

FITC labelled GR_L620A:

FITC labelled GR_L620A peptide was prepared following General Protocol for SPPS. The crude peptide was purified by reverse-phase HPLC (gradient: 5% B for 1 min, 5-29% B in 3 min, 29-34% B in 15min, Waters Atlantis T3 column) to afford the desired peptide as a white fluffy solid. Yield (1 mg) in > 95% purity according to analytical UPLC. Rt 6.16 min (3-60% B in 10 min).

$[M + 3H]^{3+}$ calculated for $C_{164}H_{247}N_{43}O_{54}P_2S_2$, 1271.0; found 1270.6.

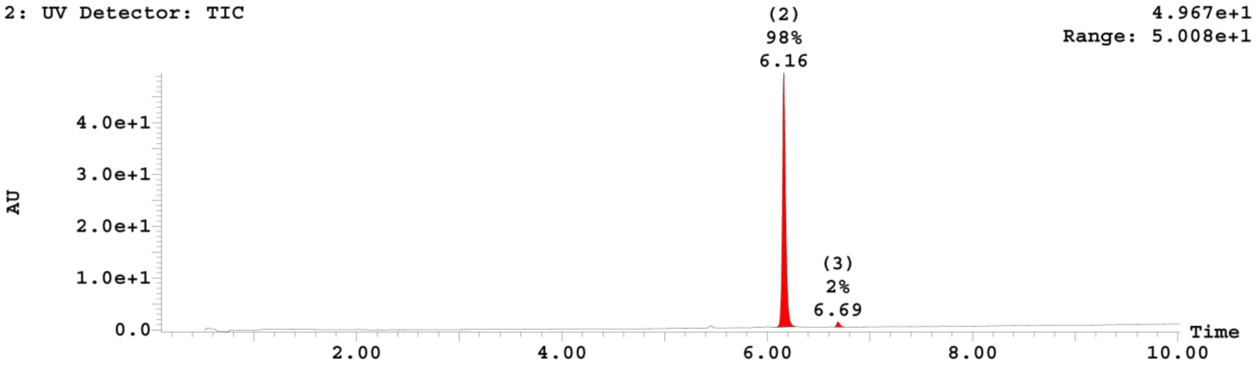

Peak Number	Compound	Time	AreaAbs	Area %Total	Width	Height	Mass Found
2		6.16	2e+006	97.89	0	5e+007	
3		6.69	4e+004	2.11	0	1e+006	

Peak ID	Time	Mass Found
2	6.16	

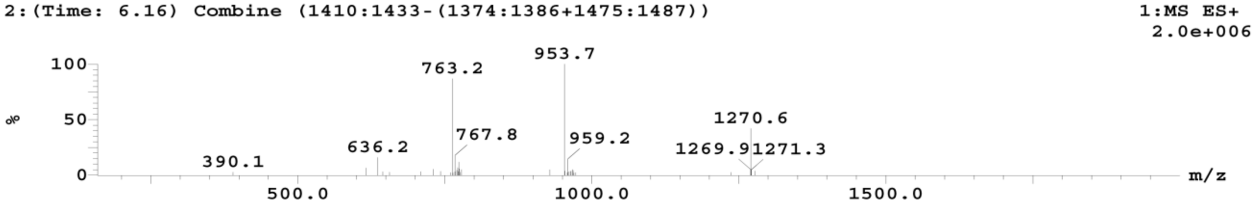

FITC labelled GR_L621A:

FITC labelled GR_L621A peptide was prepared following General Protocol for SPPS. The crude peptide was purified by reverse-phase HPLC (gradient: 5% B for 1 min, 5-26% B in 3 min, 26-31% B in 15min, Waters CSH C18 column) to afford the desired peptide as a white fluffy solid. Yield (9 mg) in > 95% purity according to analytical UPLC. Rt 6.01 min (3-60% B in 10 min).

$[M + 3H]^{3+}$ calculated for $C_{164}H_{247}N_{43}O_{54}P_2S_2$, 1271.0; found 1271.0.

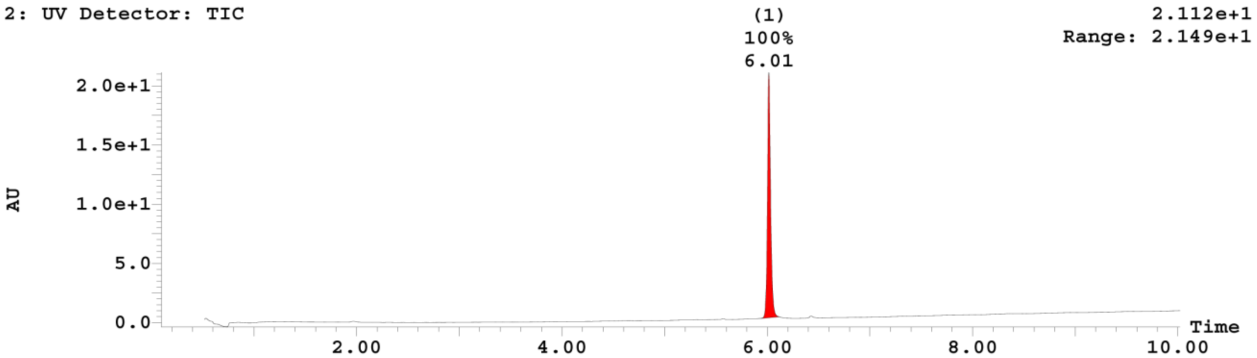

Peak Number	Compound	Time	AreaAbs	Area %Total	Width	Height	Mass Found
1		6.01	7e+005	100.00	0	2e+007	

Peak ID	Time	Mass Found
1	6.01	

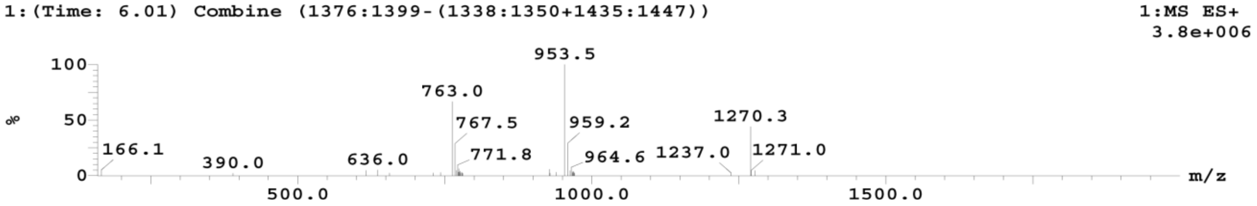

FITC labelled GR_C622A:

FITC labelled GR_C622A peptide was prepared following General Protocol for SPPS. The crude peptide was purified by reverse-phase HPLC (gradient: 5% B for 1 min, 5-27% B in 3 min, 27-32% B in 15min, Waters CSH C18 column) to afford the desired peptide as a white fluffy solid. Yield (5 mg) in > 95% purity according to analytical UPLC. Rt 6.24 min (3-60% B in 10 min).

$[M + 3H]^{3+}$ calculated for $C_{167}H_{253}N_{43}O_{54}P_2S$, 1274.4; found 1273.6.

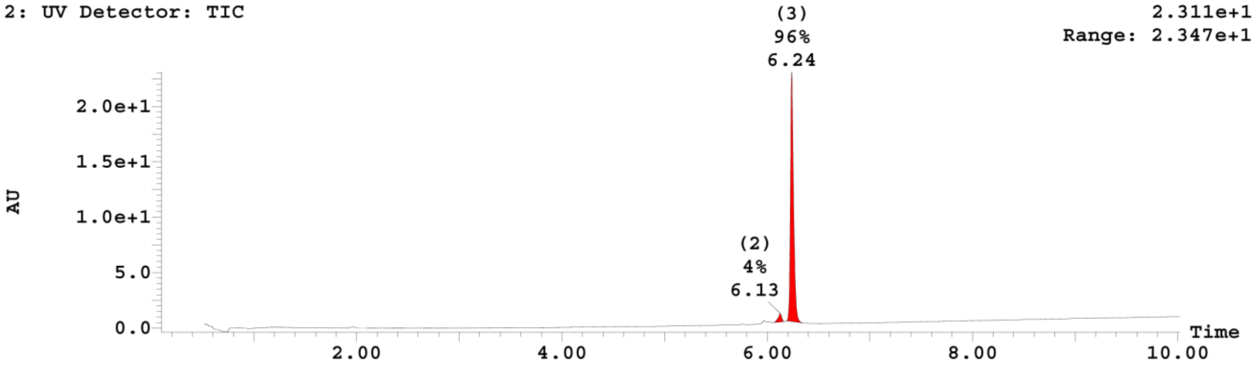

Peak Number	Compound	Time	AreaAbs	Area %Total	Width	Height	Mass Found
2		6.13	3e+004	3.75	0	8e+005	
3		6.24	8e+005	96.25	0	2e+007	

Peak ID	Time	Mass Found
3	6.23	

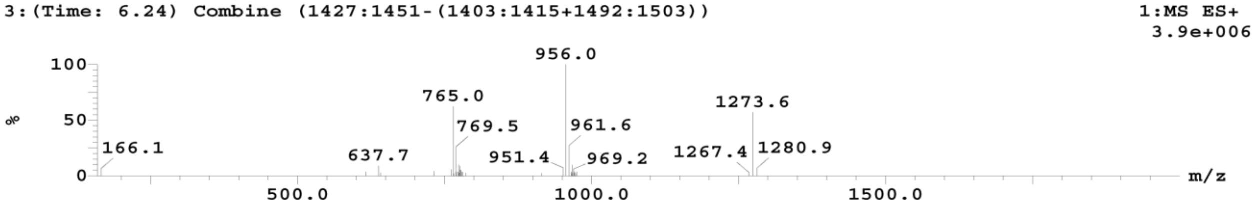

FITC labelled GR_F623A:

FITC labelled GR_F623A peptide was prepared following General Protocol for SPPS. The crude peptide was purified by reverse-phase HPLC (gradient: 5% B for 1 min, 5-25% B in 3 min, 25-30% B in 15min, Waters CSH C18 column) to afford the desired peptide as a white fluffy solid. Yield (10 mg) in > 95% purity according to analytical UPLC. Rt 5.91 min (3-60% B in 10 min).

$[M + 3H]^{3+}$ calculated for $C_{161}H_{249}N_{43}O_{54}P_2S_2$, 1259.7; found 1259.1.

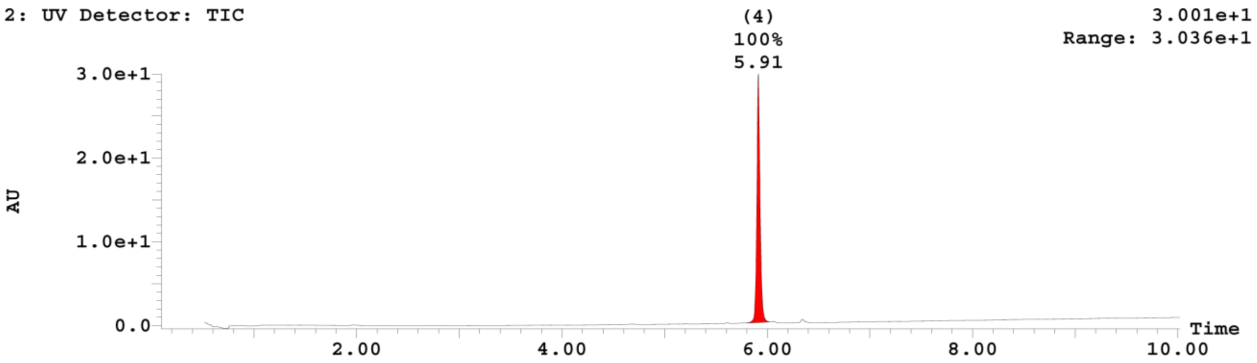

Peak Number	Compound	Time	AreaAbs	Area %Total	Width	Height	Mass Found
4		5.91	1e+006	100.00	0	3e+007	

Peak ID	Time	Mass Found
4	5.91	

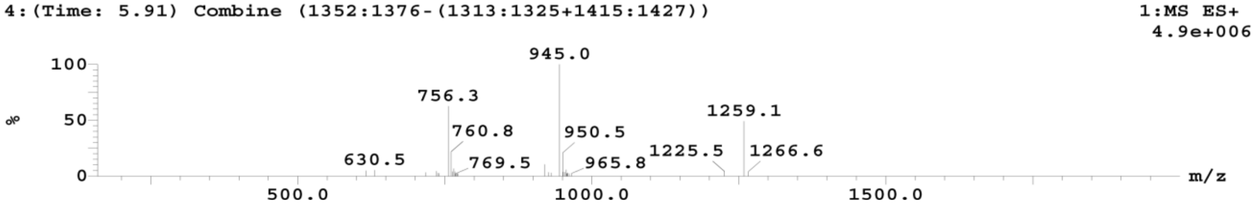

FITC labelled GR_pS524-pS617:

FITC labelled GR_pS524-pS617 peptide was prepared following General Protocol for SPPS. The crude peptide was purified by reverse-phase HPLC (gradient: 5% B for 1 min, 5-29% B in 3 min, 29-34% B in 15min, Waters Atlantis T3 column) to afford the desired peptide as a white fluffy solid. Yield (8 mg) in > 95% purity according to analytical UPLC. Rt 4.31 min (5-95% B in 10 min).

$[M + 3H]^{3+}$ calculated for $C_{166}H_{251}N_{43}O_{54}P_2S_2$, 1280.4; found 1280.7.

Sample ID: EN08354-57-006 <has no SN number> **Weight:** <has no weight> **Purity:** 99,49 %

UV Chromatogram

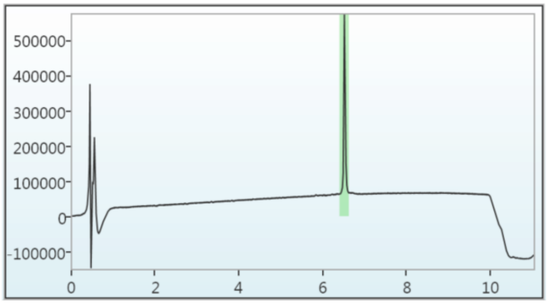

MS Spectrum (+) for selected peak

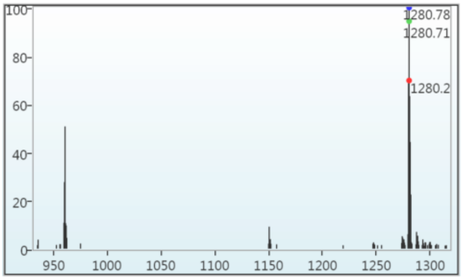

MS Spectrum (-) for selected peak

N/A

Acetylated GR_pT524-pT562:

Sample Information

Acquired by	: Admin
Sample Name	: A3059-1
Sample ID	: A3059-1
Data Filename	: A3059-1
Method Filename	: ANAPEP_2.lcm

Project: PKB100189.1 MW: 5734.45
[Acetyl]KTIVPA[(pT)]LPQLTPTLVSLLEVIEPEVLYAG
YDSSVPDSTWRIMT[(pT)]LNMLGG[COOH]
ID:GRpS524pS562_Acetyl

A3059-1 10 mg
Thermo SCIENTIFIC
800-874-3723 or 815-968-0747 • FOR RESEARCH USE ONLY
3747 N. Meridian Rd., Rockford, IL 61101 U.S.A.
www.thermoscientific.com/pierce

Column:	250X4.6mm 5u C18 120A
Solvent:	A:0.05%TFA in Water; B: 0.05 % TFA in ACN
ID :	CD-296/EQ-289

Chromatogram

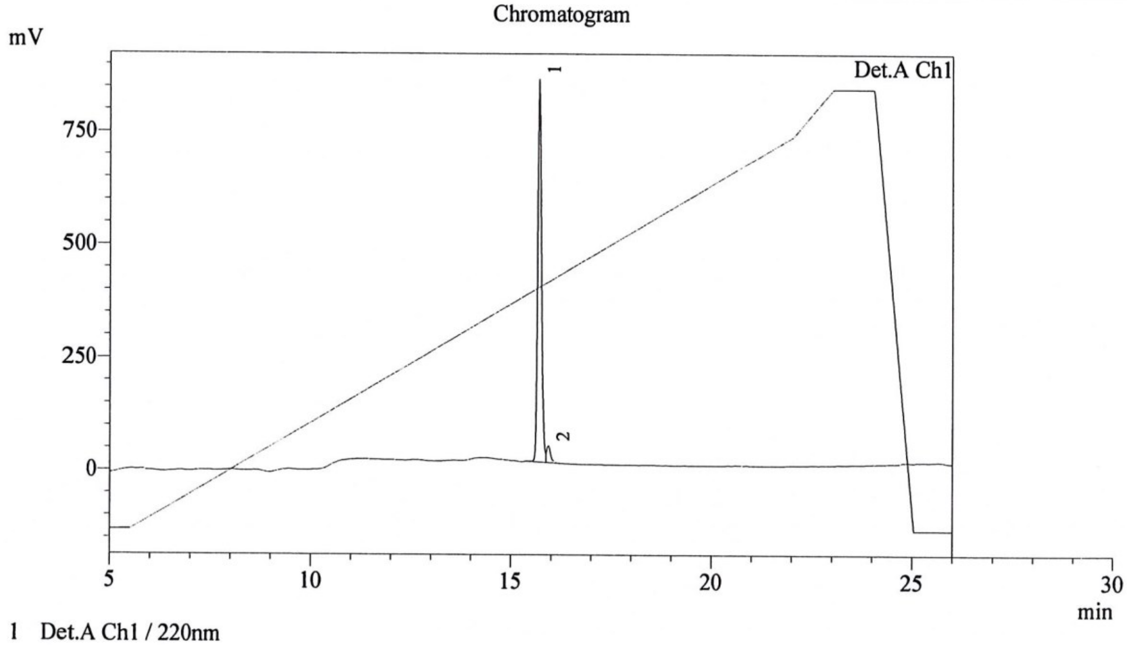

1 Det.A Ch1 / 220nm

PeakTable

Detector A Ch1 220nm

Peak#	Ret. Time	Area	Height	Height %	Area %
1	15.688	5593198	848848	95.712	95.661
2	15.918	253671	38027	4.288	4.339
Total				100.000	100.000

AB SCIEX Voyager System 6407

Voyager Spec #1=>SM11[BP = 5734.0, 23605]

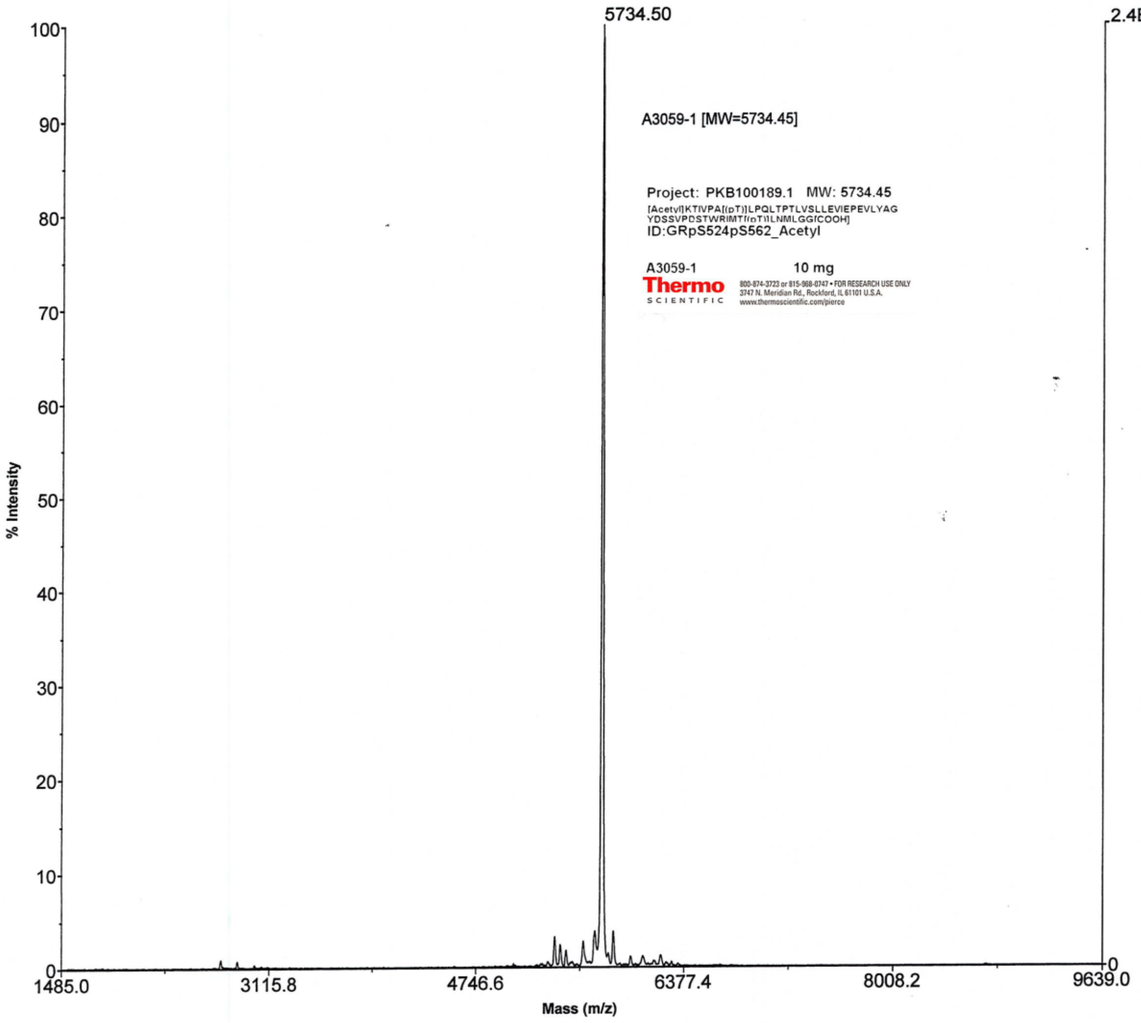

Mode of operation:	Linear
Extraction mode:	Delayed
Polarity:	Positive
Acquisition control:	Manual
Accelerating voltage:	20000 V
Grid voltage:	94%
Guide wire 0:	0.05%
Extraction delay time:	200 nsec
Acquisition mass range:	1000 -- 10000 Da
Number of laser shots:	200/spectrum
Laser intensity:	1800
Laser Rep Rate:	20.0 Hz
Calibration type:	Default
Calibration matrix:	a-Cyano-4-hydroxycinnamic acic
Low mass gate:	500 Da
Digitizer start time:	12.166
Bin size:	2 nsec
Number of data points:	13006
Vertical scale:	500 mV
Vertical offset:	0%
Input bandwidth:	500 MHz
Sample well:	91
Plate ID:	100 WELL PLATE
Serial number:	6407
Instrument name:	Voyager-DE PRO
Plate type filename:	C:\VOYAGER\100 well plate.plt
Lab name:	
Absolute x-position:	1774.2
Absolute y-position:	994.024
Relative x-position:	186.702
Relative y-posititon:	-593.476
Shots in spectrum:	48
Source pressure:	1.647e-007
Mirror pressure:	4.111e-008
TC2 pressure:	0.01426
TIS gate width:	30
TIS flight length:	678

Acquired: 17:05:00, December 06, 2019

Printed: 17:05, December 06, 2019

C:\VOYAGER\Data\2019\A3059-1 0011.dat

FITC labelled GR_pT524-pT562:

Sample Information

Acquired by	: Admin
Sample Name	: A3059-2
Sample ID	: A3059-2
Data Filename	: A3059-2
Method Filename	: ANAPEP_2.lcm

Project: PKB100189.2 MW: 6050.73
[FAM]KTIVPA[(pT)]LPQLTPTLVSLLEVIEPEVLYAGY
DSSVPDSTWRIMT[(pT)]LNMLGG[COOH]
ID:GRpS524pS562_Fluo

A3059-2 2 mg
Thermo SCIENTIFIC 800-874-3723 or 815-968-0747 • FOR RESEARCH USE ONLY
3747 N. Meridian Rd., Rockford, IL 61101 U.S.A.
www.thermoscientific.com/pierce

Column:	250X4.6mm 5u C18 120A
Solvent:	A:0.05%TFA in Water; B: 0.05 % TFA in ACN
ID :	CD-296/EQ-289

Chromatogram

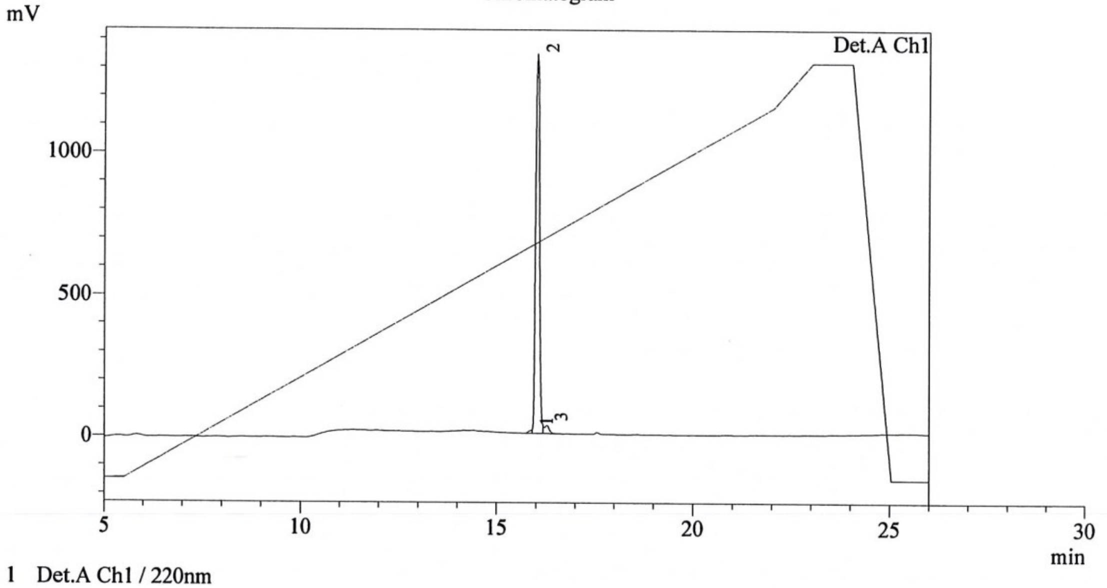

1 Det.A Ch1 / 220nm

PeakTable

Detector A Ch1 220nm

Peak#	Ret. Time	Area	Height	Height %	Area %
1	15.900	67013	10556	0.768	0.681
2	16.021	9520823	1336350	97.202	96.712
3	16.274	256646	27912	2.030	2.607
Total				100.000	100.000

AB SCIEX Voyager System 6407

Voyager Spec #1=>SM11=>SM13[BP = 6050.3, 13162]

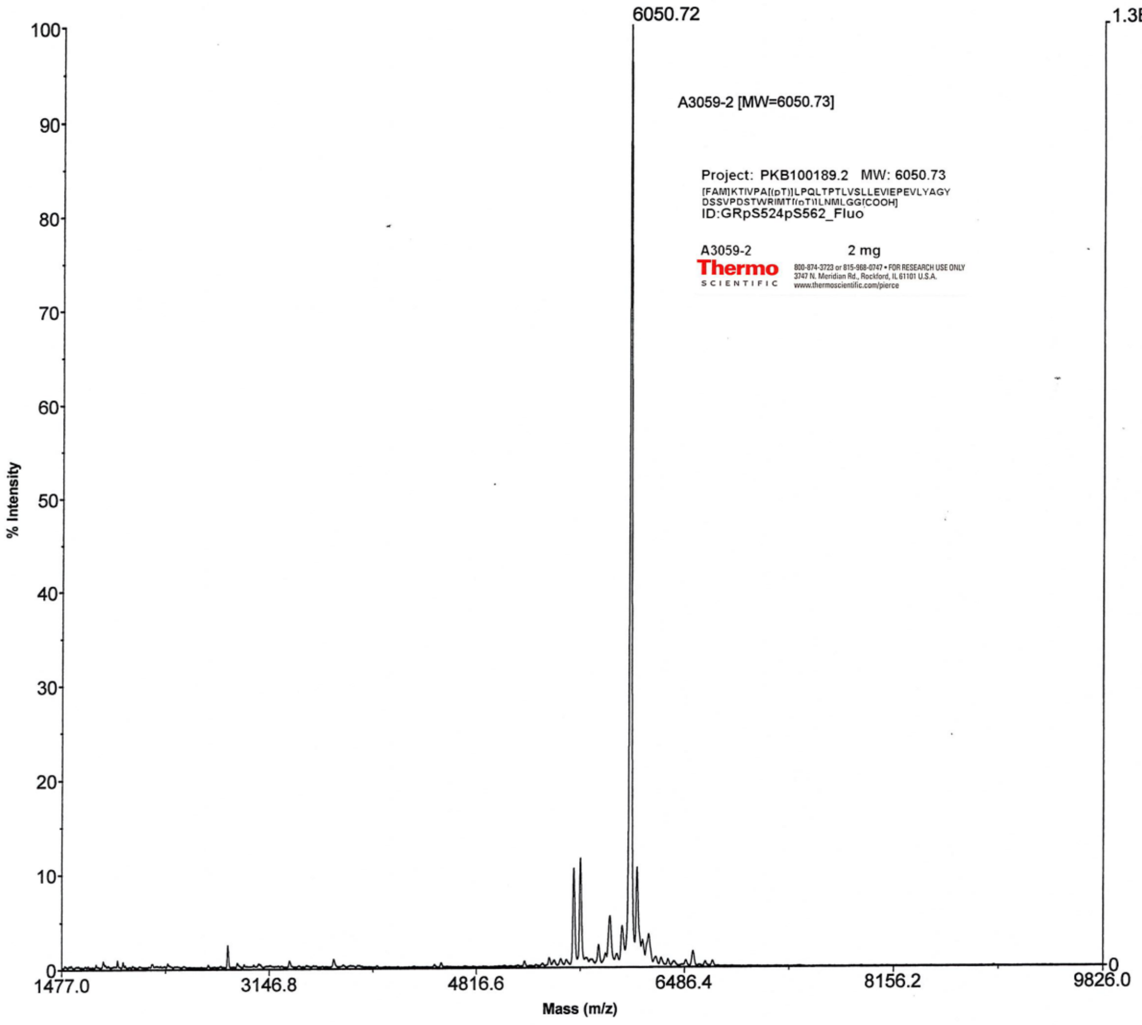

Mode of operation:	Linear
Extraction mode:	Delayed
Polarity:	Positive
Acquisition control:	Manual
Accelerating voltage:	20000 V
Grid voltage:	94%
Guide wire 0:	0.05%
Extraction delay time:	200 nsec
Acquisition mass range:	1000 -- 10000 Da
Number of laser shots:	200/spectrum
Laser intensity:	1800
Laser Rep Rate:	20.0 Hz
Calibration type:	Default
Calibration matrix:	a-Cyano-4-hydroxycinnamic acic
Low mass gate:	500 Da
Digitizer start time:	11.624
Bin size:	2 nsec
Number of data points:	12421
Vertical scale:	500 mV
Vertical offset:	0%
Input bandwidth:	500 MHz
Sample well:	92
Plate ID:	100 WELL PLATE
Serial number:	6407
Instrument name:	Voyager-DE PRO
Plate type filename:	C:\VOYAGER\100 well plate.plt
Lab name:	
Absolute x-position:	6703.62
Absolute y-position:	1693.99
Relative x-position:	36.1152
Relative y-posititon:	106.487
Shots in spectrum:	99
Source pressure:	1.637e-007
Mirror pressure:	4.074e-008
TC2 pressure:	0.01426
TIS gate width:	30
TIS flight length:	678

Acquired: 17:07:00, December 06, 2019

C:\VOYAGER\Data\2019\A3059-2_0002.dat

Printed: 17:07, December 06, 2019
